# Alkene Diversification through a Molecular Recasting Strategy Enabled by *N*-Heterocyclic Carbene and Photoredox Dual Catalysis

**DOI:** 10.34133/research.1010

**Published:** 2025-12-03

**Authors:** Qing-Zhu Li, Mei-Hao He, Peng-Tao Wang, Dong-Yang Xiang, Long-Hai Hong, Rong Zeng, Jun-Long Li

**Affiliations:** ^1^ Anti-infective Agent Creation Engineering Research Centre of Sichuan Province, Sichuan Industrial Institute of Antibiotics, School of Pharmacy, Chengdu University, Chengdu 610106, China.; ^2^ Key Laboratory of Drug-Targeting and Drug Delivery System of the Education Ministry and Sichuan Province, and Sichuan Research Center for Drug Precision Industrial Technology, West China School of Pharmacy, Sichuan University, Chengdu 610041, China.

## Abstract

Alkenes are fundamental structural motifs in organic synthesis, valued for their prevalence in natural products and their broad reactivity profile. While traditional de novo synthesis offers access to diverse alkene frameworks, strategies enabling the direct structural remodeling of alkenes offer more concise and efficient alternatives, yet remain underdeveloped. Herein, we present a molecular recasting strategy for alkene diversification, enabled by *N*-heterocyclic carbene/photoredox dual catalysis. The transformation involves a sequential dealkenylative acylation and realkenylation cascade, realizing precise and modular reassembly of a broad spectrum of alkenes under mild conditions. This approach demonstrates exceptional generality, accommodating up to trisubstituted alkenes, and supports interconversion among diverse alkene frameworks, as well as late-stage functionalization of complex bioactive molecules. Mechanistic investigations reveal a radical-mediated catalytic pathway and shed light on the synergy of photoredox and *N*-heterocyclic carbene dual catalysis. This work provides a conceptually different and synthetically valuable platform for alkene editing.

## Introduction

Alkenes are among the most fundamental and synthetically valuable classes of compounds in organic chemistry [1]. Their diverse reactivity and well-established transformation pathways have made them indispensable feedstocks in the synthesis of a wide array of fine chemicals, functional materials, and pharmaceutical agents [2–8]. Beyond their role as versatile synthetic building blocks, alkenes are also widely embedded in biologically active natural products and therapeutic molecules. Indeed, alkene motifs are often essential to the bioactivity of various compounds, including antibiotics, anticancer drugs, and pheromones [9–11]. Given their structural and functional significance, the development of novel strategies for rapid construction and diversification of alkenes continues to be a major objective in synthetic chemistry.

**Fig. 1.**
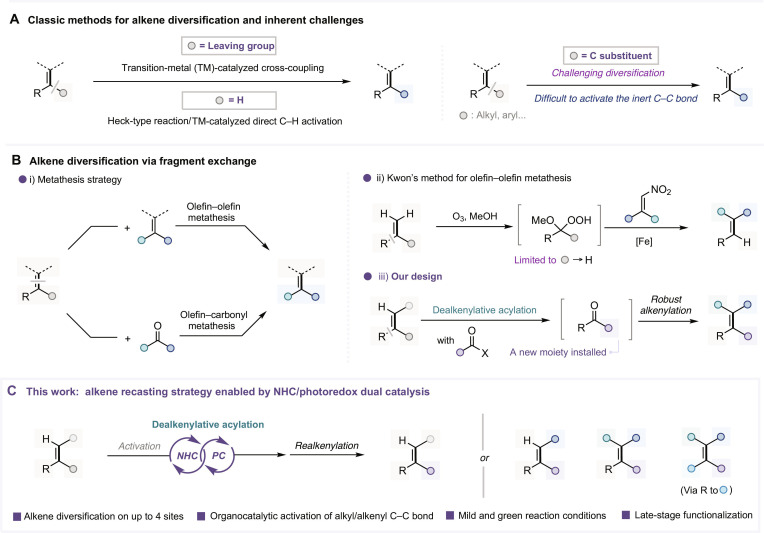
Background and research motivation for catalytic alkene diversification. (A) Classic methods for alkene diversification and inherent challenges. (B) Alkene diversification via fragment exchange. (C) This work: alkene recasting strategy enabled by *N*-heterocyclic carbene (NHC)/photoredox dual catalysis. MeOH, methanol; PC, photocatalyst.

In contrast to numerous strategies that focus on the de novo construction of alkene carbon–carbon double bonds [12–17], the direct modification of alkenes offers a more efficient approach to accessing new alkene analogs. This approach enables the rapid generation of structurally varied alkenes from readily available alkene starting materials, thereby substantially streamlining synthetic processes and improving overall efficiency. Notably, direct alkene-to-alkene transformations have been extensively explored, particularly for polarized alkenes. For instance, electron-rich alkenes can participate as nucleophiles in C(sp^2^)-H functionalization reactions [18–21], whereas electron-deficient alkenes are amenable to modification via Lewis base-catalyzed transformations, exemplified by the Baylis–Hillman reaction [22,23]. Furthermore, transition-metal-catalyzed strategies, including Heck-type reactions [24–27] and C(sp^2^)-H activation processes [28–30], have broadened the scope to encompass simple, electron-neutral alkenes. Complementarily, cross-coupling methodologies have enabled the replacement of C–X bonds on alkenes with diverse functional groups, providing powerful tools for alkene diversification [31–33]. However, despite these advances, selective modification of alkenyl carbon substituents—such as simple alkyl or aryl groups on alkenes—remains challenging, primarily due to the intrinsic difficulty associated with inert C–C bond cleavage. Traditional approaches are generally ineffective for this type of bond activation (Fig. 1A). To overcome this limitation, methods such as olefin–olefin [34–36] and olefin–carbonyl metathesis [37–41] have been developed, enabling structural remodeling through C–C double bond cleavage and recombination (Fig. 1B, i). In a complementary strategy, Kwon and co-workers [42–50] introduced an elegant method involving ozone-mediated oxidation followed by transition-metal-catalyzed hemolytic C–C bond cleavage, offering a new paradigm for alkene activation. More recently, this strategy has been adapted to olefin–olefin metathesis, although its application is primarily limited to the synthesis of alkenes bearing up to 3 substituents (Fig. 1B, ii) [51]. Inspired by these advances, we envisioned an alkene recasting strategy: a dealkenylative acylation to form ketone intermediates, followed by olefination to regenerate the carbon–carbon double bond. This approach enables the modular and programmable reconstruction of alkene structures through sequential bond cleavage and formation (Fig. 1B, iii).

Building on our ongoing interest in radical *N*-heterocyclic carbene (NHC) organocatalysis [52–64], we herein report an efficient molecular recasting strategy for alkene diversification. This approach employs NHC/photoredox dual catalysis [65,66] to achieve dealkenylative radical acylation, followed by realkenylation, thereby enabling modular diversification at up to 3 substituent sites. Furthermore, additional functionalization of the ketone intermediate provides the opportunity to modify all 4 substituent positions. This strategy exhibits excellent substrate scope and functional group tolerance and can be successfully applied to the late-stage modification of complex bioactive molecules. Moreover, mechanistic studies further illuminate the radical-based pathway and key features of the dual catalytic cycle (Fig. 1C).

## Results and Discussion

### Optimization of the reaction conditions

**Fig. 2.**
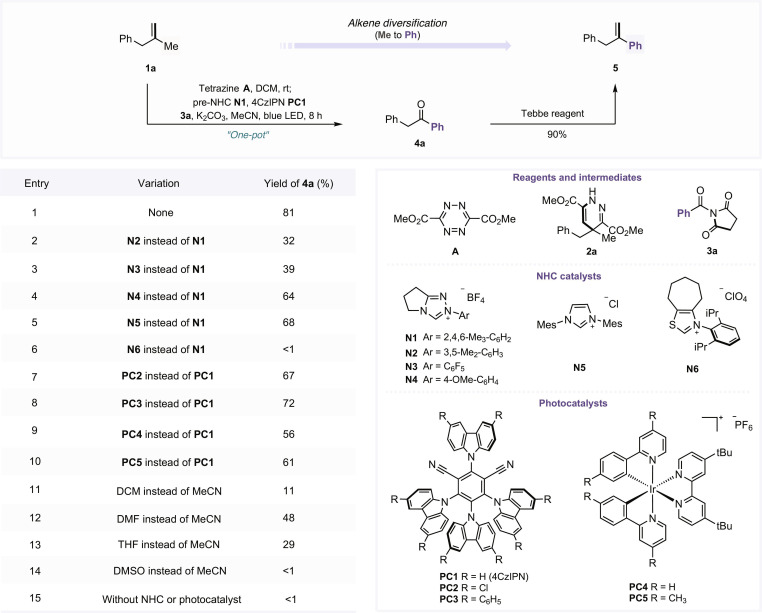
Reaction development. The reactions were conducted with 1a (0.1 mmol) and A (0.11 mmol) in dichloromethane (DCM) at room temperature for 7 h, and then the solvent was removed. Without purification, 3a (0.2 mmol), NHC catalyst N (20 mol %), photocatalyst PC (2 mol %), K_2_CO_3_ (0.20 mmol), and the solvent (1 ml) were added, and the mixture was reacted at room temperature under blue light-emitting diode (LED) irradiation for 8 h; nuclear magnetic resonance (NMR) yield using CH_2_Br_2_ as the internal standard. rt, room temperature; 4CzIPN, 1,2,3,5-tetrakis(carbazol-9-yl)-4,6-dicyanobenzene; MeCN, acetonitrile; DMF, dimethylformamide; THF, tetrahydrofuran; DMSO, dimethyl sulfoxide.

To validate our proposed alkene recasting strategy, we initiated a proof-of-concept study involving the transformation of a methyl substituent into a phenyl ring on a simple alkene model substrate (**1a**). We first conducted extensive screening of reaction parameters for the dealkenylative acylation step (see Tables S1 to S5), and the optimized conditions are summarized in Fig. 2. In this process, alkene **1a** was activated by tetrazine **A**, generating the pro-aromatic 1,4-dihydropyridazine intermediate **2a** [67,68]. Subsequent C–C bond cleavage and radical acylation with acyl-transfer reagent **3a** proceeded smoothly under the dual catalysis of a triazolium NHC (**N1**) and the photocatalyst 1,2,3,5-tetrakis(carbazol-9-yl)-4,6-dicyanobenzene (4CzIPN, **PC1**). Under these conditions, the desired ketone **4a** was obtained in 81% overall yield in a one-pot manner (entry 1). In contrast, alternative triazolium NHC catalysts (**N2** to **N4**) resulted in lower efficiencies (entries 2 to 4). Similarly, switching to the imidazolium-based NHC precursor **N5** afforded a reduced yield of 68% (entry 5), while the thiazolium variant **N6** proved entirely ineffective, delivering no detectable product (entry 6). Evaluation of alternative photocatalysts revealed that structural modifications to 4CzIPN (e.g., **PC2** and **PC3**) failed to enhance the reaction outcome (entries 7 and 8), and the use of Ir(III)-based complexes (**PC4** and **PC5**) resulted in slightly lower yields (entries 9 and 10). A solvent screen further confirmed that acetonitrile was optimal, as dichloromethane (DCM), dimethylformamide, tetrahydrofuran, and dimethyl sulfoxide all led to diminished efficiency (entries 11 to 14). As expected, the reaction did not proceed in the absence of either the NHC catalyst or photocatalyst (entry 15). Finally, the resulting ketone intermediate could be rapidly converted to alkene **5** in 90% yield using the Tebbe reagent, thereby enabling direct access to a phenyl-substituted olefin.

### Scope of the reaction

**Fig. 3.**
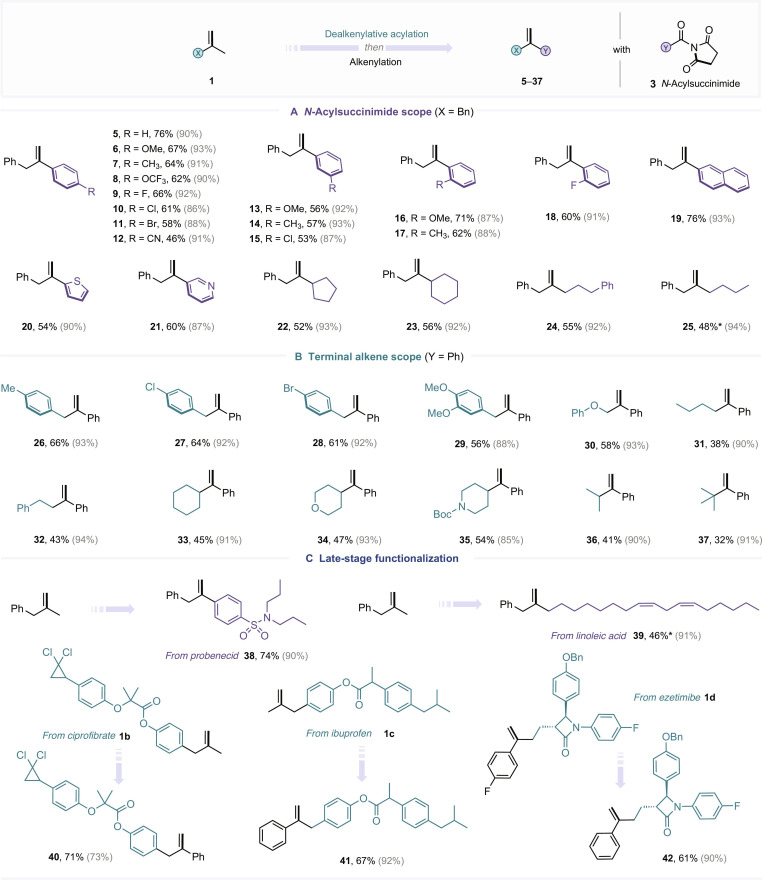
Substrate scope of the alkene recasting strategy. (A) *N*-Acylsuccinimide scope. (B) Terminal alkene scope. (C) Late-stage functionalization. See Fig. 2 and the Supplementary Materials for the detailed procedure; isolated yields are presented; the yield outside the parentheses refers to the one-pot transformation from alkene to ketone; the yield in the parentheses refers to the alkenylation step. * indicates that *N*-acylimidazole was used as the acyl-transfer reagent.

With the optimized conditions established, we next undertook a systematic investigation of the generality and limitations of this alkene recasting strategy (Fig. 3). Given the impressive efficiency observed in the final alkenylation step, our investigation primarily focused on evaluating the performance of the preceding dealkenylative acylation. As illustrated in Fig. 3A, a broad array of aryl-substituted *N*-acylsuccinimides **3** bearing diverse electronic and steric profiles smoothly reacted with methyl alkene **1a**, affording aryl ketones **5** to **18** in isolated yields ranging from 46% to 76%. The protocol was also effective with *N*-acylsuccinimides bearing a naphthyl group (**19**), as well as heteroaryl substituents such as thienyl and pyridinyl rings (**20** and **21**). Furthermore, both cyclic and linear alkyl-substituted *N*-acylsuccinimides engaged effectively in this transformation, delivering the corresponding products **22** to **25**. We then turned to examine the reactivity of a variety of terminal alkenes (Fig. 3B). Benzyl-substituted alkenes bearing different aryl groups performed efficiently, likely due to the resonance stabilization of the resulting benzylic radicals (**26** to **29**). Notably, replacing the aryl group with an ether moiety as the radical-stabilizing unit (**30**) still enabled productive acylation. Remarkably, even unactivated terminal alkenes lacking stabilizing substituents were viable substrates. Despite a modest decrease in yields, these simple alkenes bearing primary, secondary, or tertiary alkyl groups (**31** to **37**) successfully underwent the dealkenylative acylation, underscoring the broad functional group tolerance and robustness of this strategy. To further highlight the synthetic utility and broad applicability of this strategy, we applied the optimized reaction conditions to the late-stage functionalization of structurally complex, bioactive molecules. As shown in Fig. 3C, the methyl group in model alkene **1a** could be replaced with diverse pharmacophore-derived fragments. For example, substrates derived from probenecid (**38**) or linoleic acid (**39**) participated smoothly in the transformation. Moreover, the method proved amenable to structural elaboration of alkenes derived from ciprofibrate (**40**), ibuprofen (**41**), and ezetimibe (**42**), highlighting the compatibility of this protocol with medicinally relevant scaffolds and its potential in drug modification and analog development.

### The synthetic utility of the alkene recasting strategy

**Fig. 4.**
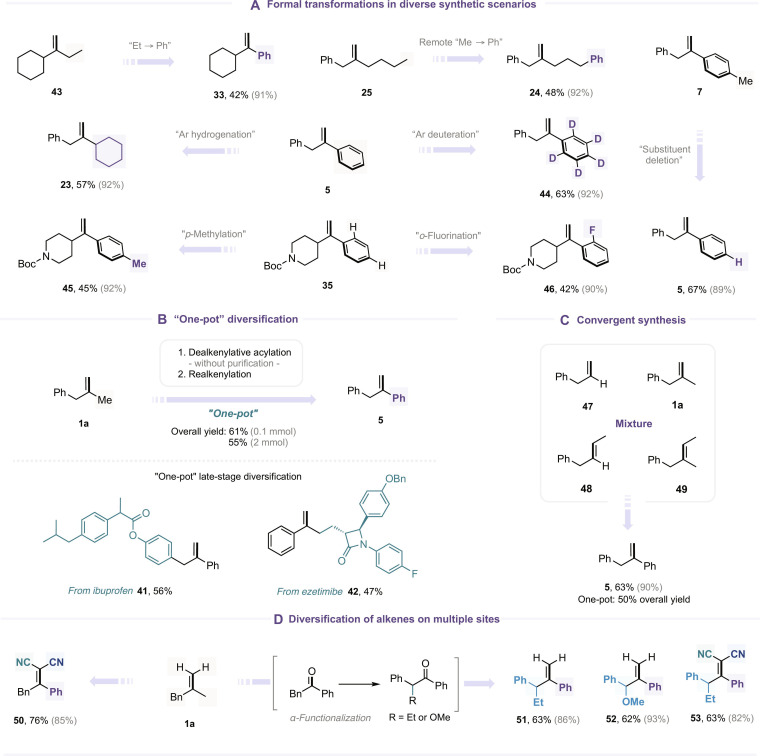
Further demonstration of the synthetic significance of the alkene recasting strategy. (A) Formal transformations in diverse synthetic scenarios. (B) “One-pot” diversification. (C) Convergent synthesis. (D) Diversification of alkenes on multiple sites. The yield outside the parentheses refers to the one-pot transformation from alkene to ketone; the yield in the parentheses refers to the alkenylation step.

The synthetic utility of this alkene recasting strategy was further demonstrated by employing alkene substrates bearing substituents beyond the simple methyl group. These alkenes successfully engaged in fragment exchange reactions with diverse acyl-transfer reagents, enabling a range of synthetically interesting transformations. As illustrated in Fig. 4A, an ethyl-substituted alkene was efficiently transformed into a phenyl-substituted alkene (**43** → **33**), while the terminal methyl group could be formally replaced with a phenyl fragment (**25** → **24**). Remarkably, the method also allowed the phenyl alkene to be converted into a cyclohexyl alkene (**5** → **23**), effectively mimicking a challenging chemoselective hydrogenation of an aromatic ring. Subsequently, we demonstrated that the aryl-to-aryl fragment exchange is also feasible, and we can thus realize the formal editing of aryl substituents on alkenes. For instance, we achieved the formal deletion of a methyl group (**7** → **5**), selective deuteration of aryl C(sp^2^)-H bonds (**5** → **44**), and diverse site-selective installation of functional groups on the aromatic ring (**35** → **45** and **35** → **46**). Next, we found that the ketone intermediate **4a**, obtained through dealkenylative acylation, could be directly employed in the subsequent realkenylation step without prior purification. The one-pot protocol afforded product **5** in 61% overall yield, thereby enabling rapid alkene structural editing. This streamlined protocol was readily scalable and applicable to the late-stage functionalization of pharmaceuticals (Fig. 4B). The reaction with a mixture of different alkenes, including **1a**, monosubstituted (**47**), 1,2-disubstituted alkenes (**48**), and trisubstituted alkenes (**49**), offered the same alkene product **5**. This convergent reaction could also be conducted in a one-pot procedure, delivering **5** in 50% overall yield (Fig. 4C).

In addition, as depicted in Fig. 4D, we explored the multi-site diversification of alkene **1a** to showcase the full potential of this alkene recasting strategy. Using malononitrile as the alkenylation reagent, the terminal alkenyl C–H bonds were successfully modified, affording a tetrasubstituted alkene (**50**). Notably, by introducing an additional transformation at the α-position of ketone intermediate **4a** prior to alkenylation, it was possible to install up to 4 distinct substituents on the alkene framework (**51** to **53**), highlighting the modularity and synthetic flexibility of this approach (for additional examples of alkene diversification on multiple sites, see the Supplementary Materials).

### Mechanistic investigations and the proposed mechanism

**Fig. 5.**
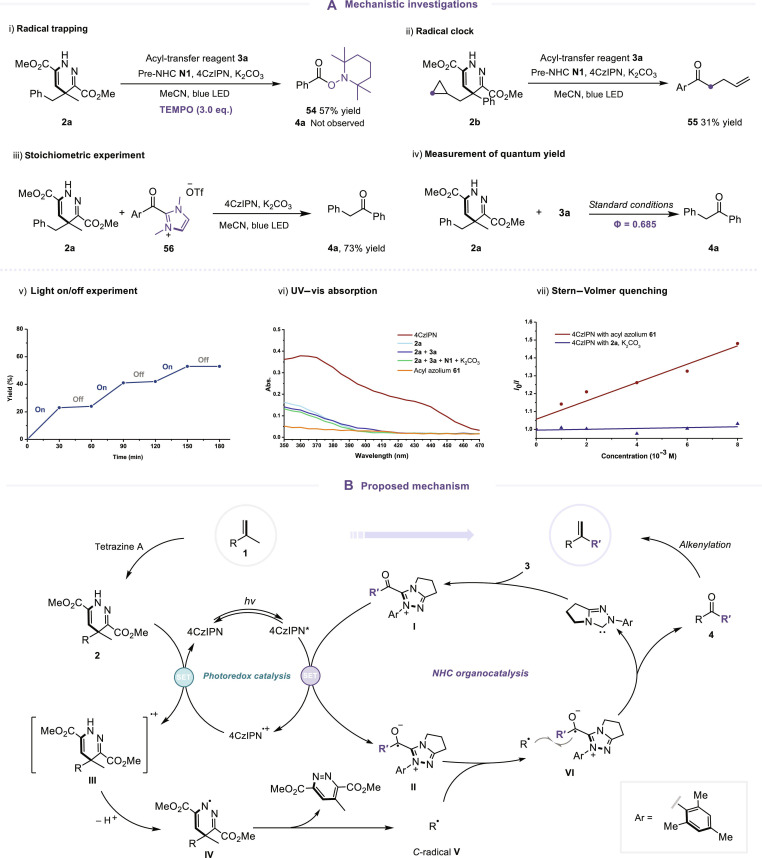
Mechanistic investigations and the proposed mechanism. (A) Mechanistic investigations. (B) Proposed mechanism. TEMPO, 2,2,6,6-tetramethyl-piperidinyloxy; UV–vis, ultraviolet–visible; SET, single-electron transfer.

To better elucidate the underlying mechanism of the key dealkenylative acylation step, we conducted a series of mechanistic studies, as outlined in Fig. 5A. We began by isolating intermediate **2a**, derived from the alkene substrate, and subjected it to the standard catalytic conditions in the presence of the radical scavenger 2,2,6,6-tetramethyl-piperidinyloxy (TEMPO). Under these conditions, ketone formation was completely suppressed, and the TEMPO-trapped adduct **54** was obtained, providing compelling evidence for a radical-based process (Fig. 5A, i). Further support came from a radical clock experiment employing a cyclopropyl-substituted substrate **2b**, which underwent ring opening to give product **55** in 31% yield, consistent with radical intermediacy (Fig. 5A, ii).

Next, to assess the role of the NHC-derived acyl azolium species, we replaced both the NHC precursor and **3a** with a pre-synthesized acyl azolium **56**. Gratifyingly, this surrogate also furnished the ketone product **4a** under otherwise identical conditions, indicating that the acyl azolium intermediate is catalytically competent and likely plays a central role in the dual catalytic cycle (Fig. 5A, iii). Then, we turned our attention to the photoredox catalytic process. Quantum yield measurement gave a value of Φ = 0.685, suggesting a photocatalytic rather than a chain-propagated mechanism (Fig. 5A, iv). This conclusion was further corroborated by a light on/off experiment, in which product **4a** was formed only during irradiation periods (Fig. 5A, v). Ultraviolet–visible absorption analysis revealed that only the photocatalyst 4CzIPN exhibited remarkable absorption in the visible region, while the substrates, NHC catalysts, and acyl azolium intermediates did not display marked absorption individually or in combination (Fig. 5A, vi). Furthermore, Stern–Volmer luminescence quenching experiments demonstrated that the excited state of 4CzIPN is selectively quenched by acyl azolium **56**, but not by **2a** (Fig. 5A, vii). These results point toward a single-electron transfer event from the excited photocatalyst 4CzIPN* (*E*_1/2_PC^+**·**^/PC* = −1.18 V vs. saturated calomel electrode [SCE]) [69] to the acyl azolium species acyl azolium **56** (*E*_red_ = −1.13 V vs. SCE) [70]. The subsequent oxidation of the intermediate **2** (*E*_ox_ = +1.42 V vs. SCE) [67,68] by 4CzIPN^+**·**^ (*E*_1/2_PC^+**·**^/PC = +1.49 V vs. SCE) [69], given its favorable redox potential, is also thermodynamically feasible.

Drawing from the experimental findings, we propose a mechanistic pathway for this alkene recasting protocol, as depicted in Fig. 5B. The cycle is initiated by the formation of acyl azolium intermediate **I** via NHC organocatalysis. Upon photoexcitation, the photocatalyst 4CzIPN reaches its excited state (4CzIPN*), which then undergoes a single-electron transfer with the acyl azolium to generate the ketyl radical species **II** alongside the oxidized photocatalyst 4CzIPN^+**·**^. Concurrently, the in situ formed dihydropyridazine intermediate **2**, originating from the alkene substrate **1**, donates an electron to 4CzIPN^+**·**^, thereby restoring the ground-state photocatalyst and generating radical species **III**. A subsequent deprotonation of **III** leads to the formation of the nitrogen-centered radical **IV**, which undergoes fragmentation to yield the carbon-centered radical **V**. These 2 radical species then engage in a radical–radical cross-coupling event, constructing the key C–C bond while simultaneously releasing the NHC catalyst to furnish the ketone intermediate **4**. This ketone is then subjected to an olefination process, completing the rapid transformation and delivering the final alkenylated product. Through this sequence, a formal editing of the alkene framework is accomplished with high structural precision.

In conclusion, we have developed a modular alkene recasting strategy based on a dealkenylative acylation and realkenylation cascade, enabled by NHC/photoredox dual catalysis. This protocol allows for efficient and selective diversification of alkenes, providing access to structurally diverse derivatives from a broad range of alkene substrates. The present approach demonstrates high functional group tolerance and broad applicability, including the late-stage modification of pharmaceutically relevant molecules. Mechanistic studies support the proposed dual catalytic cycle and underscore the role of radical intermediates in driving the transformation. This work expands the toolbox for NHC-catalyzed molecular editing, and further developments along this line are currently in progress.

## Materials and Methods

### General information

Commercial reagents and solvents were obtained from Adamas-beta, Aldrich Chemical Co., Alfa Aesar, Macklin, Energy Chemical, and Leyan. Analytical thin-layer chromatography was performed on silica gel HSGF_254_ glass plates (purchased from Jiangyou Silica Gel Development Co., Ltd, Yantai, China) containing a 254-nm fluorescent indicator. Flash column chromatography was performed over silica gel (40 to 45 μm, 300 to 400 mesh). ^1^H nuclear magnetic resonance (NMR) and ^13^C NMR spectra were recorded at 25 °C on a JEOL JNM-ECZ600R/S1 spectrometer. High-resolution mass spectrometry was performed on an Agilent 6230 time-of-flight liquid chromatography/mass spectrometry instrument or a Waters SYNAPT G2 mass spectrometer by using an electrospray ionization source analyzed by quadrupole time-of-flight mass spectrometry. Melting points were determined on a SGW X-4 digital melting point apparatus, and temperatures were not corrected.

### Procedure for “one-pot” diversification

A dry glass tube was charged with alkene **1a** (0.1 mmol), tetrazine **A** (0.11 mmol), and DCM (2 ml). Then, the resulting mixture was stirred for 7 h at room temperature. After the reaction finished, the reaction mixture was concentrated in vacuum to give the crude intermediate **2a**, and then *N*-acylsuccinimide **3a** (0.2 mmol), NHC **N1** (0.02 mmol), **PC1** (0.002 mmol), and K_2_CO_3_ (0.2 mmol) were added. The reaction tube was subjected to 3 cycles of pressurization and depressurization using dry Ar. Afterward, 1 ml of methyl cyanide was added, and the reaction mixture was stirred for 8 h under the irradiation of blue light-emitting diodes. Then, the mixture was concentrated in vacuum. Subsequently, the glass tube was subjected to 3 cycles of pressurization and depressurization using dry Ar. Under the protection of an Ar atmosphere, toluene (1 ml), the Tebbe reagent (0.3 mmol) and pyridine (0.3 mmol) were successively added. The reaction was stirred for 5 h at 40 °C. Then, the reaction mixture was diluted with water and extracted with DCM. The combined organic phases were washed with brine, dried over anhydrous Na_2_SO_4_, filtered, and concentrated in vacuum. The crude residue was then purified by column chromatography on silica gel eluting with petroleum ether/ethyl acetate to provide the alkene product **5**.

## Data Availability

The data supporting the findings of this study are available within this article and its Supplementary Materials or from the authors on reasonable request.
