# Supplementary material for: Alkene Diversification through a Molecular Recasting Strategy Enabled by N-Heterocyclic Carbene and Photoredox Dual Catalysis
Source: Research (Wash D C). 2025 Dec 3;8:1010. doi: 10.34133/research.1010 (PMC12673803; doi:10.34133/research.1010)
Supplement: Supplementary 1 — Supplementary Text Tables S1 to S5 Figs. S1 and S2 [file research.1010.f1.pdf]

Supplementary Materials for

Alkene Diversification through a Molecular Recasting Strategy Enabled by *N*-Heterocyclic Carbene and Photoredox Dual Catalysis

Qing-Zhu Li *et al*.

Corresponding author. Jun-Long Li: lijunlong709@hotmail.com

This PDF file includes:

Supplementary Text

Table S1 to S5

Fig. S1 to S2

References

Contents

1. General Information 3

2. Further Optimization Studies 5

3. General Procedure for Dealkenylative Acylation 10

4. General Procedure for the Alkenylation 29

5. The Synthetic Application 47

6. Mechanistic Studies 62

7. References and Notes 70

8. Copies of NMR Spectra 71

1. General Information

General Procedures.

All reactions were performed in over-dried or flame-dried reaction vessels, modified Schlenk flasks, or round-bottom flasks. The flasks were fitted with Teflon screw caps and reactions were conducted under an atmosphere of argon if needed. Gas-tight syringes with stainless steel needles were used to transfer air- and moisture-sensitive liquids. All moisture and/or air sensitive solid compounds were manipulated inside normal desiccators. Flash column chromatography was performed over silica gel (40 – 45 μm, 300 – 400 mesh).

Analytical thin layer chromatography (TLC) was performed on silica gel $HSGF_{254}$ glass plates (purchased from Jiangyou silica gel development Co., Ltd, Yantai, China) containing a 254 nm fluorescent indicator. TLC plates were visualized by exposure to short wave ultraviolet light (254 nm) or I_2 and to a solution of $KMnO_4$ (1 g of $KMnO_4$, 6 g of K_2CO_3 and 0.1 g of KOH in 100 mL of H_2O) or vanillin (2 g of vanillin and 4 mL of concentrated H_2SO_4 in 100 mL of EtOH) followed by heating.

Organic solutions were concentrated at 30 – 40 ℃ on rotary evaporators at ~80 mbar followed by drying on vacuum pump below 1 mbar. Reaction temperatures are reported as the temperature of the bath surrounding the vessel unless otherwise stated.

Materials.

Commercial reagents and solvents were were obtained from Adamas-beta, Aldrich Chemical Co., Alfa Aesar, Leyan, Macklin and Energy Chemical and used as received with the following exceptions: THF, 1,4-dioxane and toluene were purified by refluxing over Na-benzophenone under positive argon pressure followed by distillation.[1] *N*-acyl succinimides[2], *N*-acyl imidazoles[3], acyl azolium **56**[4], and tetrazine compound **A**[5] were prepared according to literature procedure.

Instrumentation.

- Proton nuclear magnetic resonance (^{1}H NMR) spectra were measured on a JEOL JNM-ECZ600R/S1 spectrometer at ambient temperature for ^{1}H at 600 MHz. Proton chemical shifts are reported in parts per million (δ scale), and are referenced using tetramethylsilane (TMS) as an internal standard or residual protium in the NMR solvent ($CDCl_3$: δ 7.26 ($CHCl_3$) or DMSO-d_6: δ 2.50 (CD_2HSOCD_3)). Data are reported as follows: chemical shift [multiplicity (s = singlet, d = doublet, t = triplet, q = quartet, m = multiplet, dd = dou-

blet of doublets, td = triplet of doublets, brs = broad singlet), coupling constant(s) (Hz), integration].

- Carbon-13 nuclear magnetic resonance (^{13}C NMR) spectra measured on a JEOL JNM-ECZ600R/S1 spectrometer at ambient temperature for ^{13}C at 151 MHz. Carbon chemical shifts are reported in parts per million (δ scale), and are referenced using the carbon resonances of the solvent (δ 77.00 ($CDCl_3$) or δ 39.52 (DMSO-d_6)). Data are reported as follows: chemical shift [multiplicity (if not singlet), assignment (C_q = fully substituted carbon)].

- High resolution mass spectra (HRMS) were performed on an Agilent 6230 time-of-flight (TOF) LC/MS instrument or a Waters SYNAPT G2 mass spectrometer by using an electrospray ionization (ESI) ionization source analyzed by quadrupole time-of-flight (Q-TOF). Melting points were determined on a SGW X-4 digital melting point apparatus and temperatures were not corrected.

- Melting points were determined on a SGW X-4 digital melting point apparatus using open glass capillaries and temperatures were not corrected, reported in degrees Celsius.

- Reaction setup: a custom-designed photoreaction setup was employed, featuring five blue LEDs (30 W, λ_{max} = 450 nm, purchased from XINXINGYUAN Co., Ltd.) arranged around the reaction vessel (as show in the following picture). The distance between the reaction tube and the nearest LED is 2.5 cm.

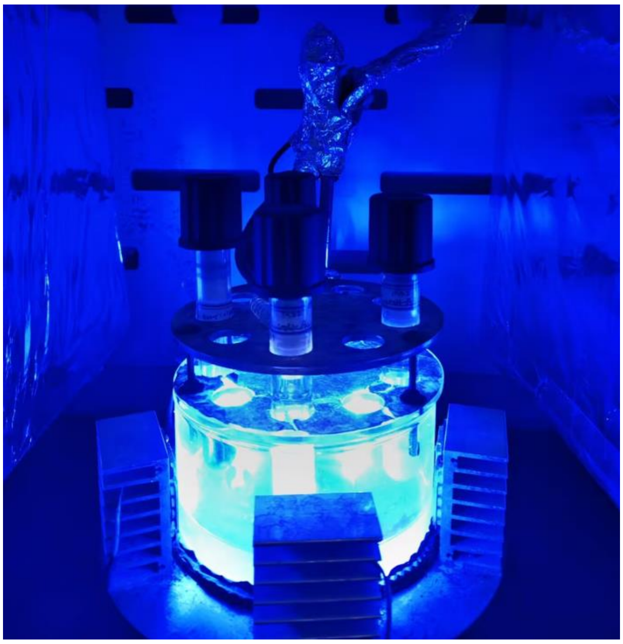

2. Further Optimization Studies

Table S1. Optimization of the base.[a]

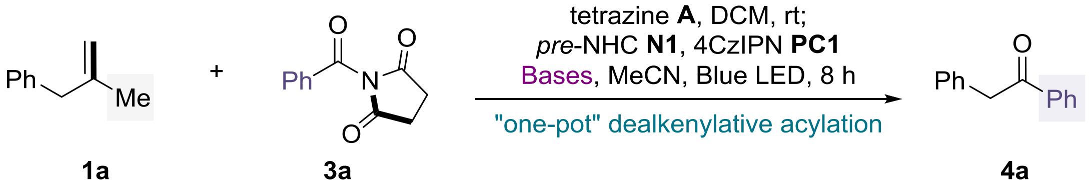

Entry	Base	Equiv.	Yield (%)[b]
1	K_2CO_3	2	81
2	Na_2CO_3	2	52
3	CH_3COONa	2	<1
4	KH_2PO_4	2	<1
5	K_3PO_4	2	11
6	KO*t*Bu	2	33
7	DBU	2	14
8	Et_3N	2	<1
9	Pyridine	2	<1
10	K_2CO_3	0.2	35
11	K_2CO_3	0.5	43
12	K_2CO_3	1	67
13	K_2CO_3	1.5	74
14	K_2CO_3	2.5	76

[a] The reactions were conducted with **1a** (0.1 mmol) and **A** (0.11 mmol) in DCM at room temperature for 7 hours and then removed the solvent. Without purification, **3a** (0.2 mmol), NHC **N1** (20 mol %), photocatalyst **PC1** (2 mol %), base and MeCN(1 mL) were added and the mixture was reacted at room temperature under blue LEDs irradiation for 8 h. [b] NMR yield using CH_2Br_2 as the internal standard.

Table S2. Optimization of the NHCs.[a]

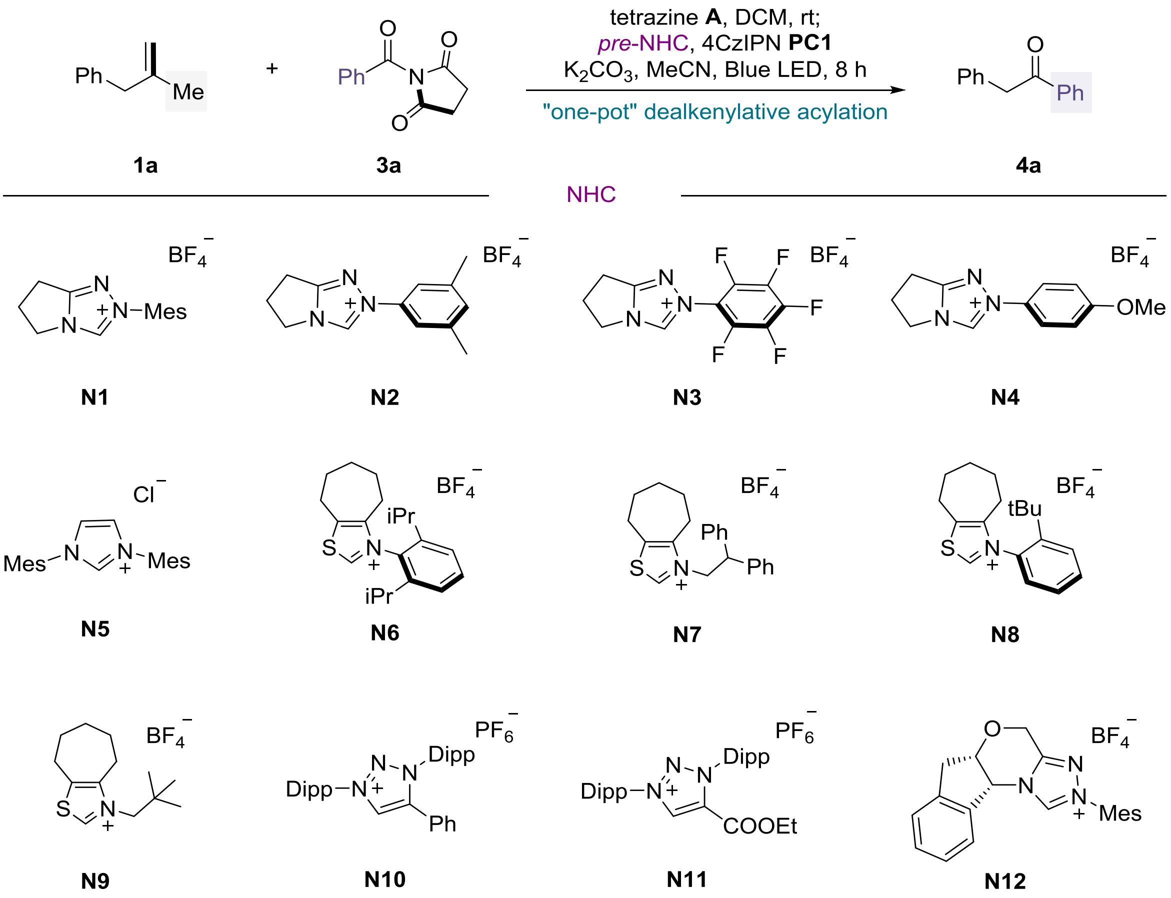

Entry	NHC	Yield (%)[b]	Entry	NHC	Yield (%)[b]
1	**N1**	81	7	**N7**	<1
2	**N2**	32	8	**N8**	<1
3	**N3**	39	9	**N9**	<1
4	**N4**	64	10	**N10**	24
5	**N5**	68	11	**N11**	8
6	**N6**	<1	12	**N12**	29

[a] The reactions were conducted with **1a** (0.1 mmol) and **A** (0.11 mmol) in DCM at room temperature for 7 hours and then removed the solvent. Without purification, **3a** (0.2 mmol), NHC catalyst **N** (20 mol %), photocatalyst **PC1** (2 mol %), K_2CO_3 (0.20 mmol) and MeCN (1 mL) was added and the mixture was reacted at room temperature under blue LEDs irradiation for 8 h. [b] NMR yield using CH_2Br_2 as the internal standard. Mes: 2, 4, 6-Me_3-C_6H_2.

Table S3. Optimization of photocatalysts.[a]

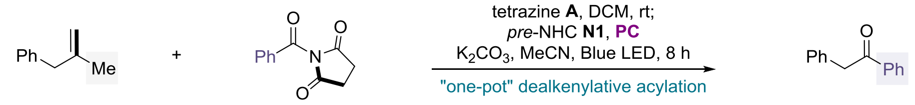

1a **3a** **4a**

Photocatalysts

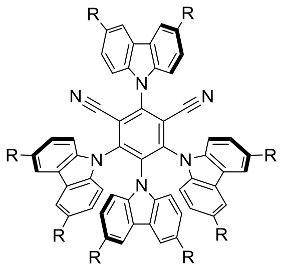

PC1 R = H (4CzIPN)
PC2 R = Cl
PC3 R = C_6H_5

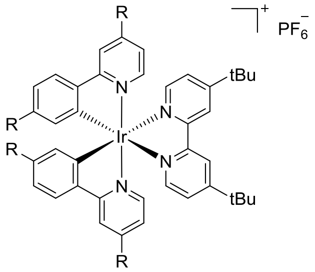

PC4 R = H
PC5 R = CH_3

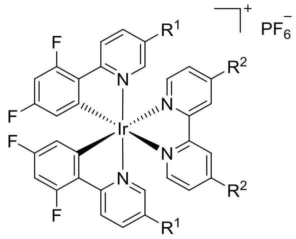

PC6 R^1 = H, R^2 = H
PC7 R^1 = CF3, R^2 = tBu
PC8 R^1 = CH_3, R^2 = tBu

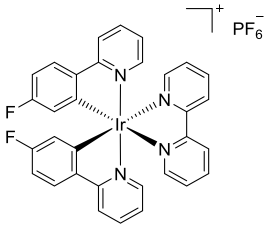

PC9

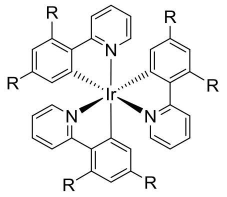

PC10 R = H
PC11 R = F

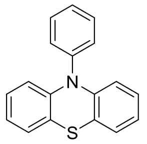

PC12

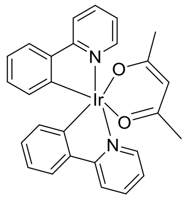

PC13

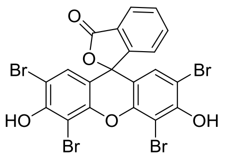

PC14

Entry	Photocatalysts	Yield (%)[b]	Entry	Photocatalysts	Yield (%)[b]
1	**PC1**	81	8	**PC8**	67
2	**PC2**	67	9	**PC9**	38
3	**PC3**	72	10	**PC10**	42
4	**PC4**	56	11	**PC11**	31
5	**PC5**	61	12	**PC12**	<1
6	**PC6**	53	13	**PC13**	26
7	**PC7**	58	14	**PC14**	<1

[a] The reactions were conducted with **1a** (0.1 mmol) and **A** (0.11 mmol) in DCM at room temperature for 7 hours and then removed the solvent. Without purification, **3a** (0.2 mmol), NHC **N1** (20 mol %), photocatalyst **PC** (2 mol %), K_2CO_3 (0.20 mmol) and MeCN (1 mL) was added and the mixture was reacted at room temperature under blue LEDs irradiation for 8 h. [b] NMR yield using CH_2Br_2 as the internal standard.

Table S4. Optimization of solvents.[a]

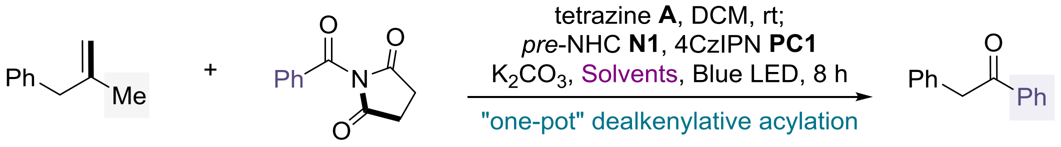

1a **3a** **4a**

Entry	Solvent	Yield(%)[b]	Entry	Solvent	Yield (%)[b]
1	MeCN	81	6	DMF	48
2	DCE	<1	7	DCM	11
3	1,4-dioxane	32	8	$CHCl_3$	52
4	toluene	25	9	THF	29
5	DMSO	<1	10	acetone	<1

[a] The reactions were conducted with **1a** (0.1 mmol) and **A** (0.11 mmol) in DCM at room temperature for 7 hours and then removed the solvent. Without purification, **3a** (0.2 mmol), NHC **N1** (20 mol %), photocatalyst **PC1** (2 mol %), K_2CO_3 (0.20 mmol) and solvents (1 mL) was added and the mixture was reacted at room temperature under blue LEDs irradiation for 8 h. [b] NMR yield using CH_2Br_2 as the internal standard.

Table S5. Optimization of leaving groups.[a]

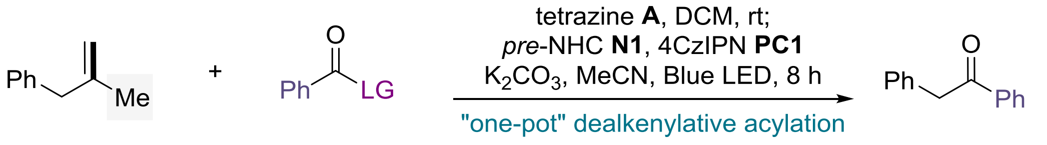

1a **4a**

LG

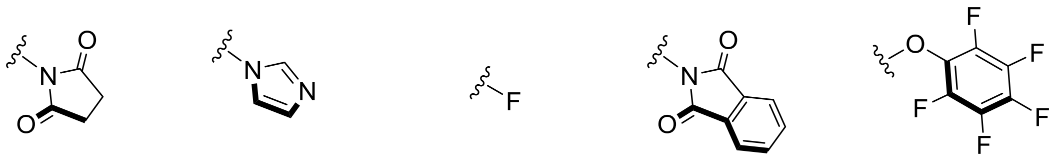

LG¹ **LG²** **LG³** **LG⁴** **LG⁵**

Entry	LG	Yield (%)[b]
**1**	**LG**^{1}	81
**2**	**LG**^{2}	63
**3**	**LG**^{3}	31
**4**	**LG**^{4}	72
**5**	**LG**^{5}	9

[a] The reactions were conducted with **1a** (0.1 mmol) and **A** (0.11 mmol) in DCM at room temperature for 7 hours and then removed the solvent. Without purification, **3** (0.2 mmol), NHC **N1** (20 mol %), photocatalyst **PC1** (2 mol %), K_2CO_3 (0.20 mmol) and solvents (1 mL) was added and the mixture was reacted at room temperature under blue LEDs irradiation for 8 h. [b] NMR yield using CH_2Br_2 as the internal standard.

Unsuccessful attempts

unsuccessful tetrazines

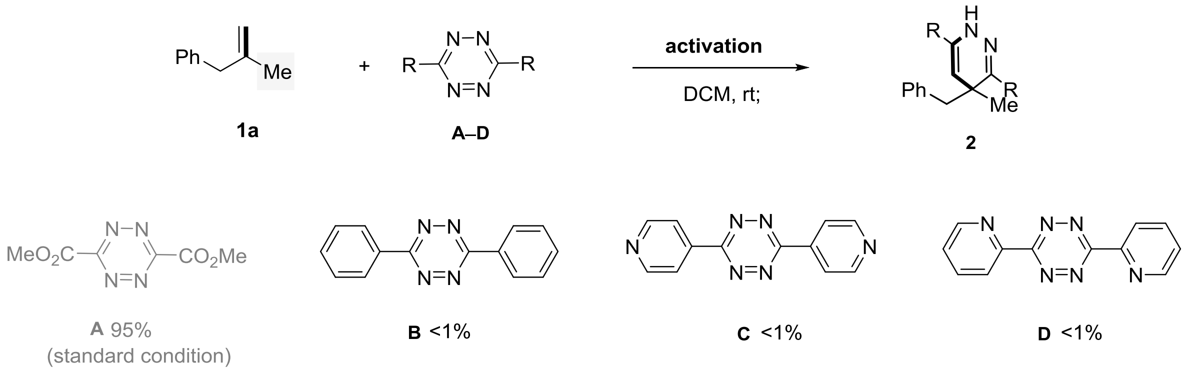

unsuccessful alkenes

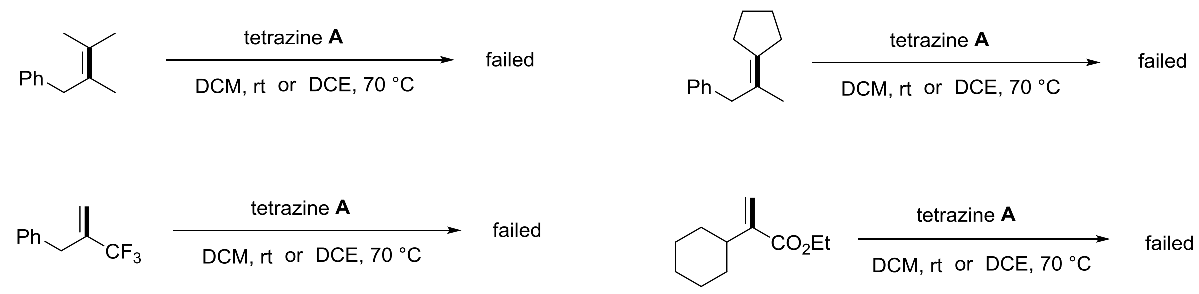

Unsaturated acyl-transfer reagent

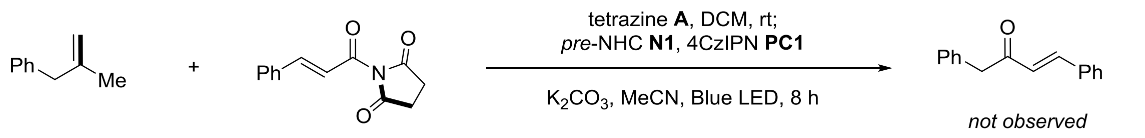

3. General Procedure for Dealkenylative Acylation

***General Procedure A**: The Dealkenylative Acylation of Alkenes*

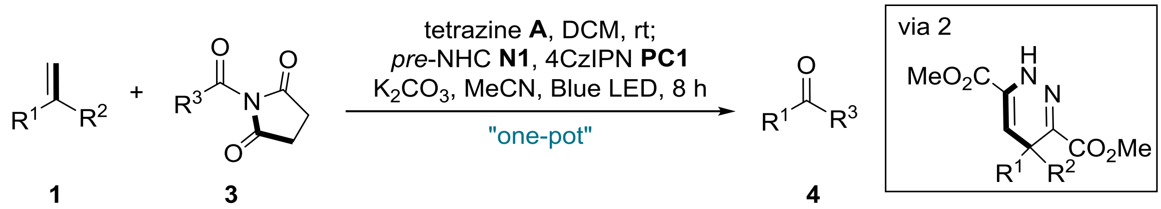

A dry glass tube was charged with alkene **1** (0.1 mmol), **A** (0.11 mmol), and DCM (2 mL). Then, the resulting mixture was stirred for 7 h at room temperature, monitored by TLC. After the reaction finished, the reaction mixture was concentrated in vacuum to give the crude intermediate **2**, then **3** (0.2 mmol), NHC **N1** (0.02 mmol), **PC1** (0.002 mmol) and K_2CO_3 (0.2 mmol) were added. The reaction tube was subjected to three cycles of pressurization and depressurization using dry Ar. Afterwards, 1 mL of MeCN was added, and the reaction mixture was stirred for 8 h under the irradiation of blue LEDs. Then the mixture was concentrated in vacuum, and purified by column chromatography on silica gel eluting from petroleum ether/ethyl acetate (400:1 to 100:1) to afford the corresponding ketone product **4**.

Figure S1. The structures of the terminal alkene substrates

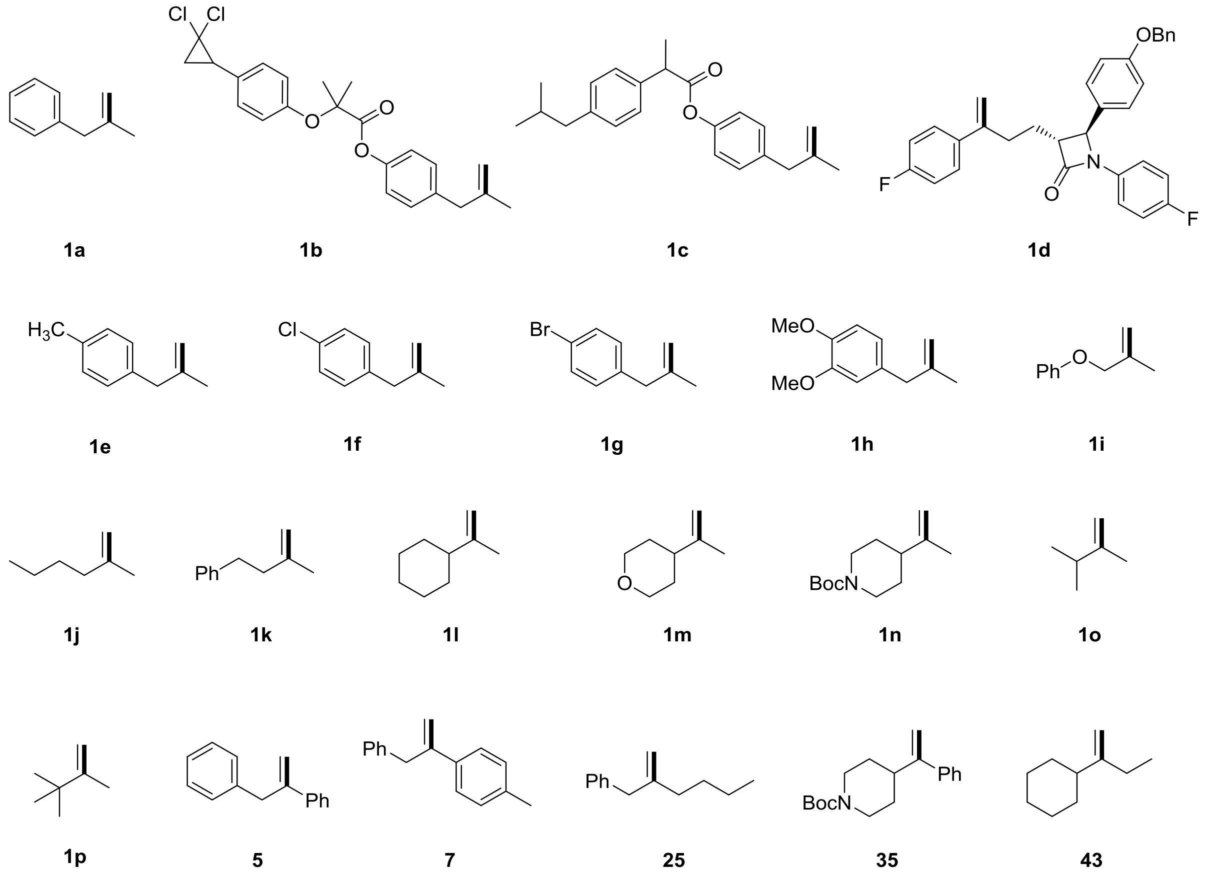

Figure S2. The structures of ketones **4**

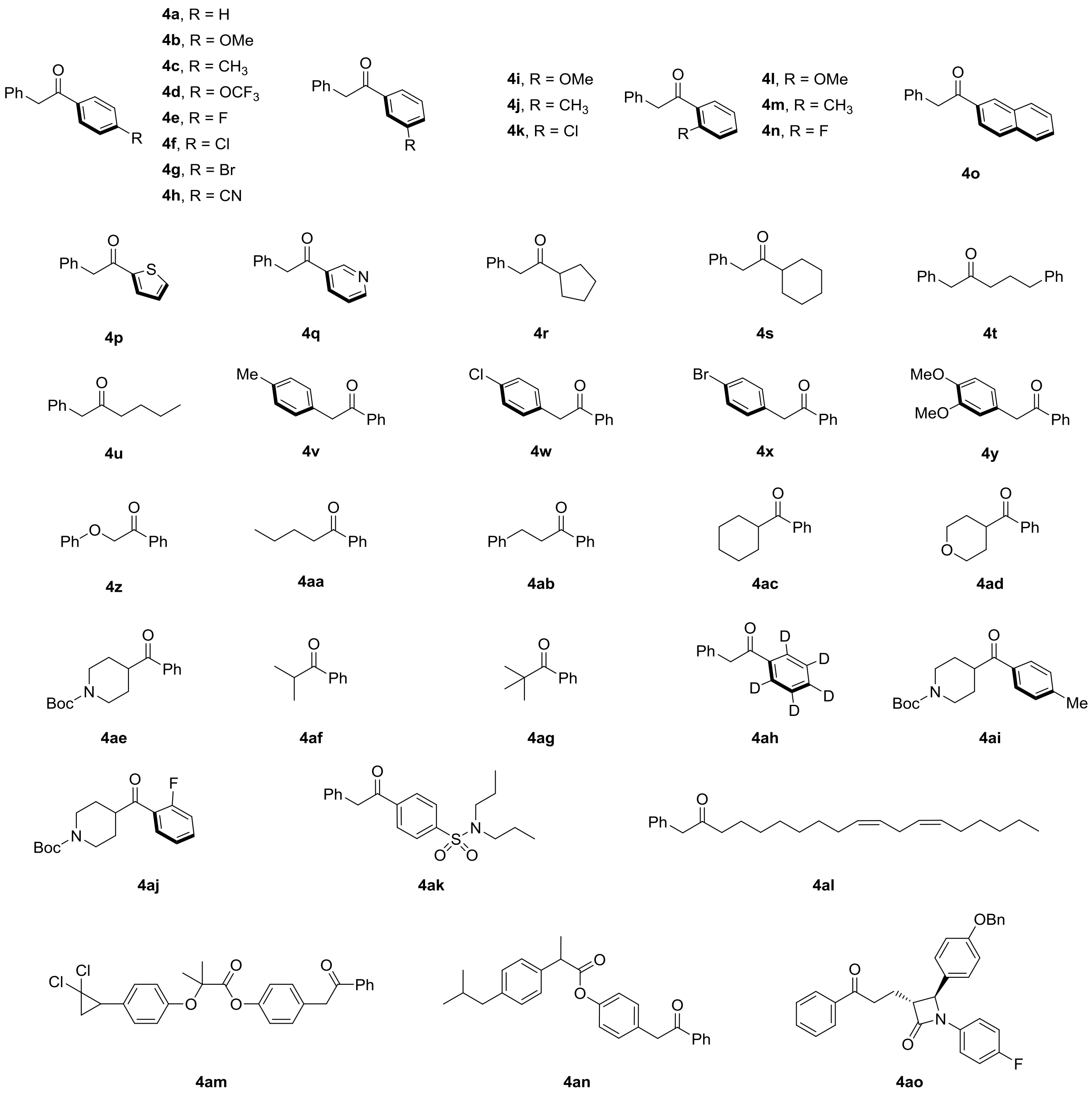

The reaction to activate alkene substrates is highly efficient. To confirm the structure of the resulting pro-aromatic intermdiate, the representative **2a** is prepared and isolated through the following procedure.

The alkene **1a** (0.1 mmol) and dimethyl 1,2,4,5-tetrazine-3,6-dicarboxylate **A** (1.1 equiv) were combined in DCM (0.2 M). The solution was stirred at room temperature, and the reaction was monitored by TLC. The color of the solution changed from red to yellow. After complete consumption of starting materials, the solvent was removed under vacuum. The resulting crude was purified by column chromatography to deliver **2a** as a gum (28.7 mg, 95% yield).

***dimethyl-4-benzyl-4-methyl-1,4-dihydropyridazine-3,6-dicarboxylate* 2a**

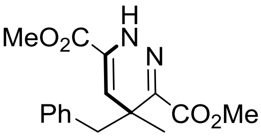

NMR and HRMS data for the substrate **2a**:

^{1}H NMR (600 MHz, $CDCl_3$) δ (ppm): 7.82 (s, 1H), 7.24-7.17 (m, 3H), 7.07 (d, *J* = 6.6 Hz, 2H), 5.52 (d, *J* = 2.4 Hz, 1H), 3.81 (s, 3H), 3.81 (s, 3H), 3.35 (d, *J* = 13.2 Hz, 1H), 2.68 (d, *J* = 13.2 Hz, 1H), 1.54 (s, 3H).

^{13}C NMR (151 MHz, $CDCl_3$) δ (ppm): 164.5, 161.4, 137.0, 135.5, 130.3, 128.0, 126.9, 126.4, 116.3, 52.5, 52.1, 46.6, 37.8, 28.2.

HRMS (ESI-TOF) m/z: $[M + Na]^+$ calculated for $C_{16}H_{18}N_2O_4Na^+$: 325.1159, found: 325.1151.

Characterization of representative ketone products:

***1,2-diphenylethan-1-one* 4a**

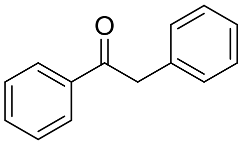

Prepared according to ***General Procedure A*** to deliver **4a** as a colourless oil. The reaction starting from **1a** afford 14.9 mg of **4a** in 76% yield; the reaction starting from **7** afford 13.1 mg of **4a** in 67% yield.

NMR and HRMS data for the substrate **4a**:

^{1}H NMR (600 MHz, $CDCl_3$) δ (ppm): 8.03 (d, *J* = 8.4 Hz, 2H), 7.56 (t, *J* = 7.2 Hz, 1H), 7.48-7.45 (m, 2H), 7.35-7.32 (m, 2H), 7.28-7.25 (m, 3H), 4.30 (s, 2H).

^{13}C NMR (151 MHz, $CDCl_3$) δ (ppm): 197.6, 136.6, 134.5, 133.1, 129.4, 128.64, 128.61, 128.58, 126.9, 45.5.

HRMS (ESI-TOF) m/z: $[M + Na]^+$ calculated for $C_{14}H_{12}ONa^+$: 219.0781, found: 219.0776.

***1-(4-methoxyphenyl)-2-phenylethan-1-one* 4b**

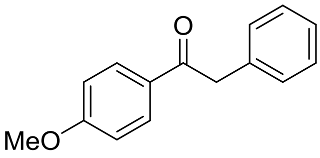

Prepared according to the ***General Procedure A*** to afford **4b** (15.2 mg, 67% yield) as a colourless oil.

NMR and HRMS data for the substrate **4b**:

^{1}H NMR (600 MHz, $CDCl_3$) δ (ppm): 8.00 (d, *J* = 9.0 Hz, 2H), 7.34-7.31 (m, 2H), 7.28-7.23 (m, 3H), 6.93 (d, *J* = 8.4 Hz, 2H), 4.24 (s, 2H), 3.86 (s, 3H).

^{13}C NMR (151 MHz, $CDCl_3$) δ (ppm): 196.2, 163.5, 134.9, 130.9, 129.6, 129.3, 128.6, 126.7, 113.7, 55.4, 45.2.

HRMS (ESI-TOF) m/z: $[M + Na]^+$ calculated for $C_{15}H_{14}O_2Na^+$: 249.0886, found: 249.0892.

***2-phenyl-1-(p-tolyl)ethan-1-one* 4c**

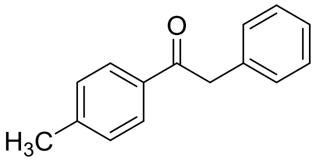

Prepared according to the ***General Procedure A*** to afford **4c** (13.5 mg, 64% yield) as a colourless oil.

NMR and HRMS data for the substrate **4c**:

^{1}H NMR (600 MHz, $CDCl_3$) δ (ppm): 7.93 (d, *J* = 8.4 Hz, 2H), 7.34 (t, *J* = 7.8 Hz, 2H), 7.29-7.25 (m, 5H), 4.28 (s, 2H), 2.42 (s, 3H).

^{13}C NMR (151 MHz, $CDCl_3$) δ (ppm): 197.3, 144.0, 134.7, 134.1, 129.4, 129.3, 128.7, 128.6, 126.8, 45.4, 21.6.

HRMS (ESI-TOF) m/z: $[M + Na]^+$ calculated for $C_{15}H_{14}ONa^+$: 233.0937, found: 233.0934.

***2-phenyl-1-(4-(trifluoromethoxy)phenyl)ethan-1-one* 4d**

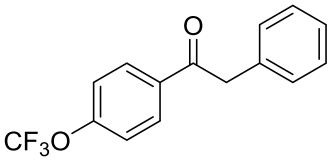

Prepared according to the ***General Procedure A*** to afford **4d** (17.4 mg, 62% yield) as a colourless oil.

NMR and HRMS data for the substrate **4d**:

^{1}H NMR (600 MHz, $CDCl_3$) δ (ppm): 8.06 (d, *J* = 9.0 Hz, 2H), 7.36-7.33 (m, 2H), 7.29-7.26 (m, 5H), 4.28 (s, 2H).

^{13}C NMR (151 MHz, $CDCl_3$) δ (ppm): 196.1, 152.6, 134.7, 134.0, 130.6, 129.4, 128.8, 127.1, 120.4, 120.2 (C-F, $^{1}J_{C-F}$ = 258.8 Hz), 45.6.

^{19}F NMR (564 MHz, $CDCl_3$) δ (ppm): -57.5.

HRMS (ESI-TOF) m/z: $[M + Na]^+$ calculated for $C_{15}H_{11}F_3O_2Na^+$: 303.0604, found: 303.0607.

***1-(4-fluorophenyl)-2-phenylethan-1-one* 4e**

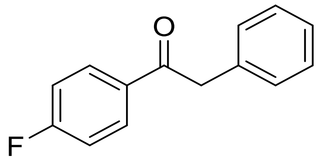

Prepared according to the ***General Procedure A*** to afford **4e** (14.1 mg, 66% yield) as a colourless oil.

NMR and HRMS data for the substrate **4e**:

^{1}H NMR (600 MHz, CDCl$_3$) δ (ppm): 8.05-8.02 (m, 2H), 7.34-7.32 (m, 2H), 7.27-7.25 (m, 3H), 7.12 (t, J = 9.0 Hz, 2H), 4.26 (s, 2H).

^{13}C NMR (151 MHz, CDCl$_3$) δ (ppm): 196.0, 165.7 (C-F, $^1J_{C-F}$ = 254.4 Hz), 134.3, 132.9, 131.2 (C-F, $^3J_{C-F}$ = 10.1 Hz), 129.3, 128.7, 127.0, 115.7 (C-F, $^2J_{C-F}$ = 21.7 Hz), 45.5.

^{19}F NMR (564 MHz, CDCl$_3$) δ (ppm): -104.9.

HRMS (ESI-TOF) m/z: [M + Na]$^+$ calculated for $C_{14}H_{11}FONa^+$: 237.0687, found: 237.0681.

***1-(4-chlorophenyl)-2-phenylethan-1-one* 4f**

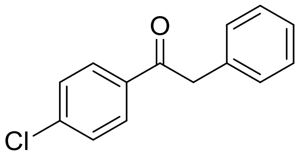

Prepared according to the ***General Procedure A*** to afford **4f** (14.1 mg, 61% yield) as a colourless oil.

NMR and HRMS data for the substrate **4f**:

^{1}H NMR (600 MHz, CDCl$_3$) δ (ppm): 7.95 (d, J = 8.4 Hz, 2H), 7.43 (d, J = 7.8 Hz, 2H), 7.35-7.32 (m, 2H), 7.28-7.25 (m, 3H), 4.26 (s, 2H).

^{13}C NMR (151 MHz, CDCl$_3$) δ (ppm): 196.4, 139.6, 134.8, 134.1, 130.0, 129.3, 128.9, 128.7, 127.0, 45.5.

HRMS (ESI-TOF) m/z: [M + Na]$^+$ calculated for $C_{14}H_{11}{}^{35}ClONa^+$: 253.0391, found: 253.0383; calculated for $C_{14}H_{11}{}^{37}ClONa^+$: 255.0362, found: 255.0358.

***1-(4-bromophenyl)-2-phenylethan-1-one* 4g**

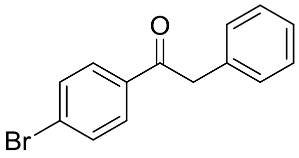

Prepared according to the ***General Procedure A*** to afford **4g** (16.0 mg, 58% yield) as a colourless oil.

NMR and HRMS data for the substrate **4g**:

^{1}H NMR (600 MHz, CDCl$_3$) δ (ppm): 7.87 (d, J = 8.4 Hz, 2H), 7.60 (d, J = 7.8 Hz, 2H), 7.35-7.33 (m, 2H), 7.28-7.25 (m, 3H), 4.26 (s, 2H).

^{13}C NMR (151 MHz, CDCl$_3$) δ (ppm): 196.6, 135.2, 134.1, 131.9, 130.1, 129.4, 128.8, 128.4, 127.0, 45.5.

HRMS (ESI-TOF) m/z: [M + Na]$^+$ calculated for $C_{14}H_{11}{}^{79}BrONa^+$: 296.9886, found: 296.9890; calculated for $C_{14}H_{11}{}^{81}BrONa^+$: 298.9866, found: 298.9871.

***4-(2-phenylacetyl)benzonitrile* 4h**

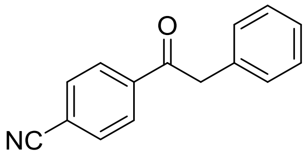

Prepared according to the ***General Procedure A*** to afford **4h** (10.2 mg, 46% yield) as a colourless oil.

NMR and HRMS data for the substrate **4h**:

^{1}H NMR (600 MHz, CDCl$_3$) δ (ppm): 8.08 (d, J = 8.4 Hz, 2H), 7.76 (d, J = 8.4 Hz, 2H), 7.36-7.33 (m, 2H), 7.29-7.24 (m, 3H), 4.30 (s, 2H).

^{13}C NMR (151 MHz, CDCl$_3$) δ (ppm): 196.2, 139.4, 133.4, 132.5, 129.3, 129.0, 128.9, 127.3, 117.9, 116.4, 45.8.

HRMS (ESI-TOF) m/z: [M + Na]$^+$ calculated for $C_{15}H_{11}NONa^+$: 244.0733, found: 244.0736.

***1-(3-methoxyphenyl)-2-phenylethan-1-one* 4i**

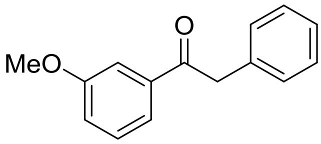

Prepared according to the ***General Procedure A*** to afford **4i** (12.7 mg, 56% yield) as a colourless oil.

NMR and HRMS data for the substrate **4i**:

^{1}H NMR (600 MHz, CDCl$_3$) δ (ppm): 7.60 (d, J = 7.8 Hz, 1H), 7.53 (s, 1H), 7.38-7.31 (m, 3H), 7.27-7.24 (m, 3H), 7.10 (d, J = 7.6 Hz, 1H), 4.27 (s, 2H), 3.84 (s, 3H).

^{13}C NMR (151 MHz, CDCl$_3$) δ (ppm): 197.4, 159.8, 137.9, 134.5, 129.6, 129.4, 128.7, 126.9, 121.3, 119.6, 112.8, 55.4, 45.6.

HRMS (ESI-TOF) m/z: [M + Na]$^+$ calculated for $C_{15}H_{14}O_2Na^+$: 249.0886, found: 249.0887.

***2-phenyl-1-(m-tolyl)ethan-1-one* 4j**

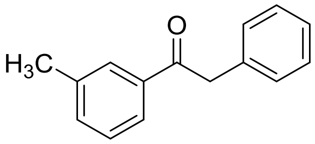

Prepared according to the ***General Procedure A*** to afford **4j** (12.0 mg, 57% yield) as a colourless oil.

NMR and HRMS data for the substrate **4j**:

^{1}H NMR (600 MHz, $CDCl_3$) δ (ppm): 7.84-7.81 (m, 2H), 7.38-7.32 (m, 4H), 7.28-7.25 (m, 3H), 4.28 (s, 2H), 2.41 (s, 3H).

^{13}C NMR (151 MHz, $CDCl_3$) δ (ppm): 197.8, 138.4, 136.6, 134.6, 133.9, 129.4, 129.1, 128.6, 128.5, 126.8, 125.8, 45.5, 21.3.

HRMS (ESI-TOF) m/z: $[M + H]^+$ calculated for $C_{15}H_{15}O^+$: 211.1118, found: 211.1126.

***1-(3-chlorophenyl)-2-phenylethan-1-one* 4k**

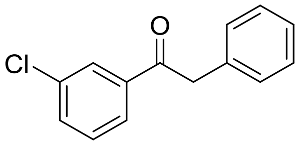

Prepared according to the ***General Procedure A*** to afford **4k** (12.2 mg, 53% yield) as a colourless oil.

NMR and HRMS data for the substrate **4k**:

^{1}H NMR (600 MHz, $CDCl_3$) δ (ppm): 7.97 (t, *J* = 1.8 Hz, 1H), 7.87 (d, *J* = 7.8 Hz, 1H), 7.53-7.51 (m, 1H), 7.39 (t, *J* = 7.2 Hz, 1H), 7.34-7.32 (m, 2H), 7.27-7.24 (m, 3H), 4.25 (s, 2H).

^{13}C NMR (151 MHz, $CDCl_3$) δ (ppm): 196.3, 138.1, 135.0, 133.9, 133.1, 129.9, 129.4, 128.8, 128.7, 127.1, 126.7, 45.6.

HRMS (ESI-TOF) m/z: $[M + H]^+$ calculated for $C_{14}H_{12}{}^{35}ClO^+$: 231.0572, found: 231.0568; calculated for $C_{14}H_{12}{}^{37}ClO^+$: 233.0542, found: 233.0537.

***1-(2-methoxyphenyl)-2-phenylethan-1-one* 4l**

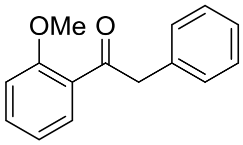

Prepared according to the ***General Procedure A*** to afford **4l** (16.1 mg, 71% yield) as a colourless oil.

NMR and HRMS data for the substrate **4l**:

^{1}H NMR (600 MHz, CDCl$_3$) δ (ppm): 7.68-7.66 (m, 1H), 7.47-7.44 (m, 1H), 7.32-7.29 (m, 2H), 7.24-7.22 (m, 3H), 6.99 (t, *J* = 7.8 Hz, 1H), 6.96 (d, *J* = 8.4 Hz, 1H), 4.31 (s, 2H), 3.92 (s, 3H).

^{13}C NMR (151 MHz, CDCl$_3$) δ (ppm): 200.1, 158.3, 135.2, 133.5, 130.6, 129.7, 128.3, 128.2, 126.5, 120.7, 111.4, 55.4, 50.1.

HRMS (ESI-TOF) m/z: [M + Na]$^+$ calculated for $C_{15}H_{14}O_2Na^+$: 249.0886, found: 249.0883.

***2-phenyl-1-(o-tolyl)ethan-1-one* 4m**

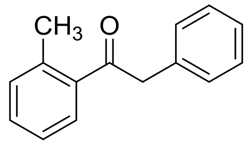

Prepared according to the ***General Procedure A*** to afford **4m** (13.0 mg, 62% yield) as a colourless oil.

NMR and HRMS data for the substrate **4m**:

^{1}H NMR (600 MHz, CDCl$_3$) δ (ppm): 7.72 (d, *J* = 7.8 Hz, 1H), 7.36 (t, *J* = 7.2 Hz, 1H), 7.33-7.31 (m, 2H), 7.27-7.23 (m, 5H), 4.21 (s, 2H), 2.44 (s, 3H).

^{13}C NMR (151 MHz, CDCl$_3$) δ (ppm): 201.4, 138.5, 137.6, 134.4, 131.9, 131.3, 129.5, 128.6, 126.8, 125.6, 48.4, 21.2.

HRMS (ESI-TOF) m/z: [M + H]$^+$ calculated for $C_{15}H_{15}O^+$: 211.1118, found: 211.1124.

***1-(2-fluorophenyl)-2-phenylethan-1-one* 4n**

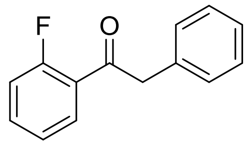

Prepared according to the ***General Procedure A*** to afford **4n** (12.9 mg, 60% yield) as a colourless oil.

NMR and HRMS data for the substrate **4n**:

^{1}H NMR (600 MHz, CDCl$_3$) δ (ppm): 7.86-7.83 (m, 1H), 7.53-7.49 (m, 1H), 7.33-7.30 (m, 2H), 7.26-7.24 (m, 3H), 7.21 (t, *J* = 7.8 Hz, 1H), 7.15-7.11 (m, 1H), 4.29 (d, *J* = 2.4 Hz, 2H).

^{13}C NMR (151 MHz, CDCl$_3$) δ (ppm): 196.2 (C-F, $^3J_{C-F}$ = 4.2 Hz), 161.7 (C-F, $^1J_{C-F}$ = 254.4 Hz), 134.6 (C-F, $^3J_{C-F}$ = 8.8 Hz), 134.0, 131.0 (C-F, $^4J_{C-F}$ = 2.9 Hz), 129.7, 128.5, 126.9, 125.5 (C-F, $^2J_{C-F}$ = 12.3 Hz), 124.5 (C-F, $^3J_{C-F}$ = 3.0 Hz), 116.6 (C-F, $^2J_{C-F}$ = 23.1 Hz), 49.9(C-F, $^4J_{C-F}$ = 7.2 Hz).

^{19}F NMR (564 MHz, CDCl$_3$) δ (ppm): -108.8.

HRMS (ESI-TOF) m/z: [M + Na]$^+$ calculated for $C_{14}H_{11}FONa^+$: 237.0687, found: 237.0689.

***1-(naphthalen-2-yl)-2-phenylethan-1-one* 4o**

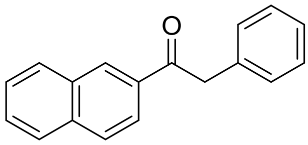

Prepared according to the ***General Procedure A*** to afford **4o** (18.7 mg, 76% yield) as a colourless oil.

NMR and HRMS data for the substrate **4o**:

^{1}H NMR (600 MHz, CDCl$_3$) δ (ppm): 8.56 (s, 1H), 8.08-8.06 (m, 1H), 7.97 (d, *J* = 8.4 Hz, 1H), 7.90-7.87 (m, 2H), 7.60 (t, *J* = 7.8 Hz, 1H), 7.56 (t, *J* = 7.8 Hz, 1H), 7.36-7.32 (m, 4H), 7.28-7.25 (m, 1H), 4.43 (s, 2H).

^{13}C NMR (151 MHz, CDCl$_3$) δ (ppm): 197.6, 135.5, 134.6, 133.9, 132.4, 130.4, 129.6, 129.5, 128.7, 128.52, 128.48, 127.7, 126.9, 126.8, 124.2, 45.5.

HRMS (ESI-TOF) m/z: [M + Na]$^+$ calculated for $C_{18}H_{14}ONa^+$: 269.0937, found: 269.0936.

***2-phenyl-1-(thiophen-2-yl)ethan-1-one* 4p**

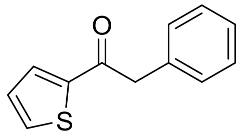

Prepared according to the ***General Procedure A*** to afford **4p** (10.9 mg, 54% yield) as a colourless oil.

NMR and HRMS data for the substrate **4p**:

^{1}H NMR (600 MHz, CDCl$_3$) δ (ppm): 7.77-7.77 (m, 1H), 7.64-7.63 (m, 1H), 7.34-7.30 (m, 4H), 7.27-7.25 (m, 1H), 7.13-7.11 (m, 1H), 4.20 (s, 2H).

^{13}C NMR (151 MHz, CDCl$_3$) δ (ppm): 190.4, 143.9, 134.3, 134.0, 132.6, 129.4, 128.7, 128.1, 127.0, 46.4.

HRMS (ESI-TOF) m/z: [M + Na]$^+$ calculated for $C_{12}H_{10}OSNa^+$: 225.0345, found: 225.0348.

***2-phenyl-1-(pyridin-3-yl)ethan-1-one* 4q**

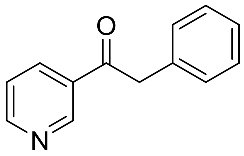

Prepared according to the ***General Procedure A*** to afford **4q** (11.8 mg, 60% yield) as a colourless oil.

NMR and HRMS data for the substrate **4q**:

^{1}H NMR (600 MHz, CDCl$_3$) δ (ppm): 9.21 (s, 1H), 8.74 (d, *J* = 4.2 Hz, 1H), 8.24 (d, *J* = 7.8 Hz, 1H), 7.39 (t, *J* = 6.6 Hz, 1H), 7.33-7.31 (m, 2H), 7.26-7.24 (m, 3H), 4.28 (s, 2H).

^{13}C NMR (151 MHz, CDCl$_3$) δ (ppm): 196.4, 153.5, 150.1, 135.9, 133.5, 131.7, 129.4, 128.8, 127.2, 123.7, 45.8.

HRMS (ESI-TOF) m/z: [M + Na]$^+$ calculated for $C_{13}H_{11}NONa^+$: 220.0733, found: 220.0731.

***1-cyclopentyl-2-phenylethan-1-one* 4r**

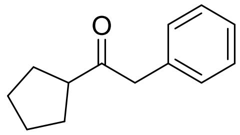

Prepared according to the ***General Procedure A*** to afford **4r** (9.8 mg, 52% yield) as a colourless oil.

NMR and HRMS data for the substrate **4r**:

^{1}H NMR (600 MHz, CDCl$_3$) δ (ppm): 7.34-7.32 (m, 2H), 7.27-7.25 (m, 1H), 7.21 (d, *J* = 7.2 Hz, 2H), 3.74 (s, 2H), 3.00-2.95 (m, 1H), 1.76-1.72 (m, 4H), 1.70-1.63 (m, 2H), 1.58-1.55 (m, 2H).

^{13}C NMR (151 MHz, CDCl$_3$) δ (ppm): 210.6, 134.5, 129.5, 128.6, 126.8, 50.6, 49.2, 29.1, 26.0.

HRMS (ESI-TOF) m/z: [M + Na]$^+$ calculated for $C_{13}H_{16}ONa^+$: 211.1094, found: 211.1097.

***1-cyclohexyl-2-phenylethan-1-one* 4s**

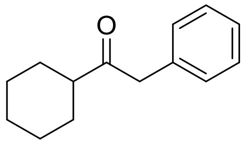

Prepared according to ***General Procedure A*** to deliver **4s** as a colourless oil. The reaction starting from **1a** afford 11.3 mg of **4s** in 56% yield; the reaction starting from **5** afford 11.5 mg of **4s** in 57% yield.

NMR and HRMS data for the substrate **4s**:

^{1}H NMR (600 MHz, CDCl$_3$) δ (ppm): 7.34-7.32 (m, 2H), 7.26 (t, *J* = 7.2 Hz, 1H), 7.20 (d, *J* = 7.2 Hz, 2H), 3.74 (s, 2H), 2.50-2.45 (m, 1H), 1.85-1.82 (m, 2H), 1.80-1.77 (m, 2H), 1.68-1.65 (m, 1H), 1.41-1.34 (m, 2H), 1.31-1.17 (m, 3H).

^{13}C NMR (151 MHz, CDCl$_3$) δ (ppm): 211.1, 134.3, 129.4, 129.2, 128.5, 126.7, 50.0, 47.7, 28.4, 25.7, 25.5.

HRMS (ESI-TOF) m/z: [M + Na]$^+$ calculated for $C_{14}H_{18}ONa^+$: 225.1250, found: 225.1251.

***1,5-diphenylpentan-2-one* 4t**

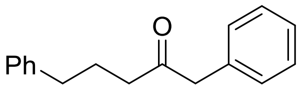

Prepared according to ***General Procedure A*** to deliver **4t** a colourless oil. The reaction starting from **1a** afford 13.1 mg of **4t** in 55% yield; the reaction starting from **25** afford 11.4 mg of **4t** in 48% yield.

NMR and HRMS data for the substrate **4t**:

^{1}H NMR (600 MHz, CDCl$_3$) δ (ppm): 7.33-7.31 (m, 2H), 7.27-7.24 (m, 3H), 7.18-7.17 (m, 3H), 7.09 (d, J = 7.8 Hz, 2H), 3.65 (s, 2H), 2.55 (t, J = 7.2 Hz, 2H), 2.46 (t, J = 7.2 Hz, 2H), 1.90-1.85 (m, 2H).

^{13}C NMR (151 MHz, CDCl$_3$) δ (ppm): 208.1, 141.5, 134.2, 129.4, 128.7, 128.4, 128.3, 127.0, 125.9, 50.2, 41.0, 34.9, 25.1.

HRMS (ESI-TOF) m/z: [M + Na]$^+$ calculated for $C_{17}H_{18}ONa^+$: 261.1250, found: 261.1253.

***1-phenylhexan-2-one* 4u**

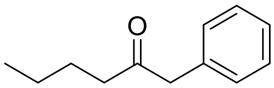

Prepared according to the ***General Procedure A*** to afford **4u** (8.5 mg, 48% yield) as a colourless oil.

NMR and HRMS data for the substrate **4u**:

^{1}H NMR (600 MHz, CDCl$_3$) δ (ppm): 7.34-7.31 (m, 2H), 7.26 (t, J = 7.8 Hz, 1H), 7.20 (d, J = 6.6 Hz, 2H), 3.68 (s, 2H), 2.44 (t, J = 7.8 Hz, 2H), 1.56-1.51 (m, 2H), 1.29-1.23 (m, 2H), 0.86 (t, J = 7.2 Hz, 3H).

^{13}C NMR (151 MHz, CDCl$_3$) δ (ppm): 208.6, 134.4, 129.4, 128.6, 126.9, 50.1, 41.7, 25.8, 22.2, 13.8.

HRMS (ESI-TOF) m/z: [M + Na]$^+$ calculated for $C_{12}H_{16}ONa^+$: 199.1094, found: 199.1103.

***1-phenyl-2-(p-tolyl)ethan-1-one* 4v**

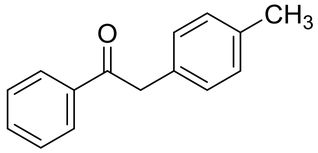

Prepared according to the ***General Procedure A*** to afford **4v** (13.9 mg, 66% yield) as a colourless oil.

NMR and HRMS data for the substrate **4v**:

^{1}H NMR (600 MHz, CDCl$_3$) δ (ppm): 8.03-8.01 (m, 2H), 7.56 (t, *J* = 7.2 Hz, 1H), 7.47-7.45 (m, 2H), 7.18-7.14 (m, 4H), 4.26 (s, 2H), 2.33 (s, 3H).

^{13}C NMR (151 MHz, CDCl$_3$) δ (ppm): 197.8, 136.6, 136.4, 133.0, 131.4, 129.4, 129.3, 128.6, 45.1, 21.0.

HRMS (ESI-TOF) m/z: $[M + Na]^+$ calculated for $C_{15}H_{14}ONa^+$: 233.0937, found: 233.0930.

***2-(4-chlorophenyl)-1-phenylethan-1-one* 4w**

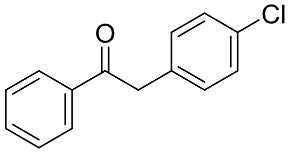

Prepared according to the ***General Procedure A*** to afford **4w** (14.8 mg, 64% yield) as a colourless oil.

NMR and HRMS data for the substrate **4w**:

^{1}H NMR (600 MHz, CDCl$_3$) δ (ppm): 8.00 (d, *J* = 6.6 Hz, 2H), 7.58 (t, *J* = 7.2 Hz, 1H), 7.49-7.46 (m, 2H), 7.30 (d, *J* = 7.8 Hz, 2H), 7.20 (d, *J* = 8.4 Hz, 2H), 4.26 (s, 2H).

^{13}C NMR (151 MHz, CDCl$_3$) δ (ppm): 197.1, 136.4, 133.3, 132.9, 132.8, 130.9, 128.8, 128.7, 128.5, 44.7.

HRMS (ESI-TOF) m/z: $[M + H]^+$ calculated for $C_{14}H_{12}{}^{35}ClO^+$: 231.0572, found: 231.0568; calculated for $C_{14}H_{12}{}^{37}ClO^+$: 233.0542, found: 233.0533.

***2-(4-bromophenyl)-1-phenylethan-1-one* 4x**

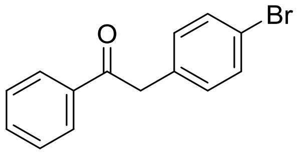

Prepared according to the ***General Procedure A*** to afford **4x** (16.8 mg, 61% yield) as a colourless oil.

NMR and HRMS data for the substrate **4x**:

^{1}H NMR (600 MHz, CDCl$_3$) δ (ppm): 8.01-7.99 (m, 2H), 7.58 (t, *J* = 7.2 Hz, 1H), 7.49-7.44 (m, 4H), 7.14 (d, *J* = 8.4 Hz, 2H), 4.25 (s, 2H).

^{13}C NMR (151 MHz, CDCl$_3$) δ (ppm): 197.0, 136.4, 133.42, 133.36, 131.7, 131.2, 128.7, 128.5, 120.9, 44.7.

HRMS (ESI-TOF) m/z: $[M + Na]^+$ calculated for $C_{14}H_{11}{}^{79}BrONa^+$: 296.9886, found: 296.9891; calculated for $C_{14}H_{11}{}^{79}BrONa^+$: 298.9866, found: 298.9868.

***2-(3,4-dimethoxyphenyl)-1-phenylethan-1-one* 4y**

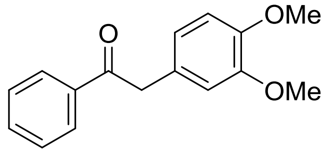

Prepared according to the ***General Procedure A*** to afford **4y** (14.4 mg, 56% yield) as a colourless oil.

NMR and HRMS data for the substrate **4y**:

^{1}H NMR (600 MHz, CDCl$_3$) δ (ppm): 8.02 (d, *J* = 6.6 Hz, 2H), 7.56 (t, *J* = 7.2 Hz, 1H), 7.47-7.45 (m, 2H), 6.83-6.79 (m, 3H), 4.23 (s, 2H), 3.86 (s, 6H).

^{13}C NMR (151 MHz, CDCl$_3$) δ (ppm): 197.9, 148.9, 147.9, 136.5, 133.1, 128.60, 128.55, 126.9, 121.6, 112.4, 111.2, 55.81, 55.79, 45.1.

HRMS (ESI-TOF) m/z: [M + Na]$^+$ calculated for $C_{16}H_{16}O_3Na^+$: 279.0992, found: 279.0985.

***2-phenoxy-1-phenylethan-1-one* 4z**

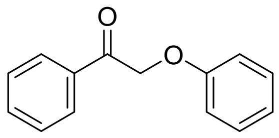

Prepared according to the ***General Procedure A*** to afford **4z** (12.3 mg, 58% yield) as a colourless oil.

NMR and HRMS data for the substrate **4z**:

^{1}H NMR (600 MHz, CDCl$_3$) δ (ppm): 8.01 (d, *J* = 6.6 Hz, 2H), 7.62 (t, *J* = 7.8 Hz, 1H), 7.52-7.49 (m, 2H), 7.31-7.28 (m, 2H), 6.99 (t, *J* = 7.2 Hz, 1H), 6.96 (d, *J* = 7.8 Hz, 2H), 5.28 (s, 2H).

^{13}C NMR (151 MHz, CDCl$_3$) δ (ppm): 194.5, 158.0, 134.6, 133.8, 129.5, 128.8, 128.1, 121.6, 114.8, 70.8.

HRMS (ESI-TOF) m/z: [M + H]$^+$ calculated for $C_{14}H_{13}O_2H^+$: 213.0911, found: 213.0919.

***1-phenylpentan-1-one* 4aa**

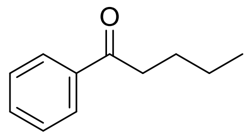

Prepared according to the ***General Procedure A*** to afford **4aa** (6.2 mg, 38% yield) as a colourless oil.

NMR and HRMS data for the substrate **4aa**:

^{1}H NMR (600 MHz, CDCl$_3$) δ (ppm): 7.97-7.95 (m, 2H), 7.55 (t, *J* = 7.2 Hz, 1H), 7.47-7.44 (m, 2H), 2.97 (t, *J* = 7.2 Hz, 2H), 1.75-1.70 (m, 2H), 1.44-1.38 (m, 2H), 0.95 (t, *J* = 7.2 Hz, 3H).

^{13}C NMR (151 MHz, CDCl$_3$) δ (ppm): 200.6, 137.1, 132.8, 128.5, 128.0, 38.3, 26.5, 22.5, 13.9.

HRMS (ESI-TOF) m/z: $[M + Na]^+$ calculated for $C_{11}H_{14}ONa^+$: 185.0937, found: 185.0942.

***1,3-diphenylpropan-1-one* 4ab**

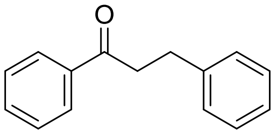

Prepared according to the ***General Procedure A*** to afford **4ab** (9.0 mg, 43% yield) as a colourless oil.

NMR and HRMS data for the substrate **4ab**:

^{1}H NMR (600 MHz, CDCl$_3$) δ (ppm): 7.98-7.96 (m, 2H), 7.58-7.55 (m, 1H), 7.47-7.45 (m, 2H), 7.32-7.29 (m, 2H), 7.27 (d, *J* = 6.0 Hz, 2H), 7.22 (t, *J* = 7.2 Hz, 1H), 3.31 (t, *J* = 7.8 Hz, 2H), 3.08 (t, *J* = 7.8 Hz, 2H).

^{13}C NMR (151 MHz, CDCl$_3$) δ (ppm): 199.2, 141.3, 136.8, 133.0, 128.6, 128.5, 128.4, 128.0, 126.1, 40.4, 30.1.

HRMS (ESI-TOF) m/z: $[M + Na]^+$ calculated for $C_{15}H_{14}ONa^+$: 233.0937, found: 233.0943.

***cyclohexyl(phenyl)methanone* 4ac**

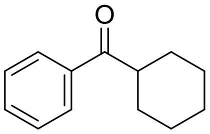

Prepared according to ***General Procedure A*** to deliver **4ac** as a colourless oil. The reaction starting from **1** afford 8.5 mg of **4ac** in 45% yield; the reaction starting from **41** afford 7.9 mg of **4ac** in 42% yield;

NMR and HRMS data for the substrate **4ac**:

^{1}H NMR (600 MHz, CDCl$_3$) δ (ppm): 7.94 (d, *J* = 7.2 Hz, 2H), 7.54 (t, *J* = 7.2 Hz, 1H), 7.47-7.44 (m, 2H), 3.29-3.24 (m, 1H), 1.90-1.83 (m, 4H), 1.75-1.73 (m, 1H), 1.53-1.46 (m, 2H), 1.43-1.37 (m, 2H), 1.31-1.23 (m, 1H).

^{13}C NMR (151 MHz, CDCl$_3$) δ (ppm): 203.9, 136.3, 132.7, 128.5, 128.2, 45.6, 29.4, 25.9, 25.8.

HRMS (ESI-TOF) m/z: $[M + H]^+$ calculated for $C_{13}H_{17}O^+$: 189.1274, found: 189.1280.

***phenyl(tetrahydro-2H-pyran-4-yl)methanone* 4ad**

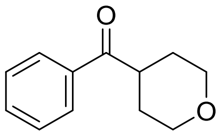

Prepared according to the ***General Procedure A*** to afford **4ad** (8.9 mg, 47% yield) as a colourless oil.

NMR and HRMS data for the substrate **4ad**:

^{1}H NMR (600 MHz, $CDCl_3$) δ (ppm): 7.94 (d, *J* = 6.6 Hz, 2H), 7.58-7.55 (m, 1H), 7.49-7.46 (m, 2H), 4.07-4.04 (m, 2H), 3.58-3.54 (m, 2H), 3.53-3.48 (m, 1H), 1.92-1.85 (m, 2H), 1.80-1.77 (m, 2H).

^{13}C NMR (151 MHz, $CDCl_3$) δ (ppm): 201.8, 135.8, 133.0, 128.7, 128.2, 67.3, 42.6, 29.1.

HRMS (ESI-TOF) m/z: $[M + Na]^+$ calculated for $C_{12}H_{14}O_2Na^+$: 213.0886, found: 213.0887.

***tert-butyl 4-benzoylpiperidine-1-carboxylate* 4ae**

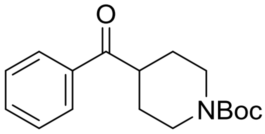

Prepared according to the ***General Procedure A*** to afford **4ae** (15.6 mg, 54% yield) as a colourless oil.

NMR and HRMS data for the substrate **4ae**:

^{1}H NMR (600 MHz, $CDCl_3$) δ (ppm): 7.93 (d, *J* = 7.2 Hz, 2H), 7.56 (t, *J* = 6.6 Hz, 1H), 7.48-7.46 (m, 2H), 4.29-4.01 (m, 2H), 3.42-3.38 (m, 1H), 2.89-2.89 (m, 2H), 1.83-1.76 (m, 2H), 1.70-1.69 (m, 2H), 1.46 (s, 9H).

^{13}C NMR (151 MHz, $CDCl_3$) δ (ppm): 202.1, 154.7, 135.8, 133.1, 128.7, 128.2, 79.6, 43.5, 42.8, 28.4.

HRMS (ESI-TOF) m/z: $[M + Na]^+$ calculated for $C_{17}H_{23}NO_3Na^+$: 312.1571, found: 312.1579.

***2-methyl-1-phenylpropan-1-one* 4af**

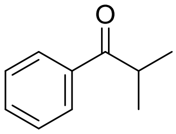

Prepared according to the ***General Procedure A*** to afford **4af** (6.1 mg, 41% yield) as a colourless oil.

NMR and HRMS data for the substrate **4af**:

^{1}H NMR (600 MHz, $CDCl_3$) δ (ppm): 7.96 (d, *J* = 6.6 Hz, 2H), 7.55 (t, *J* = 7.2 Hz, 1H), 7.48-7.45 (m, 2H), 3.58-3.54 (m, 1H), 1.22 (d, *J* = 6.6 Hz, 6H).
^{13}C NMR (151 MHz, $CDCl_3$) δ (ppm): 204.5, 136.2, 132.8, 128.6, 128.3, 35.3, 19.1.
HRMS (ESI-TOF) m/z: $[M + H]^+$ calculated for $C_{10}H_{13}O^+$: 149.0961, found: 149.0963.

***2,2-dimethyl-1-phenylpropan-1-one* 4ag**

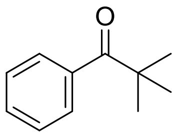

Prepared according to the ***General Procedure A*** to afford **4ag** (5.2 mg, 32% yield) as a colourless oil.
NMR and HRMS data for the substrate **4ag**:
^{1}H NMR (600 MHz, $CDCl_3$) δ (ppm): 7.69 (d, *J* = 7.2 Hz, 2H), 7.46 (t, *J* = 7.2 Hz, 1H), 7.41-7.38 (m, 2H), 1.35 (s, 9H).
^{13}C NMR (151 MHz, $CDCl_3$) δ (ppm): 209.3, 138.6, 130.8, 128.0, 127.8, 44.2, 28.0.
HRMS (ESI-TOF) m/z: $[M + H]^+$ calculated for $C_{11}H_{15}O^+$: 163.1118, found: 163.1125.

***2-phenyl-1-(phenyl-d5)ethan-1-one* 4ah**

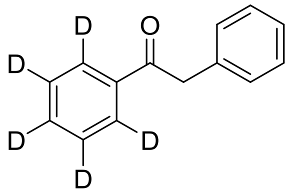

Prepared according to the ***General Procedure A*** to afford **4ah** (12.7 mg, 63% yield) as a colourless oil.
NMR and HRMS data for the substrate **4ah**:
^{1}H NMR (600 MHz, $CDCl_3$) δ (ppm): 7.35-7.32 (m, 2H), 7.29-7.25 (m, 3H), 4.30 (s, 2H).
^{13}C NMR (151 MHz, $CDCl_3$) δ (ppm): 197.6, 136.4, 134.5, 132.6 (t, *J* = 24.6 Hz), 129.4, 128.6, 128.2 (t, *J* = 24.5 Hz), 128.1 (t, *J* = 24.5 Hz), 126.9, 45.5.
HRMS (ESI-TOF) m/z: $[M + H]^+$ calculated for $C_{14}H_5D_5O^+$: 202.1275, found: 202.1274.

***tert-butyl 4-(4-methylbenzoyl)piperidine-1-carboxylate* 4ai**

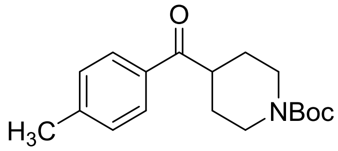

Prepared according to the ***General Procedure A*** to afford **4ai** (13.7 mg, 45% yield) as a colourless oil.

NMR and HRMS data for the substrate **4ai**:

^{1}H NMR (600 MHz, CDCl$_3$) δ (ppm): 7.84 (d, *J* = 8.4 Hz, 2H), 7.27 (d, *J* = 8.4 Hz, 2H), 4.30-4.03 (m, 2H), 3.40-3.35 (m, 1H), 2.89-2.78 (m, 2H), 2.41 (s, 3H), 1.90-1.82 (m, 2H), 1.70-1.68 (m, 2H), 1.46 (s, 9H).

^{13}C NMR (151 MHz, CDCl$_3$) δ (ppm): 201.7, 154.7, 143.9, 133.3, 129.4, 128.4, 79.6, 43.3, 42.9, 28.4, 21.6.

HRMS (ESI-TOF) m/z: [M + Na]$^+$ calculated for $C_{18}H_{25}NO_3Na^+$: 326.1727, found: 326.1737.

***tert-butyl 4-(2-fluorobenzoyl)piperidine-1-carboxylate* 4aj**

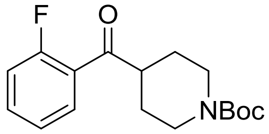

Prepared according to the ***General Procedure A*** to afford **4aj** (12.9 mg, 42% yield) as a colourless oil.

NMR and HRMS data for the substrate **4aj**:

^{1}H NMR (600 MHz, CDCl$_3$) δ (ppm): 7.78-7.75 (m, 1H), 7.53-7.50 (m, 1H), 7.23 (t, *J* = 7.2 Hz, 1H), 7.14-7.11 (m, 1H), 4.24-4.12 (m, 2H), 3.29-3.24 (m, 1H), 2.87-2.77 (m, 2H), 1.90-1.88 (m, 2H), 1.62-1.56 (m, 2H), 1.45 (s, 9H).

^{13}C NMR (151 MHz, CDCl$_3$) δ (ppm): 200.9 (C-F, $^3J_{C-F}$ = 3.0 Hz), 161.1 (C-F, $^1J_{C-F}$ = 252.9 Hz), 154.7, 134.3 (C-F, $^3J_{C-F}$ = 8.6 Hz), 130.9 (C-F, $^4J_{C-F}$ = 2.9 Hz), 125.3 (C-F, $^2J_{C-F}$ = 13.0 Hz), 124.7 (C-F, $^3J_{C-F}$ = 4.4 Hz), 116.5 (C-F, $^2J_{C-F}$ = 24.6 Hz), 79.5, 48.1 (C-F, $^4J_{C-F}$ = 5.9 Hz), 43.0, 28.4, 27.8.

^{19}F NMR (564 MHz, CDCl$_3$) δ (ppm): -111.4.

HRMS (ESI-TOF) m/z: [M + Na]$^+$ calculated for $C_{17}H_{22}FNO_3Na^+$: 330.1476, found: 330.1480.

***4-(2-phenylacetyl)-N,N-dipropylbenzenesulfonamide* 4ak**

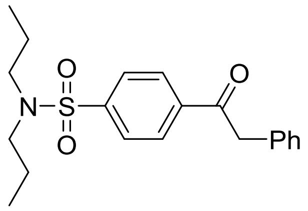

Prepared according to the ***General Procedure A*** to afford **4ak** (26.6 mg, 74% yield) as a colourless oil.

NMR and HRMS data for the substrate **4ak**:

^{1}H NMR (600 MHz, $CDCl_3$) δ (ppm): 8.09 (d, *J* = 8.4 Hz, 2H), 7.87 (d, *J* = 9.0 Hz, 2H), 7.34-7.32 (m, 2H), 7.28-7.24 (m, 3H), 4.30 (s, 2H), 3.08 (t, *J* = 7.2 Hz, 4H), 1.57-1.50 (m, 4H), 0.85 (t, *J* = 7.2 Hz, 6H).

^{13}C NMR (151 MHz, $CDCl_3$) δ (ppm): 196.5, 144.2, 139.2, 133.7, 129.4, 129.1, 128.8, 127.25, 127.15, 49.9, 45.8, 21.9, 11.1.

HRMS (ESI-TOF) m/z: $[M + H]^+$ calculated for $C_{20}H_{26}NO_3S^+$: 360.1628, found: 360.1631.

***(10Z,13Z)-1-phenylnonadeca-10,13-dien-2-one* 4al**

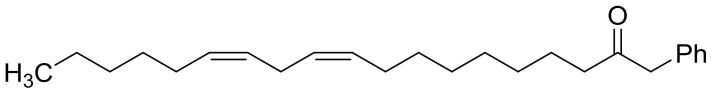

Prepared according to the ***General Procedure A*** to afford **4al** (16.3 mg, 46% yield) as a colourless oil.

NMR and HRMS data for the substrate **4al**:

^{1}H NMR (600 MHz, $CDCl_3$) δ (ppm): 7.34-7.32 (m, 2H), 7.27-7.25 (m, 1H), 7.21 (d, *J* = 7.2 Hz, 2H), 5.43-5.33 (m, 4H), 3.68 (s, 2H), 2.79 (t, *J* = 6.6 Hz, 2H), 2.45 (t, *J* = 7.2 Hz, 2H), 2.09-2.02 (m, 4H), 1.59-1.54 (m, 2H), 1.41-1.25 (m, 14H), 0.92 (t, *J* = 7.2 Hz, 3H).

^{13}C NMR (151 MHz, $CDCl_3$) δ (ppm): 208.2, 134.3, 130.0, 129.9, 129.2, 128.5, 127.9, 127.8, 126.8, 50.0, 41.8, 31.4, 29.5, 29.2, 29.1, 29.0, 28.9, 27.08, 27.06, 25.5, 23.6, 22.5, 14.0.

HRMS (ESI-TOF) m/z: $[M + H]^+$ calculated for $C_{25}H_{39}O^+$: 355.2996, found: 355.2997.

***4-(2-oxo-2-phenylethyl)phenyl-2-(4-(2,2-dichlorocyclopropyl)phenoxy)-2-methylpropanoate* 4am**

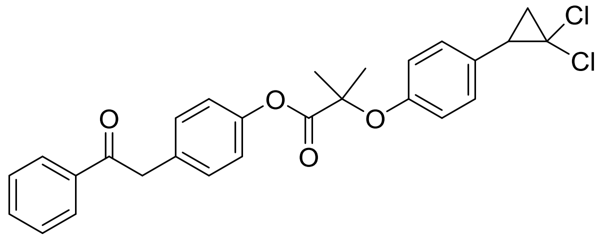

Prepared according to the ***General Procedure A*** to afford **4am** (34.3 mg, 71% yield) as a colourless oil.

NMR and HRMS data for the substrate **4am**:

^{1}H NMR (600 MHz, $CDCl_3$) δ (ppm): 7.98 (d, J = 8.4 Hz, 2H), 7.56 (t, J = 7.8 Hz, 1H), 7.47-7.44 (m, 2H), 7.24 (d, J = 9.0 Hz, 2H), 7.14 (d, J = 9.0 Hz, 2H), 6.94-6.91 (m, 4H), 4.26 (s, 2H), 2.86-2.83 (m, 1H), 1.96-1.92 (m, 1H), 1.79 (t, J = 7.8 Hz, 1H), 1.74 (s, 6H).

^{13}C NMR (151 MHz, $CDCl_3$) δ (ppm): 197.2, 172.8, 155.0, 149.4, 136.4, 133.3, 132.3, 130.5, 129.8, 128.7, 128.5, 128.4, 121.4, 118.6, 79.3, 60.8, 44.7, 34.8, 25.8, 25.5.

HRMS (ESI-TOF) m/z: $[M + H]^+$ calculated for $C_{27}H_{25}{}^{35}Cl_2O_4{}^+$: 483.1125, found: 483.1128; calculated for $C_{27}H_{25}{}^{35}Cl^{37}ClO_4{}^+$: 485.1095, found: 485.1103.

4. General Procedure for the Alkenylation

4.1 ***General Procedure B****: Access Terminal Alkene Product through Tebbe Reagent*

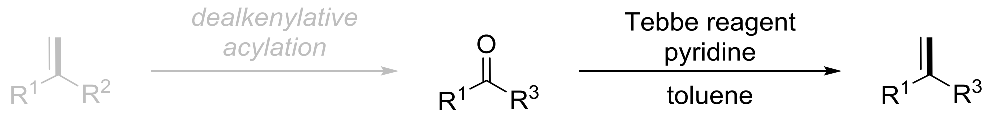

A 10 mL over-dried Schlenck tube was subjected to three cycles of pressurization and depressurization using dry Ar. Under the protection of Ar atmosphere, the Tebbe reagent (0.3 mmol), toluene (3 mL) and pyridine (0.3 mmol) were added successively at -40 ℃. The reaction mixture was stirred for 5 min. After that, the ketone was added at the same temperature. Then, the reaction mixture was raised to 40 ℃ slowly, and stirred for 5 h. After the reaction finished, the reaction mixture diluted with water and extracted with dichloromethane. The combined organic phases were washed with brine, dried over anhydrous Na_2SO_4, filtered and concentrated in vacuum. The crude residue was then purified by column chromatography on silica gel eluting with petroleum ether/ethyl acetate to provide the alkene product. All products were dried under vacuum and further analyzed by ^{1}H NMR, ^{13}C NMR, HRMS analysis, *etc*.

4.2 ***General Procedure C****: Access Terminal Alkene Product through Wittig Reagent*

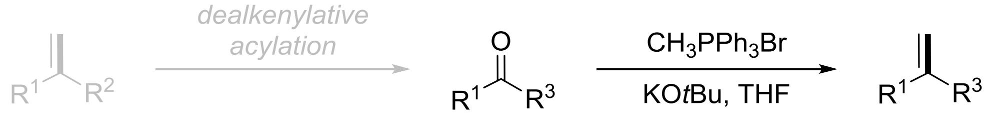

To a 10 mL over-dried Schlenck tube was added KO*t*Bu (0.2 mmol, 2 equiv.). The Schlenck tube was subjected to three cycles of pressurization and depressurization using dry Ar. After that, under the protection of Ar atmosphere, dry THF (1 mL) and CH_3PPh_3Br (0.2 mmol, 2 equiv.) were successively added. The suspension was stirred at room temperature for 1 h, and then the ketone (0.1 mmol, 1 equiv.) was added under Ar atmosphere. The resulting mixture was stirred at 50 ℃ for 5 h. After the reaction finished, the mixture was concentrated in vacuum, and purified by column chromatography on silica gel eluting from petroleum ether/ethyl acetate to provide the alkene product. All products were dried under vacuum and further analyzed by ^{1}H NMR, ^{13}C NMR, HRMS analysis, *etc*.

***prop-2-ene-1,2-diyldibenzene* 5**

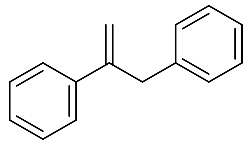

Prepared according to the ***General Procedure B*** to afford **5** (17.5 mg, 90% yield) as a colourless oil.

NMR and HRMS data for the substrate **5**:

^{1}H NMR (600 MHz, $CDCl_3$) δ (ppm): 7.46-7.45 (m, 2H), 7.32-7.24 (m, 7H), 7.21-7.19 (m, 1H), 5.52 (s, 1H), 5.04 (s, 1H), 3.86 (s, 2H).

^{13}C NMR (151 MHz, $CDCl_3$) δ (ppm): 146.9, 140.8, 139.5, 128.9, 128.3, 128.2, 127.4, 126.10, 126.07, 114.6, 41.6.

HRMS (ESI-TOF) m/z: $[M + H]^+$ calculated for $C_{15}H_{15}^+$: 195.1169, found: 195.1160.

***1-methoxy-4-(3-phenylprop-1-en-2-yl)benzene* 6**

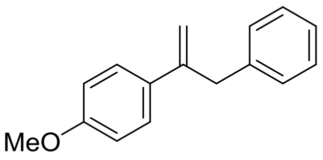

Prepared according to the ***General Procedure C*** to afford **6** (20.9 mg, 93% yield) as a colourless oil.

NMR and HRMS data for the substrate **6**:

^{1}H NMR (600 MHz, $CDCl_3$) δ (ppm): 7.42 (d, J = 9.0 Hz, 2H), 7.31-7.26 (m, 4H), 7.21 (t, J = 7.2 Hz, 1H), 6.85 (d, J = 7.8 Hz, 2H), 5.47 (s, 1H), 4.99 (s, 1H), 3.85 (s, 2H), 3.81 (s, 3H).

^{13}C NMR (151 MHz, $CDCl_3$) δ (ppm): 159.0, 146.0, 139.6, 133.1, 128.8, 128.3, 127.2, 126.0, 113.5, 113.0, 55.2, 41.7.

HRMS (ESI-TOF) m/z: $[M + H]^+$ calculated for $C_{16}H_{17}O^+$: 225.1274, found: 225.1276.

***1-methyl-4-(3-phenylprop-1-en-2-yl)benzene* 7**

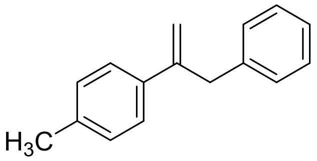

Prepared according to the ***General Procedure C*** to afford **7** (19.0 mg, 91% yield) as a colourless oil.

NMR and HRMS data for the substrate **7**:

^{1}H NMR (600 MHz, $CDCl_3$) δ (ppm): 7.39 (d, J = 8.4 Hz, 2H), 7.35-7.27 (m, 4H), 7.25-7.21 (m, 1H), 7.14 (d, J = 8.4 Hz, 2H), 5.53 (s, 1H), 5.03 (s, 1H), 3.87 (s, 2H), 2.36 (s, 3H).

^{13}C NMR (151 MHz, $CDCl_3$) δ (ppm): 146.6, 139.6, 137.8, 137.1, 128.92, 128.88, 128.3, 126.00, 125.96, 113.8, 41.6, 21.0.

HRMS (ESI-TOF) m/z: $[M + H]^+$ calculated for $C_{16}H_{17}^+$: 209.1325, found: 209.1327.

***1-(3-phenylprop-1-en-2-yl)-4-(trifluoromethoxy)benzene* 8**

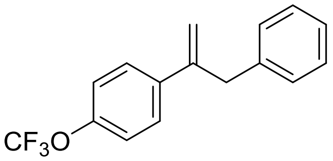

Prepared according to the ***General Procedure C*** to afford **8** (25.0 mg, 90% yield) as a colourless oil.

NMR and HRMS data for the substrate **8**:

^{1}H NMR (600 MHz, CDCl$_3$) δ (ppm): 7.45 (d, J = 9.0 Hz, 2H), 7.31-7.29 (m, 2H), 7.25-7.21 (m, 3H), 7.14 (d, J = 8.4 Hz, 2H), 5.51 (s, 1H), 5.10 (s, 1H), 3.84 (s, 2H).

^{13}C NMR (151 MHz, CDCl$_3$) δ (ppm): 148.5, 145.6, 139.4, 139.0, 128.8, 128.4, 127.5, 126.3, 120.6, 120.5 (C-F, $^1J_{C\text{-}F}$ = 257.3 Hz), 115.4, 41.7.

^{19}F NMR (564 MHz, CDCl$_3$) δ (ppm): -57.7.

HRMS (ESI-TOF) m/z: [M + Na]$^+$ calculated for $C_{16}H_{13}F_3ONa^+$: 301.0811, found: 301.0804.

***1-fluoro-4-(3-phenylprop-1-en-2-yl)benzene* 9**

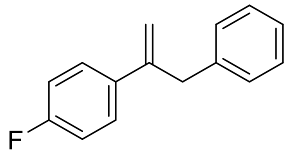

Prepared according to the ***General Procedure C*** to afford **9** (19.5 mg, 92% yield) as a colourless oil.

NMR and HRMS data for the substrate **9**:

^{1}H NMR (600 MHz, CDCl$_3$) δ (ppm): 7.42-7.40 (m, 2H), 7.31-7.28 (m, 2H), 7.25-7.20 (m, 3H), 6.99 (t, J = 8.4 Hz, 2H), 5.46 (s, 1H), 5.05 (s, 1H), 3.84 (s, 2H).

^{13}C NMR (151 MHz, CDCl$_3$) δ (ppm): 162.2 (C-F, $^1J_{C\text{-}F}$ = 247.2 Hz), 145.9, 139.2, 136.8 (C-F, $^4J_{C\text{-}F}$ = 2.9 Hz), 128.8, 128.4, 127.7 (C-F, $^3J_{C\text{-}F}$ = 7.2 Hz), 126.2, 115.0 (C-F, $^2J_{C\text{-}F}$ = 21.7 Hz), 114.5, 41.8.

^{19}F NMR (564 MHz, CDCl$_3$) δ (ppm): -115.0.

HRMS (ESI-TOF) m/z: [M + H]$^+$ calculated for $C_{15}H_{14}F^+$: 213.1075, found: 213.1073.

***1-chloro-4-(3-phenylprop-1-en-2-yl)benzene* 10**

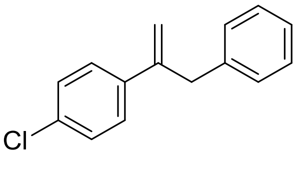

Prepared according to the ***General Procedure C*** to afford **10** (19.7 mg, 86% yield) as a

colourless oil.

NMR and HRMS data for the substrate **10**:

^{1}H NMR (600 MHz, $CDCl_3$) δ (ppm): 7.37 (d, *J* = 9.0 Hz, 2H), 7.30-7.25 (m, 4H), 7.23-7.20 (m, 3H), 5.50 (s, 1H), 5.09 (s, 1H), 3.83 (s, 2H).

^{13}C NMR (151 MHz, $CDCl_3$) δ (ppm): 145.7, 139.09, 139.05, 133.2, 128.8, 128.4, 128.3, 127.4, 126.2, 115.1, 41.6.

HRMS (ESI-TOF) m/z: $[M + H]^+$ calculated for $C_{15}H_{14}{}^{35}Cl^+$: 229.0779, found: 229.0785; calculated for $C_{15}H_{14}{}^{37}Cl^+$: 231.0750, found: 231.0748.

***1-bromo-4-(3-phenylprop-1-en-2-yl)benzene* 11**

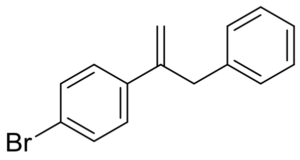

Prepared according to the ***General Procedure C*** to afford **11** (24.0 mg, 88% yield) as a colourless oil.

NMR and HRMS data for the substrate **11**:

^{1}H NMR (600 MHz, $CDCl_3$) δ (ppm): 7.42 (d, *J* = 8.4 Hz, 2H), 7.31-7.28 (m, 4H), 7.23-7.20 (m, 3H), 5.51 (s, 1H), 5.09 (s, 1H), 3.83 (s, 2H).

^{13}C NMR (151 MHz, $CDCl_3$) δ (ppm): 145.8, 139.6, 139.0, 131.3, 128.8, 128.4, 127.8, 126.2, 121.4, 115.2, 41.5.

HRMS (ESI-TOF) m/z: $[M + H]^+$ calculated for $C_{15}H_{14}{}^{79}Br^+$: 273.0274, found: 273.0275; calculated for $C_{15}H_{14}{}^{81}Br^+$: 275.0253, found: 275.0255.

***4-(3-phenylprop-1-en-2-yl)benzonitrile* 12**

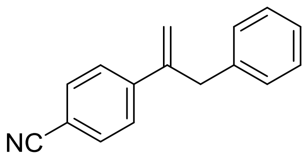

Prepared according to the ***General Procedure C*** to afford **12** (20.0 mg, 91% yield) as a colourless oil.

NMR and HRMS data for the substrate **12**:

^{1}H NMR (600 MHz, $CDCl_3$) δ (ppm): 7.56 (d, *J* = 7.8 Hz, 2H), 7.51 (d, *J* = 8.4 Hz, 2H), 7.30-7.27 (m, 2H), 7.22-7.20 (m, 3H), 5.60 (s, 1H), 5.23 (s, 1H), 3.85 (s, 2H).

^{13}C NMR (151 MHz, $CDCl_3$) δ (ppm): 145.4, 145.1, 138.4, 132.0, 128.7, 128.5, 126.7, 126.4, 118.8, 117.5, 110.9, 41.2.

HRMS (ESI-TOF) m/z: $[M + H]^+$ calculated for $C_{16}H_{14}N^+$: 220.1121, found: 220.1112.

***1-methoxy-3-(3-phenylprop-1-en-2-yl)benzene* 13**

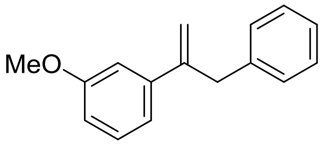

Prepared according to the ***General Procedure C*** to afford **13** (20.6 mg, 92% yield) as a colourless oil.

NMR and HRMS data for the substrate **13**:

^{1}H NMR (600 MHz, $CDCl_3$) δ (ppm): 7.32-7.30 (m, 2H), 7.28-7.21 (m, 4H), 7.08 (d, *J* = 7.2 Hz, 1H), 7.01-7.01 (m, 1H), 6.84-6.82 (m, 1H), 5.54 (s, 1H), 5.07 (s, 1H), 3.86 (s, 2H), 3.80 (s, 3H).

^{13}C NMR (151 MHz, $CDCl_3$) δ (ppm): 159.4, 146.8, 142.3, 139.4, 129.2, 128.9, 128.3, 126.1, 118.7, 114.8, 112.7, 112.1, 55.1, 41.7.

HRMS (ESI-TOF) m/z: $[M + H]^+$ calculated for $C_{16}H_{17}O^+$: 225.1274, found: 225.1277.

***1-methyl-3-(3-phenylprop-1-en-2-yl)benzene* 14**

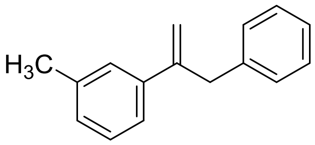

Prepared according to the ***General Procedure C*** to afford **14** (19.4 mg, 93% yield) as a colourless oil.

NMR and HRMS data for the substrate **14**:

^{1}H NMR (600 MHz, $CDCl_3$) δ (ppm): 7.32-7.29 (m, 3H), 7.27-7.26 (m, 3H), 7.23-7.20 (m, 2H), 7.09 (d, *J* = 7.2 Hz, 1H), 5.51 (s, 1H), 5.01 (s, 1H), 3.86 (s, 2H), 2.36 (s, 3H).

^{13}C NMR (151 MHz, $CDCl_3$) δ (ppm): 147.1, 140.8, 139.6, 137.7, 128.9, 128.3, 128.2, 128.1, 126.8, 126.0, 123.2, 114.4, 41.6, 21.5.

HRMS (ESI-TOF) m/z: $[M + H]^+$ calculated for $C_{16}H_{17}^+$: 209.1325, found: 209.1319.

***1-chloro-3-(3-phenylprop-1-en-2-yl)benzene* 15**

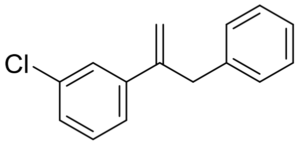

Prepared according to the ***General Procedure C*** to afford **15** (19.9 mg, 87% yield) as a colourless oil.

NMR and HRMS data for the substrate **15**:

^{1}H NMR (600 MHz, $CDCl_3$) δ (ppm): 7.45-7.45 (m, 1H), 7.33-7.29 (m, 3H), 7.24-7.21 (m, 5H), 5.51 (s, 1H), 5.09 (s, 1H), 3.83 (s, 2H).

^{13}C NMR (151 MHz, $CDCl_3$) δ (ppm): 145.8, 142.7, 138.9, 134.2, 129.5, 128.9, 128.4, 127.4, 126.33, 126.25, 124.3, 115.7, 41.4.

HRMS (ESI-TOF) m/z: $[M + H]^+$ calculated for $C_{15}H_{14}{}^{35}Cl^+$: 229.0779, found: 229.0784; calculated for $C_{15}H_{14}{}^{375}Cl^+$: 231.0750, found: 231.0749.

***1-methoxy-2-(3-phenylprop-1-en-2-yl)benzene* 16**

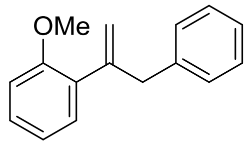

Prepared according to the ***General Procedure B*** to afford **16** (19.5 mg, 87% yield) as a colourless oil.

NMR and HRMS data for the substrate **16**:

^{1}H NMR (600 MHz, $CDCl_3$) δ (ppm): 7.26-7.22 (m, 3H), 7.19-7.15 (m, 3H), 7.07 (m, 1H), 6.88-6.85 (m, 2H), 5.14 (s, 1H), 5.09 (s, 1H), 3.86 (s, 3H), 3.82 (s, 2H).

^{13}C NMR (151 MHz, $CDCl_3$) δ (ppm): 156.5, 148.3, 139.9, 131.7, 130.2, 129.2, 128.4, 128.1, 125.8, 120.4, 115.8, 110.6, 55.4, 42.6.

HRMS (ESI-TOF) m/z: $[M + Na]^+$ calculated for $C_{16}H_{16}ONa^+$: 247.1094, found: 247.1096.

***1-methyl-2-(3-phenylprop-1-en-2-yl)benzene* 17**

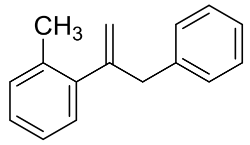

Prepared according to the ***General Procedure B*** to afford **17** (18.3 mg, 88% yield) as a colourless oil.

NMR and HRMS data for the substrate **17**:

^{1}H NMR (600 MHz, $CDCl_3$) δ (ppm): 7.30-7.28 (m, 2H), 7.24-7.21 (m, 1H), 7.18-7.16 (m, 4H), 7.14-7.12 (m, 1H), 7.04 (d, J = 6.6 Hz, 1H), 5.11 (s, 1H), 4.97 (s, 1H), 3.64 (s, 2H), 2.28 (s, 3H).

^{13}C NMR (151 MHz, $CDCl_3$) δ (ppm): 149.3, 142.6, 138.9, 134.8, 130.0, 129.3, 128.4, 128.2, 126.8, 126.1, 125.3, 115.3, 44.5, 19.7.

HRMS (ESI-TOF) m/z: $[M + Na]^+$ calculated for $C_{16}H_{16}Na^+$: 231.1145, found: 231.1145.

***1-fluoro-2-(3-phenylprop-1-en-2-yl)benzene* 18**

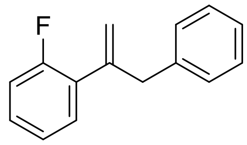

Prepared according to the ***General Procedure C*** to afford **18** (19.3 mg, 91% yield) as a colourless oil.

NMR and HRMS data for the substrate **18**:

^{1}H NMR (600 MHz, $CDCl_3$) δ (ppm): 7.26-7.24 (m, 2H), 7.22-7.16 (m, 5H), 7.04-7.00 (m, 2H), 5.30 (s, 1H), 5.19 (s, 1H), 3.81 (s, 2H).

^{13}C NMR (151 MHz, $CDCl_3$) δ (ppm): 159.8 (C-F, $^{1}J_{C\text{-}F}$ = 247.3 Hz), 144.1, 139.1, 130.1 (C-F, $^{3}J_{C\text{-}F}$ = 4.4 Hz), 129.5 (C-F, $^{2}J_{C\text{-}F}$ = 14.5 Hz), 129.0, 128.8 (C-F, $^{3}J_{C\text{-}F}$ = 8.6 Hz), 128.2, 126.1, 123.8 (C-F, $^{4}J_{C\text{-}F}$ = 2.9 Hz), 117.7 (C-F, $^{4}J_{C\text{-}F}$ = 2.9 Hz), 115.7 (C-F, $^{2}J_{C\text{-}F}$ = 23.1 Hz), 42.7 (C-F, $^{4}J_{C\text{-}F}$ = 2.9 Hz).

^{19}F NMR (564 MHz, $CDCl_3$) δ (ppm): -114.7.

HRMS (ESI-TOF) m/z: $[M + H]^+$ calculated for $C_{15}H_{14}F^+$: 213.1074, found: 213.1075.

***2-(3-phenylprop-1-en-2-yl)naphthalene* 19**

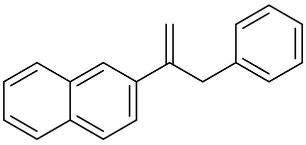

Prepared according to the ***General Procedure B*** to afford **19** (22.7 mg, 93% yield) as a colourless oil.

NMR and HRMS data for the substrate **19**:

^{1}H NMR (600 MHz, $CDCl_3$) δ (ppm): 7.91 (s, 1H), 7.84-7.80 (m, 3H), 7.68-7.67 (m, 1H), 7.50-7.46 (m, 2H), 7.34-7.30 (m, 4H), 7.24-7.21 (m, 1H), 5.70 (s, 1H), 5.19 (s, 1H), 4.01 (s, 2H).

^{13}C NMR (151 MHz, $CDCl_3$) δ (ppm): 146.7, 139.5, 138.0, 133.3, 132.8, 128.9, 128.3, 128.2, 127.8, 127.4, 126.1, 126.0, 125.8, 124.9, 124.5, 115.1, 41.6.

HRMS (ESI-TOF) m/z: $[M + Na]^+$ calculated for $C_{19}H_{16}Na^+$: 267.1144, found: 267.1145.

***2-(3-phenylprop-1-en-2-yl)thiophene* 20**

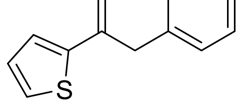

Prepared according to the ***General Procedure C*** to afford **20** (18.0 mg, 90% yield) as a colourless oil.

NMR and HRMS data for the substrate **20**:

^{1}H NMR (600 MHz, CDCl$_3$) δ (ppm): 7.34-7.30 (m, 4H), 7.26-7.23 (m, 1H), 7.16 (d, *J* = 4.8 Hz, 1H), 7.04 (d, *J* = 3.6 Hz, 1H), 6.94 (t, *J* = 4.2 Hz, 1H), 5.59 (s, 1H), 4.93 (s, 1H), 3.84 (s, 2H).

^{13}C NMR (151 MHz, CDCl$_3$) δ (ppm): 144.9, 140.4, 138.9, 128.9, 128.4, 127.3, 126.3, 124.2, 124.0, 113.2, 41.7.

HRMS (ESI-TOF) m/z: [M + H]$^+$ calculated for $C_{13}H_{13}S^+$: 201.0733, found: 201.0735.

***3-(3-phenylprop-1-en-2-yl)pyridine* 21**

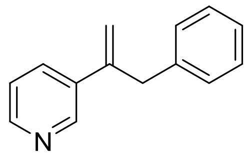

Prepared according to the ***General Procedure B*** to afford **21** (17.0 mg, 87% yield) as a colourless oil.

NMR and HRMS data for the substrate **21**:

^{1}H NMR (600 MHz, CDCl$_3$) δ (ppm): 8.69 (d, *J* = 1.2 Hz, 1H), 8.46-8.45 (m, 1H), 7.68-7.65 (m, 1H), 7.28-7.26 (m, 2H), 7.22-7.18 (m, 4H), 5.53 (s, 1H), 5.15 (s, 1H), 3.83 (s, 2H).

^{13}C NMR (151 MHz, CDCl$_3$) δ (ppm): 148.5, 147.7, 144.1, 138.5, 136.1, 133.3, 128.8, 128.5, 126.4, 123.0, 116.2, 41.4.

HRMS (ESI-TOF) m/z: [M + H]$^+$ calculated for $C_{14}H_{14}N^+$: 196.1121, found: 196.1115.

***(2-cyclopentylallyl)benzene* 22**

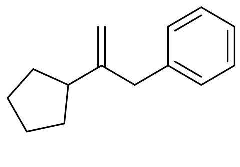

Prepared according to the ***General Procedure C*** to afford **22** (17.3 mg, 93% yield) as a colourless oil.

NMR and HRMS data for the substrate **22**:

^{1}H NMR (600 MHz, CDCl$_3$) δ (ppm): 7.32-7.29 (m, 2H), 7.23-7.22 (m, 3H), 4.90 (s, 1H), 4.68 (s, 1H), 3.40 (s, 2H), 2.38-2.32 (m, 1H), 1.81-1.81 (m, 2H), 1.71-1.66 (m, 2H), 1.59-1.52 (m, 2H), 1.45-1.40 (m, 2H).

^{13}**C NMR (151 MHz, $CDCl_3$) δ (ppm):** 152.6, 140.1, 129.1, 128.2, 125.9, 109.0, 45.3, 42.7, 31.5, 24.9.
HRMS (ESI-TOF) m/z: $[M + H]^+$ calculated for $C_{14}H_{19}^+$: 187.1482, found: 187.1480.

***(2-cyclohexylallyl)benzene* 23**

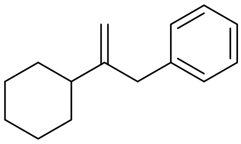

Prepared according to the ***General Procedure C*** to afford **23** (18.4 mg, 92% yield) as a colourless oil.
NMR and HRMS data for the substrate **23**:
^{1}**H NMR (600 MHz, $CDCl_3$) δ (ppm):** 7.31-7.29 (m, 2H), 7.22-7.19 (m, 3H), 4.86 (s, 1H), 4.65 (s, 1H), 3.38 (s, 2H), 1.86-1.75 (m, 5H), 1.68-1.66 (m, 1H), 1.28-1.16 (m, 5H).
^{13}**C NMR (151 MHz, $CDCl_3$) δ (ppm):** 154.5, 140.2, 129.1, 128.2, 125.9, 109.5, 43.4 41.8, 32.5, 26.7, 26.4.
HRMS (ESI-TOF) m/z: $[M + H]^+$ calculated for $C_{15}H_{21}^+$: 201.1638, found: 201.1639.

***(2-methylenepentane-1,5-diyl)dibenzene* 24**

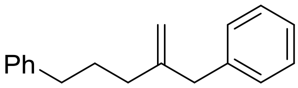

Prepared according to the ***General Procedure C*** to afford **24** (21.7 mg, 92% yield) as a colourless oil.
NMR and HRMS data for the substrate **24**:
^{1}**H NMR (600 MHz, $CDCl_3$) δ (ppm):** 7.33-7.29 (m, 4H), 7.25-7.18 (m, 6H), 4.88 (s, 1H), 4.80 (s, 1H), 3.38 (s, 2H), 2.61 (t, J = 7.8 Hz, 2H), 2.06 (t, J = 7.8 Hz, 2H), 1.83-1.78 (m, 2H).
^{13}**C NMR (151 MHz, $CDCl_3$) δ (ppm):** 148.7, 142.4, 139.7, 129.0, 128.4, 128.2, 126.0, 125.7, 111.3, 43.0, 35.5, 35.0, 29.4.
HRMS (ESI-TOF) m/z: $[M + Na]^+$ calculated for $C_{18}H_{20}Na^+$: 259.1458, found: 259.1449.

***(2-methylenehexyl)benzene* 25**

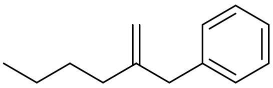

Prepared according to the ***General Procedure C*** to afford **25** (16.4 mg, 94% yield) as a colourless oil.

NMR and HRMS data for the substrate **25**:

^{1}H NMR (600 MHz, CDCl$_3$) δ (ppm): 7.31 (t, J = 7.2 Hz, 2H), 7.23-7.21 (m, 3H), 4.84 (s, 1H), 4.75 (s, 1H), 3.36 (s, 2H), 1.99 (t, J = 7.8 Hz, 2H), 1.47-1.42 (m, 2H), 1.35-1.28 (m, 2H), 0.91 (t, J = 7.2 Hz, 3H).

^{13}C NMR (151 MHz, CDCl$_3$) δ (ppm): 149.2, 139.9, 129.0, 128.2, 126.0, 110.9, 43.0, 35.1, 29.8, 22.4, 14.0.

HRMS (ESI-TOF) m/z: [M + H]$^+$ calculated for $C_{13}H_{19}^+$: 175.1482, found: 175.1490.

***1-methyl-4-(2-phenylallyl)benzene* 26**

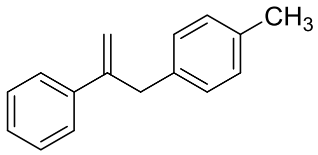

Prepared according to the ***General Procedure C*** to afford **26** (19.4 mg, 93% yield) as a colourless oil.

NMR and HRMS data for the substrate **26**:

^{1}H NMR (600 MHz, CDCl$_3$) δ (ppm): 7.47 (d, J = 7.2 Hz, 2H), 7.33-7.30 (m, 2H), 7.26 (t, J = 6.6 Hz, 1H), 7.15 (d, J = 7.8 Hz, 2H), 7.11 (d, J = 7.8 Hz, 2H), 5.52 (s, 1H), 5.05 (s, 1H), 3.83 (s, 2H), 2.33 (s, 3H).

^{13}C NMR (151 MHz, CDCl$_3$) δ (ppm): 147.1, 140.8, 136.4, 135.5, 129.0, 128.8, 128.2, 127.4, 126.1, 114.4, 41.2, 21.0.

HRMS (ESI-TOF) m/z: [M + Na]$^+$ calculated for $C_{16}H_{16}Na^+$: 231.1145, found: 231.1147.

***1-chloro-4-(2-phenylallyl)benzene* 27**

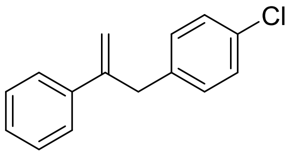

Prepared according to the ***General Procedure C*** to afford **27** (21.0 mg, 92% yield) as a colourless oil.

NMR and HRMS data for the substrate **27**:

^{1}H NMR (600 MHz, CDCl$_3$) δ (ppm): 7.43-7.41 (m, 2H), 7.32-7.30 (m, 2H), 7.28-7.24 (m, 3H), 7.17 (d, J = 7.8 Hz, 2H), 5.52 (s, 1H), 5.05 (s, 1H), 3.82 (s, 2H).

^{13}C NMR (151 MHz, CDCl$_3$) δ (ppm): 146.5, 140.4, 137.9, 131.8, 130.2, 128.4, 128.3, 127.6, 126.1, 114.8, 41.0.

HRMS (ESI-TOF) m/z: $[M + H]^+$ calculated for $C_{15}H_{14}{}^{35}Cl^+$: 229.0779, found: 229.0781; calculated for $C_{15}H_{14}{}^{37}Cl^+$: 231.0750, found: 231.0750.

***1-bromo-4-(2-phenylallyl)benzene* 28**

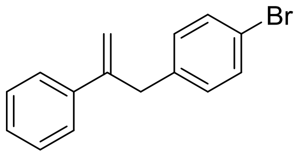

Prepared according to the ***General Procedure C*** to afford **28** (25.1 mg, 92% yield) as a colourless oil.

NMR and HRMS data for the substrate **28**:

^{1}H NMR (600 MHz, $CDCl_3$) δ (ppm): 7.43-7.38 (m, 4H), 7.33-7.30 (m, 2H), 7.27-7.24 (m, 1H), 7.11 (d, J = 9.0 Hz, 2H), 5.52 (s, 1H), 5.05 (s, 1H), 3.80 (s, 2H).

^{13}C NMR (151 MHz, $CDCl_3$) δ (ppm): 146.4, 140.3, 138.5, 131.4, 130.6, 128.3, 127.6, 126.1, 119.9, 114.8, 41.0.

HRMS (ESI-TOF) m/z: $[M + H]^+$ calculated for $C_{15}H_{14}{}^{79}Br^+$: 273.0274, found: 273.0275; calculated for $C_{15}H_{14}{}^{81}Br^+$: 275.0253, found: 275.0255.

***1,2-dimethoxy-4-(2-phenylallyl)benzene* 29**

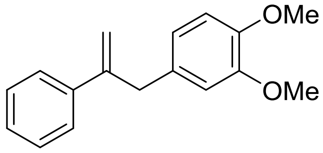

Prepared according to the ***General Procedure C*** to afford **29** (22.4 mg, 88% yield) as a colourless oil.

NMR and HRMS data for the substrate **29**:

^{1}H NMR (600 MHz, $CDCl_3$) δ (ppm): 7.44-7.42 (m, 2H), 7.31-7.28 (m, 2H), 7.26-7.23 (m, 1H), 6.77-6.77 (m, 2H), 6.74 (s, 1H), 5.48 (s, 1H), 5.04 (s, 1H), 3.85 (s, 3H), 3.82 (s, 3H), 3.78 (s, 2H).

^{13}C NMR (151 MHz, $CDCl_3$) δ (ppm): 148.7, 147.3, 147.2, 140.9, 132.0, 128.2, 127.4, 126.1, 120.9, 114.4, 112.1, 111.0, 55.8, 55.7, 41.2.

HRMS (ESI-TOF) m/z: $[M + Na]^+$ calculated for $C_{17}H_{18}O_2Na^+$: 277.1199, found: 277.1193.

***(3-phenoxyprop-1-en-2-yl)benzene* 30**

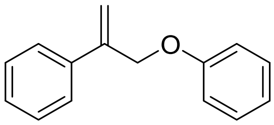

Prepared according to the ***General Procedure C*** to afford **30** (19.6 mg, 93% yield) as a colourless oil.

NMR and HRMS data for the substrate **30**:

^{1}H NMR (600 MHz, CDCl$_3$) δ (ppm): 7.53 (d, *J* = 7.8 Hz, 2H), 7.41-7.39 (m, 2H), 7.36-7.32 (m, 3H), 7.02-7.00 (m, 3H), 5.66 (s, 1H), 5.51 (s, 1H), 4.94 (s, 2H).

^{13}C NMR (151 MHz, CDCl$_3$) δ (ppm): 158.6, 143.0, 138.3, 129.4, 128.4, 128.0, 126.0, 121.0, 114.9, 114.8, 69.7.

HRMS (ESI-TOF) m/z: $[M + H]^+$ calculated for $C_{15}H_{15}O^+$: 211.1118, found: 211.1113.

***hex-1-en-2-ylbenzene* 31**

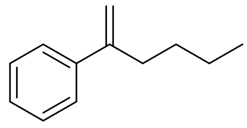

Prepared according to the ***General Procedure C*** to afford **31** (14.4 mg, 90% yield) as a colourless oil.

NMR and HRMS data for the substrate **31**:

^{1}H NMR (600 MHz, CDCl$_3$) δ (ppm): 7.41-7.39 (m, 2H), 7.33-7.31 (m, 2H), 7.27-7.25 (m, 1H), 5.26 (s, 1H), 5.05 (s, 1H), 2.50 (t, *J* = 7.2 Hz, 2H), 1.46-1.41 (m, 2H), 1.37-1.31 (m, 2H), 0.89 (t, *J* = 7.2 Hz, 3H).

^{13}C NMR (151 MHz, CDCl$_3$) δ (ppm): 148.8, 141.5, 128.2, 127.2, 126.1, 112.0, 35.1, 30.4, 22.4, 13.9.

HRMS (ESI-TOF) m/z: $[M + H]^+$ calculated for $C_{12}H_{17}^+$: 161.1325, found: 161.1328.

***but-3-ene-1,3-diyldibenzene* 32**

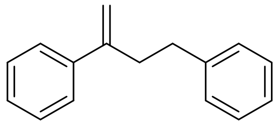

Prepared according to the ***General Procedure C*** to afford **32** (19.6 mg, 94% yield) as a colourless oil.

NMR and HRMS data for the substrate **32**:

^{1}H NMR (600 MHz, CDCl$_3$) δ (ppm): 7.49 (d, *J* = 6.6 Hz, 2H), 7.41-7.39 (m, 2H), 7.35-7.32 (m, 3H), 7.25-7.23 (m, 3H), 5.35 (s, 1H), 5.12 (s, 1H), 2.89-2.86 (m, 2H), 2.84-2.81 (m, 2H).

^{13}C NMR (151 MHz, CDCl$_3$) δ (ppm): 147.8, 141.9, 141.1, 128.4, 128.33, 128.28, 127.4, 126.1, 125.8, 112.6, 37.2, 34.7.

HRMS (ESI-TOF) m/z: $[M + Na]^+$ calculated for $C_{16}H_{16}Na^+$: 231.1145, found: 231.1139.

***(1-cyclohexylvinyl)benzene* 33**

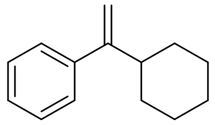

Prepared according to the ***General Procedure C*** to afford **33** (17.0 mg, 91% yield) as a colourless oil.

NMR and HRMS data for the substrate **33**:

^{1}H NMR (600 MHz, CDCl$_3$) δ (ppm): 7.35-7.30 (m, 4H), 7.27-7.24 (m, 1H), 5.14 (s, 1H), 5.01 (s, 1H), 2.45-2.40 (m, 1H), 1.86-1.83 (m, 2H), 1.81-1.77 (m, 2H), 1.73-1.70 (m, 1H), 1.37-1.29 (m, 2H), 1.23-1.14 (m, 3H).

^{13}C NMR (151 MHz, CDCl$_3$) δ (ppm): 155.0, 143.0, 128.1, 127.0, 126.6, 110.3, 42.6, 32.7, 26.8, 26.4.

HRMS (ESI-TOF) m/z: $[M + H]^+$ calculated for $C_{14}H_{19}^+$: 187.1482, found: 187.1481.

***4-(1-phenylvinyl)tetrahydro-2H-pyran* 34**

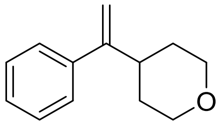

Prepared according to the ***General Procedure C*** to afford **34** (17.5 mg, 93% yield) as a colourless oil.

NMR and HRMS data for the substrate **34**:

^{1}H NMR (600 MHz, CDCl$_3$) δ (ppm): 7.34-7.32 (m, 4H), 7.30-7.26 (m, 1H), 5.20 (s, 1H), 5.04 (s, 1H), 4.04-4.02 (m, 2H), 3.48 (t, J = 11.4 Hz, 2H), 2.70-2.66 (m, 1H), 1.73-1.71 (m, 2H), 1.60-1.53 (m, 2H).

^{13}C NMR (151 MHz, CDCl$_3$) δ (ppm): 153.1, 142.0, 128.2, 127.2, 126.6, 111.1, 68.3, 39.6, 32.3.

HRMS (ESI-TOF) m/z: $[M + H]^+$ calculated for $C_{13}H_{17}O^+$: 189.1274, found: 189.1281.

***tert-butyl 4-(1-phenylvinyl)piperidine-1-carboxylate* 35**

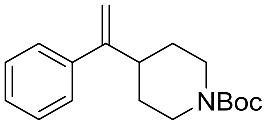

Prepared according to the ***General Procedure B*** to afford **35** (24.4 mg, 85% yield) as a colourless oil.

NMR and HRMS data for the substrate **35**:

^{1}H NMR (600 MHz, $CDCl_3$) δ (ppm): 7.34-7.31 (m, 4H), 7.28-7.25 (m, 1H), 5.18 (s, 1H), 5.01 (s, 1H), 4.17-3.98 (m, 2H), 2.74-2.64 (m, 2H), 2.64-2.54 (m, 1H), 1.78-1.76 (m, 2H), 1.45 (s, 9H), 1.39-1.33 (m, 2H).

^{13}C NMR (151 MHz, $CDCl_3$) δ (ppm): 154.8, 153.0, 142.1, 128.2, 127.2, 126.6, 111.2, 79.3, 43.9, 40.7, 31.4, 28.4.

HRMS (ESI-TOF) m/z: $[M + Na]^+$ calculated for $C_{18}H_{25}NO_2Na^+$: 310.1778, found: 310.1783.

***(3-methylbut-1-en-2-yl)benzene* 36**

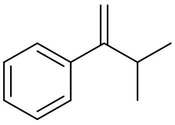

Prepared according to the ***General Procedure C*** to afford **36** (13.2 mg, 90% yield) as a colourless oil.

NMR and HRMS data for the substrate **36**:

^{1}H NMR (600 MHz, $CDCl_3$) δ (ppm): 7.35-7.30 (m, 4H), 7.27-7.25 (m, 1H), 5.14 (s, 1H), 5.03 (s, 1H), 2.86-2.81 (m, 1H), 1.10 (d, J = 6.6 Hz, 6H).

^{13}C NMR (151 MHz, $CDCl_3$) δ (ppm): 155.8, 142.8, 128.1, 127.0, 126.6, 110.0, 32.3, 22.0.

HRMS (ESI-TOF) m/z: $[M + H]^+$ calculated for $C_{11}H_{15}^+$: 147.1169, found: 147.1159.

***(3,3-dimethylbut-1-en-2-yl)benzene* 37**

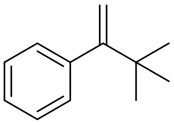

Prepared according to the ***General Procedure B*** to afford **37** (14.6 mg, 91% yield) as a colourless oil.

NMR and HRMS data for the substrate **37**:

^{1}H NMR (600 MHz, CDCl$_3$) δ (ppm): 7.31-7.25 (m, 3H), 7.15 (d, *J* = 7.8 Hz, 2H), 5.19 (s, 1H), 4.78 (s, 1H), 1.13 (s, 9H).

^{13}C NMR (151 MHz, CDCl$_3$) δ (ppm): 159.9, 143.5, 129.0, 127.3, 126.2, 111.5, 36.1, 29.6.

HRMS (ESI-TOF) m/z: [M + H]$^+$ calculated for $C_{12}H_{17}^+$: 161.1325, found: 161.1323.

***4-(3-phenylprop-1-en-2-yl)-N,N-dipropylbenzenesulfonamide* 38**

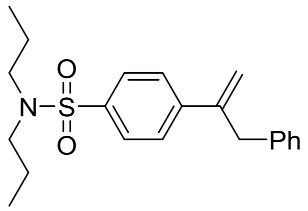

Prepared according to the ***General Procedure C*** to afford **38** (32.2 mg, 90% yield) as a colourless oil.

NMR and HRMS data for the substrate **38**:

^{1}H NMR (600 MHz, CDCl$_3$) δ (ppm): 7.70 (d, *J* = 8.4 Hz, 2H), 7.52 (d, *J* = 8.4 Hz, 2H), 7.28-7.26 (m, 2H), 7.20-7.18 (m, 3H), 5.57 (s, 1H), 5.17 (s, 1H), 3.84 (s, 2H), 3.05 (t, *J* = 7.8 Hz, 4H), 1.57-1.50 (m, 4H), 0.85 (t, *J* = 7.8 Hz, 6H).

^{13}C NMR (151 MHz, CDCl$_3$) δ (ppm): 145.7, 144.7, 138.8, 138.7, 128.8, 128.5, 127.0, 126.6, 126.3, 116.9, 50.0, 41.5, 22.0, 11.2.

HRMS (ESI-TOF) m/z: [M + Na]$^+$ calculated for $C_{21}H_{27}NSO_2^+$: 380.1655, found: 380.1662.

***((10Z,13Z)-2-methylenenonadeca-10,13-dien-1-yl)benzene* 39**

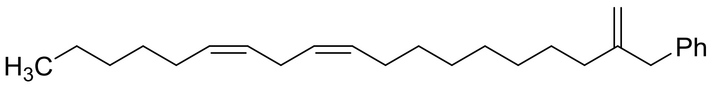

Prepared according to the ***General Procedure C*** to afford **39** (32.1 mg, 91% yield) as a colourless oil.

NMR and HRMS data for the substrate **39**:

^{1}H NMR (600 MHz, CDCl$_3$) δ (ppm): 7.30-7.28 (m, 2H), 7.22-7.19 (m, 3H), 5.42-5.32 (m, 4H), 4.83 (s, 1H), 4.73 (s, 1H), 3.34 (s, 2H), 2.79 (t, *J* = 6.6 Hz, 2H), 2.08-2.04 (m, 4H), 1.97 (t, *J* = 7.2 Hz, 2H), 1.47-1.42 (m, 2H), 1.39-1.28 (m, 14H), 0.90 (t, *J* = 6.6 Hz, 3H).

^{13}C NMR (151 MHz, CDCl$_3$) δ (ppm): 149.2, 139.9, 130.2, 130.1, 129.0, 128.2, 128.0, 127.9, 126.0, 110.9, 43.0, 35.4, 31.5, 29.6, 29.4, 29.35, 29.27, 29.2, 27.6, 27.2, 25.6, 22.6, 14.1.

HRMS (ESI-TOF) m/z: [M + H]$^+$ calculated for $C_{26}H_{41}^+$: 353.3203, found: 353.3200.

***4-(2-phenylallyl)phenyl-2-(4-(2,2-dichlorocyclopropyl)phenoxy)-2-methylpropanoate* 40**

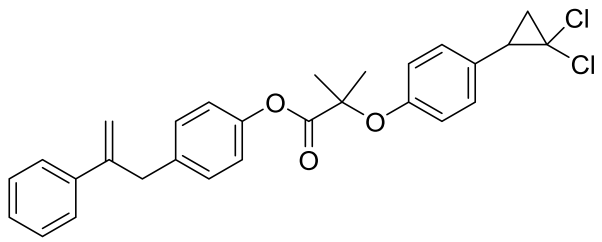

Prepared according to the ***General Procedure B*** to afford **40** (35.1 mg, 73% yield) as a colourless oil.

NMR and HRMS data for the substrate **40**:

^{1}H NMR (600 MHz, CDCl$_3$) δ (ppm): 7.40 (d, *J* = 7.8 Hz, 2H), 7.29-7.27 (m, 2H), 7.23 (t, *J* = 7.2 Hz, 1H), 7.19 (d, *J* = 7.8 Hz, 2H), 7.15 (d, *J* = 9.0 Hz, 2H), 6.92 (d, *J* = 8.4 Hz, 2H), 6.86 (d, *J* = 7.8 Hz, 2H), 5.49 (s, 1H), 5.01 (s, 1H), 3.81 (s, 2H), 2.87-2.83 (m, 1H), 1.97-1.93 (m, 1H), 1.81-1.78 (m, 1H), 1.74 (s, 6H).

^{13}C NMR (151 MHz, CDCl$_3$) δ (ppm): 172.9, 155.0, 148.8, 146.6, 140.5, 137.4, 129.82, 129.78, 128.4, 128.3, 127.5, 126.1, 121.0, 118.5, 114.8, 79.3, 60.8, 41.0, 34.8, 25.8, 25.5.

HRMS (ESI-TOF) m/z: $[M + H]^+$ calculated for $C_{28}H_{27}{}^{35}Cl_2O_3{}^+$: 481.1332, found: 481.1330; calculated for $C_{28}H_{27}{}^{35}Cl^{37}ClO_3{}^+$: 483.1303, found: 483.1302; calculated for $C_{28}H_{27}{}^{37}Cl_2O_3{}^+$: 485.1273, found: 485.1279.

***4-(2-phenylallyl)phenyl 2-(4-isobutylphenyl)propanoate* 41**

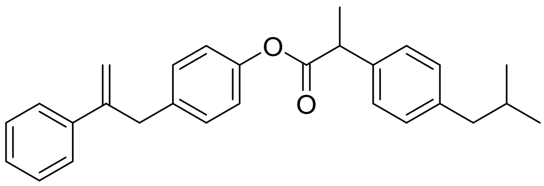

Prepared according to the ***General Procedure B*** to afford **41** (36.7 mg, 92% yield) as a colourless oil.

NMR and HRMS data for the substrate **41**:

^{1}H NMR (600 MHz, CDCl$_3$) δ (ppm): 7.38 (d, *J* = 7.2 Hz, 2H), 7.27-7.24 (m, 4H), 7.21 (t, *J* = 7.2 Hz, 1H), 7.16 (d, *J* = 8.4 Hz, 2H), 7.11 (d, *J* = 8.4 Hz, 2H), 6.88 (d, *J* = 8.4 Hz, 2H), 5.46 (s, 1H), 4.99 (s, 1H), 3.89 (q, *J* = 7.2 Hz, 1H), 3.78 (s, 2H), 2.45 (d, *J* = 6.6 Hz, 2H), 1.87-1.82 (m, 1H), 1.57 (d, *J* = 6.6 Hz, 3H), 0.89 (d, *J* = 6.6 Hz, 6H).

^{13}C NMR (151 MHz, CDCl$_3$) δ (ppm): 173.3, 149.2, 146.7, 140.8, 140.6, 137.3, 136.9, 129.7, 129.5, 128.3, 127.5, 127.2, 126.1, 121.2, 114.7, 45.2, 45.0, 41.0, 30.2, 22.4, 18.5.

HRMS (ESI-TOF) m/z: $[M + H]^+$ calculated for $C_{28}H_{31}O_2{}^+$: 399.2319, found:399.2311.

***(3R,4S)-4-(4-(benzyloxy)phenyl)-1-(4-fluorophenyl)-3-(3-phenylbut-3-en-1-yl)azetidin-2-one* 42**

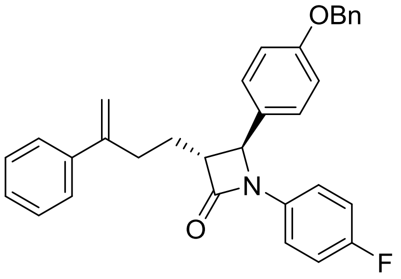

Prepared according to the ***General Procedure B*** to afford **42** (43.0 mg, 90% yield) as a colourless oil.

NMR and HRMS data for the substrate **42**:

^{1}H NMR (600 MHz, CDCl$_3$) δ (ppm): 7.42 (d, *J* = 7.8 Hz, 2H), 7.40-7.36 (m, 4H), 7.34-7.30 (m, 3H), 7.27-7.22 (m, 5H), 6.97 (d, *J* = 8.4 Hz, 2H), 6.91 (t, *J* = 8.4 Hz, 2H), 5.25 (s, 1H), 5.05 (s, 2H), 5.00 (s, 1H), 4.57 (d, *J* = 1.8 Hz, 1H), 3.15-3.13 (m, 1H), 2.76-2.66 (m, 2H), 2.12-2.06 (m, 1H), 2.01-1.94 (m, 1H).

^{13}C NMR (151 MHz, CDCl$_3$) δ (ppm): 167.5, 159.0, 158.9 (C-F, $^{1}J_{C\text{-}F}$ = 244.3 Hz), 147.1, 140.5, 136.7, 134.0, 129.8, 128.6, 128.4, 128.1, 127.6, 127.5, 127.2, 126.1, 118.3 (C-F, $^{3}J_{C\text{-}F}$ = 8.6 Hz), 115.7 (C-F, $^{2}J_{C\text{-}F}$ = 21.7 Hz), 115.5, 113.3, 70.1, 61.1, 60.2, 32.8, 27.7.

^{19}F NMR (564 MHz, CDCl$_3$) δ (ppm): -118.2.

HRMS (ESI-TOF) m/z: [M + H]$^+$ calculated for $C_{32}H_{29}FNO_2^+$: 478.2177, found: 478.2174.

***1-(3-phenylprop-1-en-2-yl)benzene-2,3,4,5,6-d5* 44**

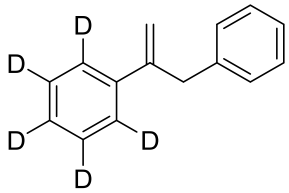

Prepared according to the ***General Procedure C*** to afford **44** (18.3 mg, 92% yield) as a colourless oil.

NMR and HRMS data for the substrate **44**:

^{1}H NMR (600 MHz, CDCl$_3$) δ (ppm): 7.30-7.28 (m, 2H), 7.25 (d, *J* = 6.6 Hz, 2H), 7.20 (t, *J* = 7.2 Hz, 1H), 5.52 (s, 1H), 5.04 (s, 1H), 3.86 (s, 2H).

^{13}C NMR (151 MHz, CDCl$_3$) δ (ppm): 146.8, 140.6, 139.5, 128.9, 128.3, 127.7 (t, *J* = 23.9 Hz), 126.9 (t, *J* = 25.3 Hz), 126.1, 125.7 (t, *J* = 23.9 Hz), 114.5, 41.6.

HRMS (ESI-TOF) m/z: [M + H]$^+$ calculated for $C_{15}H_{10}D_5^+$: 200.1482, found: 200.1483.

***tert-butyl 4-(1-(p-tolyl)vinyl)piperidine-1-carboxylate* 45**

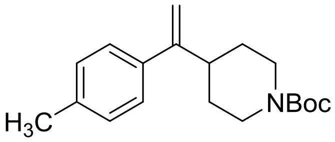

Prepared according to the ***General Procedure B*** to afford **45** (27.7 mg, 92% yield) as a colourless oil.

NMR and HRMS data for the substrate **45**:

^{1}H NMR (600 MHz, $CDCl_3$) δ (ppm): 7.22 (d, *J* = 8.4 Hz, 2H), 7.14 (d, *J* = 8.4 Hz, 2H), 5.16 (s, 1H), 4.98 (s, 1H), 4.17-4.01 (m, 2H), 2.85-2.67 (m, 2H), 2.56-2.53 (m, 1H), 2.35 (s, 3H), 1.78-1.76 (m, 2H), 1.46 (s, 9H), 1.39-1.32 (m, 2H).

^{13}C NMR (151 MHz, $CDCl_3$) δ (ppm): 154.8, 152.8, 139.2, 137.0, 128.9, 126.5, 110.6, 79.3, 44.7, 40.7, 31.5, 28.4, 21.0.

HRMS (ESI-TOF) m/z: $[M + Na]^+$ calculated for $C_{19}H_{27}NO_2Na^+$: 324.1934, found: 324.1934.

***tert-butyl 4-(1-(2-fluorophenyl)vinyl)piperidine-1-carboxylate* 46**

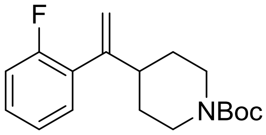

Prepared according to the ***General Procedure B*** to afford **46** (27.5 mg, 90% yield) as a colourless oil.

NMR and HRMS data for the substrate **46**:

^{1}H NMR (600 MHz, $CDCl_3$) δ (ppm): 7.27-7.24 (m, 1H), 7.18-7.15 (m, 1H), 7.10 (t, *J* = 7.2 Hz, 1H), 7.04 (t, *J* = 9.0 Hz, 1H), 5.20 (s, 1H), 5.10 (s, 1H), 4.29-3.98 (m, 2H), 2.72-2.64 (m, 2H), 2.53-2.49 (m, 1H), 1.78-1.76 (m, 2H), 1.46 (s, 9H), 1.39-1.30 (m, 2H).

^{13}C NMR (151 MHz, $CDCl_3$) δ (ppm): 159.6 (C-F, $^1J_{C-F}$ = 245.8 Hz), 154.8 148.9, 130.5 (C-F, $^3J_{C-F}$ = 4.4 Hz), 130.2 (C-F, $^2J_{C-F}$ = 15.9 Hz), 128.8 (C-F, $^3J_{C-F}$ = 7.2 Hz), 123.8 (C-F, $^3J_{C-F}$ = 2.9 Hz), 115.5 (C-F, $^2J_{C-F}$ = 23.1Hz), 114.1, 79.3,44.4, 41.5, 31.1, 28.4.

^{19}F NMR (564 MHz, $CDCl_3$) δ (ppm): -115.2.

HRMS (ESI-TOF) m/z: $[M + Na]^+$ calculated for $C_{18}H_{24}FNO_2Na^+$: 328.1684, found: 328.1680.

5. The Synthetic Application

5.1 The "one-pot" diversification

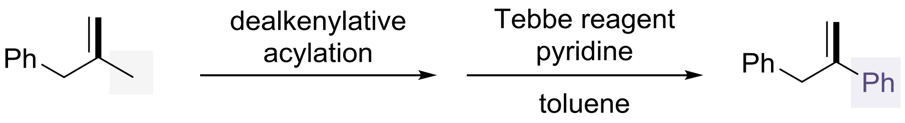

1a 0.1 mmol　　one-pot　　**5**, 61% yield

A dry glass tube was charged with alkene **1a** (0.1 mmol), **A** (0.11 mmol), and DCM (2 mL). Then, the resulting mixture was stirred for 7 h at room temperature, monitored by TLC. After the reaction finished, the reaction mixture was concentrated in vacuum to give the crude intermediate **2**, then **3a** (0.2 mmol), NHC **N1** (0.02 mmol), **PC1** (0.002 mmol) and K_2CO_3 (0.2 mmol) were added. The reaction tube was subjected to three cycles of pressurization and depressurization using dry Ar. Afterwards, 1 mL of MeCN was added, and the reaction mixture was stirred for 8 h under the irradiation of blue LEDs. Then the mixture was concentrated in vacuum. Subsequently, the glass tube was subjected to three cycles of pressurization and depressurization using dry Ar. Under the protection of Ar atmosphere, toluene (1 mL), the Tebbe reagent (0.3 mmol) and pyridine (0.3 mmol) were added successively at −40 ℃. Then, the reaction mixture was raised to 40 ℃ slowly, and stirred for 5 h. After the reaction finished, the reaction mixture diluted with water and extracted with dichloromethane. The combined organic phases were washed with brine, dried over anhydrous Na_2SO_4, filtered and concentrated in vacuum. The crude residue was then purified by column chromatography on silica gel eluting with petroleum ether/ethyl acetate to provide 61% overall yield of alkene product **5**.

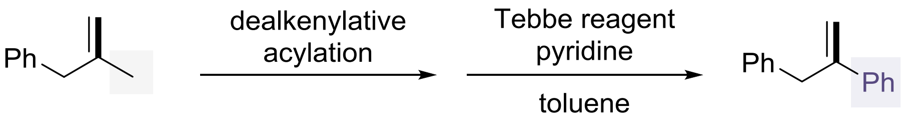

1a 2 mmol　　one-pot　　**5**, 55%

A dry glass tube was charged with alkene **1a** (2 mmol), **A** (2.2 mmol), and DCM (10 mL). Then, the resulting mixture was stirred for 7 h at room temperature, monitored by TLC. After the reaction finished, the reaction mixture was concentrated in vacuum to give the crude intermediate **2**, then **3a** (4 mmol), NHC **N1** (0.4 mmol), **PC1** (0.04 mmol) and K_2CO_3 (4.4 mmol) were added. The reaction tube was subjected to three cycles of pressurization and depressurization using dry Ar. Afterwards, 15 mL of MeCN was added, and the reaction mixture was stirred for 12 h under the irradiation of blue LEDs. Then the mixture was concentrated in

vacuum. Subsequently, the glass tube was subjected to three cycles of pressurization and depressurization using dry Ar. Under the protection of Ar atmosphere, toluene (15 mL), the Tebbe reagent (6 mmol) and pyridine (6 mmol) were added successively at –40 ℃. Then, the reaction mixture was raised to 40 ℃ slowly, and stirred for 6 h. After the reaction finished, the reaction mixture diluted with water and extracted with dichloromethane. The combined organic phases were washed with brine, dried over anhydrous Na_2SO_4, filtered and concentrated in vacuum. The crude residue was then purified by column chromatography on silica gel eluting with petroleum ether/ethyl acetate to provide 55% overall yield of alkene product **5**.

5.2 The"one-pot" late-stage diversification

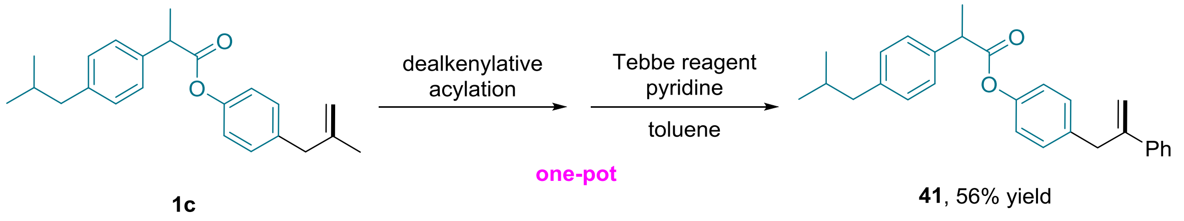

A dry glass tube was charged with alkene **1c** (0.1 mmol), **A** (0.11 mmol), and DCM (2 mL). Then, the resulting mixture was stirred for 7 h at room temperature, monitored by TLC. After the reaction finished, the reaction mixture was concentrated in vacuum to give the crude intermediate **2**, then **3a** (0.2 mmol), NHC **N1** (0.02 mmol), **PC1** (0.002 mmol) and K_2CO_3 (0.2 mmol) were added. The reaction tube was subjected to three cycles of pressurization and depressurization using dry Ar. Afterwards, 1 mL of MeCN was added, and the reaction mixture was stirred for 8 h under the irradiation of blue LEDs. Then the mixture was concentrated in vacuum. Subsequently, the glass tube was subjected to three cycles of pressurization and depressurization using dry Ar. Under the protection of Ar atmosphere, toluene (1 mL), the Tebbe reagent (0.3 mmol.) and pyridine (0.3 mmol) were added successively at –40 ℃. Then, the reaction mixture was raised to 40 ℃ slowly, and stirred for 5 h. After the reaction finished, the reaction mixture diluted with water and extracted with dichloromethane. The combined organic phases were washed with brine, dried over anhydrous Na_2SO_4, filtered and concentrated in vacuum. The crude residue was then purified by column chromatography on silica gel eluting with petroleum ether/ethyl acetate to provide 56% overall yield of alkene product **41**.

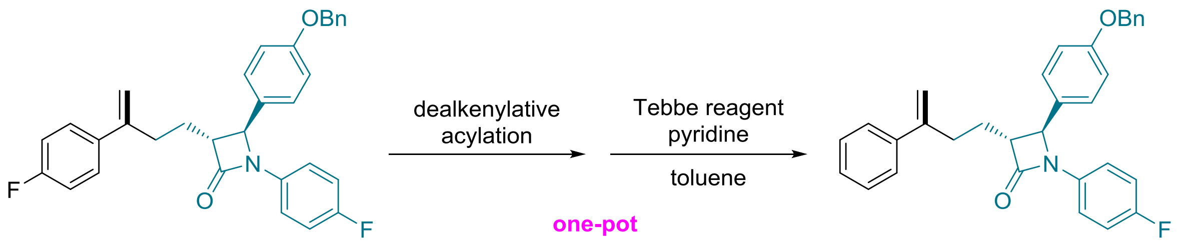

1d **42**, 47% yield

A dry glass tube was charged with alkene **1d** (0.1 mmol), **A** (0.11 mmol), and DCM (2 mL). Then, the resulting mixture was stirred for 7 h at room temperature, monitored by TLC. After the reaction finished, the reaction mixture was concentrated in vacuum to give the crude intermediate **2**, then **3a** (0.2 mmol), NHC **N1** (0.02 mmol), **PC1** (0.002 mmol) and K_2CO_3 (0.2 mmol) were added. The reaction tube was subjected to three cycles of pressurization and depressurization using dry Ar. Afterwards, 1 mL of MeCN was added, and the reaction mixture was stirred for 8 h under the irradiation of blue LEDs. Then the mixture was concentrated in vacuum. Subsequently, the glass tube was subjected to three cycles of pressurization and depressurization using dry Ar. Under the protection of Ar atmosphere, toluene (1 mL), the Tebbe reagent (0.3 mmol.) and pyridine (0.3 mmol) were added successively at –40 ℃. Then, the reaction mixture was raised to 40 ℃ slowly, and stirred for 5 h. After the reaction finished, the reaction mixture diluted with water and extracted with dichloromethane. The combined organic phases were washed with brine, dried over anhydrous Na_2SO_4, filtered and concentrated in vacuum. The crude residue was then purified by column chromatography on silica gel eluting with petroleum ether/ethyl acetate to provide 47% overall yield of alkene product **42**.

5.3 The convergent synthesis starting from a mixture of alkene compounds

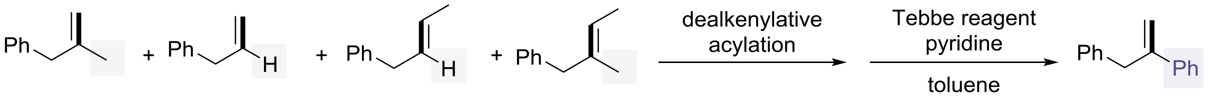

1a **47** **48** **49** 63% yield 90% yield **5**

A dry glass tube was charged with alkene **1a** (0.1 mmol), **47** (0.1 mmol), **48** (0.1 mmol), **49** (0.1 mmol), **A** (0.44 mmol), and DCM (5 mL). Then, the resulting mixture was stirred for 7 h at room temperature, monitored by TLC. After the reaction finished, the reaction mixture was concentrated in vacuum to give the crude intermediate **2**, then **3a** (0.8 mmol), NHC **N1** (0.08 mmol), **PC1** (0.008 mmol) and K_2CO_3 (0.8 mmol) were added. The reaction tube was subjected to three cycles of pressurization and depressurization using dry Ar. Afterwards, 5 mL of MeCN was added, and the reaction mixture was stirred for 10 h under the irradiation of blue LEDs. Then the mixture was concentrated in vacuum, and purified by column chromatography on silica gel eluting from petroleum ether/ethyl acetate (400:1 to 100:1) to afford the

corresponding ketone **4a** in 63% yield. Subsequently, to a 35 mL over-dried Schlenck tube was subjected to three cycles of pressurization and depressurization using dry Ar. Under the protection of Ar atmosphere, the Tebbe reagent (3 eq.), toluene (5 mL) and pyridine (3 eq.) were added successively at –40 ℃. The reaction mixture was stirred for 5 min. After that, the ketone **4a** (1 equiv.) was added at the same temperature. Then, the reaction mixture was raised to 40 ℃ slowly, and stirred for 5 h. After the reaction finished, the reaction mixture diluted with water and extracted with dichloromethane. The combined organic phases were washed with brine, dried over anhydrous Na_2SO_4, filtered and concentrated in vacuum. The crude residue was then purified by column chromatography on silica gel eluting with petroleum ether/ethyl acetate to provide the alkene product **5** in 90% yield as a colourless oil.

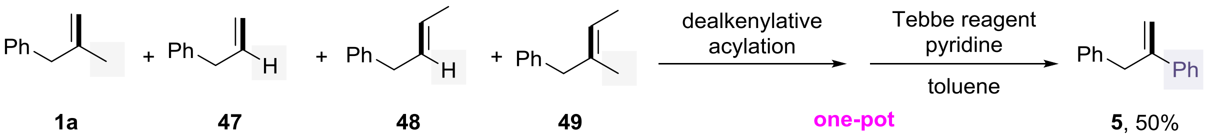

A dry glass tube was charged with alkene **1a** (0.1 mmol), **47** (0.1 mmol), **48** (0.1 mmol), **49** (0.1 mmol), **A** (0.44 mmol), and DCM (5 mL). Then, the resulting mixture was stirred for 7 h at room temperature, monitored by TLC. After the reaction finished, the reaction mixture was concentrated in vacuum to give the crude intermediate **2**, then **3a** (0.8 mmol), NHC **N1** (0.08 mmol), **PC1** (0.008 mmol) and K_2CO_3 (0.8 mmol) were added. The reaction tube was subjected to three cycles of pressurization and depressurization using dry Ar. Afterwards, 5 mL of MeCN was added, and the reaction mixture was stirred for 10 h under the irradiation of blue LEDs. Then the mixture was concentrated in vacuum. Subsequently, the glass tube was subjected to three cycles of pressurization and depressurization using dry Ar. Under the protection of Ar atmosphere, toluene (5 mL), the Tebbe reagent (1.2 mmol) and pyridine (1.2 mmol) were added successively at –40 ℃. Then, the reaction mixture was raised to 40 ℃ slowly, and stirred for 5 h. After the reaction finished, the reaction mixture diluted with water and extracted with dichloromethane. The combined organic phases were washed with brine, dried over anhydrous Na_2SO_4, filtered and concentrated in vacuum. The crude residue was then purified by column chromatography on silica gel eluting with petroleum ether/ethyl acetate to provide 50% overall yield of alkene product **5**.

5.4 Investigation of other types of alkenes

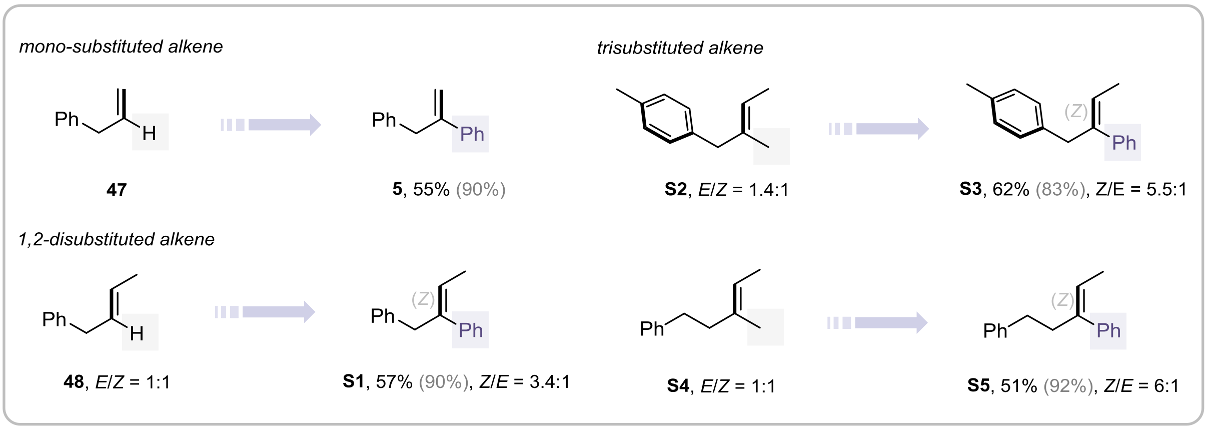

Investigation of mono-substituted alkene

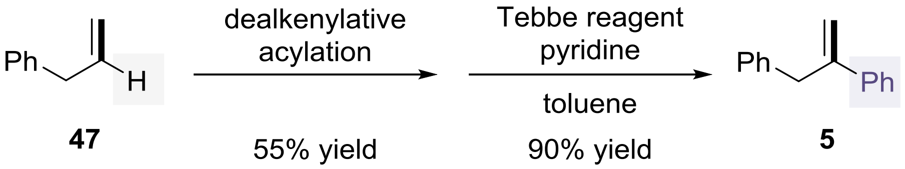

A dry glass tube was charged with alkene **47** (0.1 mmol), **A** (0.11 mmol), and DCM (2 mL). Then, the resulting mixture was stirred for 7 h at room temperature, monitored by TLC. After the reaction finished, the reaction mixture was concentrated in vacuum to give the crude intermediate **2**, then **3a** (0.2 mmol), NHC **N1** (0.02 mmol), **PC1** (0.002 mmol) and K_2CO_3 (0.2 mmol) were added. The reaction tube was subjected to three cycles of pressurization and depressurization using dry Ar. Afterwards, 1 mL of MeCN was added, and the reaction mixture was stirred for 8 h under the irradiation of blue LEDs. Then the mixture was concentrated in vacuum, and purified by column chromatography on silica gel eluting from petroleum ether/ethyl acetate (400:1 to 100:1) to afford the corresponding ketone **4a** in 55% yield. Subsequently, to a 10 mL over-dried Schlenck tube was subjected to three cycles of pressurization and depressurization using dry Ar. Under the protection of Ar atmosphere, the Tebbe reagent (3 eq.), toluene (2 mL) and pyridine (3 eq.) were added successively at −40 ℃. The reaction mixture was stirred for 5 min. After that, the ketone **4a** (1 equiv.) was added at the same temperature. Then, the reaction mixture was raised to 40 ℃ slowly, and stirred for 5 h. After the reaction finished, the reaction mixture diluted with water and extracted with dichloromethane. The combined organic phases were washed with brine, dried over anhydrous Na_2SO_4, filtered and concentrated in vacuum. The crude residue was then purified by column chromatography on silica gel eluting with petroleum ether/ethyl acetate to provide the alkene product **5** in 90% yield as a colourless oil.

Investigation of 1,2-substituted alkene

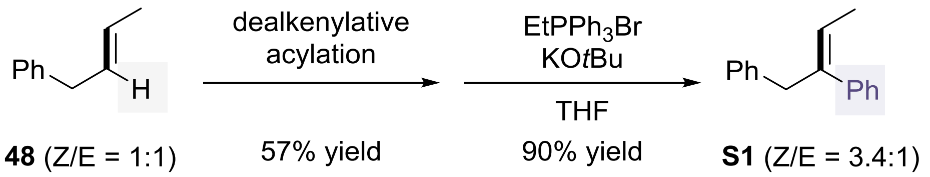

A dry glass tube was charged with alkene **48** (0.1 mmol, Z/E = 1:1), **A** (0.11 mmol), and DCM (2 mL). Then, the resulting mixture was stirred for 7 h at room temperature, monitored by TLC. After the reaction finished, the reaction mixture was concentrated in vacuum to give the crude intermediate **2**, then **3a** (0.2 mmol), NHC **N1** (0.02 mmol), **PC1** (0.002 mmol) and K_2CO_3 (0.2 mmol) were added. The reaction tube was subjected to three cycles of pressurization and depressurization using dry Ar. Afterwards, 1 mL of MeCN was added, and the reaction mixture was stirred for 8 h under the irradiation of blue LEDs. Then the mixture was concentrated in vacuum, and purified by column chromatography on silica gel eluting from petroleum ether/ethyl acetate (400:1 to 100:1) to afford the corresponding ketone **4a** in 57% yield. Subsequently, to a 10 mL over-dried Schlenck tube was added KO*t*Bu (2 equiv.). The Schlenck tube was subjected to three cycles of pressurization and depressurization using dry Ar. After that, under the protection of Ar atmosphere, dry THF (1 mL) and $EtPPh_3Br$ (2 equiv.) were successively added. The suspension was stirred at room temperature for 1 h, and then the ketone **4a** (1 equiv.) was added under Ar atmosphere. The resulting mixture was stirred at 50 ℃ for 5 h. After the reaction finished, the mixture was concentrated in vacuum, and purified by column chromatography on silica gel eluting from petroleum ether/ethyl acetate to provide the alkene product **S1** in 90% yield (Z/E = 3.4:1) as a colourless oil. The Z/E configuration of **S1** was determined by NOEDS analysis.

***(Z)-but-2-ene-1,2-diyldibenzene* S1**

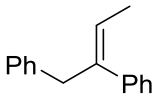

NMR and HRMS data for the substrate **S1**:

^{1}H NMR (600 MHz, $CDCl_3$) δ (ppm): 7.28 (t, J = 7.2 Hz, 2H), 7.25-7.23 (m, 3H), 7.20 (t, J = 7.2 Hz, 1H), 7.14 (d, J = 7.2 Hz, 2H), 7.10 (d, J = 7.8 Hz, 2H), 5.56 (q, J = 7.2 Hz, 1H), 3.65 (s, 2H), 1.60 (d, J = 7.2 Hz, 3H).

^{13}C NMR (151 MHz, $CDCl_3$) δ (ppm): 140.81, 140.76, 139.9, 129.1, 128.6, 128.1, 127.9, 126.4, 125.9, 123.3,, 45.5, 14.8.

HRMS (APCI-TOF) m/z: $[M + H]^+$ calculated for $C_{16}H_{17}^+$: 209.1325, found: 209.1324.

Investigation of tridisubstituted alkene

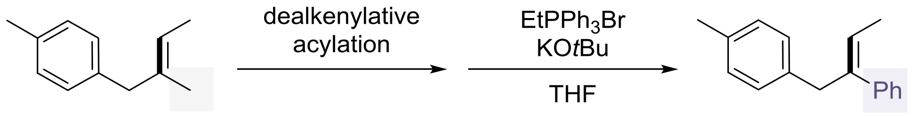

S2 (Z/E = 1.4:1) **S3** (Z/E = 5.5:1)

A dry glass tube was charged with alkene **S2** (0.1 mmol, Z/E = 1.4:1), **A** (0.11 mmol), and DCE (2 mL). Then, the resulting mixture was stirred for 7 h at 70 ℃, monitored by TLC. After the reaction finished, the reaction mixture was concentrated in vacuum to give the crude intermediate **2**, then **3a** (0.2 mmol), NHC **N1** (0.02 mmol), **PC1** (0.002 mmol) and K_2CO_3 (0.2 mmol) were added. The reaction tube was subjected to three cycles of pressurization and depressurization using dry Ar. Afterwards, 1 mL of MeCN was added, and the reaction mixture was stirred for 8 h under the irradiation of blue LEDs. Then the mixture was concentrated in vacuum, and purified by column chromatography on silica gel eluting from petroleum ether/ethyl acetate (400:1 to 100:1) to afford the corresponding ketone **4v** in 62% yield as a colourless oil. Subsequently, to a 10 mL over-dried Schlenck tube was added KO*t*Bu (2 equiv.). The Schlenck tube was subjected to three cycles of pressurization and depressurization using dry Ar. After that, under the protection of Ar atmosphere, dry THF (1 mL) and $EtPPh_3Br$ (2 equiv.) were successively added. The suspension was stirred at room temperature for 1 h, and then the ketone **4v** (1 equiv.) was added under Ar atmosphere. The resulting mixture was stirred at 50 ℃ for 5 h. After the reaction finished, the mixture was concentrated in vacuum, and purified by column chromatography on silica gel eluting from petroleum ether/ethyl acetate to provide the alkene product **S3** in 83% yield (Z/E = 5.5:1) as a colourless oil. The Z/E configuration of **S3** was determined by NOEDS analysis.

(Z)-1-methyl-4-(2-phenylbut-2-en-1-yl)benzene S3

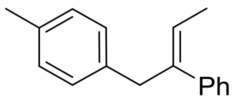

NMR and HRMS data for the substrate **S3**:

^{1}H NMR (600 MHz, $CDCl_3$) δ (ppm): 7.29-7.26 (m, 2H), 7.20-7.18 (m, 1H), 7.12 (d, *J* = 6.0 Hz, 2H), 7.05-7.05 (m, 4H), 5.57-5.55 (m, 1H), 3.63 (s, 2H), 2.30 (s, 3H), 1.61 (d, *J* = 5.4 Hz, 3H).

^{13}C NMR (151 MHz, $CDCl_3$) δ (ppm): 141.0, 140.8, 136.8, 135.2, 128.9, 128.8, 128.5, 127.9, 126.3, 123.0, 45.0, 21.0, 14.7.

HRMS (ESI-TOF) m/z: $[M + Na]^+$ calculated for $C_{17}H_{18}Na^+$: 245.1301, found: 245.1301.

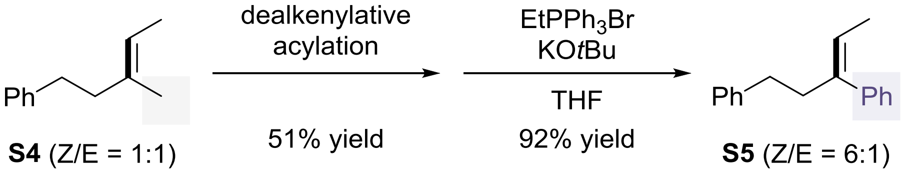

S4 (Z/E = 1:1) **S5** (Z/E = 6:1)

A dry glass tube was charged with alkene **S4** (0.1 mmol, Z/E = 1:1), **A** (0.11 mmol), and DCE (2 mL). Then, the resulting mixture was stirred for 7 h at 70 ℃, monitored by TLC. After the reaction finished, the reaction mixture was concentrated in vacuum to give the crude intermediate **2**, then **3a** (0.2 mmol), NHC **N1** (0.02 mmol), **PC1** (0.002 mmol) and K_2CO_3 (0.2 mmol) were added. The reaction tube was subjected to three cycles of pressurization and depressurization using dry Ar. Afterwards, 1 mL of MeCN was added, and the reaction mixture was stirred for 8 h under the irradiation of blue LEDs. Then the mixture was concentrated in vacuum, and purified by column chromatography on silica gel eluting from petroleum ether/ethyl acetate (400:1 to 100:1) to afford the corresponding ketone **4ab** in 51% yield as a colourless oil. Subsequently, to a 10 mL over-dried Schlenck tube was added KO*t*Bu (2 equiv.). The Schlenck tube was subjected to three cycles of pressurization and depressurization using dry Ar. After that, under the protection of Ar atmosphere, dry THF (1 mL) and $EtPPh_3Br$ (2 equiv.) were successively added. The suspension was stirred at room temperature for 1 h, and then the ketone **4ab** (1 equiv.) was added under Ar atmosphere. The resulting mixture was stirred at 50 ℃ for 5 h. After the reaction finished, the mixture was concentrated in vacuum, and purified by column chromatography on silica gel eluting from petroleum ether/ethyl acetate to provide the alkene product **S5** in 92% yield (Z/E = 6:1) as a colourless oil. The Z/E configuration of **S5** was determined by NOEDS analysis.

***(Z)-pent-3-ene-1,3-diyldibenzene* S5**

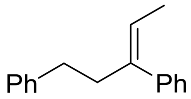

NMR and HRMS data for the substrate **S5**:

^{1}H NMR (600 MHz, $CDCl_3$) δ (ppm): 7.43 (t, J = 7.2 Hz, 2H), 7.34-7.31 (m, 3H), 7.28 (d, J = 7.8 Hz, 2H), 7.24 (d, J = 7.8 Hz, 1H), 7.21 (d, J = 7.8 Hz, 2H), 5.64 (q, J = 6.6 Hz, 1H), 2.73-2.71 (m, 4H), 1.65 (d, J = 6.6 Hz, 3H).

^{13}C NMR (151 MHz, $CDCl_3$) δ (ppm): 142.2, 141.0, 140.7, 128.6, 128.3, 128.2, 128.1, 126.4, 125.6, 121.7, 41.1, 34.8, 14.6.

HRMS (ESI-TOF) m/z: $[M + Na]^+$ calculated for $C_{17}H_{18}Na^+$: 245.1301, found: 245.1298.

5.5 Alkene diversification on multiple substituants

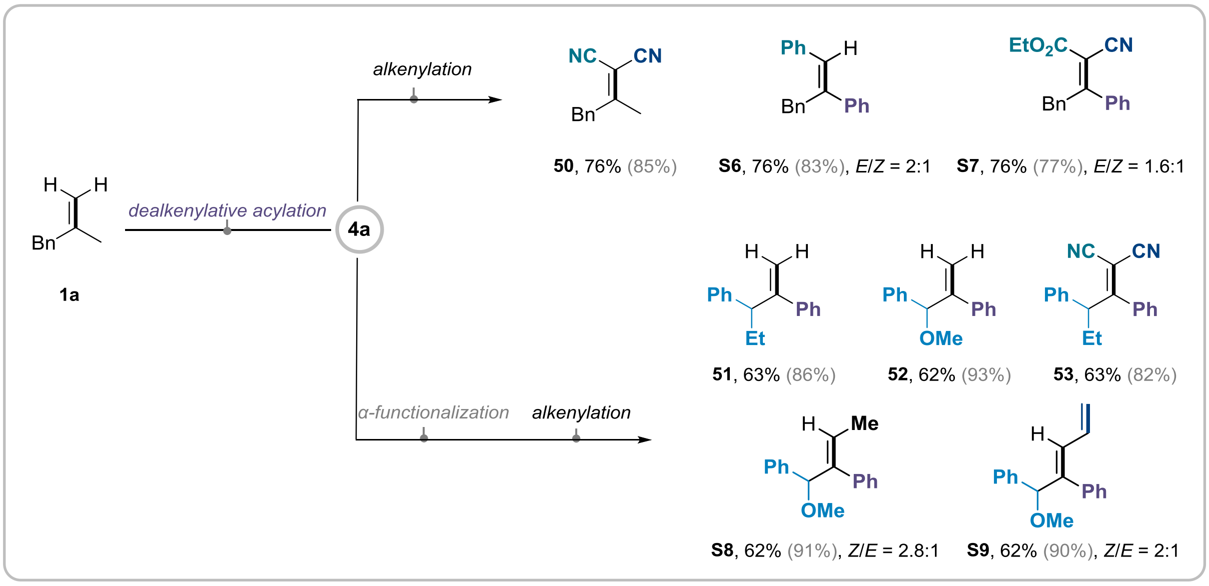

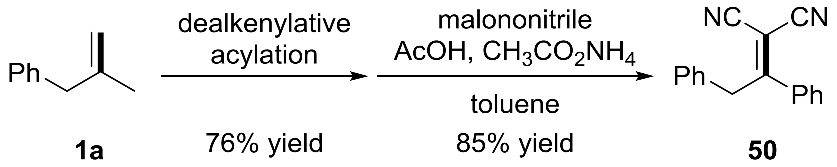

The dealkenylative acylation process is achieved through the ***General Procedure A***. This process can obtain **4a** in 76% yield as a colourless oil. Subsequently, to a 25 mL round-bottom was added malononitrile (1.2 equiv), ammonium acetate (0.2 equiv), the ketone **4a** (1 equiv), acetic acid (1.1 equiv), and toluene (3 mL). The round-bottom was attached to a Dean-Stark apparatus and heated to reflux for 12 h monitored by TLC. After the reaction finished, the reaction mixture diluted with water and extracted by dichloromethane. The combined organic phases were washed with brine, dried over anhydrous Na_2SO_4, filtered and concentrated in vacuum. The crude residue was then purified by column chromatography on silica gel eluting from petroleum ether/ethyl acetate to provide the product **50** in 85% yield as a yellow solid.

2-(1,2-diphenylethylidene)malononitrile 50

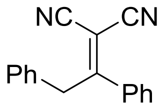

The m.p. of **50** was measured to be 87 – 91 ^{o}C.

NMR and HRMS data for the compound **50**:

^{1}H NMR (600 MHz, CDCl$_3$) δ (ppm): 7.50 (t, *J* = 7.2 Hz, 1H), 7.45-7.43 (m, 2H), 7.40 (d, *J* = 7.2 Hz, 2H), 7.27-7.22 (m, 3H), 7.08-7.06 (m, 2H), 4.27 (s, 2H).

^{13}**C NMR (151 MHz, CDCl$_3$) δ (ppm):** 177.5, 134.7, 134.2, 131.9, 129.0, 128.7, 127.8, 127.7, 112.8, 112.6, 85.4, 43.2.

HRMS (ESI-TOF) m/z: [M + H]$^+$ calculated for $C_{17}H_{13}N_2^+$: 245.1073, found: 245.1074.

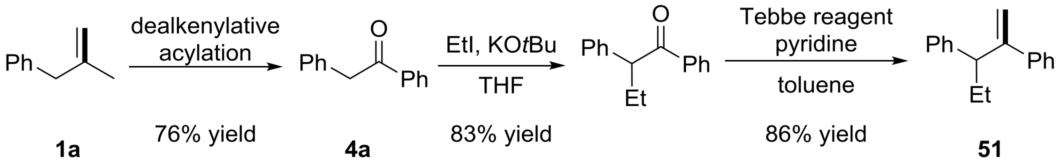

1a 4a 51

The dealkenylative acylation process is achieved through the ***General Procedure A***. This process can obtain **4a** in 76% yield as a colourless oil. Subsequently, to a 10 mL over-dried Schlenck tube was added KO*t*Bu (1.1 equiv.). The Schlenck tube was subjected to three cycles of pressurization and depressurization using dry Ar. After that, under the protection of Ar atmosphere, dry THF (2 mL), EtI (1.2 equiv.) and ketone **4a** (1 equiv.) were successively added at 0 ℃. The solution was stirred for 4 h and then from petroleum ether/ethyl acetate to provide the intermediate ketone in 83% yield. Then, the alkenylation process is achieved through the ***General Procedure B***. This process can obtain **51** in 86% yield as a colourless oil.

pent-1-ene-2,3-diyldibenzene 51

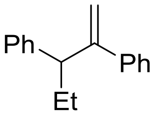

NMR and HRMS data for the substrate **51**:

^{1}**H NMR (600 MHz, CDCl$_3$) δ (ppm):** 7.31-7.26 (m, 8H), 7.24-7.19 (m, 2H), 5.43 (s, 1H), 5.24 (s, 1H), 3.71 (t, *J* = 7.2 Hz, 1H), 2.02-1.97 (m, 1H), 1.88-1.83 (m, 1H), 0.97-0.94 (m, 3H).

^{13}**C NMR (151 MHz, CDCl$_3$) δ (ppm):** 151.8, 143.4, 142.7, 128.2, 128.2, 128.0, 127.0, 126.8, 126.1, 113.0, 52.4, 28.0, 12.7.

HRMS (ESI-TOF) m/z: [M + Na]$^+$ calculated for $C_{17}H_{18}Na^+$: 245.1301, found: 245.1297.

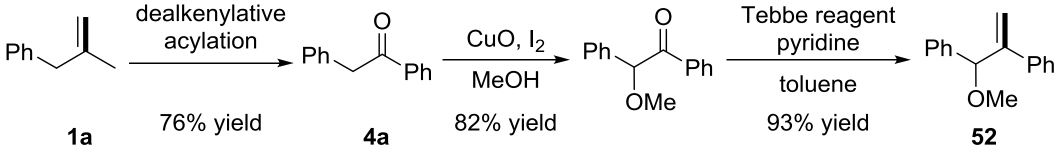

1a 4a 52

The dealkenylative acylation process is achieved through the ***General Procedure A***. This process can obtain **4a** in 76% yield as a colourless oil. Finely powdered CuO (1 equiv.) and I_2 (1 equiv.) were added to a well-stirred soln of the ketone (1 equiv.) in dry MeOH (2 mL). The

mixture was stirred for 5 min and then refluxed for 12 h. After disappearance of the reactant monitored by TLC, the mixture was concentrated in vacuum, and purified by column chromatography on silica gel eluting from petroleum ether/ethyl acetate to afford the corresponding intermediate ketone in 82% yield. Then, the alkenylation process is achieved through the ***General Procedure B***. This process can obtain **52** in 93% yield as a colourless oil.

(1-methoxyprop-2-ene-1,2-diyl)dibenzene 52

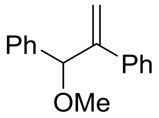

NMR and HRMS data for the substrate **52**:

^{1}H NMR (600 MHz, $CDCl_3$) δ (ppm): 7.50-7.49 (m, 2H), 7.46-7.44 (m, 2H), 7.42-7.39 (m, 2H), 7.37-7.31 (m, 4H), 5.65 (s, 1H), 5.56 (s, 1H), 5.24 (s, 1H), 3.53 (s, 3H).

^{13}C NMR (151 MHz, $CDCl_3$) δ (ppm): 148.0, 140.0, 139.5, 128.1, 128.0, 127.4, 127.4, 127.1, 127.0, 115.0, 85.4, 56.8.

HRMS (ESI-TOF) m/z: $[M + H]^+$ calculated for $C_{16}H_{17}O^+$: 225.1274, found: 225.1278.

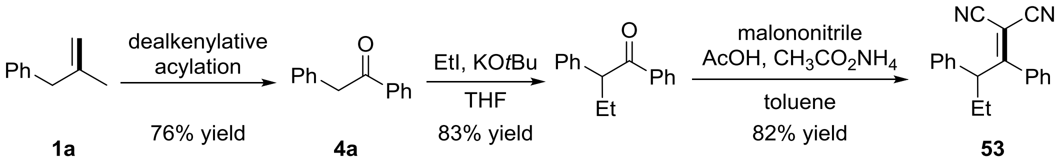

The dealkenylative acylation process is achieved through the ***General Procedure A***. This process can obtain **4a** in 76% yield as a colourless oil. Subsequently, to a 10 mL over-dried Schlenck tube was added KO*t*Bu (1.1 equiv.). The Schlenck tube was subjected to three cycles of pressurization and depressurization using dry Ar. After that, under the protection of Ar atmosphere, dry THF (2 mL), EtI (1.2 equiv.) and ketone **4a** (1 equiv.) were successively added at 0 ℃. The solution was stirred for 4 h and then from petroleum ether/ethyl acetate to provide the intermediate ketone in 83% yield. Then, to a 25 mL round-bottom was added malononitrile (1.2 equiv), ammonium acetate (0.2 equiv), the intermediate ketone (1 equiv), acetic acid (1.1 equiv), and toluene (3 mL). The round-bottom was attached to a Dean-Stark apparatus and heated to reflux for 12 h monitored by TLC. After the reaction finished, the reaction mixture diluted with water and extracted by dichloromethane. The combined organic phases were washed with brine, dried over anhydrous Na_2SO_4, filtered and concentrated in vacuum. The crude residue was then purified by column chromatography on silica gel eluting from petroleum ether/ethyl acetate to provide the product **53** in 82% yield as a colourless oil.

2-(1,2-diphenylbutylidene)malononitrile 53

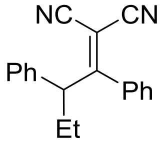

NMR and HRMS data for the substrate **53**:

^{1}H NMR (600 MHz, $CDCl_3$) δ (ppm): 7.42 (t, *J* = 7.2 Hz, 1H), 7.33-7.29 (m, 5H), 7.11-7.09 (m, 2H), 6.72 (d, *J* = 7.2 Hz, 2H), 4.46 (t, *J* = 7.8 Hz, 1H), 1.98-1.94 (m, 1H), 1.88-1.83 (m, 1H), 1.06 (t, *J* = 7.2 Hz, 3H).

^{13}C NMR (151 MHz, $CDCl_3$) δ (ppm): 182.8, 136.8, 133.4, 130.4, 128.9, 128.5, 128.4, 128.2, 127.4, 112.3, 111.9, 87.8, 53.9, 24.5, 12.0.

HRMS (APCI-TOF) m/z: $[M + H]^+$ calculated for $C_{19}H_{17}N_2^+$: 273.1387, found: 273.1382.

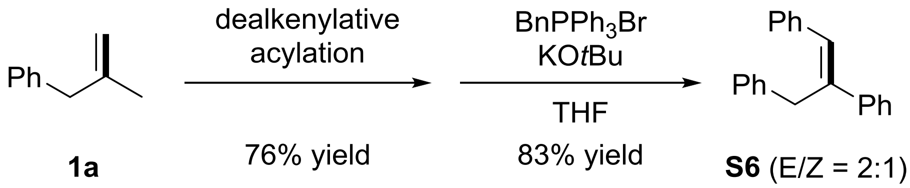

1a **S6** (E/Z = 2:1)

The dealkenylative acylation process is achieved through the ***General Procedure A***. This process can obtain **4a** in 76% yield as a colourless oil. Subsequently, to a 10 mL over-dried Schlenck tube was added KO*t*Bu (2 equiv.). The Schlenck tube was subjected to three cycles of pressurization and depressurization using dry Ar. After that, under the protection of Ar atmosphere, dry THF (1 mL) and $BnPPh_3Br$ (2 equiv.) were successively added. The suspension was stirred at room temperature for 1 h, and then the ketone **4a** (1 equiv.) was added under Ar atmosphere. The resulting mixture was stirred at 50 ℃ for 5 h. After the reaction finished, the mixture was concentrated in vacuum, and purified by column chromatography on silica gel eluting from petroleum ether/ethyl acetate to provide the alkene product **S6** in 83% yield (E/Z = 2:1) as a colourless oil. The Z/E configuration of **S6** was determined by NOEDS analysis.

(E)-prop-1-ene-1,2,3-triyltribenzene S6

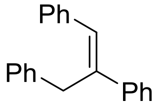

NMR and HRMS data for the compound **S6**:

^{1}H NMR (600 MHz, $CDCl_3$) δ (ppm): 7.54 (d, *J* = 7.2 Hz, 2H), 7.39 (d, *J* = 7.8 Hz, 2H), 7.37-7.30 (m, 2H), 7.29-7.23 (m, 6H), 7.22-7.18 (m, 1H), 7.16 (s, 1H), 7.13-7.08 (m, 2H), 4.18 (s, 2H).

^{13}C NMR (151 MHz, $CDCl_3$) δ (ppm): 142.4, 139.7, 139.0, 137.7, 130.3, 129.2, 129.0, 128.6, 128.5, 128.3, 127.3, 127.0, 126.5, 125.9, 36.1.
HRMS (ESI-TOF) m/z: $[M + Na]^+$ calculated for $C_{21}H_{18}Na^+$: 293.1301, found: 293.1291.

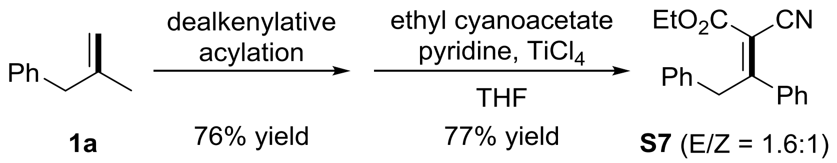

1a **S7** (E/Z = 1.6:1)

The dealkenylative acylation process is achieved through the ***General Procedure A***. This process can obtain **4a** in 76% yield as a colourless oil. Subsequently, To a 25 mL over-dried Schlenck tube was added ethyl cyanoacetate (2 equiv.). The Schlenck tube was subjected to three cycles of pressurization and depressurization using dry Ar. After that, under the protection of Ar atmosphere, dry THF (1 mL), pyridine (2 equiv.) and ketone **4a** (1equiv.) were successively added at 0 ℃. Then, the $TiCl_4$ (0.4 equiv.) was added. The solution was allowed to warm up to room temperature. After the reaction finished, the reaction mixture diluted with water and extracted by dichloromethane. The combined organic phases were washed with brine, dried over anhydrous Na_2SO_4, filtered and concentrated in vacuum. The crude residue was then purified by column chromatography on silica gel eluting from petroleum ether/ethyl acetate to provide the alkene **S7** in 77% yield (E/Z = 1.6:1) as a colourless oil. The Z/E configuration of **S7** was determined by NOEDS analysis.

ethyl (E)-2-cyano-3,4-diphenylbut-2-enoate S7

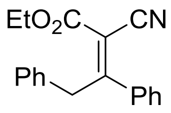

NMR and HRMS data for the substrate **S7**:
^{1}H NMR (600 MHz, $CDCl_3$) δ (ppm): 7.40-7.34 (m, 2H), 7.32-7.29 (m, 2H), 7.23-7.18 (m, 3H), 7.10-7.09 (m, 2H), 6.99-6.98 (m, 1H), 4.52 (s, 2H), 4.39 (q, J = 7.2 Hz, 2H), 1.40 (t, J = 7.2 Hz, 3H).
^{13}C NMR (151 MHz, $CDCl_3$) δ (ppm): 173.1, 161.8, 138.4, 135.8, 128.91, 128.86, 128.3, 127.8, 127.4, 126.7, 115.8, 106.0, 62.1, 40.3, 13.9.
HRMS (ESI-TOF) m/z: $[M + Na]^+$ calculated for $C_{19}H_{17}NO_2Na^+$: 314.1152, found: 314.1156.

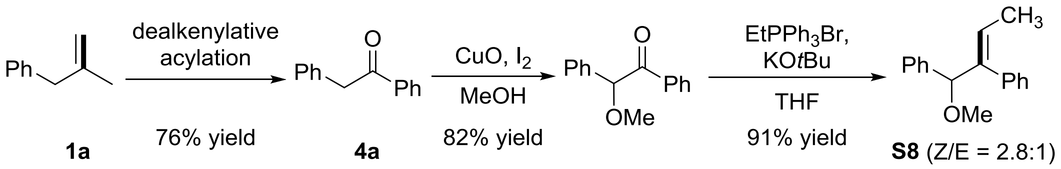

The dealkenylative acylation process is achieved through the ***General Procedure A***. This process can obtain **4a** in 76% yield as a colourless oil. Finely powdered CuO (1 equiv.) and I_2 (1 equiv.) were added to a well-stirred soln of the ketone (1 equiv.) in dry MeOH (2 mL). The mixture was stirred for 5 min and then refluxed for 12 h. After disappearance of the reactant monitored by TLC, the mixture was concentrated in vacuum, and purified by column chromatography on silica gel eluting from petroleum ether/ethyl acetate to afford the corresponding intermediate ketone in 82% yield. Then, to a 10 mL over-dried Schlenck tube was added KO*t*Bu (2 equiv.). The Schlenck tube was subjected to three cycles of pressurization and depressurization using dry Ar. After that, under the protection of Ar atmosphere, dry THF (1 mL) and $EtPPh_3Br$ (2 equiv.) were successively added. The suspension was stirred at room temperature for 1 h, and then the intermediate ketone was added under Ar atmosphere. The resulting mixture was stirred at 50 ℃ for 5 h. Then the mixture was concentrated in vacuum, and purified by column chromatography on silica gel eluting from petroleum ether/ethyl acetate to provide alkene product **S8** in 91% yield (Z/E = 2.8:1) as a colourless oil. The Z/E configuration of **S8** was determined by NOEDS analysis.

(E)-(1-methoxybut-2-ene-1,2-diyl)dibenzene S8

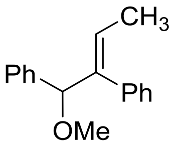

NMR and HRMS data for the substrate **S8**:

^{1}H NMR (600 MHz, $CDCl_3$) δ (ppm): 7.31-7.18 (m, 8H), 6.95 (d, *J* = 6.6 Hz, 2H), 5.95 (q, *J* = 7.2 Hz, 1H), 4.87 (s, 1H), 3.42 (s, 3H), 1.61 (d, *J* = 7.2 Hz, 3H).

^{13}C NMR (151 MHz, $CDCl_3$) δ (ppm): 141.7, 140.4, 138.2, 129.3, 127.9, 127.7, 127.1, 127.0, 126.6, 124.5, 88.0, 56.7, 14.4.

HRMS (ESI-TOF) m/z: $[M + Na]^+$ calculated for $C_{17}H_{18}ONa^+$: 261.1250, found: 261.1258.

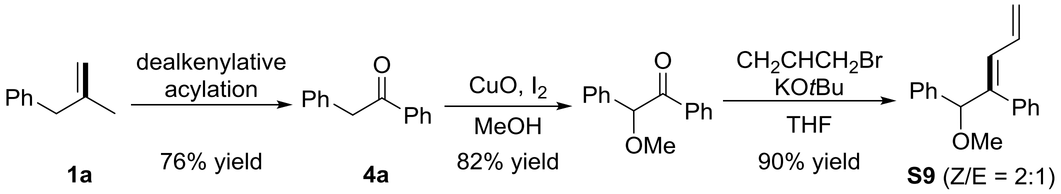

The dealkenylative acylation process is achieved through the ***General Procedure A***. This process can obtain **4a** in 76% yield as a colourless oil. Finely powdered CuO (1 equiv.) and I_2 (1 equiv.) were added to a well-stirred soln of the ketone (1 equiv.) in dry MeOH (2 mL). The mixture was stirred for 5 min and then refluxed for 12 h. After disappearance of the reactant monitored by TLC, the mixture was concentrated in vacuum, and purified by column chromatography on silica gel eluting from petroleum ether/ethyl acetate to afford the corresponding intermediate ketone in 82% yield. Then, to a 10 mL over-dried Schlenck tube was added KO*t*Bu (2 equiv.). The Schlenck tube was subjected to three cycles of pressurization and depressurization using dry Ar. After that, under the protection of Ar atmosphere, dry THF (1 mL) and $CH_2CHCH_2PPh_3Br$ (2 equiv.) were successively added. The suspension was stirred at room temperature for 1 h, and then the intermediate ketone was added under Ar atmosphere. The resulting mixture was stirred at 50 ℃ for 5 h. Then the mixture was concentrated in vacuum, and purified by column chromatography on silica gel eluting from petroleum ether/ethyl acetate to provide alkene product **S9** in 90% yield (Z/E = 2:1) as a colourless oil. The Z/E configuration of **S9** was determined by NOEDS analysis.

(E)-(1-methoxypenta-2,4-diene-1,2-diyl)dibenzene S9

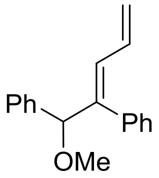

NMR and HRMS data for the substrate **S9**:

^{1}H NMR (600 MHz, $CDCl_3$) δ (ppm): 7.27-7.19 (m, 8H), 6.96 (dd, *J* = 7.2, 1.8 Hz, 2H), 6.49 (d, *J* = 10.8 Hz, 1H), 6.30-6.23 (m, 1H), 5.33 (m, 1H), 5.07 (m, 1H), 4.89 (s, 1H), 3.41 (s, 3H).

^{13}C NMR (151 MHz, $CDCl_3$) δ (ppm): 143.0, 140.0, 137.9, 133.9, 129.4, 128.4, 128.1, 127.8, 127.5, 127.2, 127.1, 118.6, 87.4, 56.9.

HRMS (APCI-TOF) m/z: $[M + H]^+$ calculated for $C_{18}H_{19}O^+$: 251.1341, found: 251.1340.

6. Mechanistic Studies

6.1 The control experiments with TEMPO

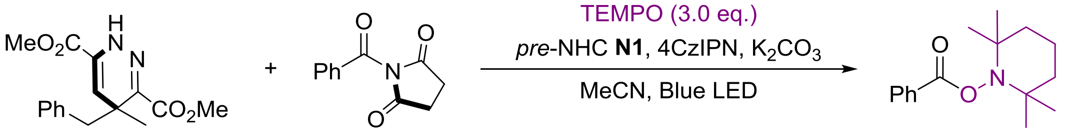

2a **3a**

54 57% yield
4a not observed

To an over-dried Schlenck tube was added substrate **2a** (0.10 mmol), substrate **3a** (0.2 mmol), *pre*-NHC **N1** (20 mol %), 4CzIPN (2 mol %), K_2CO_3 (0.2 mmol) and TEMPO (0.3 mmol). The Schlenck tube was subjected to three cycles of pressurization and depressurization using dry Ar. After that, under the protection of Ar atmosphere, dry MeCN (1 mL) was added, and the reaction mixture was irradiated with blue LEDs. After the reaction had proceeded for 8 hours, the product **4a** was not observed, but the TEMPO-trapped **54** (yellow oil) was detected by NMR and HRMS.

2,2,6,6-tetramethylpiperidin-1-yl benzoate 54

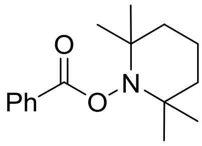

NMR and HRMS data for the substrate **54**:

^{1}H NMR (600 MHz, $CDCl_3$) δ (ppm): 8.11 (d, J = 7.8 Hz, 2H), 7.60 (t, J = 7.2 Hz, 1H), 7.51-7.48 (m, 2H), 1.84-1.79 (m, 2H), 1.76-1.69 (m, 1H), 1.63-1.61 (m, 2H), 1.51-1.48 (m, 1H), 1.31 (s, 6H), 1.16 (s, 6H).

^{13}C NMR (151 MHz, $CDCl_3$) δ (ppm): 165.7, 132.1, 129.0, 128.8, 127.8, 59.7, 38.4, 31.3, 20.1, 16.3.

HRMS (ESI-TOF) m/z: $[M + H]^+$ calculated for $C_{16}H_{24}NO_2^+$: 262.1802, found: 262.1808.

6.2 Radical clock experiment

The preparation steps of substrate **2b**:

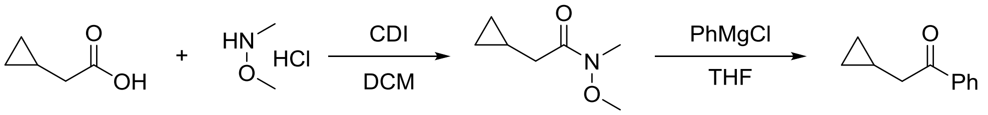

74% yield
(two steps)

The cyclopropylacetic acid (1 eq.) and DCM was added to a dry flask. Then, CDI (1.5 eq.) was added at 0 ℃ and stirred at room temperature for 1 h. After that, N,O-Dimethylhydroxylamine hydrochloride (1.5 eq.) was added to the reaction and stirred for 3 h. After the reaction finished, the reaction mixture diluted with water and extracted with dichloromethane. The combined organic phases were washed with brine, dried over anhydrous Na_2SO_4, filtered and concentrated in vacuum.

Then, the crude residue was add to a 100 mL flask and subjected to three cycles of pressurization and depressurization using dry Ar. THF and Grignard reagents (2 eq.) was added slowly at 0 ℃. Subsequently, the reaction was stirred at room temperature for 6 h. After the reaction finished, the reaction mixture diluted with water and extracted with dichloromethane. The crude residue was then purified by column chromatography on silica gel eluting with petroleum ether/ethyl acetate to provide the intermediate ketone in 74% yield for two steps.

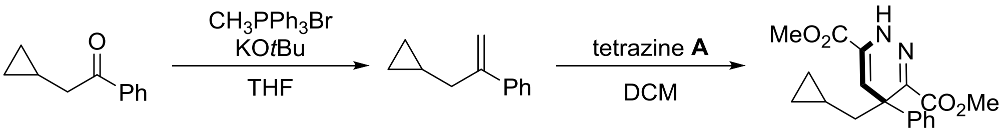

91% yield **2b** 87% yield

To a over-dried Schlenck tube was added KO*t*Bu (2 equiv.). The Schlenck tube was subjected to three cycles of pressurization and depressurization using dry Ar. After that, under the protection of Ar atmosphere, dry THF and CH_3PPh_3Br (2 equiv.) were successively added. The suspension was stirred at room temperature for 1 h, and then the intermediate ketone (1 equiv.) was added under Ar atmosphere. The resulting mixture was stirred at 50 ℃ for 5 h. After the reaction finished, the mixture was concentrated in vacuum, and purified by column chromatography on silica gel eluting from petroleum ether/ethyl acetate to provide the alkene product in 91% yield.

A dry flask was charged with alkene product (1 eq.), **A** (1.1 eq.), and DCM. Then, the resulting mixture was stirred for 7 h at room temperature, monitored by TLC. After the reaction finished, the mixture was concentrated in vacuum, and purified by column chromatography on silica gel eluting from petroleum ether/ethyl acetate to provide **2b** in 87% yield.

The radical clock experiment by using **2b**:

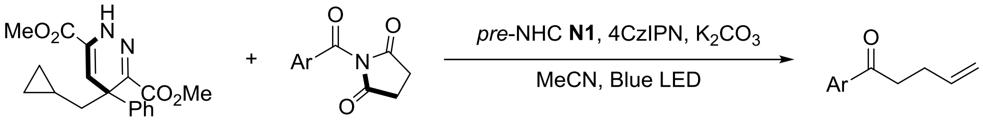

2b **3b** Ar = 4-OMe-C_6H_4 **55**, 31% yield

To an over-dried Schlenck tube was added substrate **2b** (0.10 mmol), substrate **3b** (0.2 mmol), *pre*-NHC **N1** (20 mol %), 4CzIPN (2 mol %) and K_2CO_3 (0.2 mmol). The Schlenck

tube was subjected to three cycles of pressurization and depressurization using dry Ar. After that, under the protection of Ar atmosphere, dry MeCN (1 mL) was added, and the reaction mixture was irradiated with blue LEDs for 8 h. After the reaction finished, the resulting mixture was concentrated in vacuum, and the crude residue was then purified by column chromatography on silica gel eluting with petroleum ether/ethyl acetate (200:1 to 100:1) to provide the product **55** in 31% yield as a colourless oil.

dimethyl-4-(cyclopropylmethyl)-4-phenyl-1,4-dihydropyridazine-3,6-dicarboxylate 2b

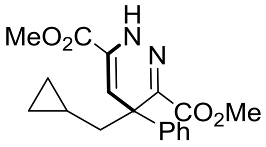

NMR and HRMS data for compound **2b**:

^{1}H NMR (600 MHz, CDCl$_3$) δ (ppm): 8.13 (s, 1H), 7.36-7.31 (m, 4H), 7.23-7.20 (m, 1H), 5.58 (d, *J* = 1.8 Hz, 1H), 3.82 (s, 3H), 3.66 (s, 3H), 2.47-2.43 (m, 1H), 1.97-1.93 (m, 1H), 0.82-0.77 (m, 1H), 0.51-0.48 (m, 2H), 0.21-0.19 (m, 1H), 0.11-0.08 (m, 1H).

^{13}C NMR (151 MHz, CDCl$_3$) δ (ppm): 164.2, 161.9, 148.0, 134.6, 128.3, 127.2, 126.7, 125.1, 116.5, 52.5, 52.0, 44.5, 42.0, 8.4, 5.1, 4.3.

HRMS (ESI-TOF) *m/z*: [M + Na]$^+$ calculated for $C_{18}H_{20}N_2O_4Na^+$: 351.1316, found 351.1312.

1-(4-methoxyphenyl)pent-4-en-1-one 55

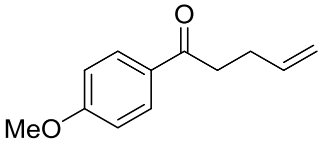

NMR and HRMS data for compound **55**:

^{1}H NMR (600 MHz, CDCl$_3$) δ (ppm): 7.95 (d, *J* = 8.4 Hz, 2H), 6.93 (d, *J* = 8.4 Hz, 2H), 5.94-5.87 (m, 1H), 5.08 (d, *J* = 16.8 Hz, 1H), 5.00 (d, *J* = 10.2 Hz, 1H), 3.87 (s, 3H), 3.02 (t, *J* = 7.8 Hz, 2H), 2.50-2.47 (m, 2H).

^{13}C NMR (151 MHz, CDCl$_3$) δ (ppm): 198.0, 163.4, 137.5, 130.3, 130.0, 115.1, 113.7, 55.4, 37.4, 28.4.

HRMS (ESI-TOF) *m/z*: [M + H]$^+$ calculated for $C_{12}H_{15}O_2^+$: 191.1067, found 191.1062.

6.3 The control experiment with acyl azolium

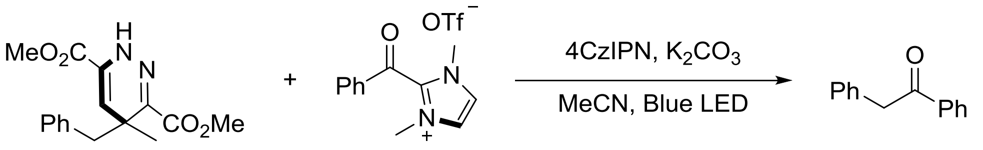

2a **56** **4a**, 73% yield

To an over-dried Schlenck tube was added substrate **2a** (0.10 mmol), acyl azolium **56** (0.2 mmol), 4CzIPN (2 mol %) and K_2CO_3 (0.2 mmol). The Schlenck tube was subjected to three cycles of pressurization and depressurization using dry Ar. After that, under the protection of Ar atmosphere, dry MeCN (1 mL) was added, and the reaction mixture was irradiated with blue LEDs for 8 h. After the reaction finished, the resulting mixture was concentrated in vacuum, and the crude residue was then purified by column chromatography on silica gel eluting with petroleum ether/ethyl acetate (400:1 to 100:1) to provide the product **4a** in 73% yield as a colourless oil.

6.4 The experiment on the variation of yield over time

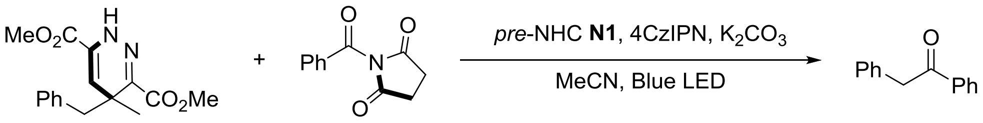

2a **3a** **4a**

The experiment on the variation of yield over time was conducted according to a general procedure. The results indicate that the optimal reaction time for this reaction is 8 hours.

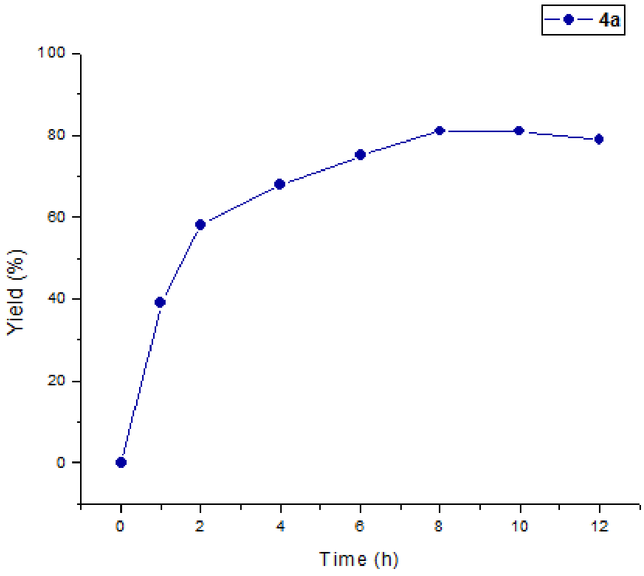

6.5 Light on-off experiments

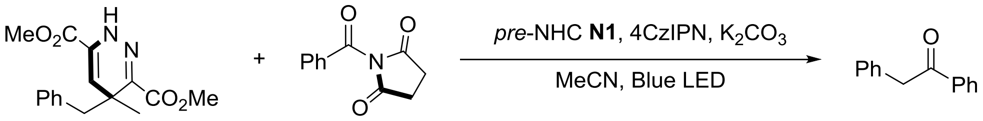

2a **3a** **4a**

The light-on experiments were performed according to the general procedure and set up with six reactions in parallel. The corresponding ketone **4a** yields were calculated by ^{1}H NMR with CH_2Br_2 as internal standard. The light on-off results indicated the reaction proceeded via a catalytic radical mechanism rather than a radical chain pathway.

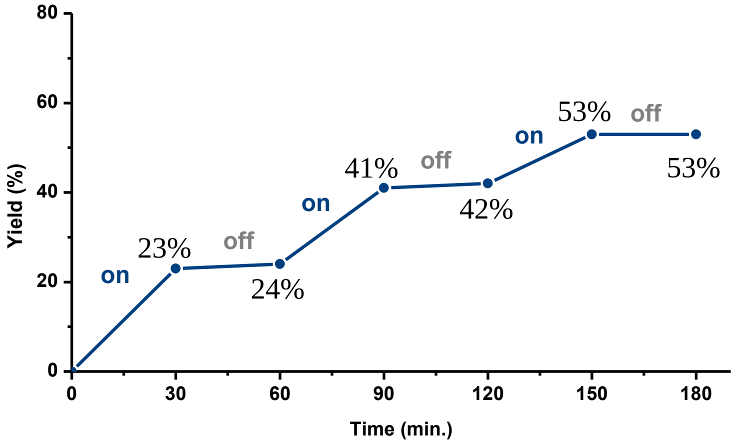

6.6 Quantum Yield Measurement for the Radical Acylation reactions

The photon flux of the spectrophotometer was determined by ferrioxalate actinometry. A ferrioxalate actinometer solution was prepared according to literature procedures[6]. The ferrioxalate actinometer solution measures the decomposition of ferric ions to ferrous ions, which are complexed by 1,10-phenanthroline and monitored by UV/Vis absorbance at 510 nm. The moles of iron-phenanthroline complex formed are related to moles of photons absorbed. The solutions were prepared and stored in a dark laboratory (red light):

1. Potassium ferrioxalate solution: 59 mg of potassium ferrioxalate and 27.8 μL of sulfuric acid (96%) were added to a 10 mL volumetric flask, and filled to the mark with water (HPLC grade).

2. Phenantroline solution: 0.2% by weight of 1,10-phenanthroline in water (20 mg in 10 mL volumetric flask).

3. Buffer solution: to a 10 mL volumetric flask, 494 mg of NaOAc and 100 μL of sulfuric acid (96%) were added and filled to the mark with water (HPLC grade).

To determine the photon flux of the spectrophotometer, 1.0 mL of the ferrioxalate solution was placed in a flame-dried Schlenk tube and irradiated for 30.0 seconds at $\lambda = 450$ nm. The

ferrioxalate solution was irradiated with five 30 W Blue LEDs which same light intensity as model reaction without stirring. After irradiation, the actinometer solution was removed and placed in a 10 mL volumetric flask containing 0.18 mL of 1,10-phenanthroline solution and 1 mL of buffer solution. This flask was filled to the mark with water (HPLC grade). The flask was then allowed to rest for 1 h to allow the ferrous ions to completely coordinate to the phenanthroline. The absorbance of the solution was measured at 510 nm. A non-irradiated sample was also prepared and the absorbance at 510 nm measured. Conversion was calculated using eq 1 .

$$moles\ Fe^{2+} = \frac{V1 \cdot V3 \cdot \Delta A(510nm)}{10^3 \cdot V^2 \cdot l \cdot \varepsilon(510nm)} \quad \text{eq 1}$$

where V1 is the irradiated volume (1 mL), V2 is the aliquot of the irradiated solution taken for the determination of the ferrous ions (1 mL), V3 is the final volume after complexation with phenanthroline (10 mL), l is the optical path-length of the irradiation cell (1 cm), ΔA(510 nm) the optical difference in absorbance between the irradiated solution and the one stored in the dark, ε(510 nm) is that of the complex $Fe(phen)_3^{2+}$ (11100 L mol^{-1}.cm^{-1}).

$$q^0_{n,p} = \frac{moles\ Fe^{2+}}{\Phi \cdot t \cdot [1-10^{-A(\lambda)}]} \quad \text{eq 2}$$

Where Φ is the quantum yield for the ferrioxalate actinometer (0.9 for a 0.011 M solution at λ = 450 nm)[7], t is the time (30.0 s), and f is the fraction of light absorbed at λ = 450 nm. The photon flux ($q^0_{n,p}$) was calculated (average of two experiments) to be 3.79 $\times 10^{-8}$ einstein s^{-1}.
Sample calculation:

$$moles\ Fe^{2+} = \frac{1mL \cdot 10mL \cdot 0.501}{10^3 \cdot 1mL \cdot 1cm \cdot 11100L \cdot mol^{-1} \cdot cm^{-1}} = 4.51 \times 10^{-7}\ mol$$

$$q^0_{n,p} = \frac{4.51 \times 10^{-7}}{0.9 \cdot 30s \cdot (1-10^{-0.252})} = 3.79 \times 10^{-8}\ einstein\ s^{-1}$$

The measurements for the reaction in this study were performed as follows: To an oven-dried 10 mL Schlenk tube were added **2a** (0.1 mmol), **3a** (0.25 mmol), NHC **N1** (0.02 mmol), 4CzIPN (0.002 mmol), and K_2CO_3 (0.2 mmol), after which the tube was evacuated and back-filled with argon three times. Subsequently, under the protection of Ar, anhydrous MeCN (1.0 mL) were added *via* syringe. The mixture was then stirred rapidly and irradiated with Blue LEDs (30 W × 5, λ_{max} = 450 nm, approximately 2 cm away from the light source) at room temperature. The product **4a** was determined calculated by 1H NMR with dibromomethane as internal standard. The moles of product per unit of time are related to the number of photons absorbed. The moles of **4a** formed (x) are plotted as a function of time (t). The slope of this line was correlated to the moles of incident photons by unit of time by the use of the following eq 3.

$$\Phi(\lambda) = \frac{dx/dt}{q^{0}{}_{n.p}\left[1-10^{-A(\lambda)}\right]} \quad \text{eq 3}$$

According to eq 3, the moles of **5** formed (x) were plotted as a function of time (t): Φ was the quantum yield to be determined and *A*(450 nm) was the absorption of the reaction under study. *A*(450 nm) was measured using a SHIMADZU UV-2450 UV/Vis spectrometer in 1 cm path quartz to carry out the measurements, obtaining an absorbance of 1.437. The quantum yield (Φ) of the reation of **2a** and **3a** was calculated to be 0.685.

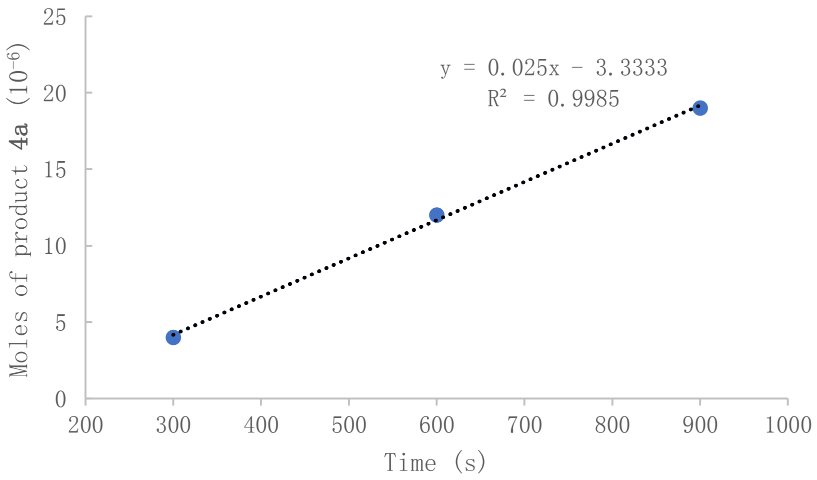

6.7 UV-Vis absorption

The UV-Vis absorption spectrum of 4CzIPN, **2a**, **3a**, acyl azolium **56** and their combinations were measured respectively by SHIMADZU UV2450 UV/Vis spectrometer. Concentration of $2*10^{-5}$ M in MeCN was chose in all cases. As shown in the following figure, the absorption spectrum of 4CzIPN revealed a significant absorption of visible light. By contrast, other compounds have little absorptions at the visible light region.

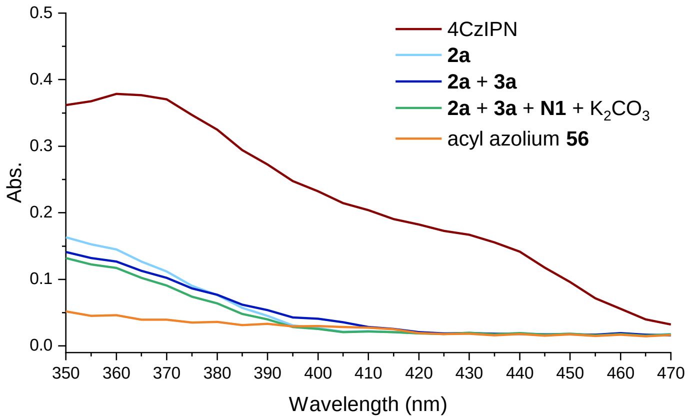

6.8 Luminescence quenching experiments

The fluoresence intensity was measured by HITACHI F-7000 spectrofluorometer. The sample was prepared by adding 1 mL of 10^{-3} M solution of 4CzIPN and degassed MeCN solution of **2a**, K_2CO_3 or acyl azolium **56** in different concentrations (1 mM, 2 mM, 4 mM, 6 mM, or 8 mM respectively), and mixed. All 4CzIPN solutions were excited at 350 nm and the emission intensity was collected at 450-650 nm.

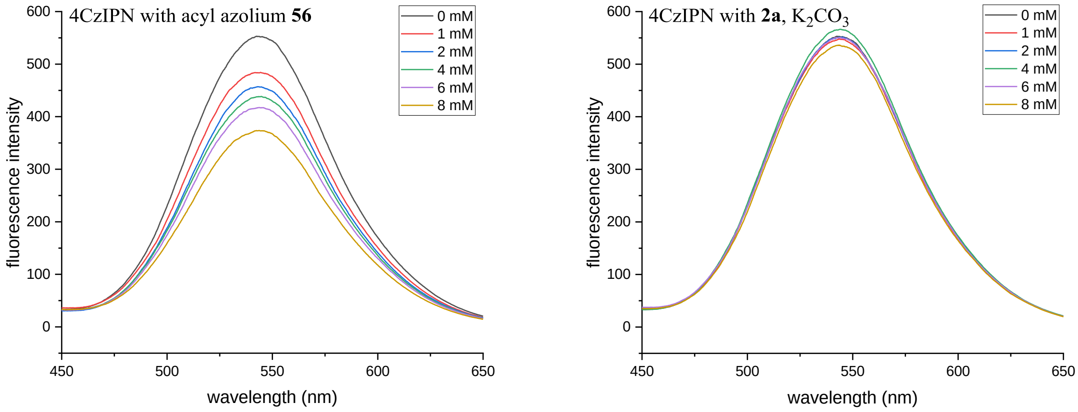

Stern-Volmer

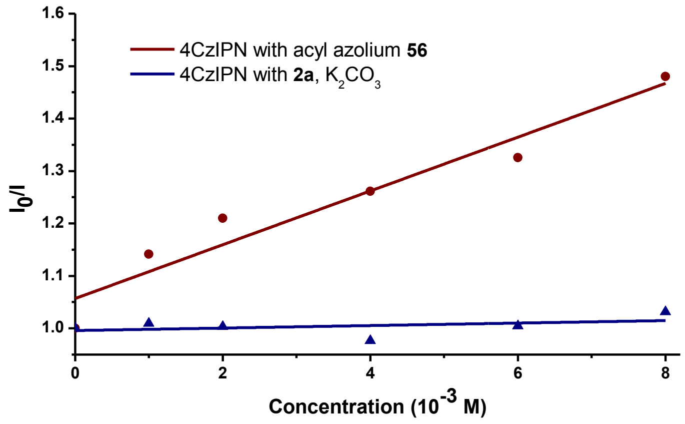

6.9 The attempt of energy transfer pathway

A control experiment between **2a** and **3a** was conducted under 390 nm LED irradiation in the absence of a photocatalyst. The desired product **4a** was not formed under these conditions. This outcome suggests that an energy-transfer pathway is unlikely to be operative in this process.

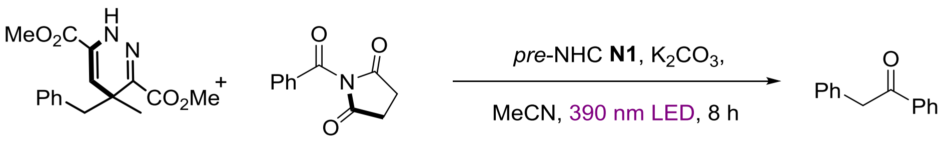

2a **3a** **4a** < 1%

7. References and Notes

[1] a) E. Krell, *Handbook of Laboratory Distillation*, Elseriver Publishing Company: Amsterdam-London-New York. **1963**. b) M. J. Rosengart, *The Technique of Distillation and Rectification in the Laboratory*, VEB Verlag Technik, Berlin, **1954**. c) F. Stage, *Angew. Chem.* **1947**, *19*, 175−183.

[2] a)G. S. Lee, J. Won, S. Choi, M.-H. Baik, S. H. Hong, *Angew. Chem., Int. Ed.* **2020**, *59*, 16933−16942; b) S. Shi, M. Szostak, *Synthesis* **2017**, *49*, 3602−3608; c) Y. Osumi, C. Liu, M. Szostak, *Org. Biomol. Chem.* **2017**, *15*, 8867−8871.

[3] a) A. V. Bay, K. P. Fitzpatrick, R. C. Betori, K. A. Scheidt, *Angew. Chem., Int. Ed.* **2020**, *59*, 9143−9148; b) A. Lee, K. A. Scheidt, *Chem. Commun.* **2015**, *51*, 3407−3410.

[4] A. Mavroskoufis, K. Rajes, P. Golz, A. Agrawal, V. Ruß, J. P. Götze, M. N. Hopkinson, *Angew. Chem., Int. Ed.* **2020**, *59*, 3190−3194.

[5] D. L. Boger, J. S. Panek, M. Patel, *Org. Synth.* **1992**, *70*, 79.

[6] A. Bahamonde, P. Melchiorre, *J. Am. Chem. Soc.* **2016**, *138*, 8019−8030.

[7] M. A. Cismesia, T. P. Yoon, *Chem. Sci.* **2015**, *6*, 5426−5434.

8. Copies of NMR Spectra

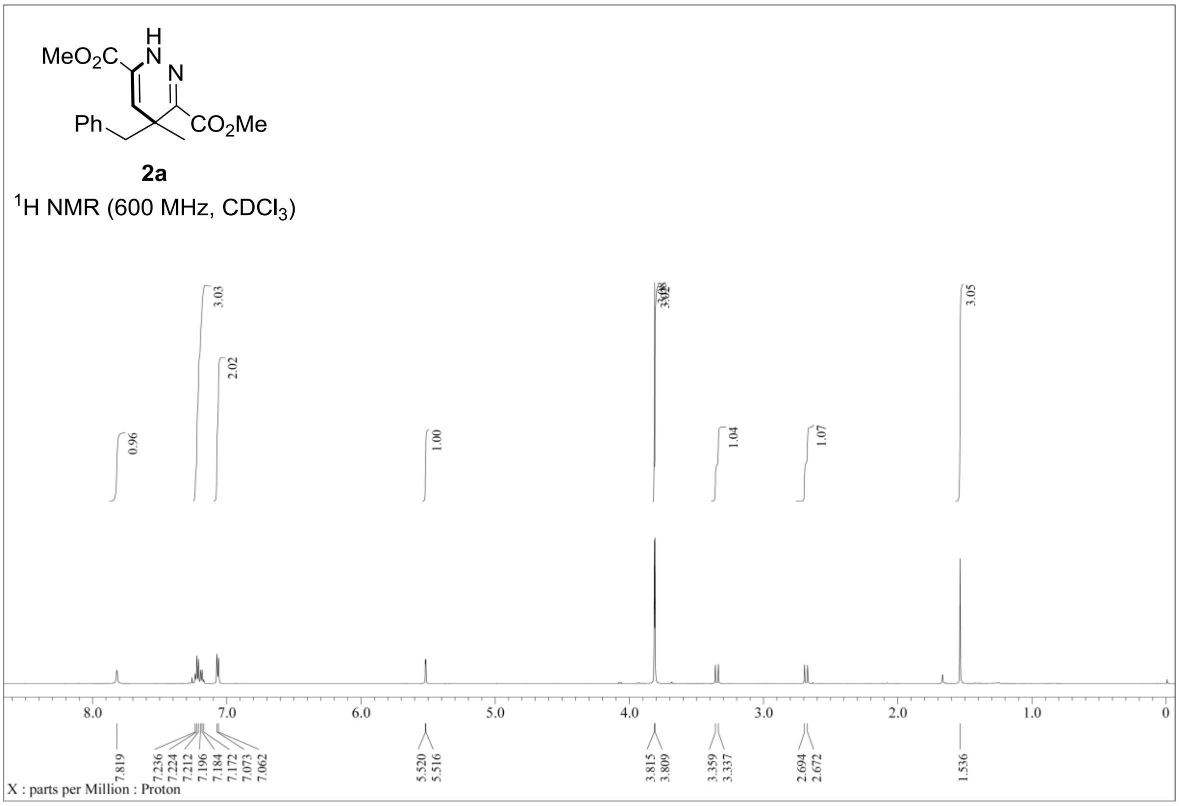

2a

^{1}H NMR (600 MHz, $CDCl_3$)

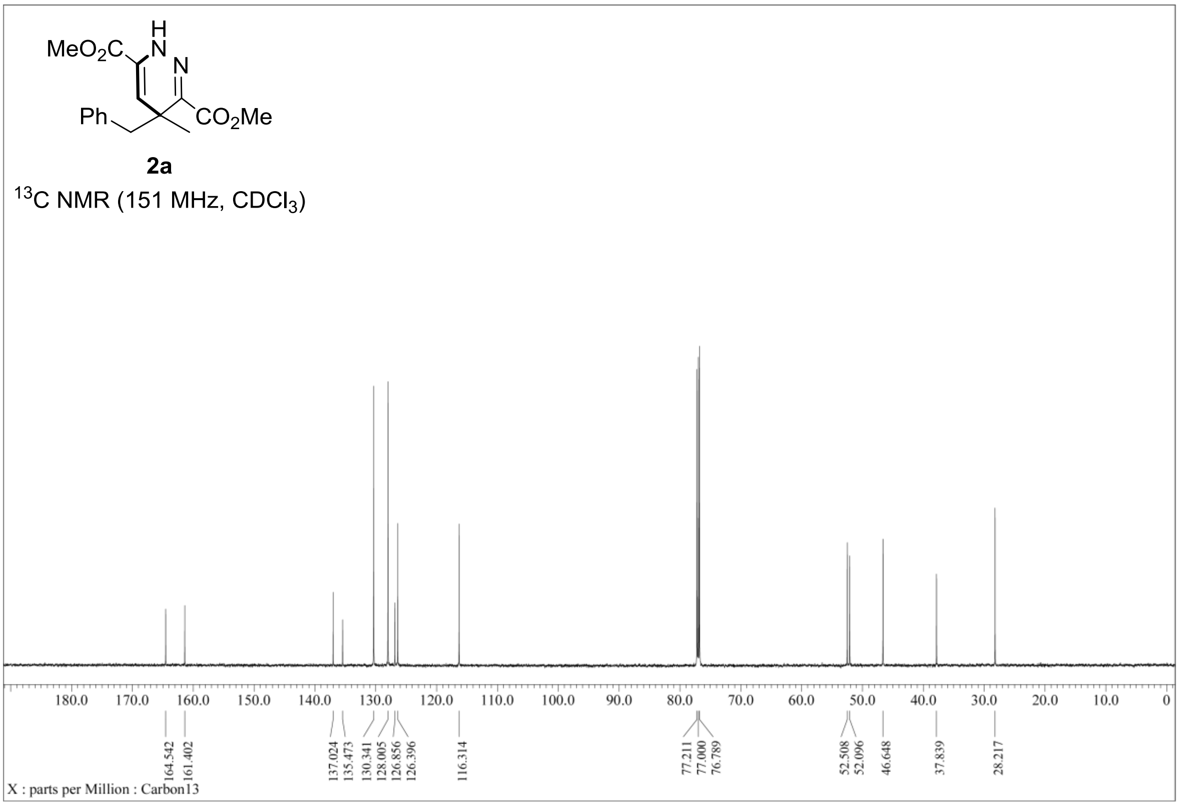

2a

^{13}C NMR (151 MHz, $CDCl_3$)

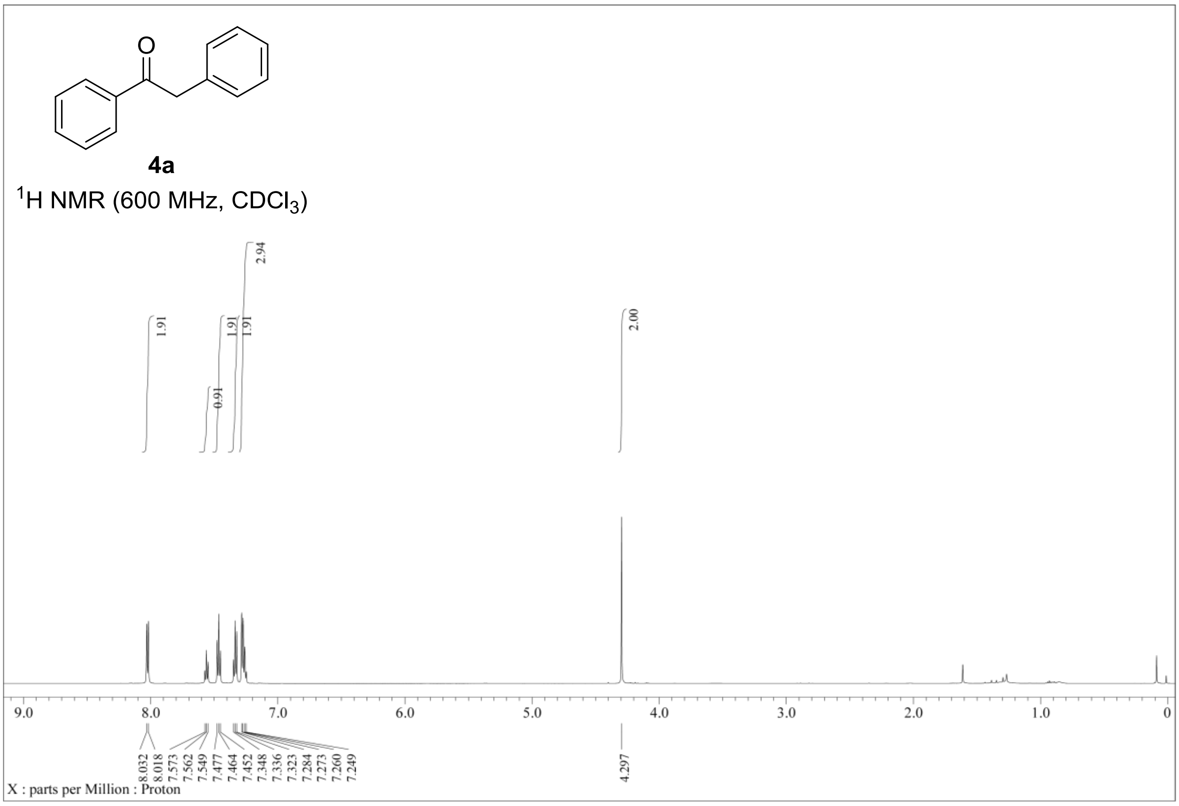
4a
^{1}H NMR (600 MHz, $CDCl_3$)
1.91
0.91
1.91
1.91
2.94
2.00
9.0
8.0
7.0
6.0
5.0
4.0
3.0
2.0
1.0
0
8.032
8.018
7.573
7.562
7.549
7.477
7.464
7.452
7.348
7.336
7.323
7.284
7.273
7.260
7.249
4.297
X : parts per Million : Proton

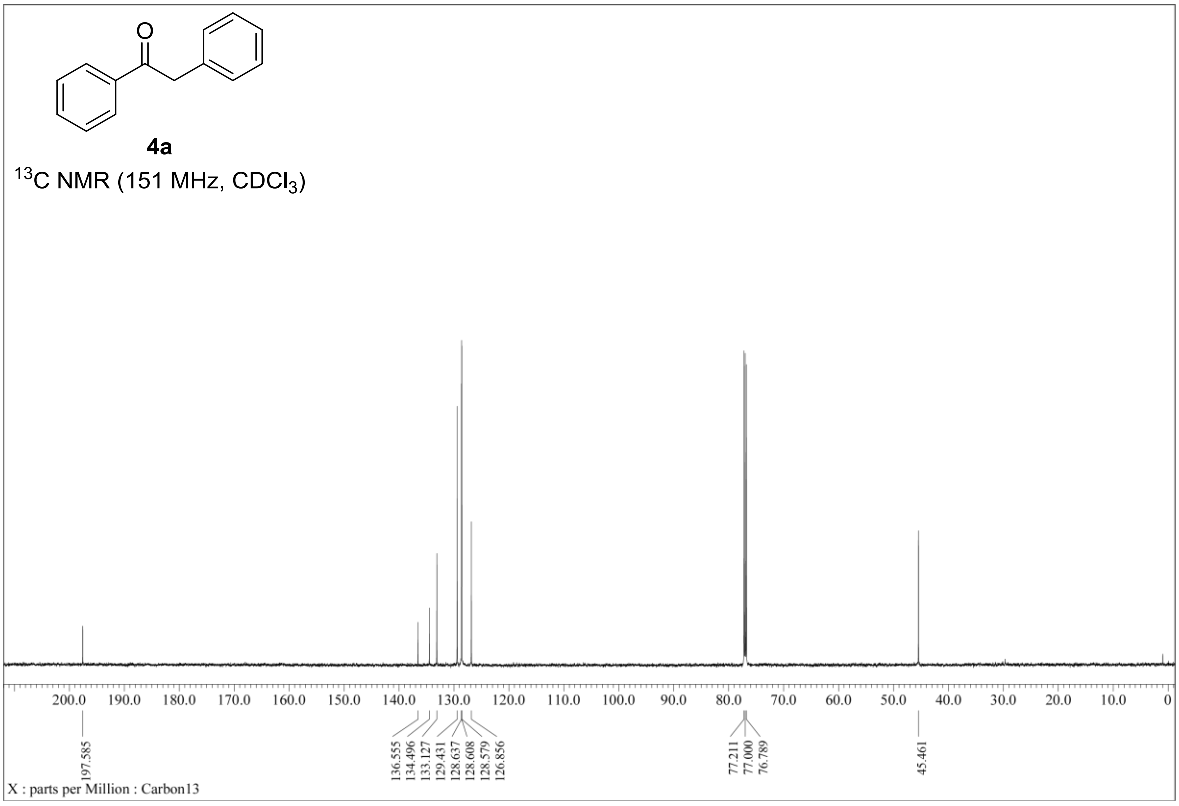
4a
^{13}C NMR (151 MHz, $CDCl_3$)
200.0
190.0
180.0
170.0
160.0
150.0
140.0
130.0
120.0
110.0
100.0
90.0
80.0
70.0
60.0
50.0
40.0
30.0
20.0
10.0
0
197.585
136.555
134.496
133.127
129.431
128.637
128.608
128.579
126.856
77.211
77.000
76.789
45.461
X : parts per Million : Carbon13

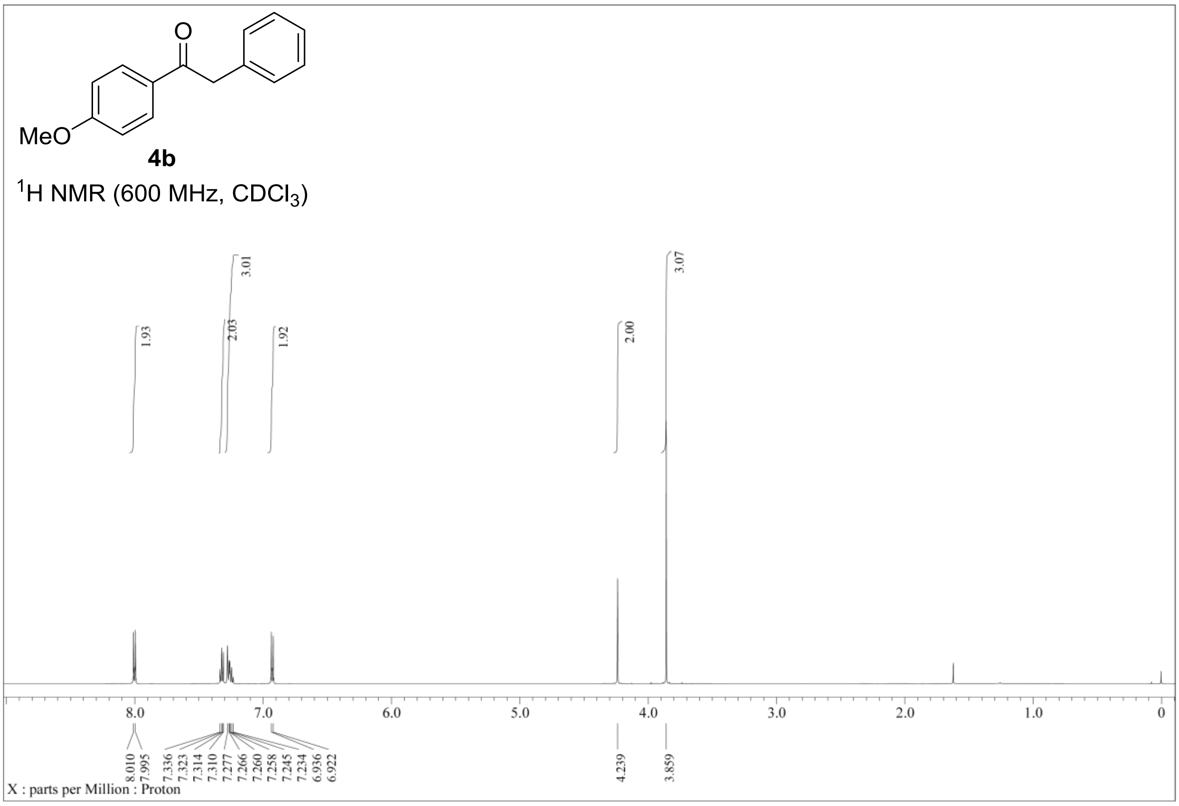
O
MeO
4b
1H NMR (600 MHz, CDCl3)
1.93
3.01
2.03
1.92
2.00
3.07
8.0
7.0
6.0
5.0
4.0
3.0
2.0
1.0
0
8.010
7.995
7.336
7.323
7.314
7.310
7.277
7.266
7.260
7.258
7.245
7.234
6.936
6.922
4.239
3.859
X : parts per Million : Proton

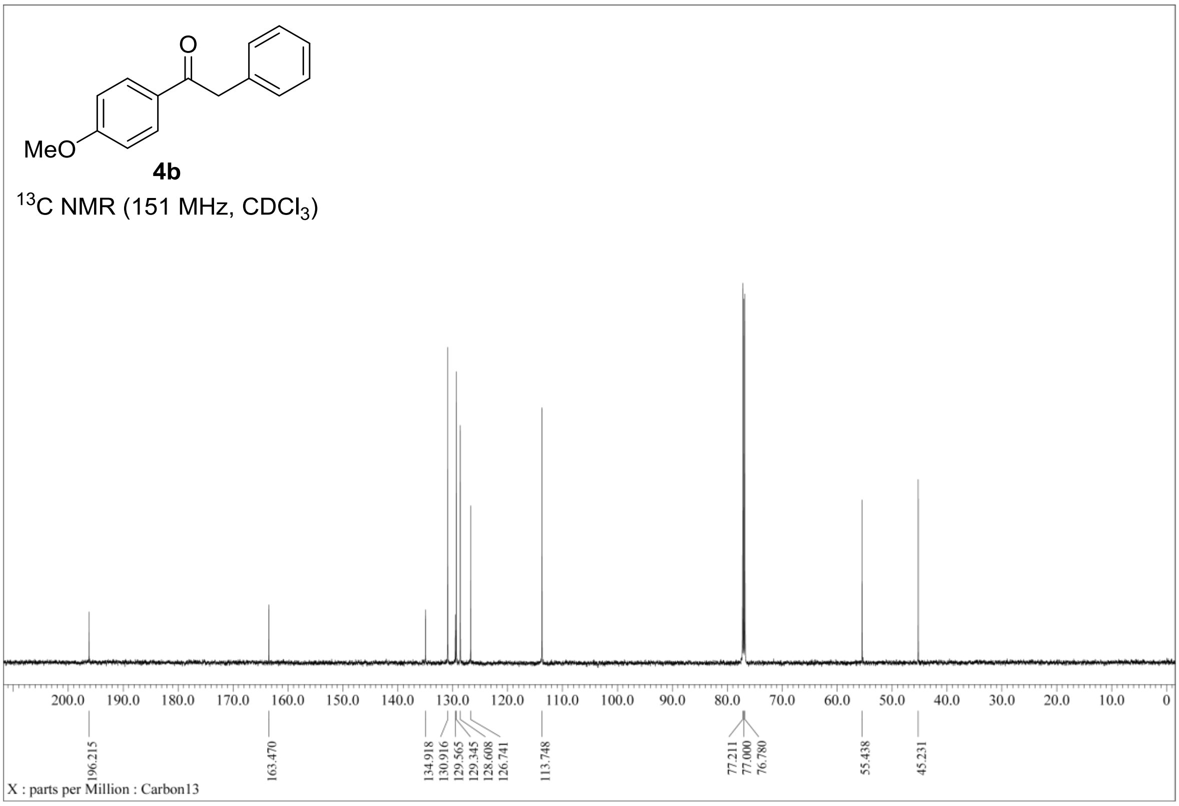
O
MeO
4b
13C NMR (151 MHz, CDCl3)
200.0
190.0
180.0
170.0
160.0
150.0
140.0
130.0
120.0
110.0
100.0
90.0
80.0
70.0
60.0
50.0
40.0
30.0
20.0
10.0
0
196.215
163.470
134.918
130.916
129.565
129.345
128.608
126.741
113.748
77.211
77.000
76.780
55.438
45.231
X : parts per Million : Carbon13

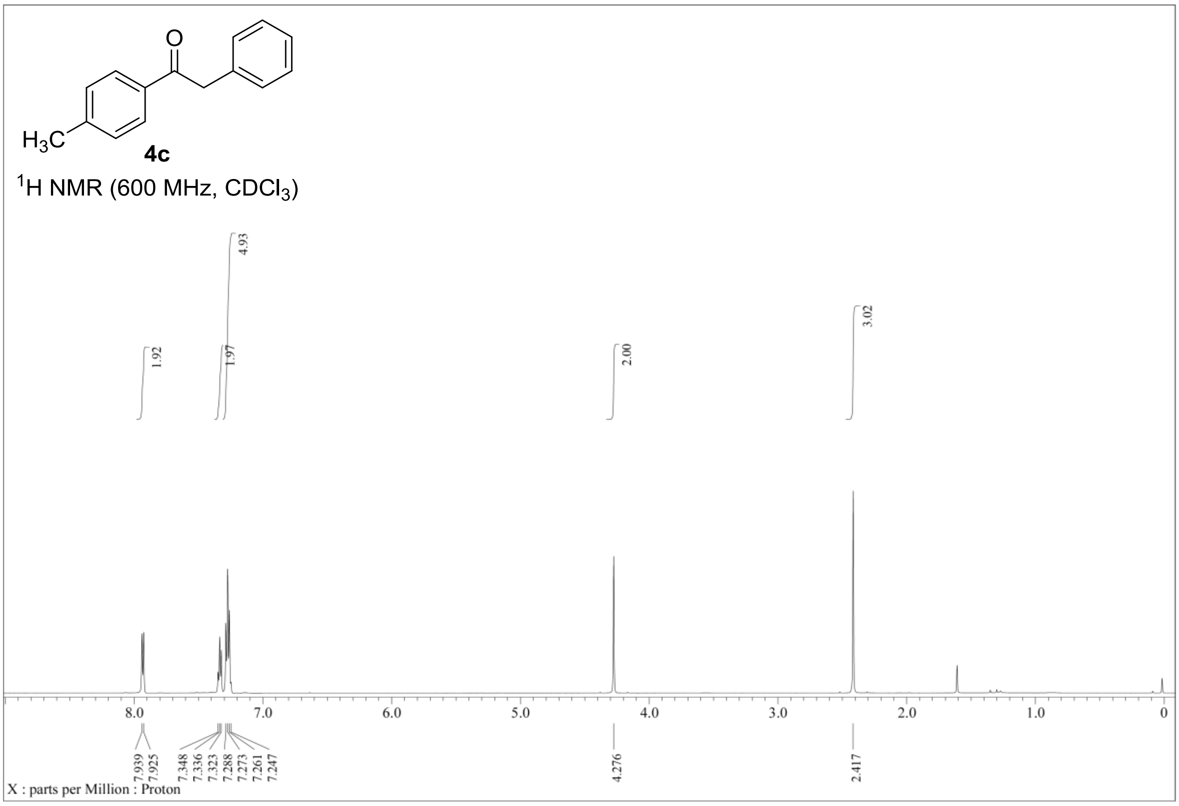
O
H3C
4c
1H NMR (600 MHz, CDCl3)
1.92
1.97
4.93
2.00
3.02
8.0
7.0
6.0
5.0
4.0
3.0
2.0
1.0
0
7.939
7.925
7.348
7.336
7.323
7.288
7.273
7.261
7.247
4.276
2.417
X : parts per Million : Proton

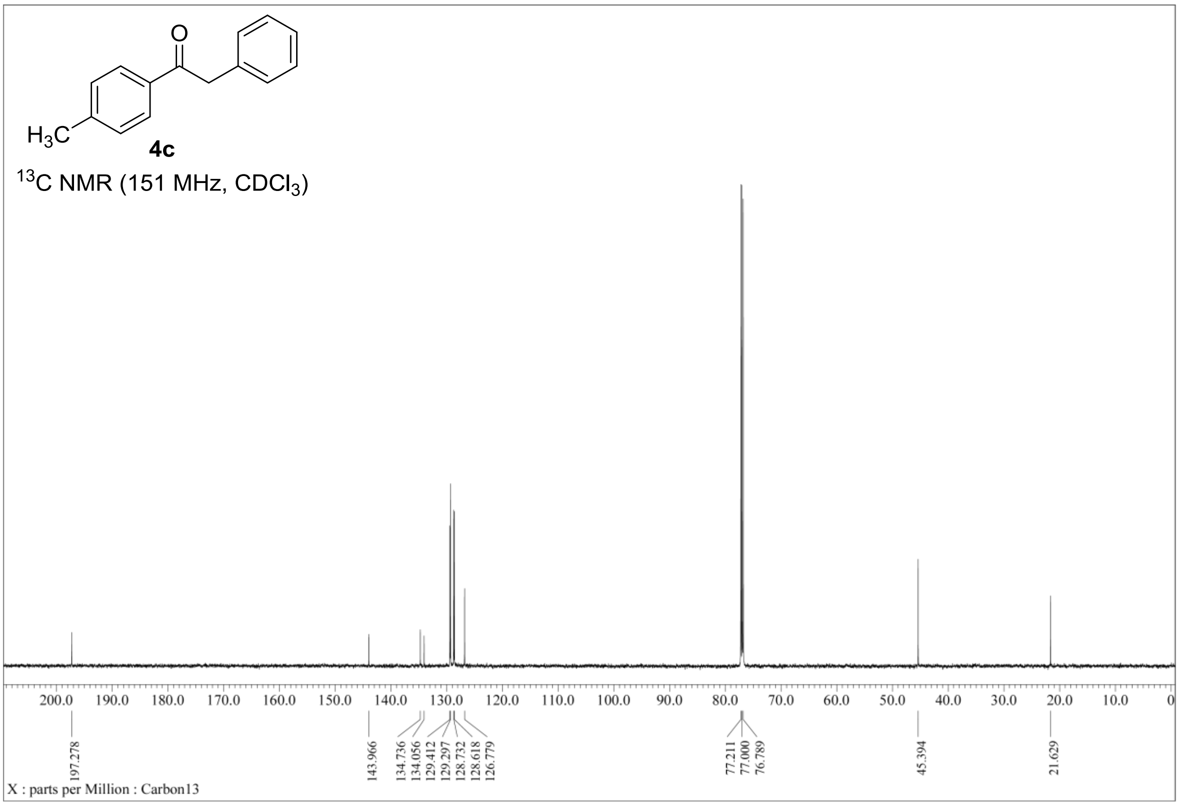
O
H3C
4c
13C NMR (151 MHz, CDCl3)
200.0
190.0
180.0
170.0
160.0
150.0
140.0
130.0
120.0
110.0
100.0
90.0
80.0
70.0
60.0
50.0
40.0
30.0
20.0
10.0
0
197.278
143.966
134.736
134.056
129.412
129.297
128.732
128.618
126.779
77.211
77.000
76.789
45.394
21.629
X : parts per Million : Carbon13

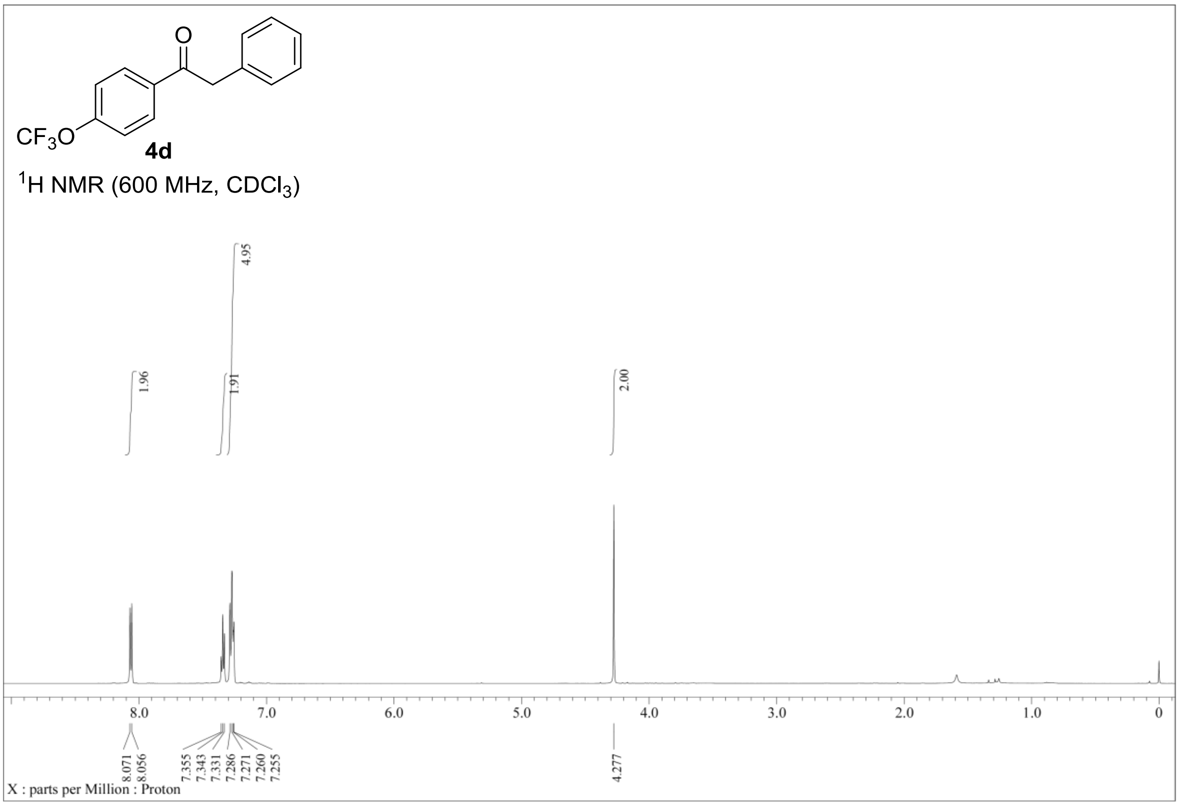

O
CF_3O
4d
^{1}H NMR (600 MHz, $CDCl_3$)
4.95
1.96
1.91
2.00
8.0
7.0
6.0
5.0
4.0
3.0
2.0
1.0
0
8.071
8.056
7.355
7.343
7.331
7.286
7.271
7.260
7.255
4.277
X : parts per Million : Proton

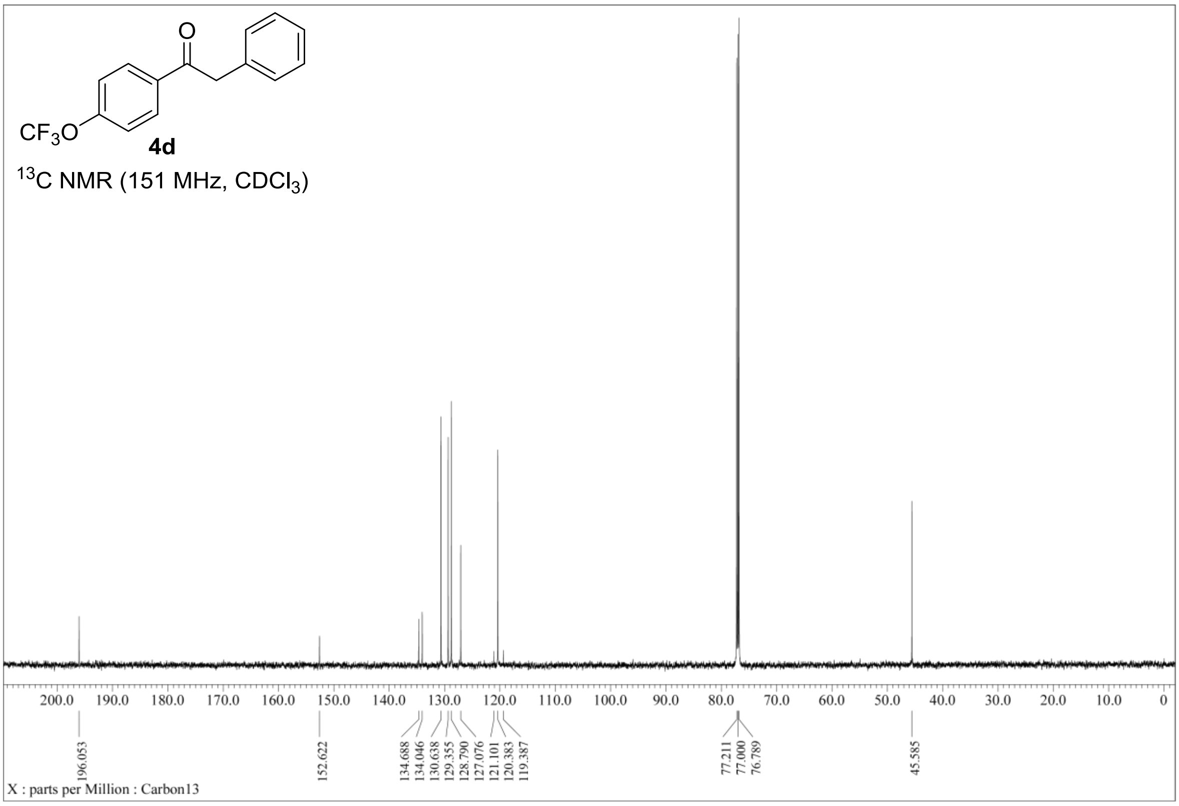

O
CF_3O
4d
^{13}C NMR (151 MHz, $CDCl_3$)
200.0
190.0
180.0
170.0
160.0
150.0
140.0
130.0
120.0
110.0
100.0
90.0
80.0
70.0
60.0
50.0
40.0
30.0
20.0
10.0
0
196.053
152.622
134.688
134.046
130.638
129.355
128.790
127.076
121.101
120.383
119.387
77.211
77.000
76.789
45.585
X : parts per Million : Carbon13

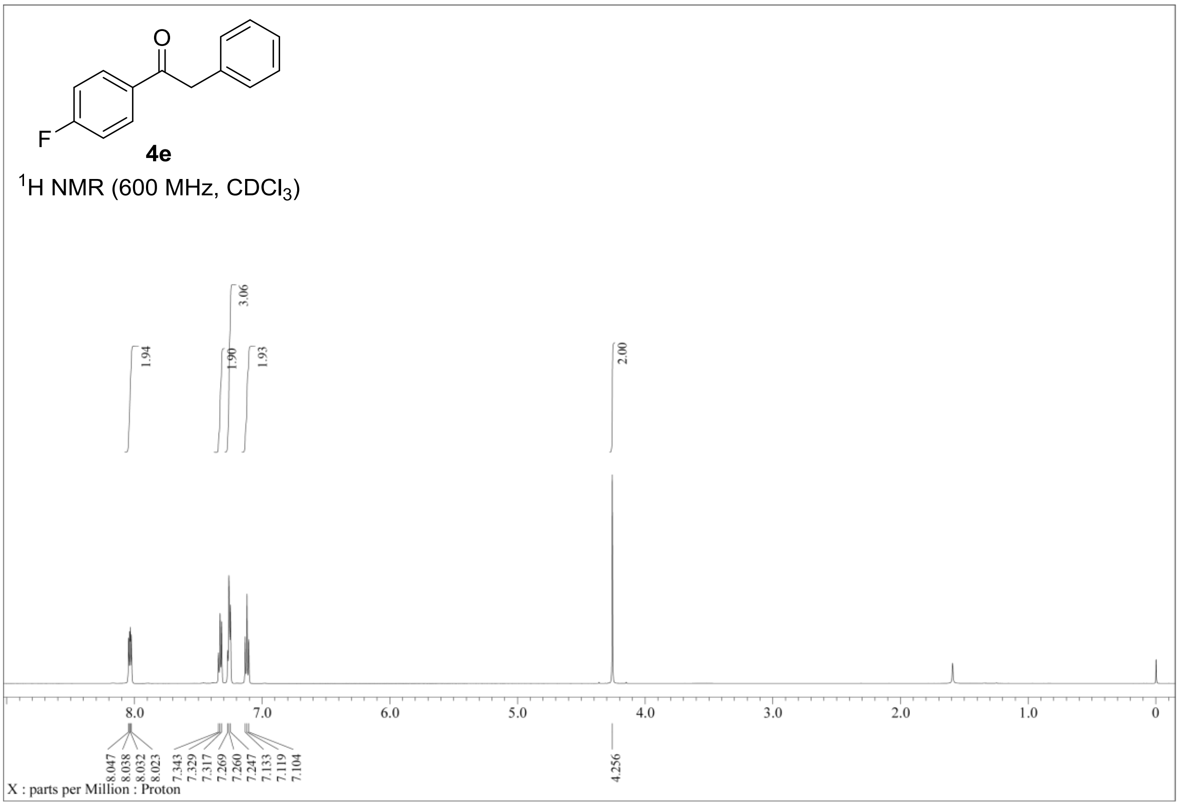
O
F
4e
¹H NMR (600 MHz, CDCl3)
1.94
3.06
1.90
1.93
2.00
8.0
7.0
6.0
5.0
4.0
3.0
2.0
1.0
0
8.047
8.038
8.032
8.023
7.343
7.329
7.317
7.269
7.260
7.247
7.133
7.119
7.104
4.256
X : parts per Million : Proton

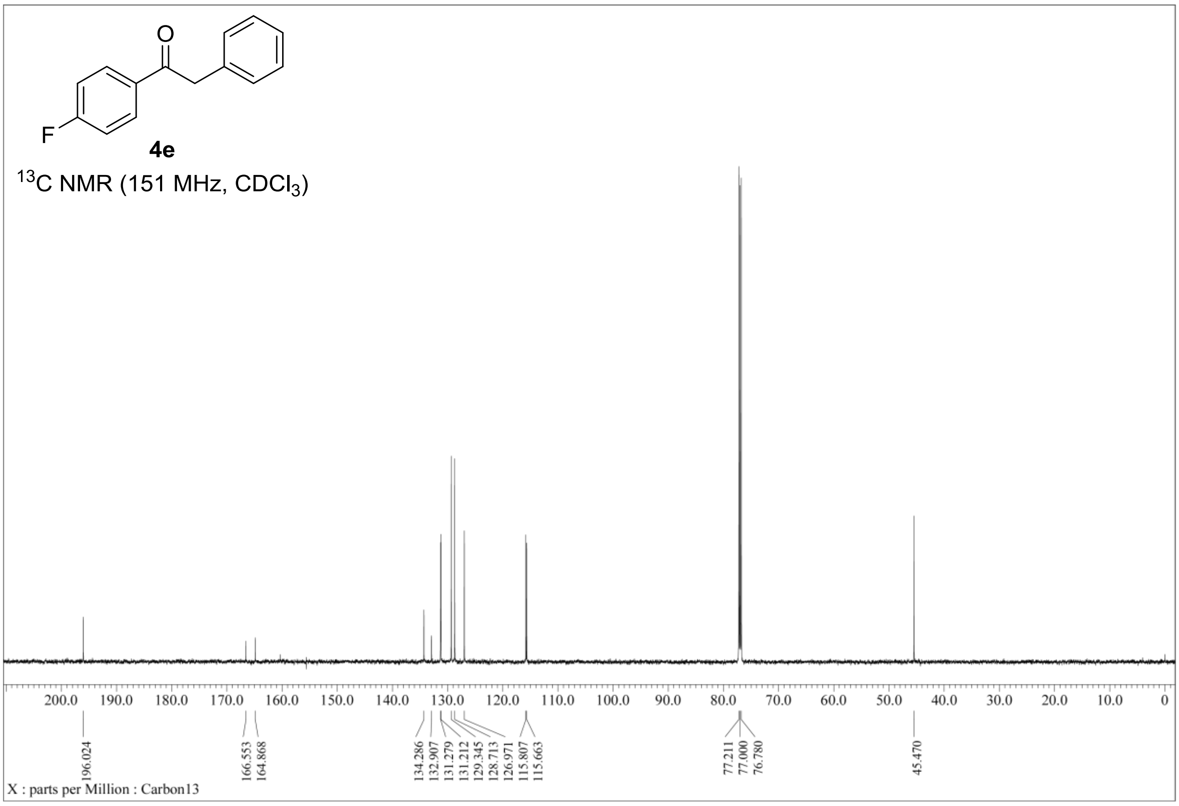
O
F
4e
¹³C NMR (151 MHz, CDCl3)
200.0
190.0
180.0
170.0
160.0
150.0
140.0
130.0
120.0
110.0
100.0
90.0
80.0
70.0
60.0
50.0
40.0
30.0
20.0
10.0
0
196.024
166.553
164.868
134.286
132.907
131.279
131.212
129.345
128.713
126.971
115.807
115.663
77.211
77.000
76.780
45.470
X : parts per Million : Carbon13

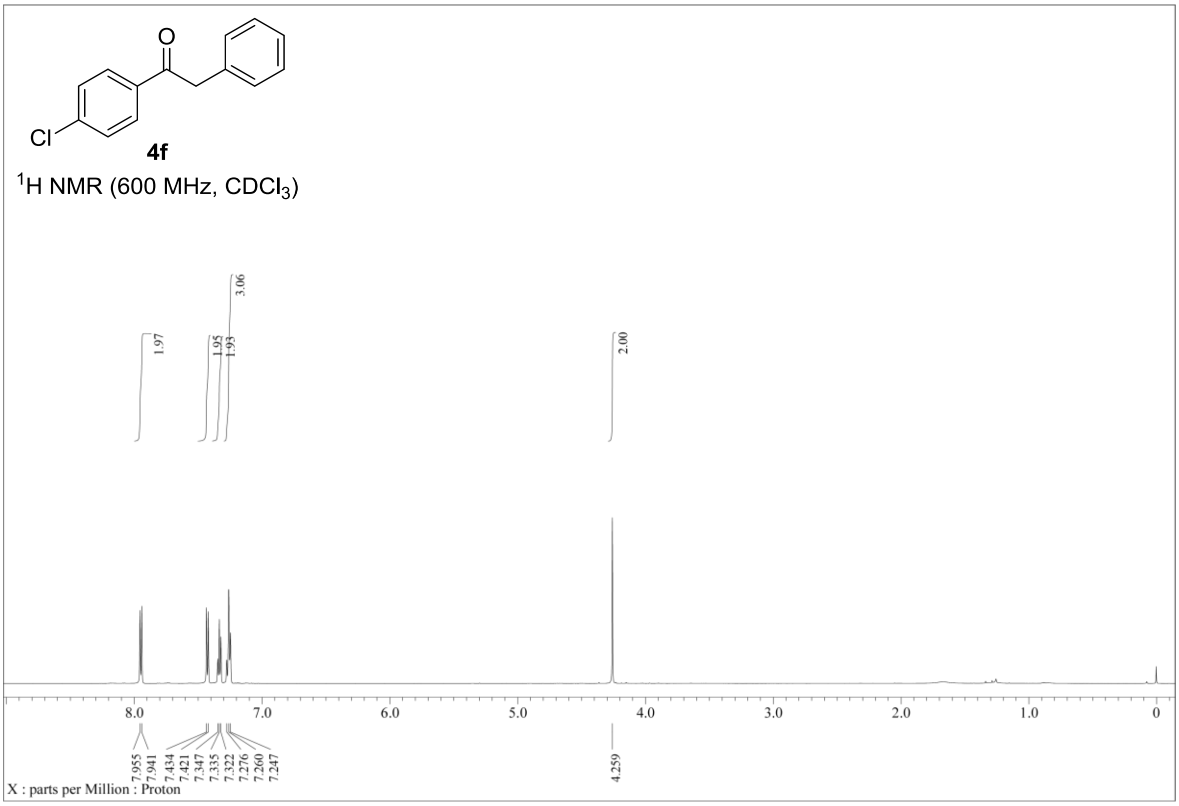

O
Cl
4f
^{1}H NMR (600 MHz, CDCl$_3$)
1.97
1.95
1.93
3.06
2.00
8.0
7.0
6.0
5.0
4.0
3.0
2.0
1.0
0
7.955
7.941
7.434
7.421
7.347
7.335
7.322
7.276
7.260
7.247
4.259
X : parts per Million : Proton

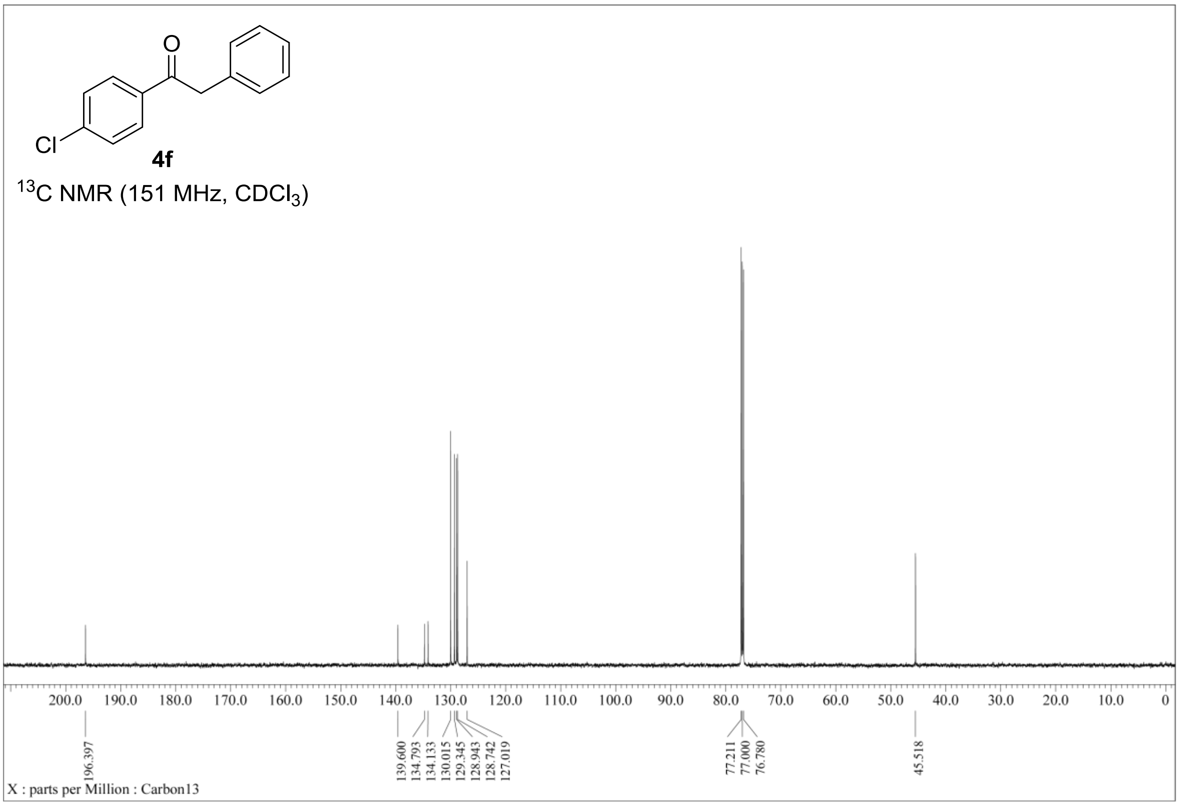

O
Cl
4f
^{13}C NMR (151 MHz, CDCl$_3$)
200.0
190.0
180.0
170.0
160.0
150.0
140.0
130.0
120.0
110.0
100.0
90.0
80.0
70.0
60.0
50.0
40.0
30.0
20.0
10.0
0
196.397
139.600
134.793
134.133
130.015
129.345
128.943
128.742
127.019
77.211
77.000
76.780
45.518
X : parts per Million : Carbon13

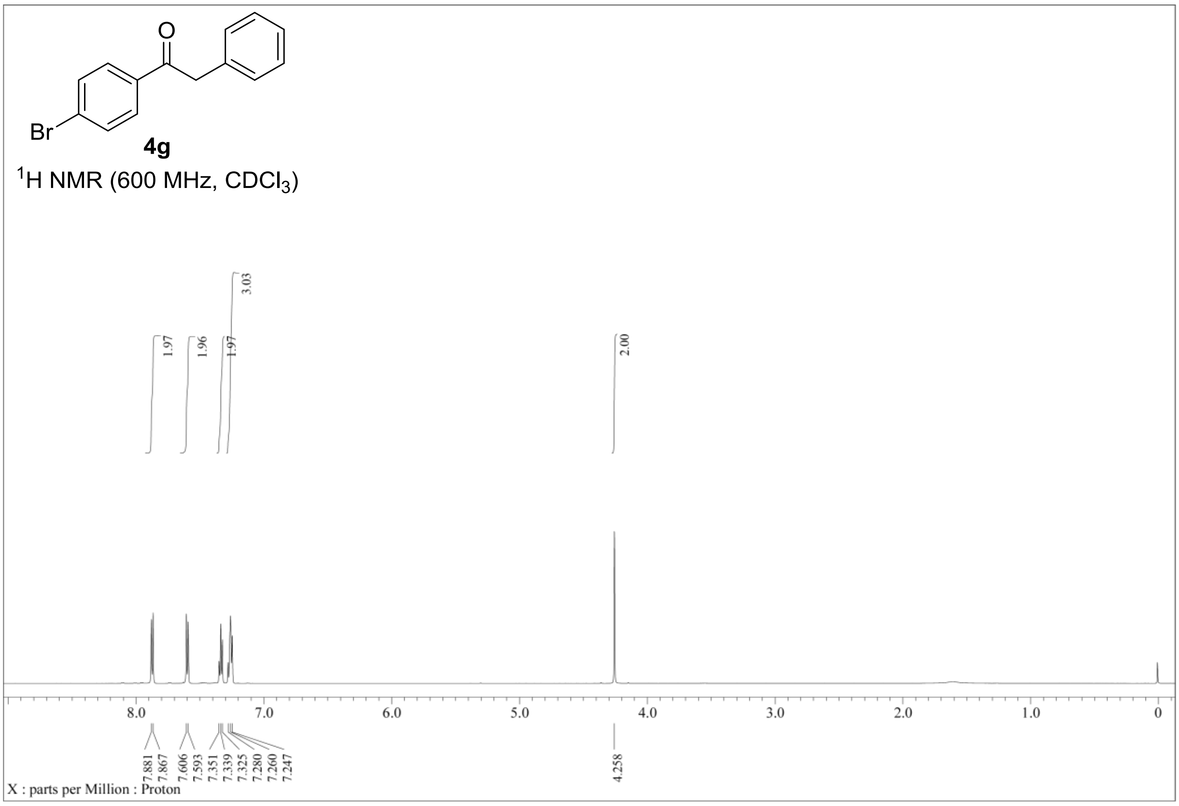

O
Br
4g
1H NMR (600 MHz, CDCl3)
1.97
1.96
1.97
3.03
2.00
8.0
7.0
6.0
5.0
4.0
3.0
2.0
1.0
0
7.881
7.867
7.606
7.593
7.351
7.339
7.325
7.280
7.260
7.247
4.258
X : parts per Million : Proton

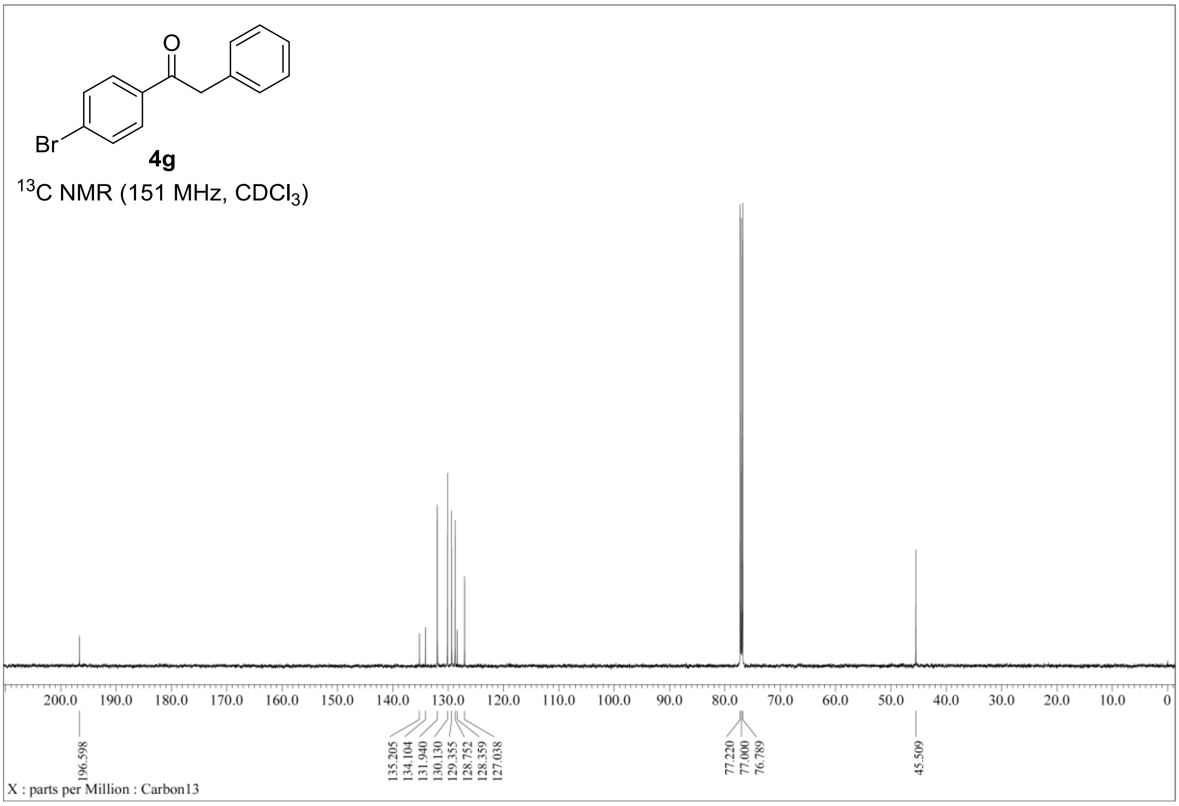

O
Br
4g
13C NMR (151 MHz, CDCl3)
200.0
190.0
180.0
170.0
160.0
150.0
140.0
130.0
120.0
110.0
100.0
90.0
80.0
70.0
60.0
50.0
40.0
30.0
20.0
10.0
0
196.598
135.205
134.104
131.940
130.130
129.355
128.752
128.359
127.038
77.220
77.000
76.789
45.509
X : parts per Million : Carbon13

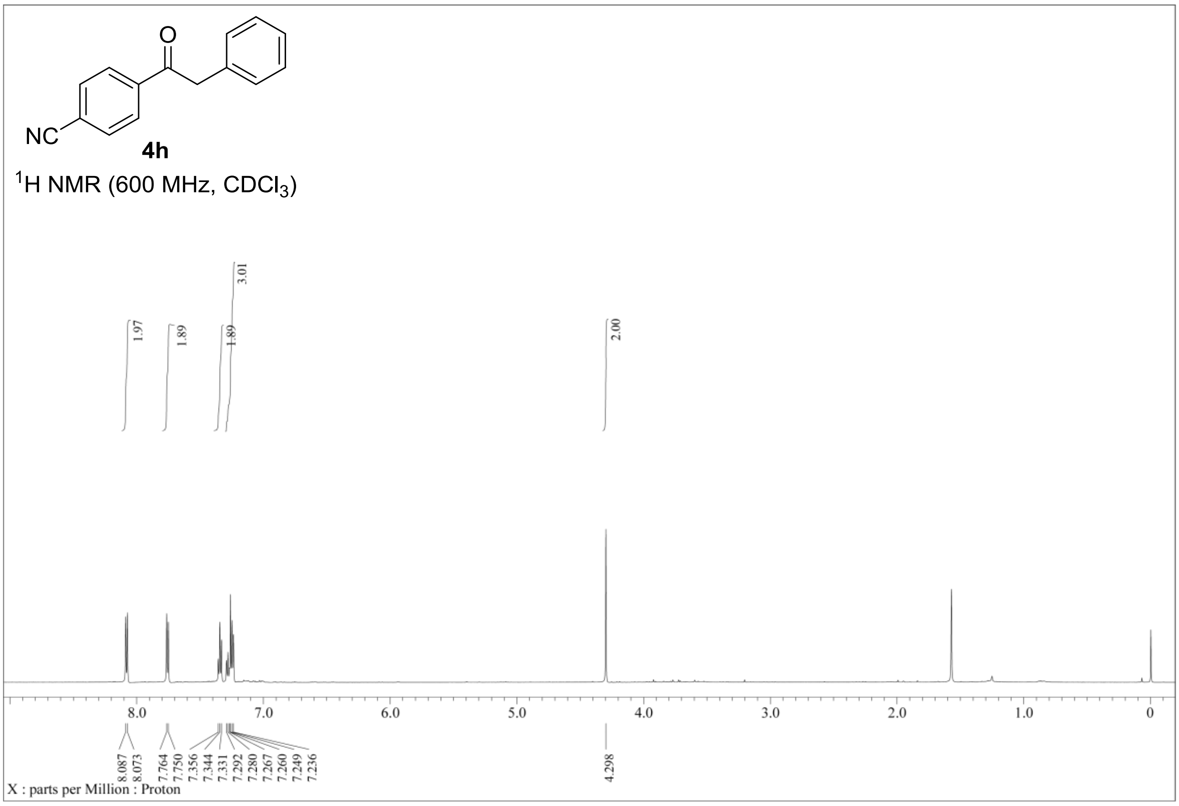
NC
O
4h
1H NMR (600 MHz, CDCl3)
1.97
1.89
1.89
3.01
2.00
8.0
7.0
6.0
5.0
4.0
3.0
2.0
1.0
0
8.087
8.073
7.764
7.750
7.356
7.344
7.331
7.292
7.280
7.267
7.260
7.249
7.236
4.298
X : parts per Million : Proton

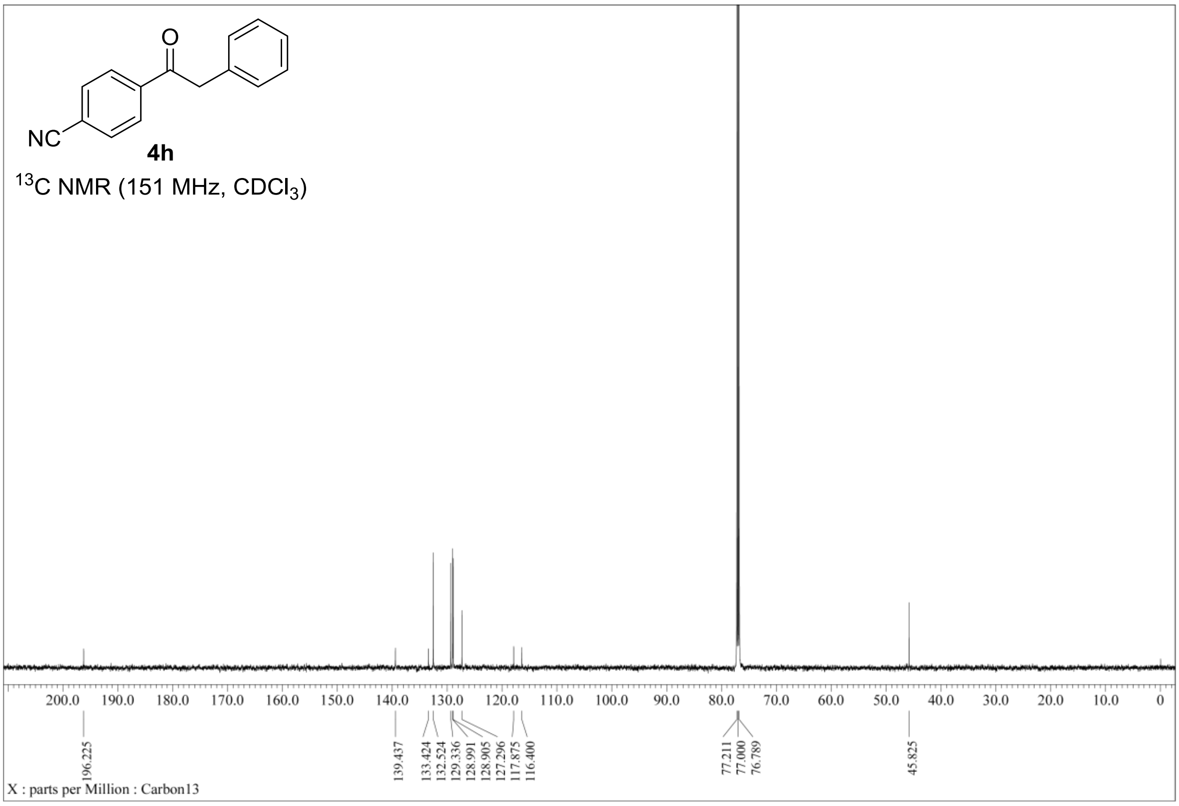
NC
O
4h
13C NMR (151 MHz, CDCl3)
200.0
190.0
180.0
170.0
160.0
150.0
140.0
130.0
120.0
110.0
100.0
90.0
80.0
70.0
60.0
50.0
40.0
30.0
20.0
10.0
0
196.225
139.437
133.424
132.524
129.336
128.991
128.905
127.296
117.875
116.400
77.211
77.000
76.789
45.825
X : parts per Million : Carbon13

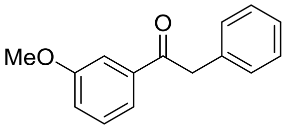

4i

^{1}H NMR (600 MHz, $CDCl_3$)

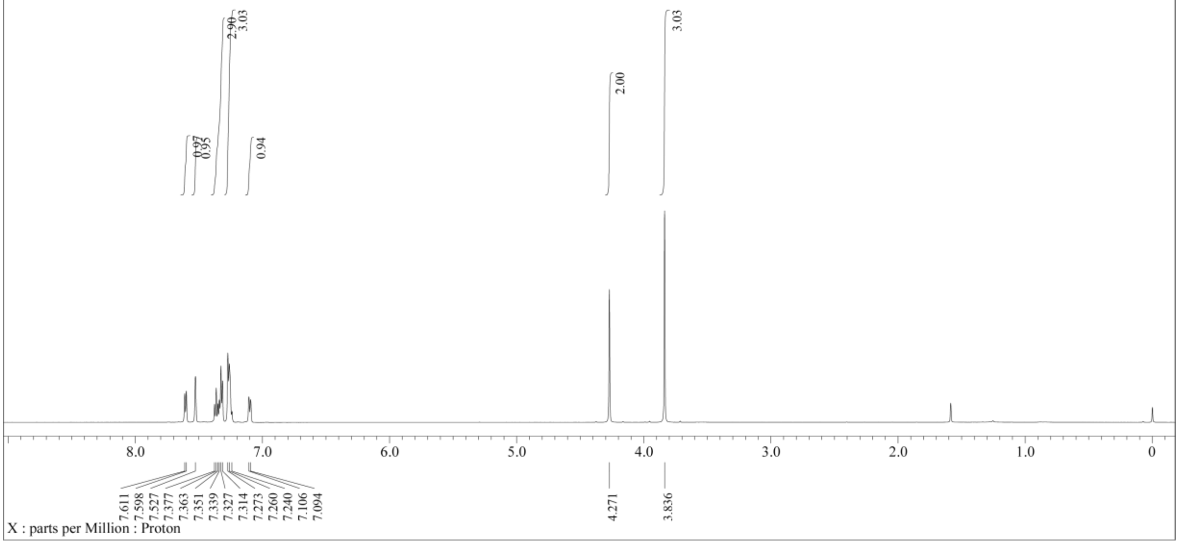

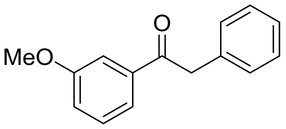

4i

^{13}C NMR (151 MHz, $CDCl_3$)

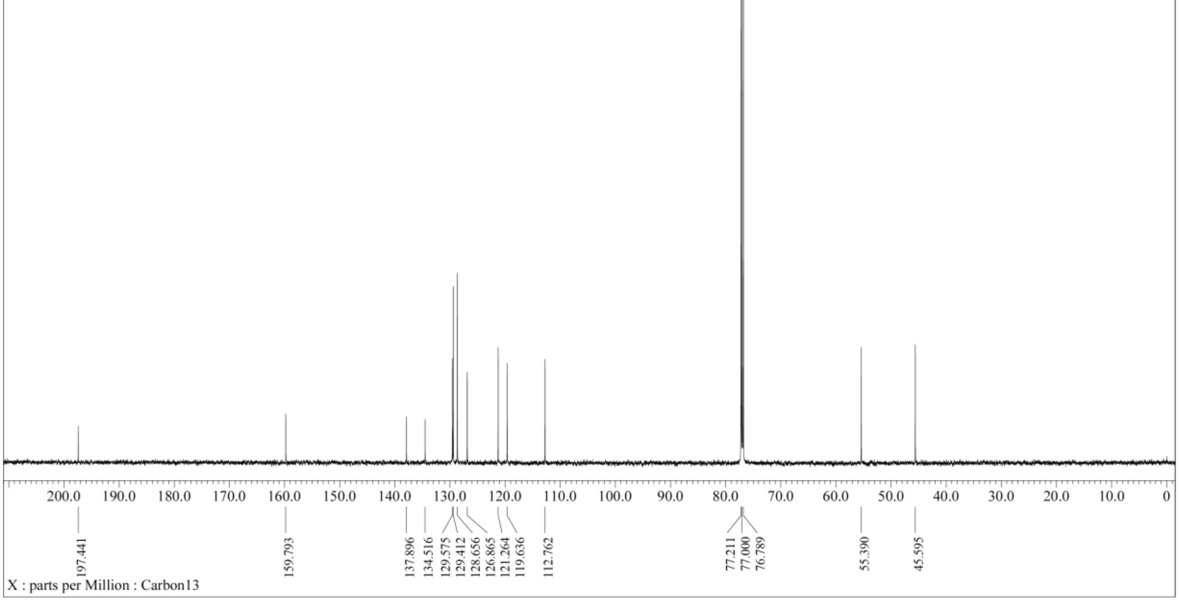

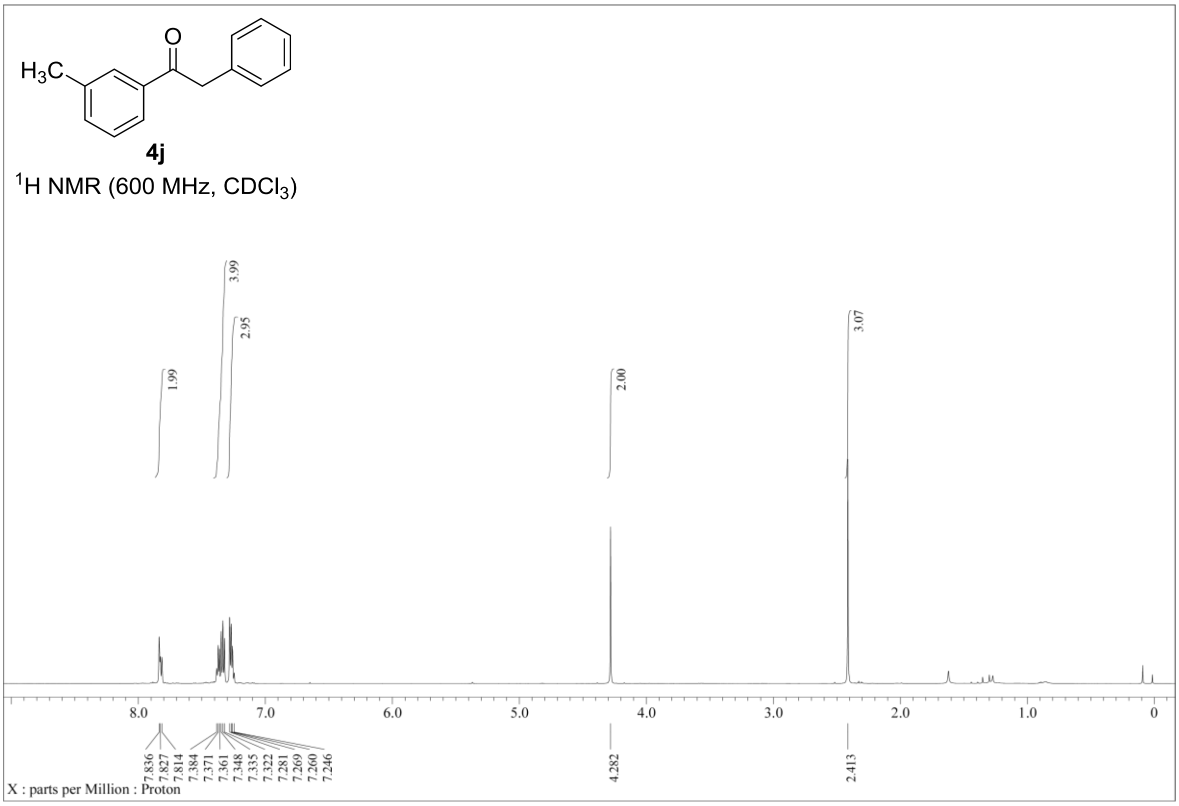
H3C
O
4j
1H NMR (600 MHz, CDCl3)
1.99
3.99
2.95
2.00
3.07
8.0 7.0 6.0 5.0 4.0 3.0 2.0 1.0 0
7.836 7.827 7.814 7.384 7.371 7.361 7.348 7.335 7.322 7.281 7.269 7.260 7.246
4.282
2.413
X : parts per Million : Proton

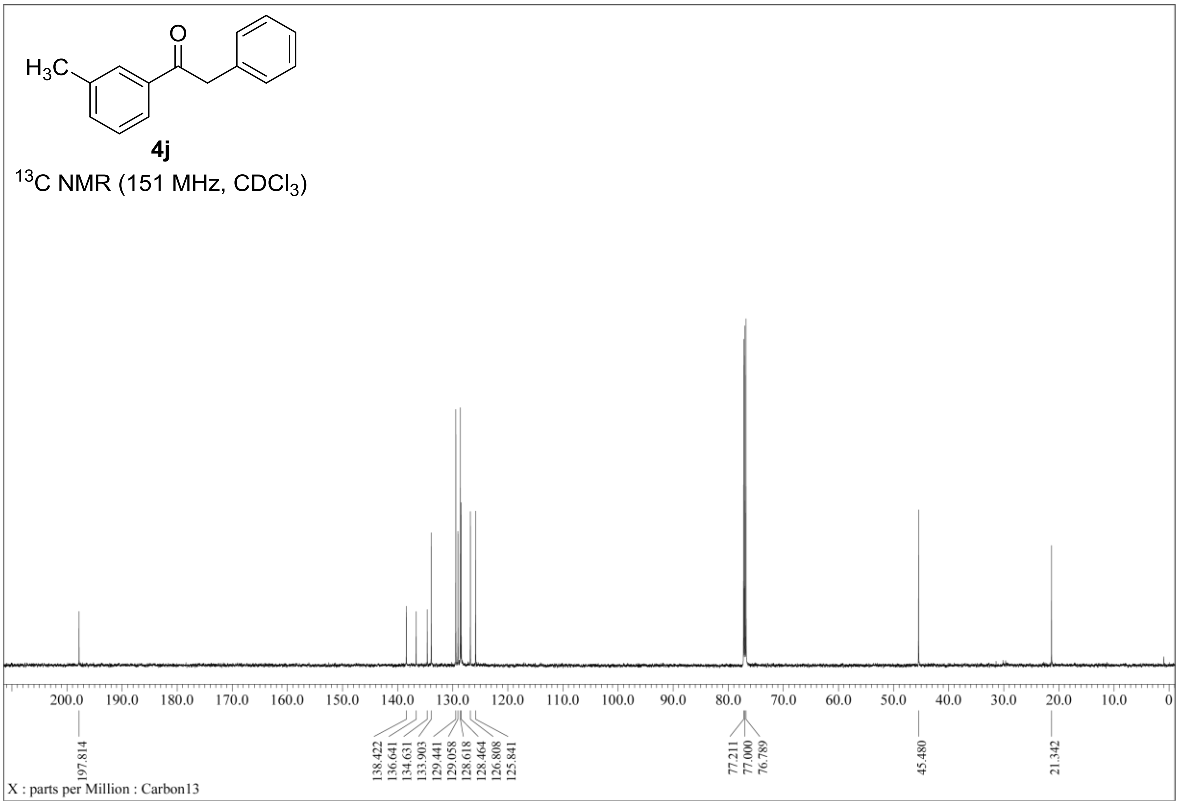
H3C
O
4j
13C NMR (151 MHz, CDCl3)
200.0 190.0 180.0 170.0 160.0 150.0 140.0 130.0 120.0 110.0 100.0 90.0 80.0 70.0 60.0 50.0 40.0 30.0 20.0 10.0 0
197.814
138.422 136.641 134.631 133.903 129.441 129.058 128.618 128.464 126.808 125.841
77.211 77.000 76.789
45.480
21.342
X : parts per Million : Carbon13

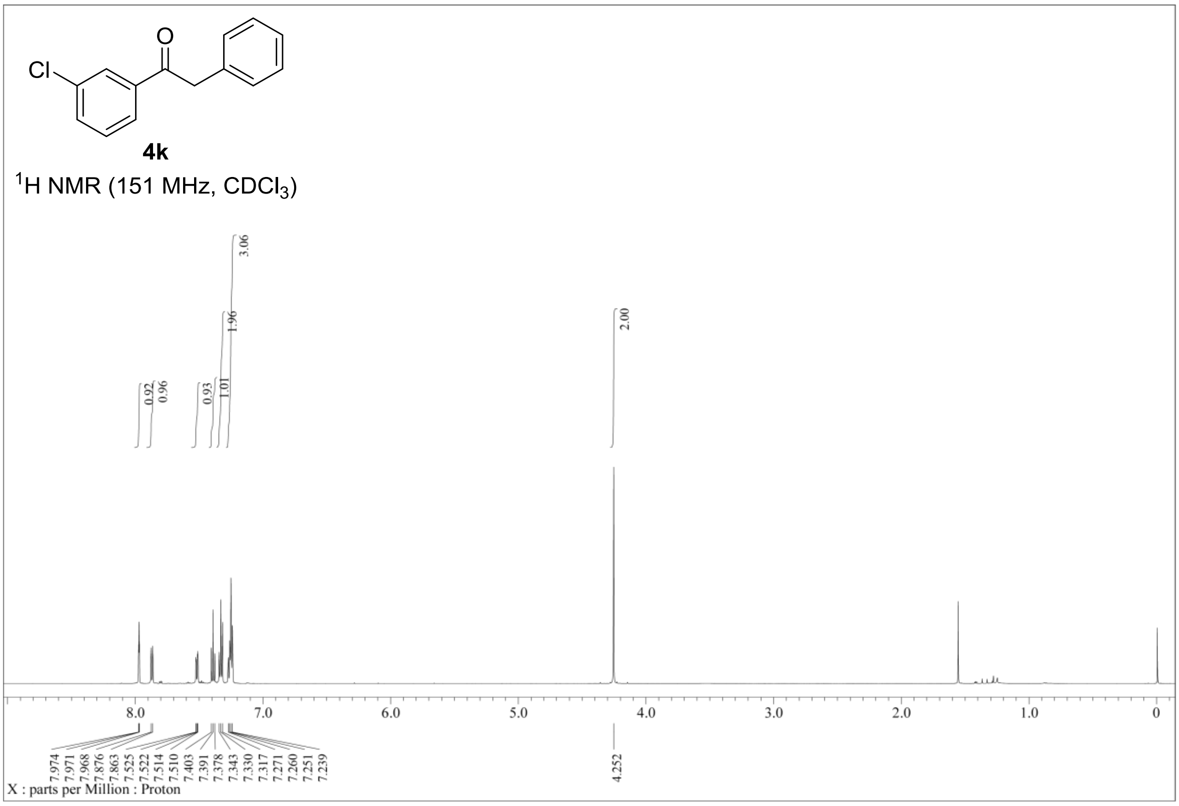

Cl
O
4k
^{1}H NMR (151 MHz, CDCl$_3$)
0.92
0.96
0.93
1.01
1.96
3.06
2.00
8.0
7.0
6.0
5.0
4.0
3.0
2.0
1.0
0
7.974
7.971
7.968
7.876
7.863
7.525
7.522
7.514
7.510
7.403
7.391
7.378
7.343
7.330
7.317
7.271
7.260
7.251
7.239
4.252
X : parts per Million : Proton

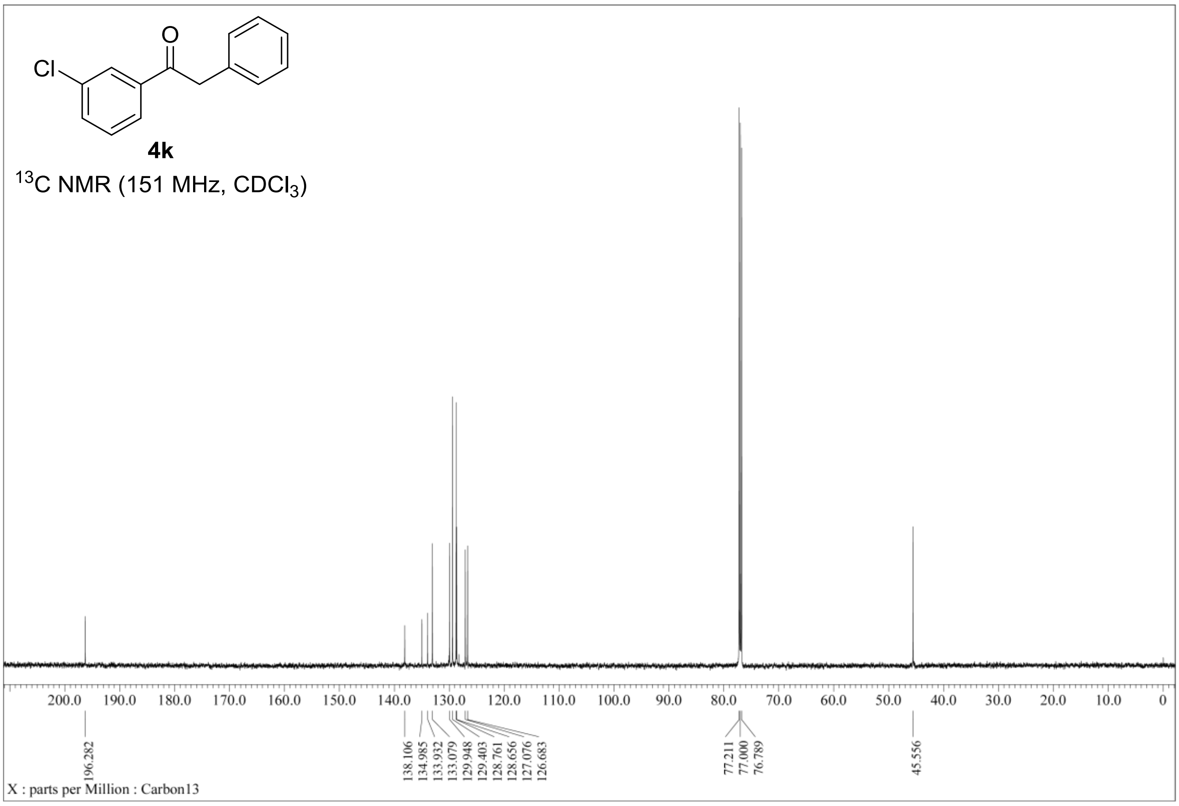

Cl
O
4k
^{13}C NMR (151 MHz, CDCl$_3$)
200.0
190.0
180.0
170.0
160.0
150.0
140.0
130.0
120.0
110.0
100.0
90.0
80.0
70.0
60.0
50.0
40.0
30.0
20.0
10.0
0
196.282
138.106
134.985
133.932
133.079
129.948
129.403
128.761
128.656
127.076
126.683
77.211
77.000
76.789
45.556
X : parts per Million : Carbon13

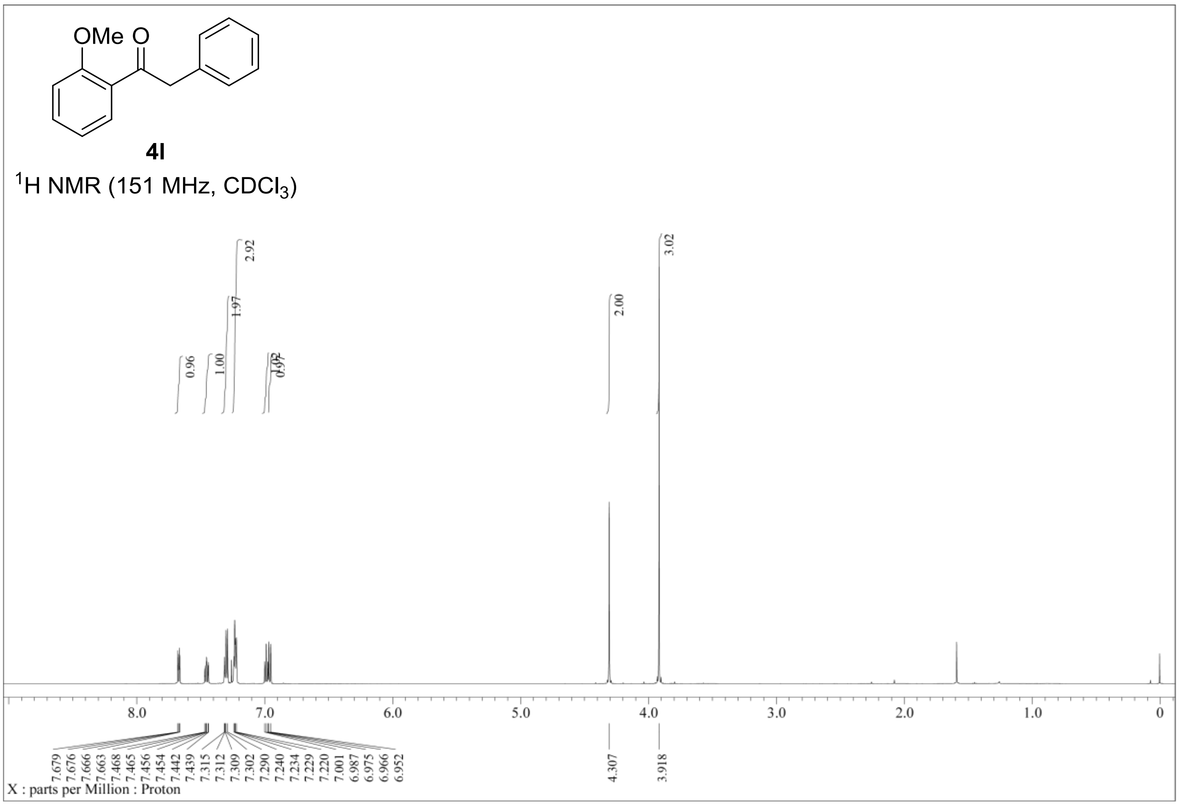
OMe O
4l
1H NMR (151 MHz, CDCl3)
0.96
1.00
1.97
2.92
2.00
3.02
8.0
7.0
6.0
5.0
4.0
3.0
2.0
1.0
0
7.679 7.676 7.666 7.663 7.468 7.465 7.456 7.454 7.442 7.439 7.315 7.312 7.309 7.302 7.290 7.240 7.234 7.229 7.220 7.001 6.987 6.975 6.966 6.952
4.307
3.918
X : parts per Million : Proton

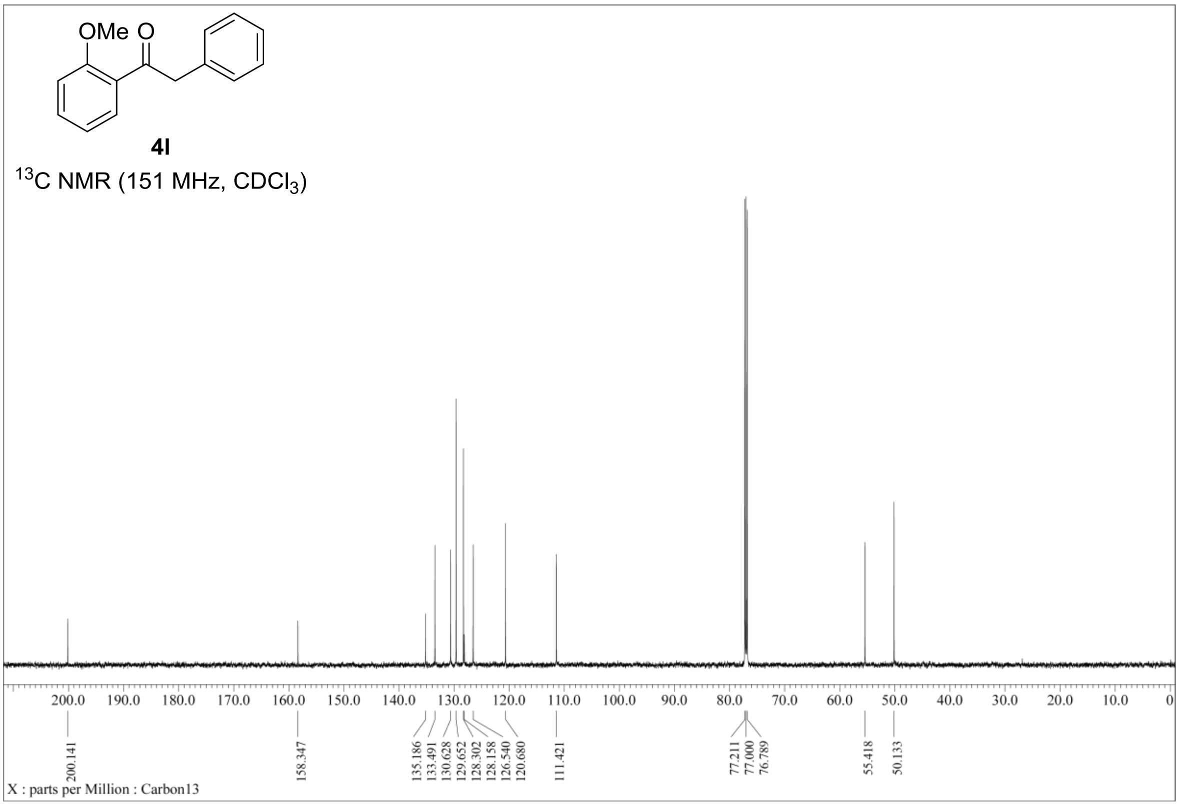
OMe O
4l
13C NMR (151 MHz, CDCl3)
200.0 190.0 180.0 170.0 160.0 150.0 140.0 130.0 120.0 110.0 100.0 90.0 80.0 70.0 60.0 50.0 40.0 30.0 20.0 10.0 0
200.141
158.347
135.186 133.491 130.628 129.652 128.302 128.158 126.540 120.680
111.421
77.211 77.000 76.789
55.418
50.133
X : parts per Million : Carbon13

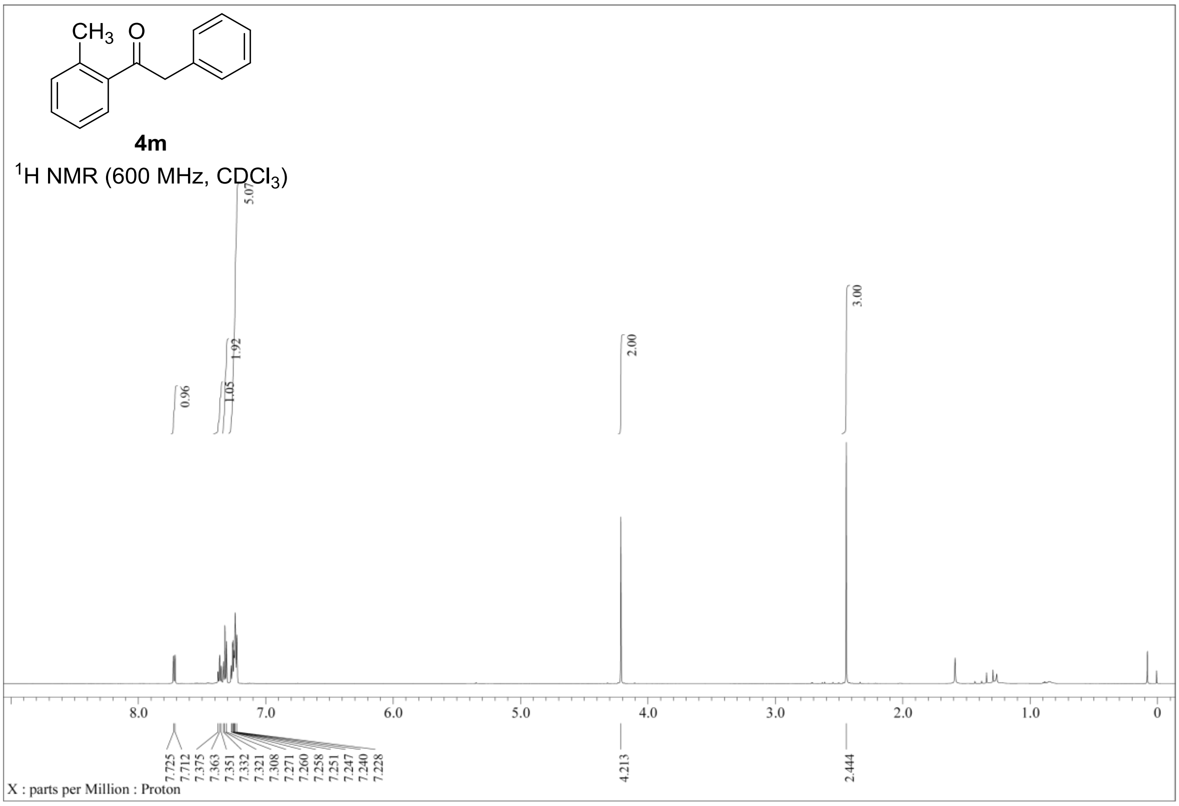
CH3
O
4m
1H NMR (600 MHz, CDCl3)
0.96
1.05
1.92
5.07
2.00
3.00
8.0
7.0
6.0
5.0
4.0
3.0
2.0
1.0
0
7.725
7.712
7.375
7.363
7.351
7.332
7.321
7.308
7.271
7.260
7.258
7.251
7.247
7.240
7.228
4.213
2.444
X : parts per Million : Proton

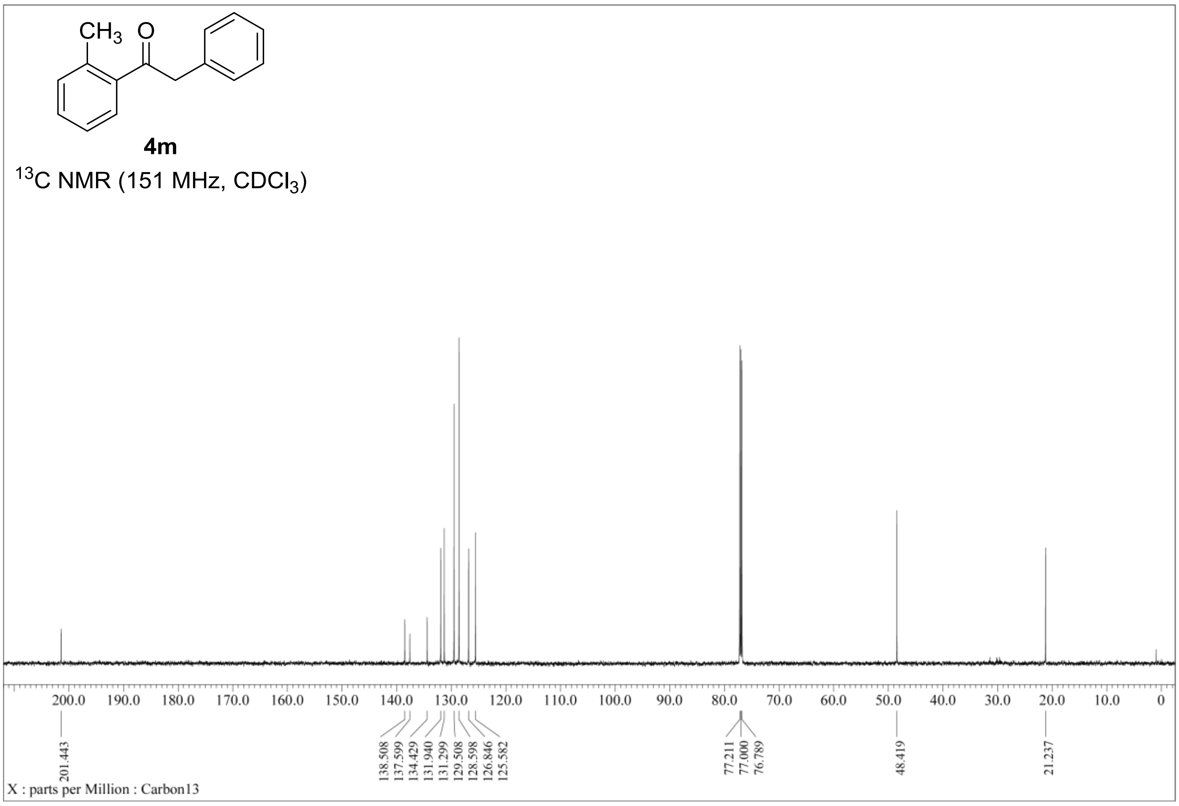
CH3
O
4m
13C NMR (151 MHz, CDCl3)
200.0
190.0
180.0
170.0
160.0
150.0
140.0
130.0
120.0
110.0
100.0
90.0
80.0
70.0
60.0
50.0
40.0
30.0
20.0
10.0
0
201.443
138.508
137.599
134.429
131.940
131.299
129.508
128.598
126.846
125.582
77.211
77.000
76.789
48.419
21.237
X : parts per Million : Carbon13

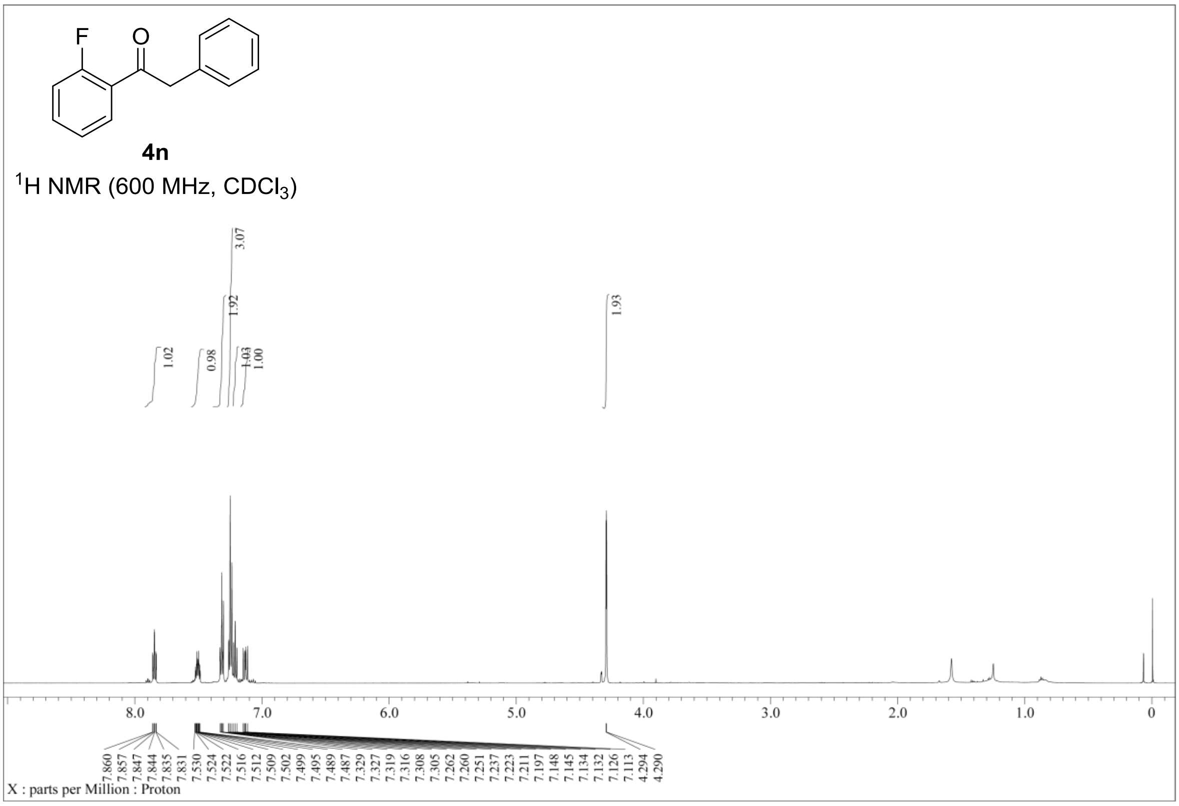

F
O
4n
1H NMR (600 MHz, CDCl3)
X : parts per Million : Proton

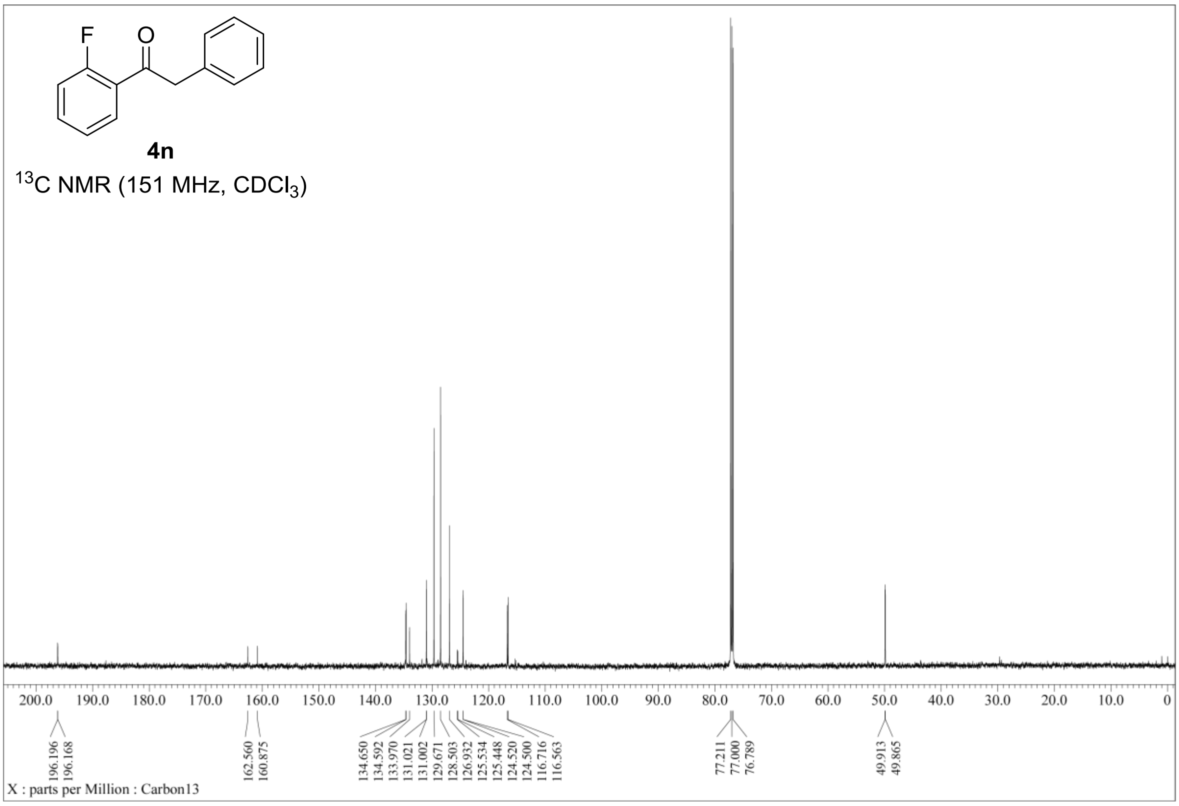

F
O
4n
13C NMR (151 MHz, CDCl3)
X : parts per Million : Carbon13

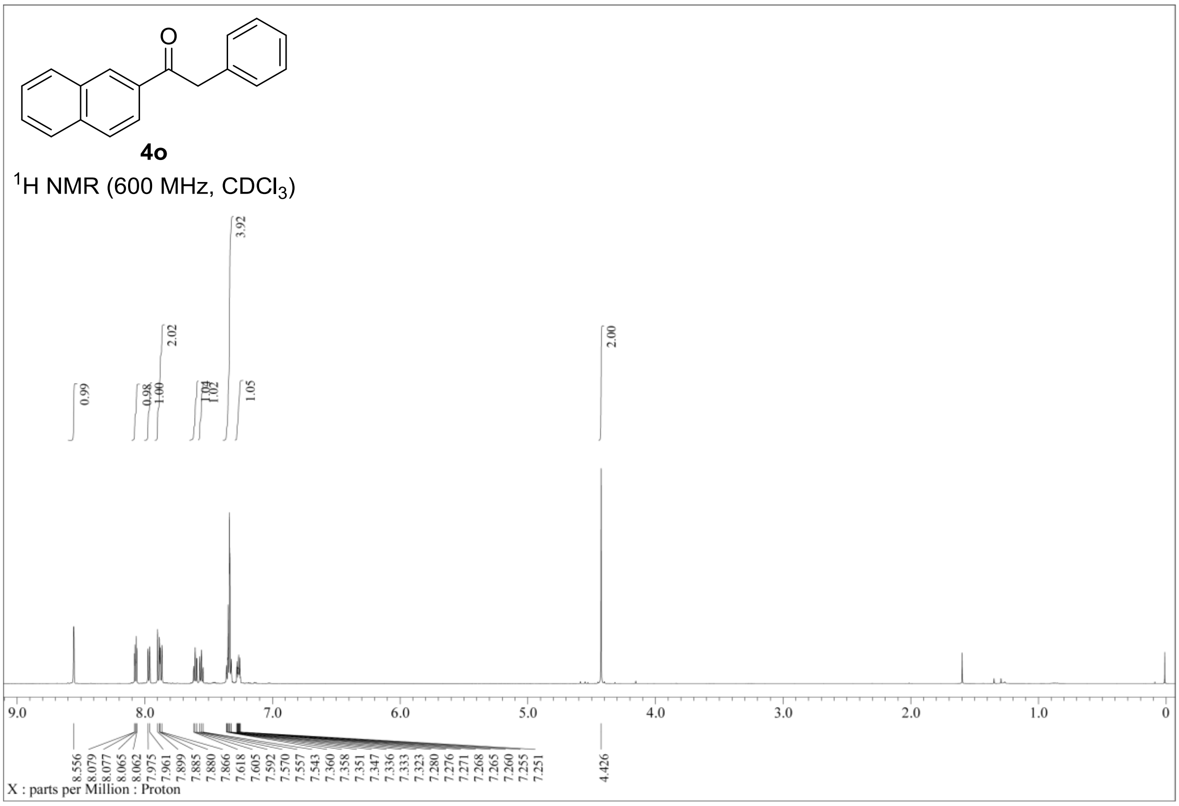
4o
^1H NMR (600 MHz, $CDCl_3$)
X : parts per Million : Proton

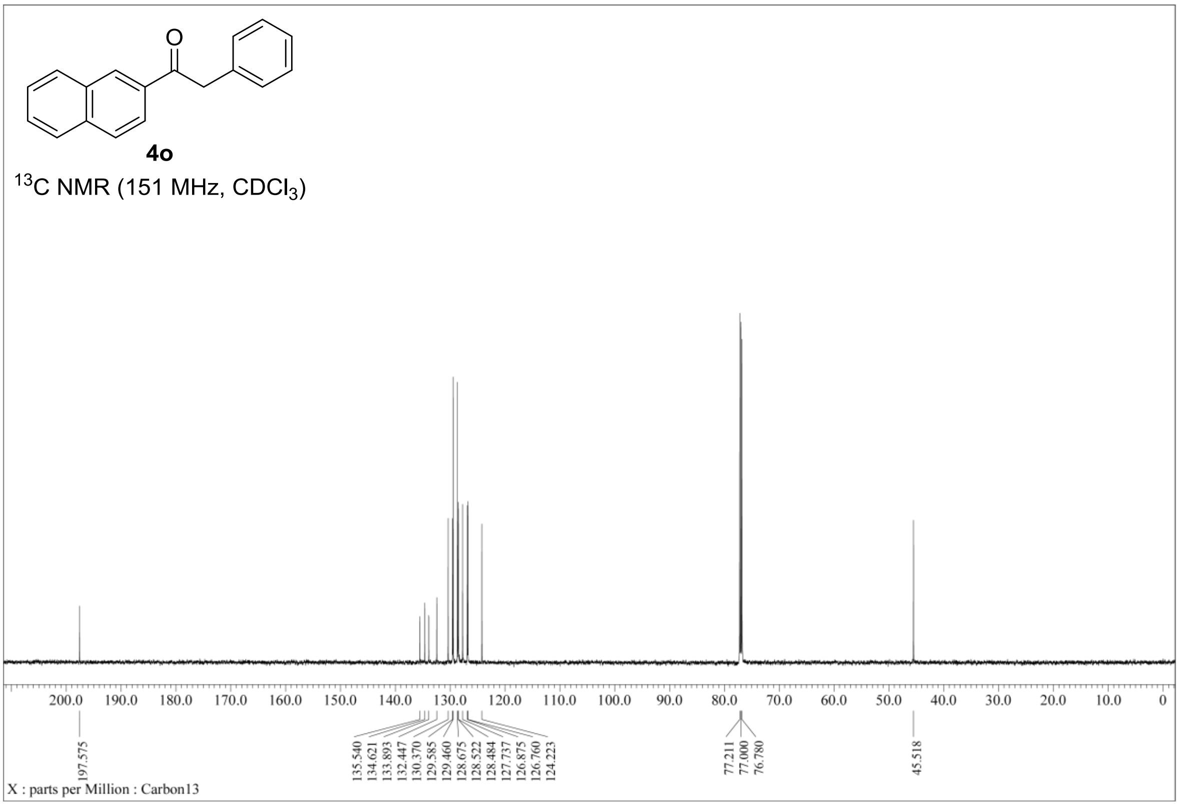
4o
^{13}C NMR (151 MHz, $CDCl_3$)
X : parts per Million : Carbon13

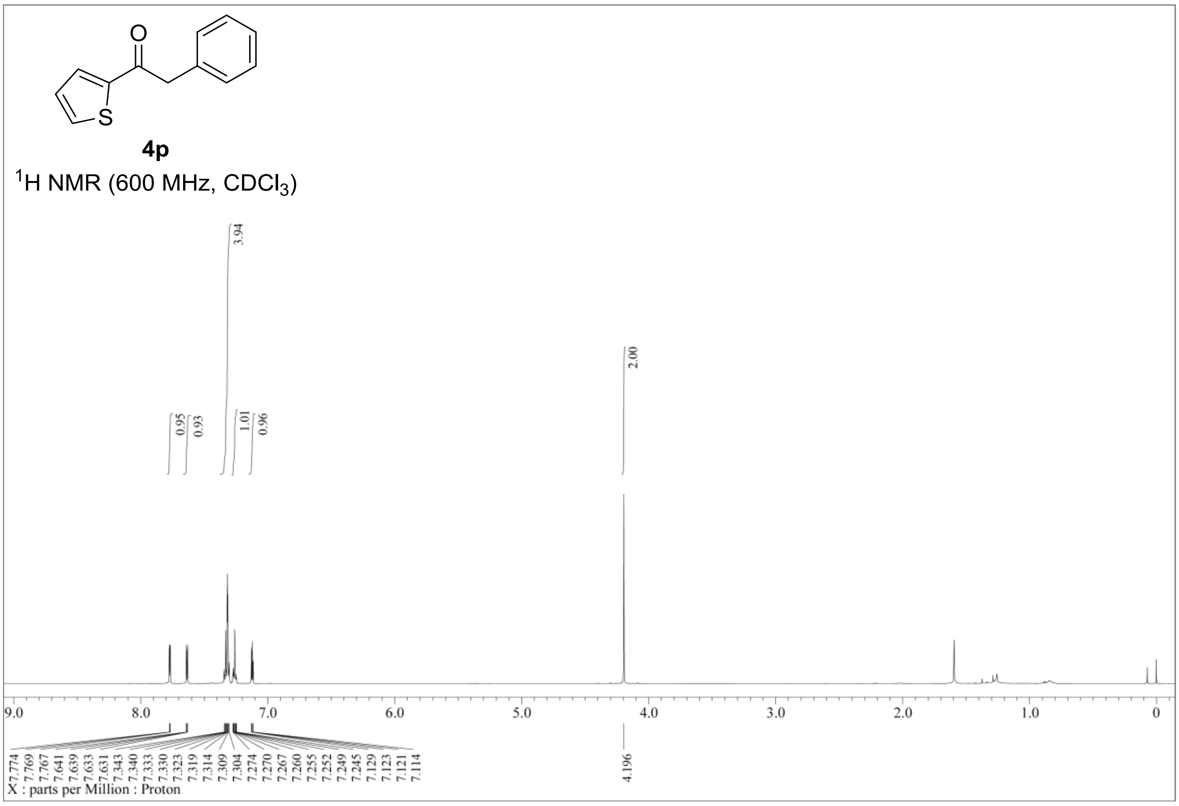
O
S
4p
1H NMR (600 MHz, CDCl3)
3.94
2.00
0.95
0.93
1.01
0.96
9.0 8.0 7.0 6.0 5.0 4.0 3.0 2.0 1.0 0
7.774 7.769 7.767 7.641 7.639 7.633 7.631 7.343 7.340 7.333 7.330 7.323 7.319 7.314 7.309 7.304 7.274 7.270 7.267 7.260 7.255 7.252 7.249 7.245 7.129 7.123 7.121 7.114
4.196
X : parts per Million : Proton

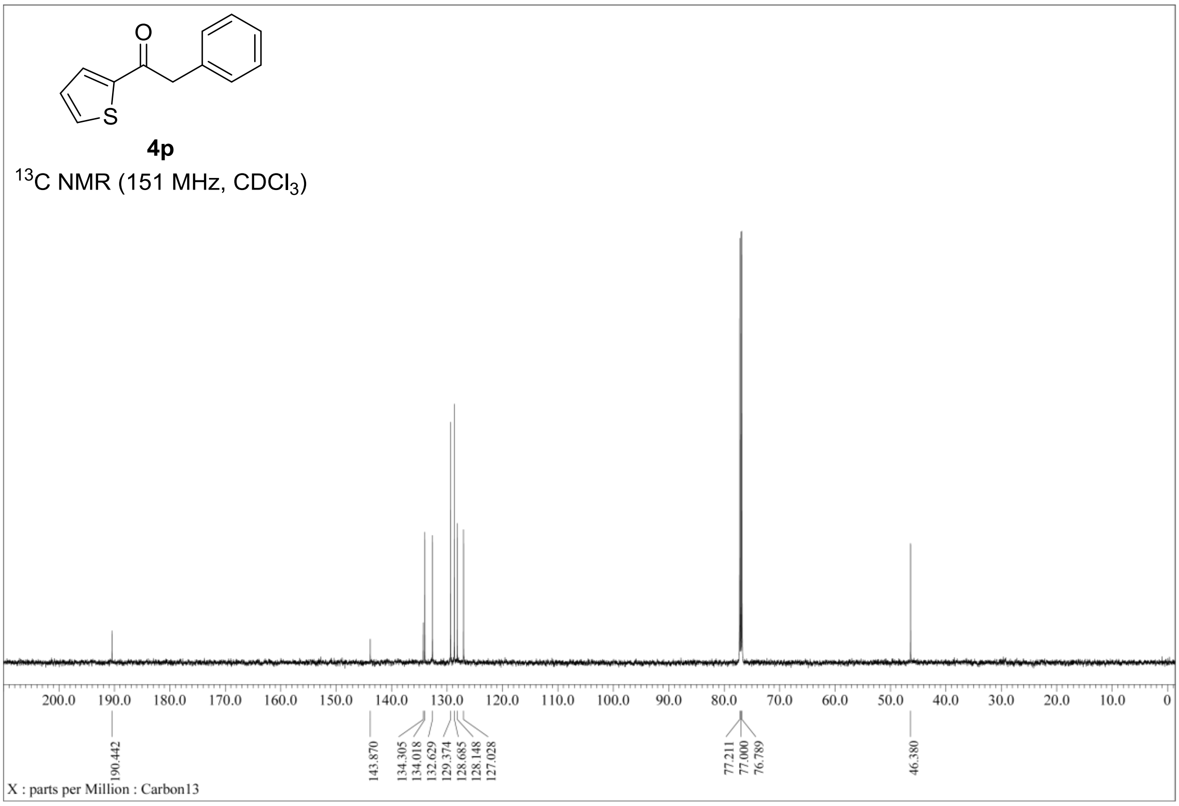
O
S
4p
13C NMR (151 MHz, CDCl3)
200.0 190.0 180.0 170.0 160.0 150.0 140.0 130.0 120.0 110.0 100.0 90.0 80.0 70.0 60.0 50.0 40.0 30.0 20.0 10.0 0
190.442
143.870
134.305 134.018 132.629 129.374 128.685 128.148 127.028
77.211 77.000 76.789
46.380
X : parts per Million : Carbon13

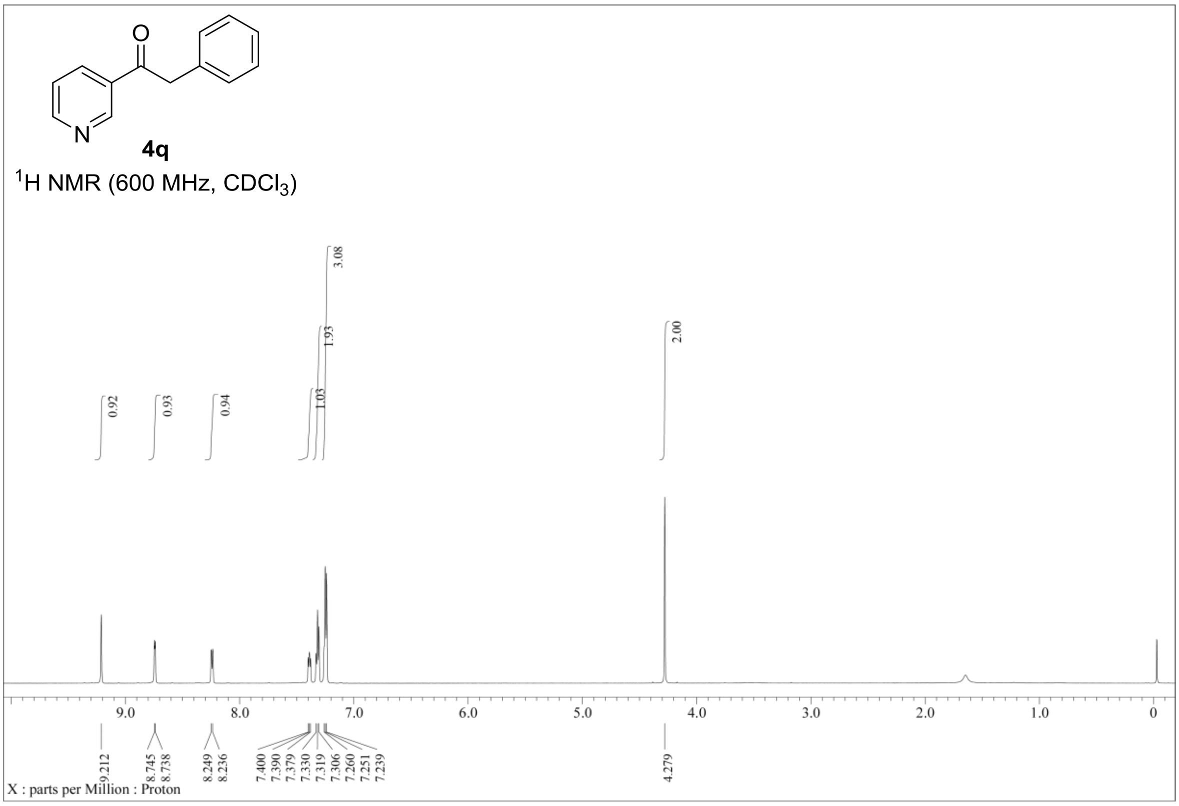
O
N
4q
^{1}H NMR (600 MHz, $CDCl_3$)
0.92
0.93
0.94
1.03
1.93
3.08
2.00
9.0 8.0 7.0 6.0 5.0 4.0 3.0 2.0 1.0 0
9.212
8.745
8.738
8.249
8.236
7.400
7.390
7.379
7.330
7.319
7.306
7.260
7.251
7.239
4.279
X : parts per Million : Proton

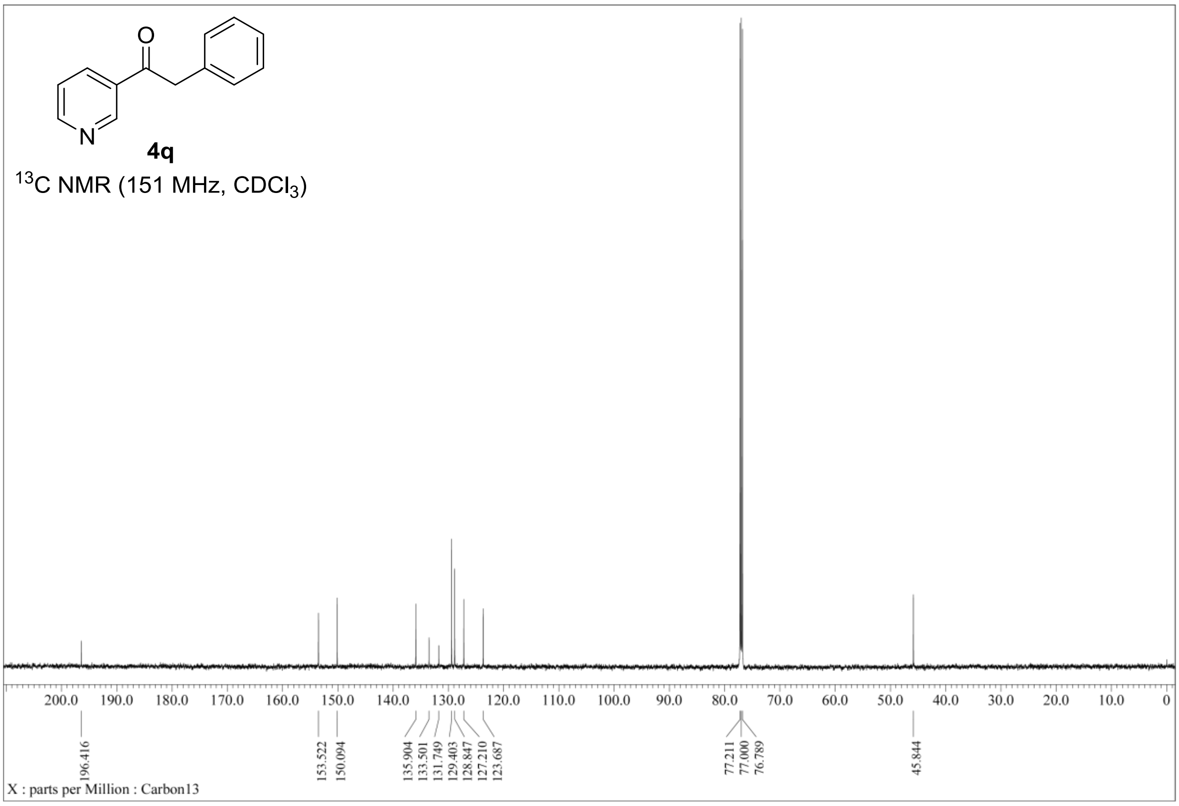
O
N
4q
^{13}C NMR (151 MHz, $CDCl_3$)
200.0 190.0 180.0 170.0 160.0 150.0 140.0 130.0 120.0 110.0 100.0 90.0 80.0 70.0 60.0 50.0 40.0 30.0 20.0 10.0 0
196.416
153.522
150.094
135.904
133.501
131.749
129.403
128.847
127.210
123.687
77.211
77.000
76.789
45.844
X : parts per Million : Carbon13

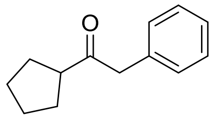

4r

^{1}H NMR (600 MHz, $CDCl_3$)

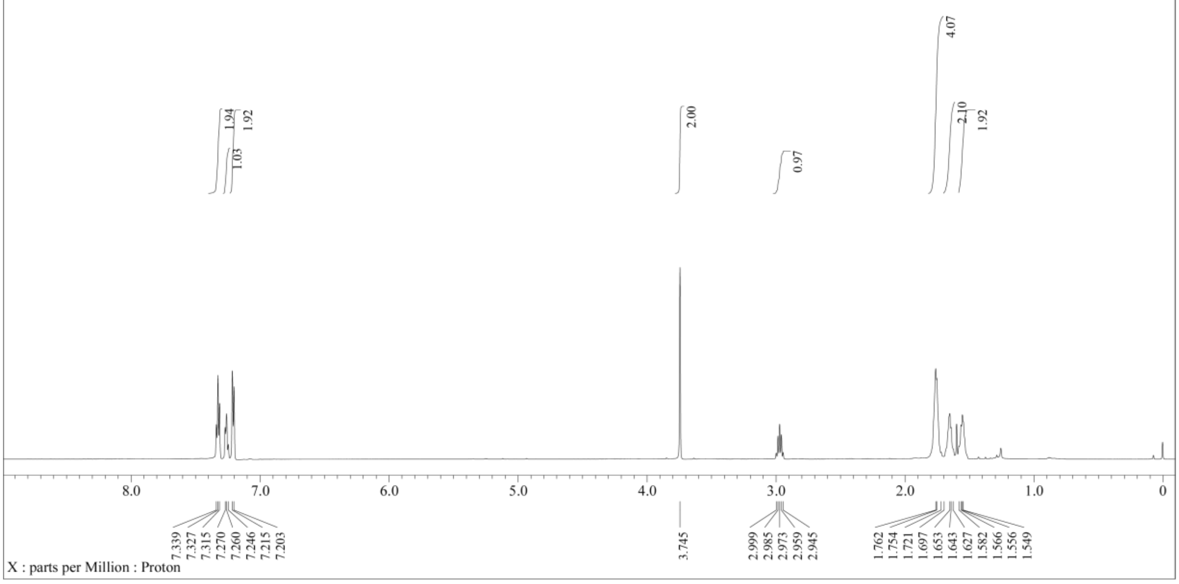

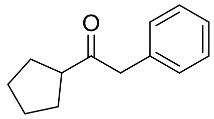

4r

^{13}C NMR (151 MHz, $CDCl_3$)

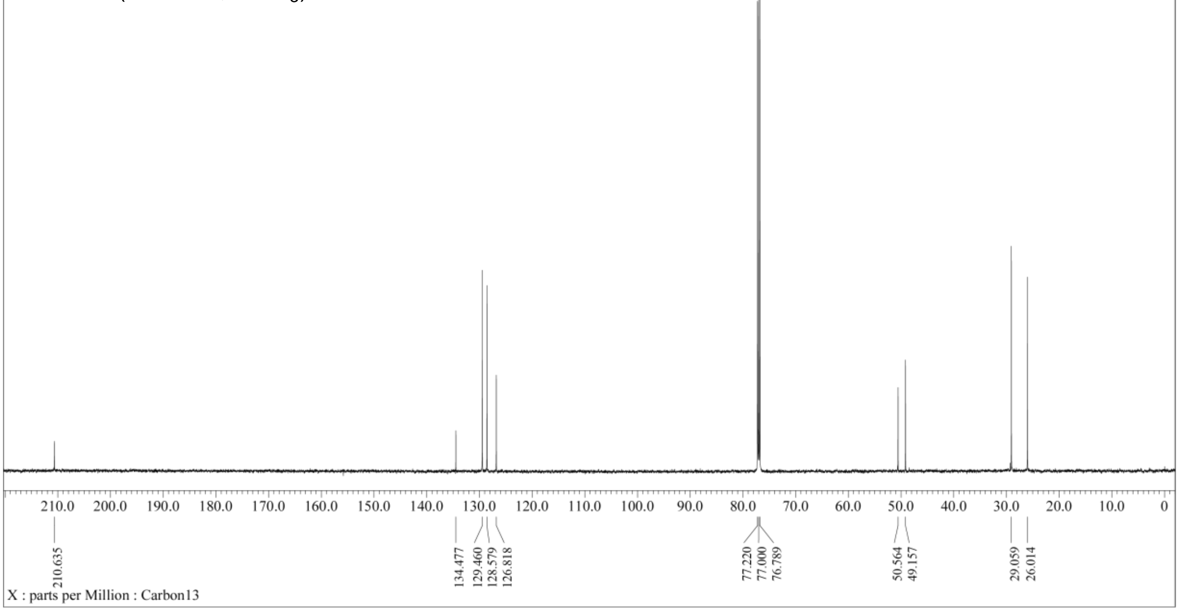

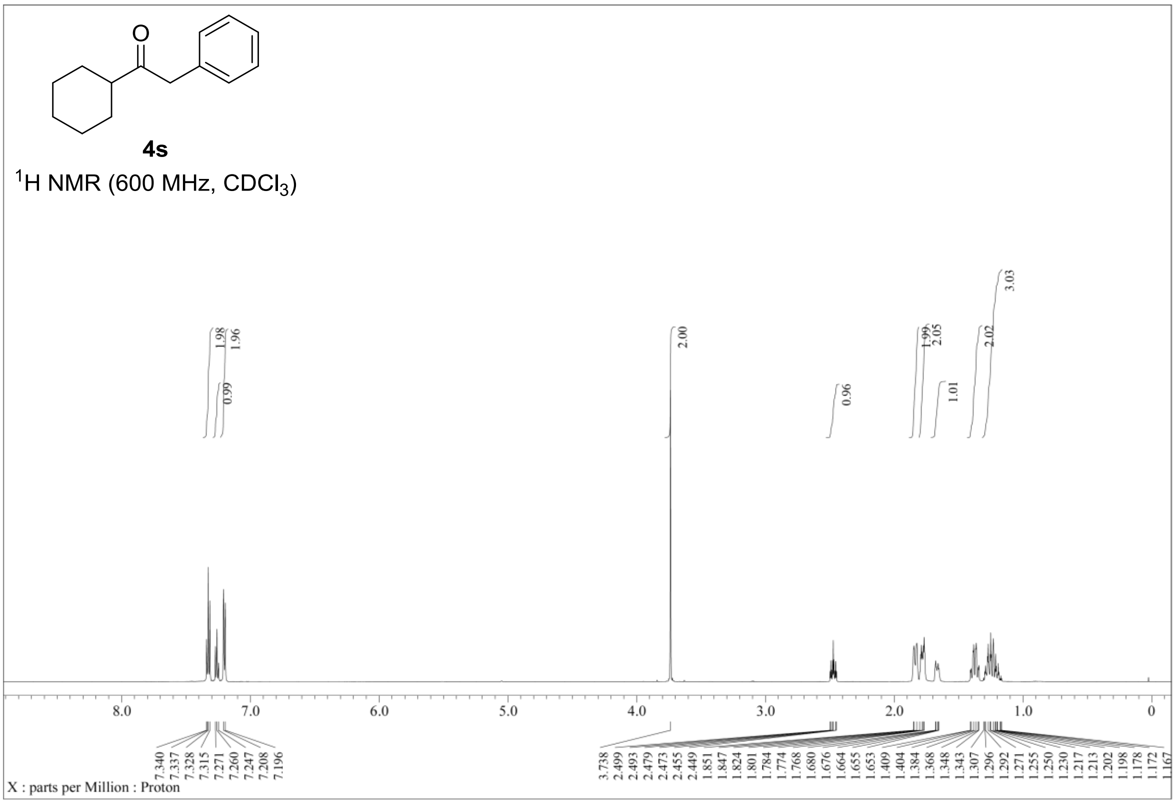
O
4s
^{1}H NMR (600 MHz, CDCl$_3$)
1.98
1.96
0.99
2.00
0.96
1.99
2.05
1.01
2.02
3.03
8.0
7.0
6.0
5.0
4.0
3.0
2.0
1.0
0
7.340
7.337
7.328
7.315
7.271
7.260
7.247
7.208
7.196
3.738
2.499
2.493
2.479
2.473
2.455
2.449
1.851
1.847
1.824
1.801
1.784
1.774
1.768
1.680
1.676
1.664
1.655
1.653
1.409
1.404
1.384
1.368
1.348
1.343
1.307
1.296
1.292
1.271
1.255
1.250
1.230
1.217
1.213
1.202
1.198
1.178
1.172
1.167
X : parts per Million : Proton

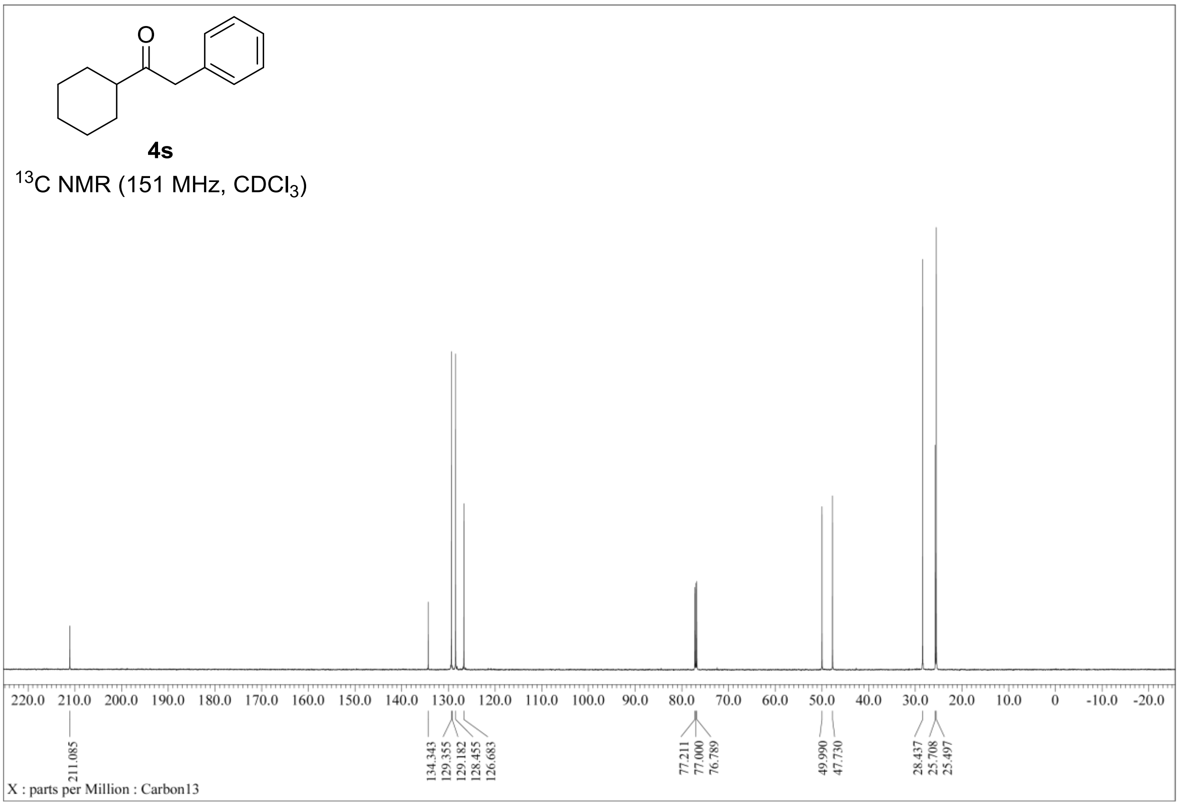
O
4s
^{13}C NMR (151 MHz, CDCl$_3$)
220.0
210.0
200.0
190.0
180.0
170.0
160.0
150.0
140.0
130.0
120.0
110.0
100.0
90.0
80.0
70.0
60.0
50.0
40.0
30.0
20.0
10.0
0
-10.0
-20.0
211.085
134.343
129.355
129.182
128.455
126.683
77.211
77.000
76.789
49.990
47.730
28.437
25.708
25.497
X : parts per Million : Carbon13

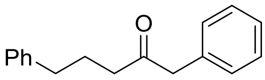

4t

^{1}H NMR (600 MHz, $CDCl_3$)

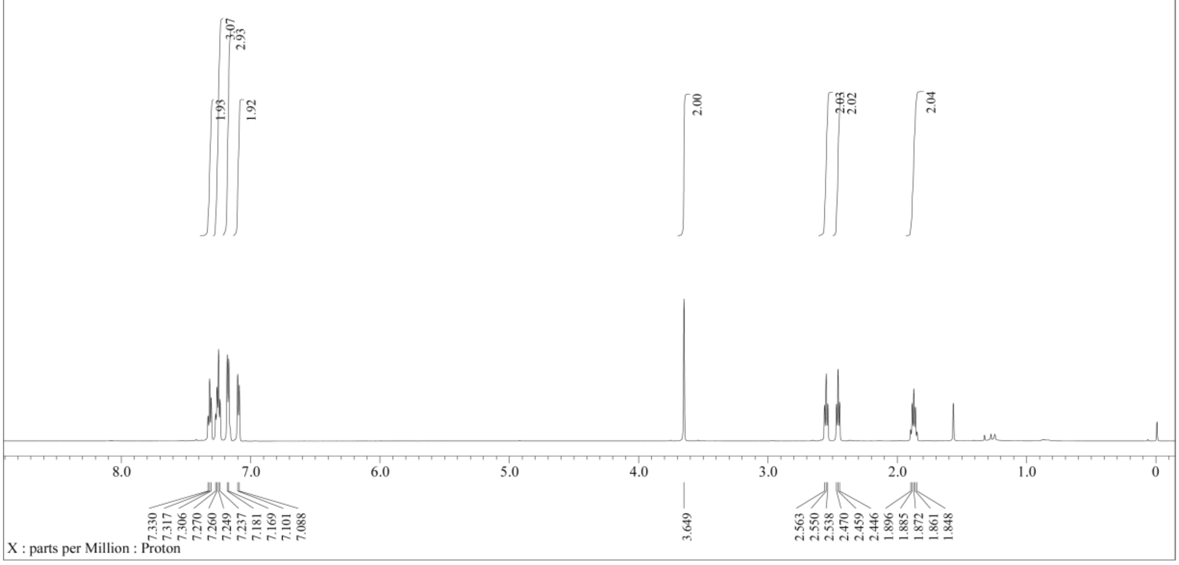

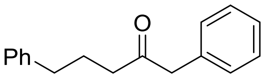

4t

^{13}C NMR (151 MHz, $CDCl_3$)

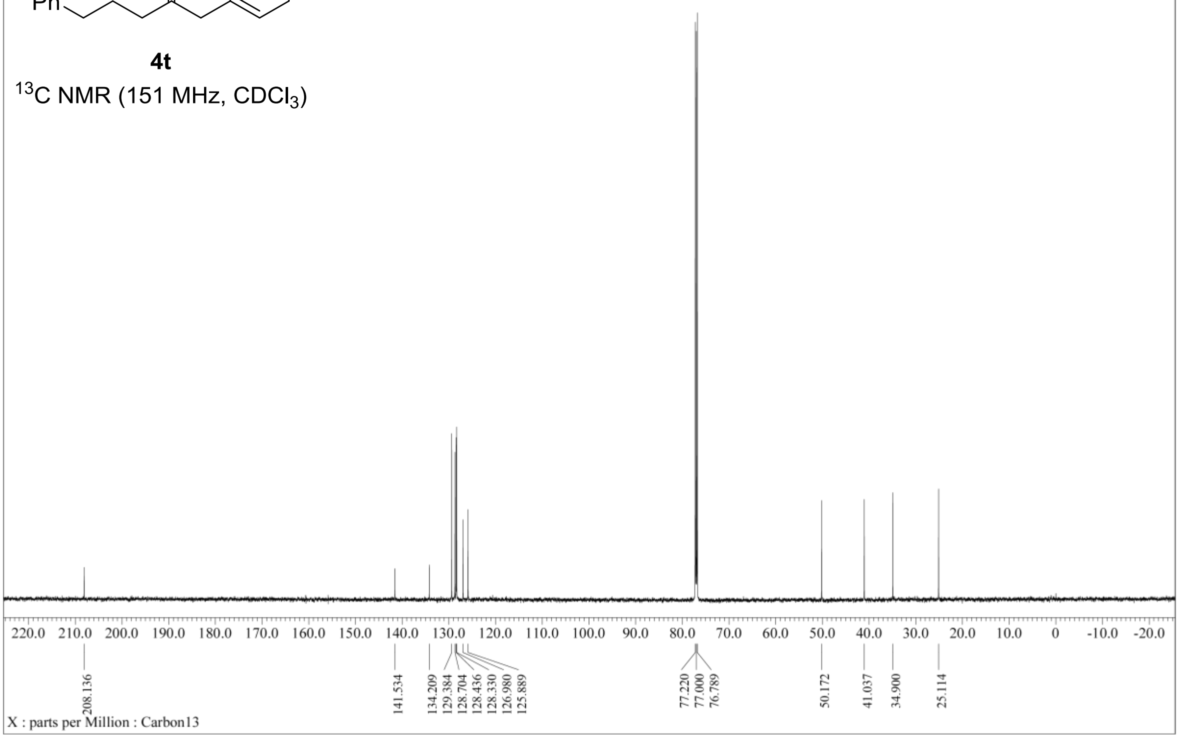

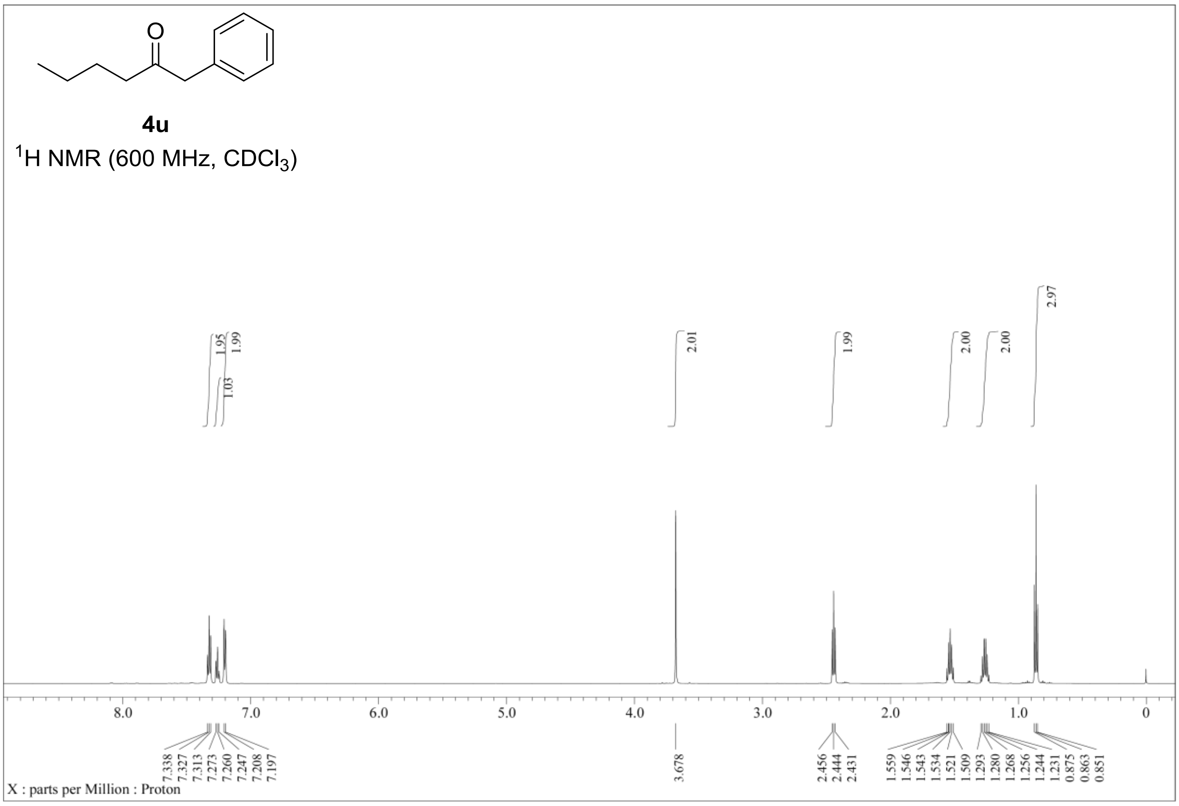
O
4u
1H NMR (600 MHz, CDCl3)
X : parts per Million : Proton

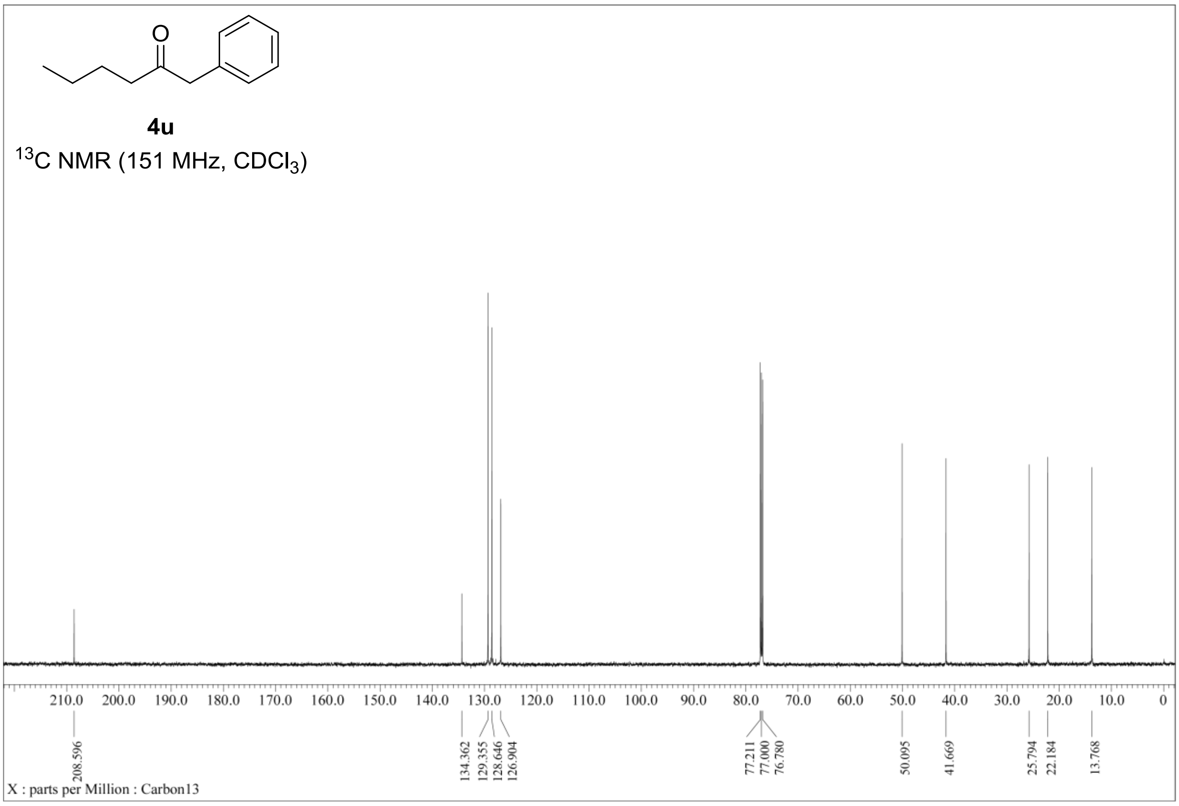
O
4u
13C NMR (151 MHz, CDCl3)
X : parts per Million : Carbon13

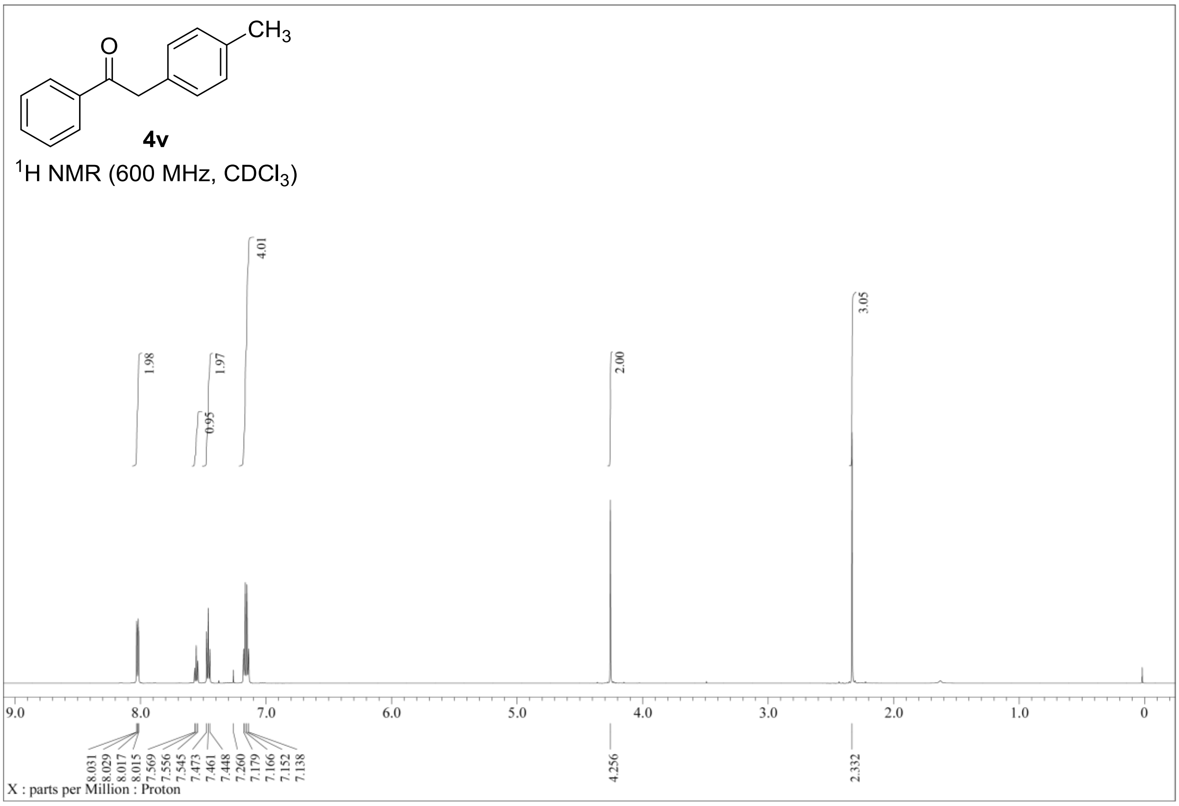
CH3
O
4v
1H NMR (600 MHz, CDCl3)
1.98
0.95
1.97
4.01
2.00
3.05
9.0 8.0 7.0 6.0 5.0 4.0 3.0 2.0 1.0 0
8.031 8.029 8.017 8.015 7.569 7.556 7.545 7.473 7.461 7.448 7.260 7.179 7.166 7.152 7.138
4.256
2.332
X : parts per Million : Proton

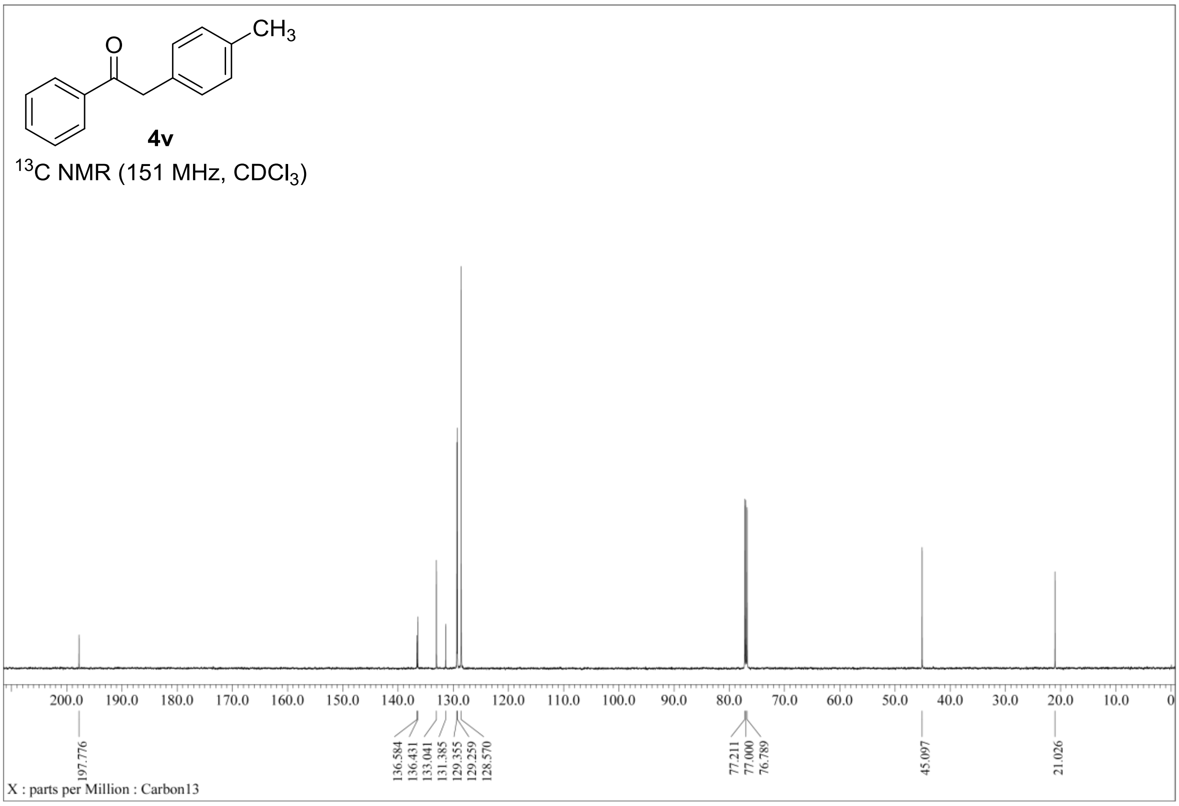
CH3
O
4v
13C NMR (151 MHz, CDCl3)
200.0 190.0 180.0 170.0 160.0 150.0 140.0 130.0 120.0 110.0 100.0 90.0 80.0 70.0 60.0 50.0 40.0 30.0 20.0 10.0 0
197.776
136.584 136.431 133.041 131.385 129.355 129.259 128.570
77.211 77.000 76.789
45.097
21.026
X : parts per Million : Carbon13

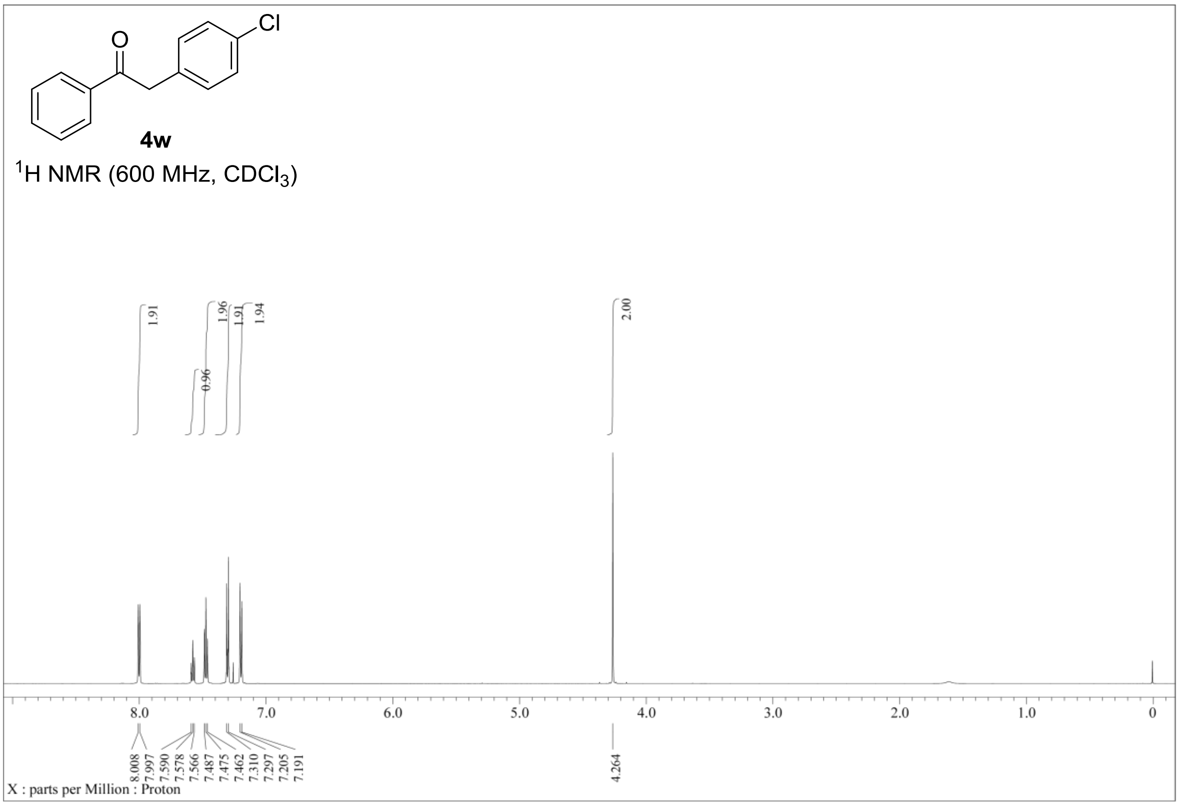
Cl
O
4w
^{1}H NMR (600 MHz, CDCl$_3$)
1.91
0.96
1.96
1.91
1.94
2.00
8.0
7.0
6.0
5.0
4.0
3.0
2.0
1.0
0
8.008
7.997
7.590
7.578
7.566
7.487
7.475
7.462
7.310
7.297
7.205
7.191
4.264
X : parts per Million : Proton

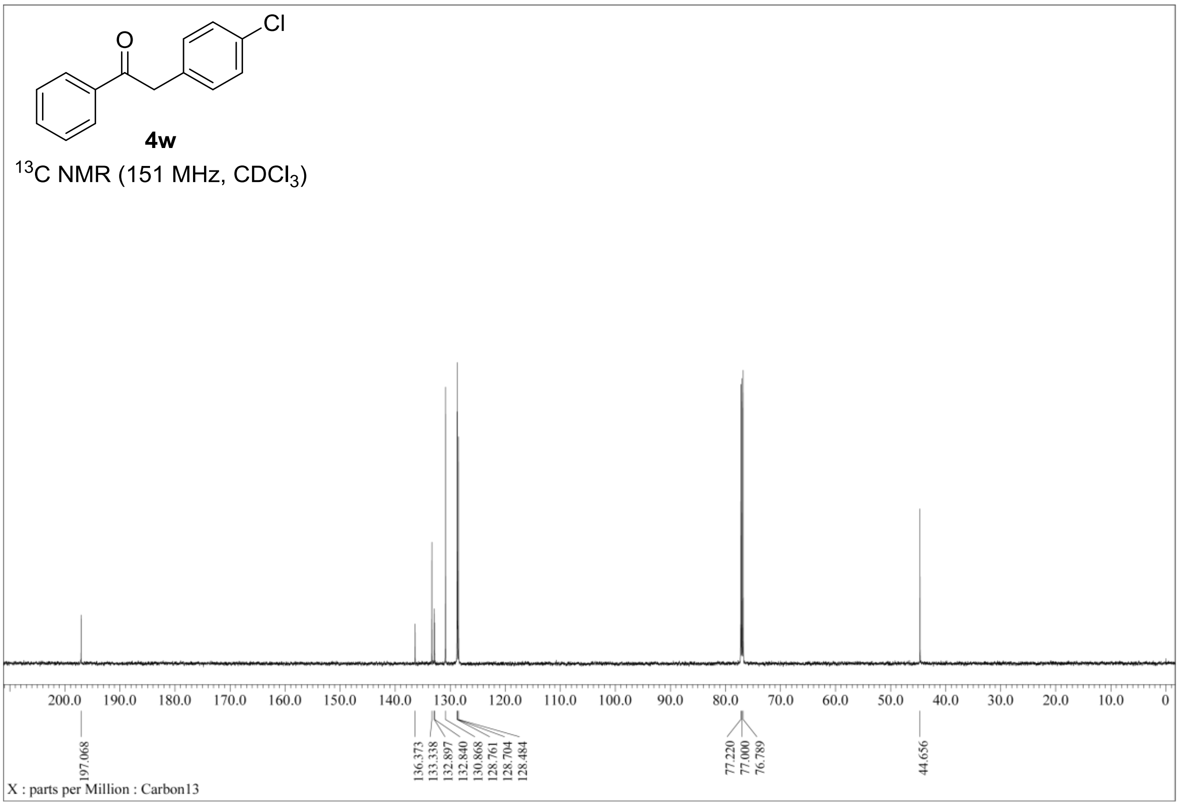
Cl
O
4w
^{13}C NMR (151 MHz, CDCl$_3$)
200.0
190.0
180.0
170.0
160.0
150.0
140.0
130.0
120.0
110.0
100.0
90.0
80.0
70.0
60.0
50.0
40.0
30.0
20.0
10.0
0
197.068
136.373
133.338
132.897
132.840
130.868
128.761
128.704
128.484
77.220
77.000
76.789
44.656
X : parts per Million : Carbon13

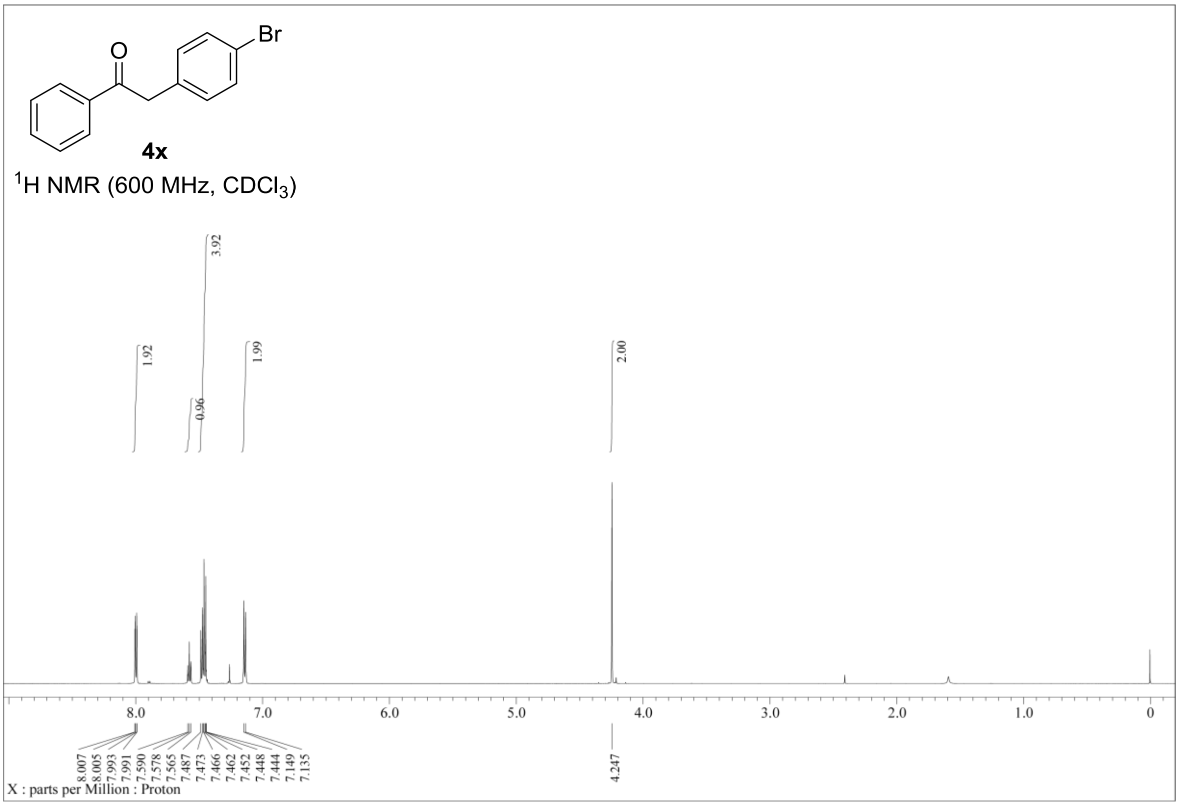
Br
O
4x
1H NMR (600 MHz, CDCl3)
1.92
0.96
3.92
1.99
2.00
8.0
7.0
6.0
5.0
4.0
3.0
2.0
1.0
0
8.007
8.005
7.993
7.991
7.590
7.578
7.565
7.487
7.473
7.466
7.462
7.452
7.448
7.444
7.149
7.135
4.247
X : parts per Million : Proton

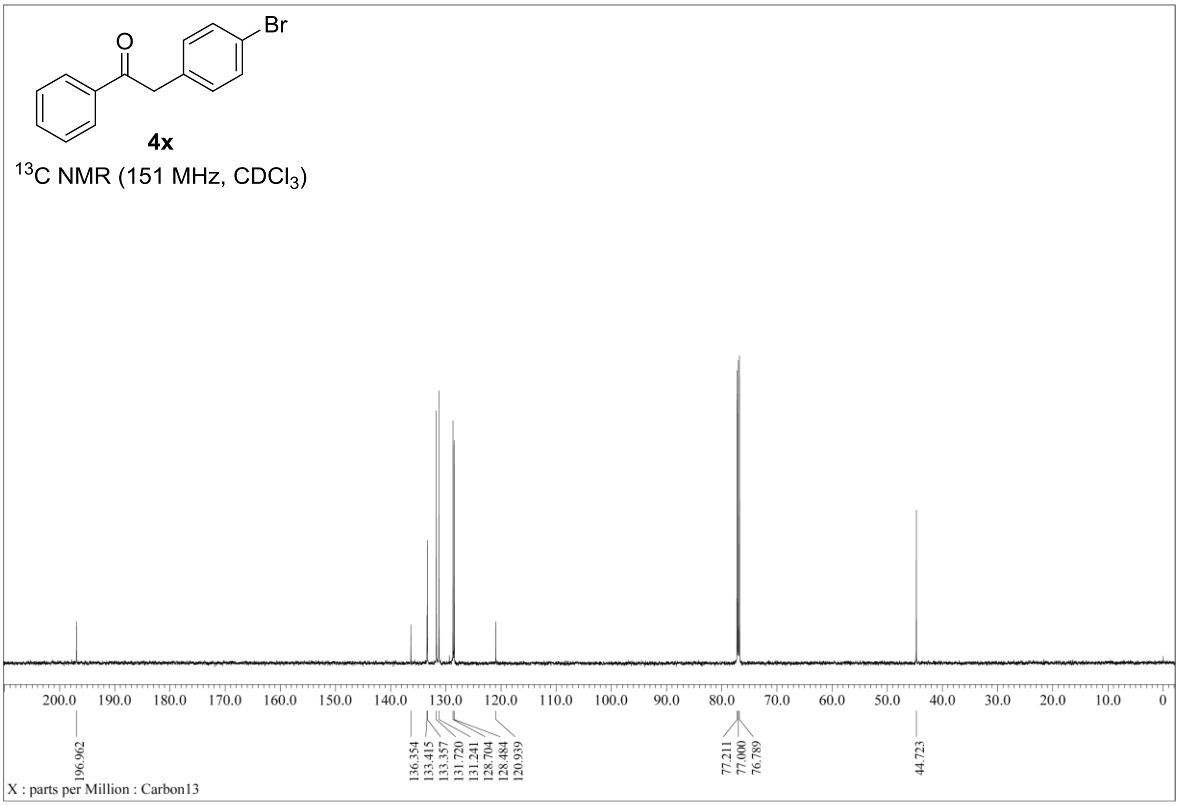
Br
O
4x
13C NMR (151 MHz, CDCl3)
200.0
190.0
180.0
170.0
160.0
150.0
140.0
130.0
120.0
110.0
100.0
90.0
80.0
70.0
60.0
50.0
40.0
30.0
20.0
10.0
0
196.962
136.354
133.415
133.357
131.720
131.241
128.704
128.484
120.939
77.211
77.000
76.789
44.723
X : parts per Million : Carbon13

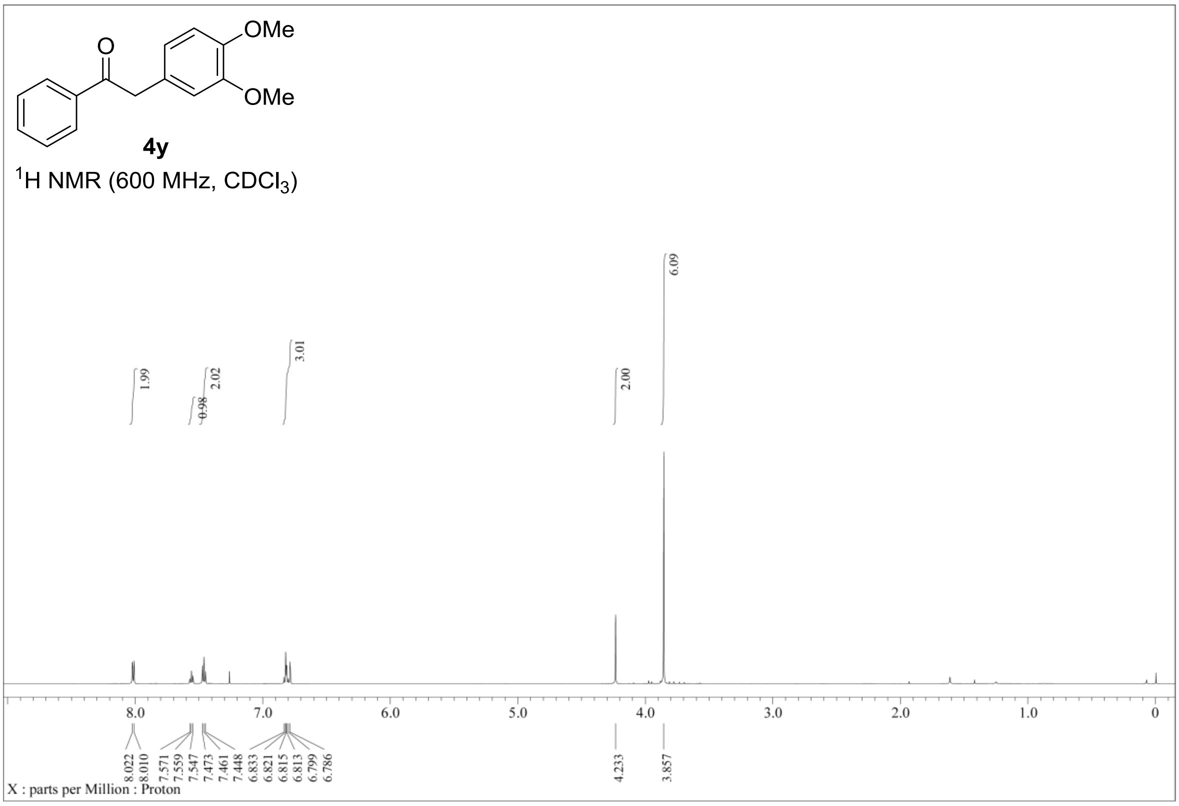
OMe
OMe
O
4y
1H NMR (600 MHz, CDCl3)
1.99
0.98
2.02
3.01
2.00
6.09
8.0
7.0
6.0
5.0
4.0
3.0
2.0
1.0
0
8.022
8.010
7.571
7.559
7.547
7.473
7.461
7.448
6.833
6.821
6.815
6.813
6.799
6.786
4.233
3.857
X : parts per Million : Proton

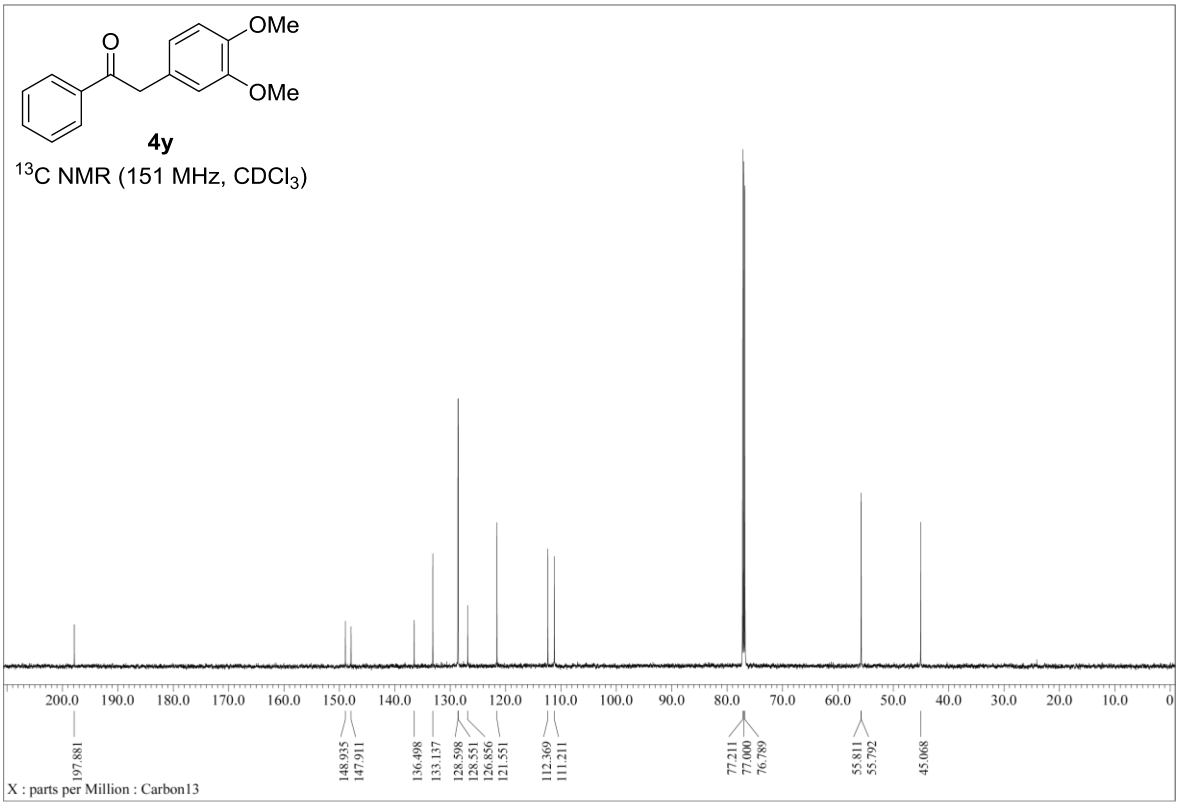
OMe
OMe
O
4y
13C NMR (151 MHz, CDCl3)
200.0
190.0
180.0
170.0
160.0
150.0
140.0
130.0
120.0
110.0
100.0
90.0
80.0
70.0
60.0
50.0
40.0
30.0
20.0
10.0
0
197.881
148.935
147.911
136.498
133.137
128.598
128.551
126.856
121.551
112.369
111.211
77.211
77.000
76.789
55.811
55.792
45.068
X : parts per Million : Carbon13

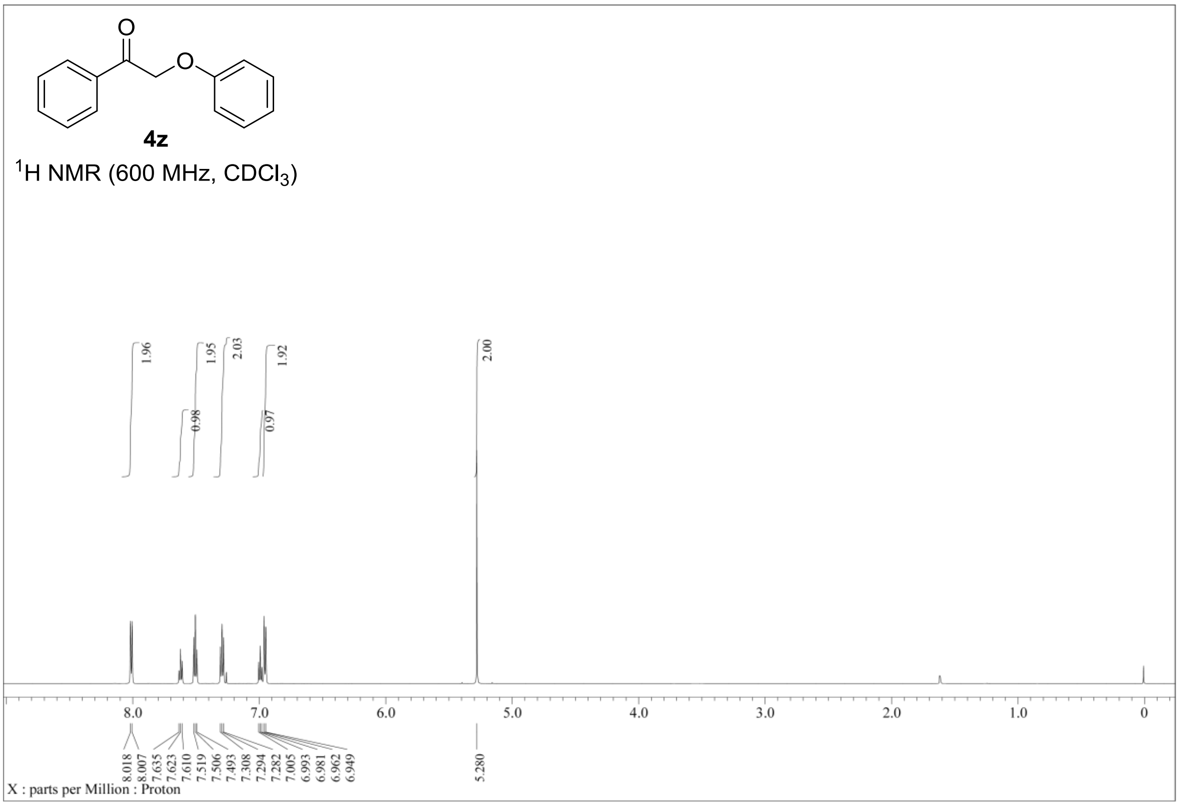

4z
^{1}H NMR (600 MHz, CDCl$_3$)
1.96
0.98
1.95
2.03
0.97
1.92
2.00
8.0
7.0
6.0
5.0
4.0
3.0
2.0
1.0
0
8.018
8.007
7.635
7.623
7.610
7.519
7.506
7.493
7.308
7.294
7.282
7.005
6.993
6.981
6.962
6.949
5.280
X : parts per Million : Proton

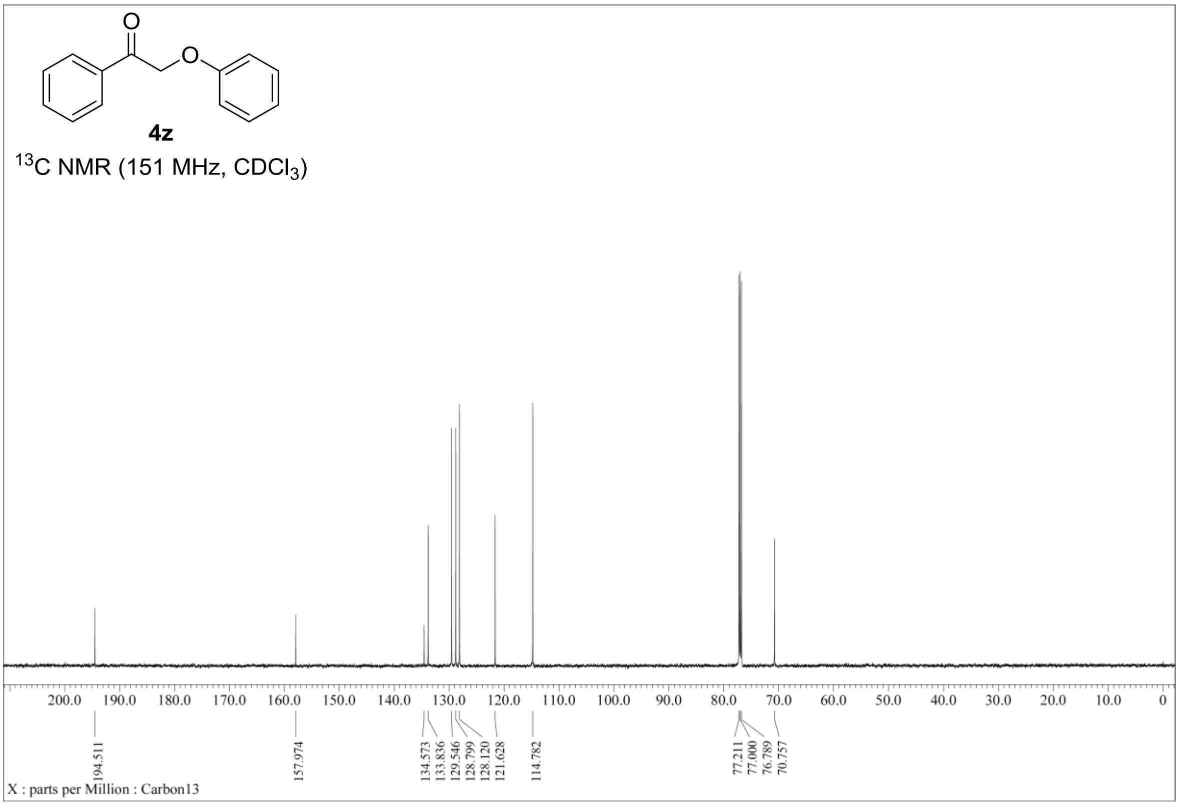

4z
^{13}C NMR (151 MHz, CDCl$_3$)
200.0
190.0
180.0
170.0
160.0
150.0
140.0
130.0
120.0
110.0
100.0
90.0
80.0
70.0
60.0
50.0
40.0
30.0
20.0
10.0
0
194.511
157.974
134.573
133.836
129.546
128.799
128.120
121.628
114.782
77.211
77.000
76.789
70.757
X : parts per Million : Carbon13

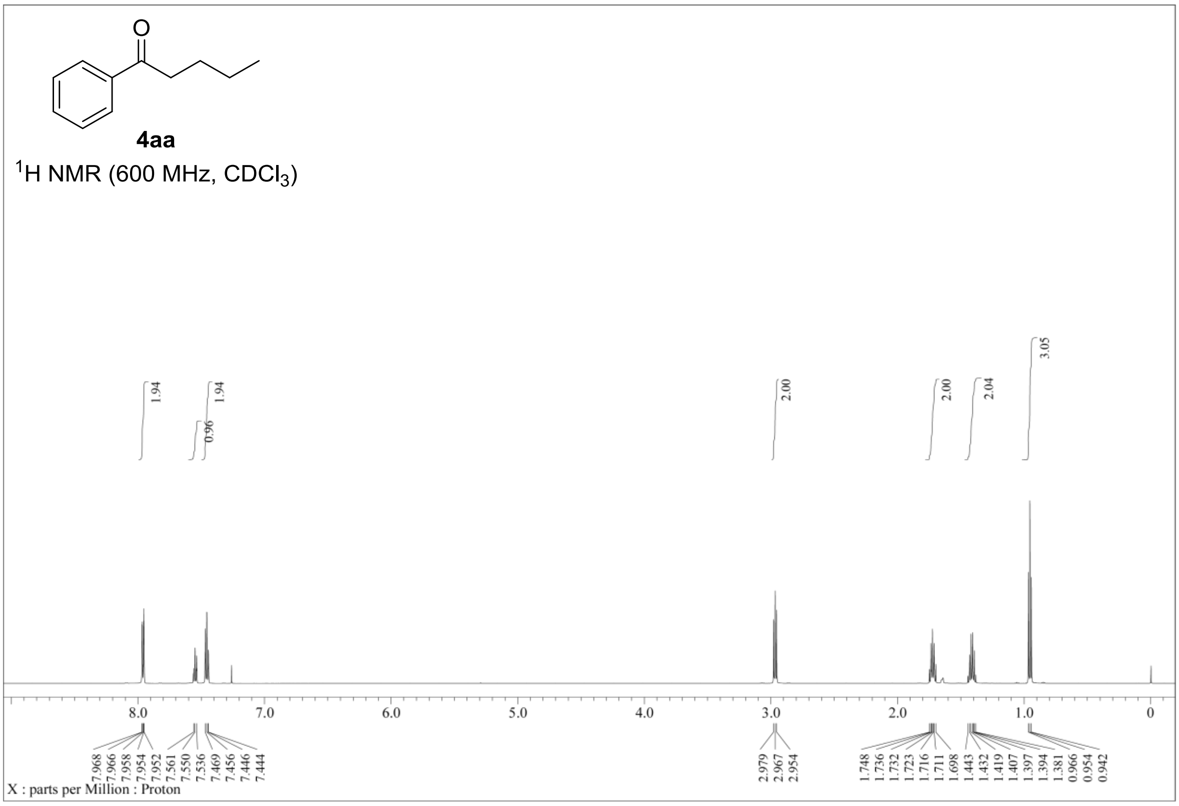
O
4aa
^{1}H NMR (600 MHz, CDCl$_3$)
1.94
0.96
1.94
2.00
2.00
2.04
3.05
8.0
7.0
6.0
5.0
4.0
3.0
2.0
1.0
0
7.968 7.966 7.958 7.954 7.952 7.561 7.550 7.536 7.469 7.456 7.446 7.444
2.979 2.967 2.954
1.748 1.736 1.732 1.723 1.716 1.711 1.698 1.443 1.432 1.419 1.407 1.397 1.394 1.381 0.966 0.954 0.942
X : parts per Million : Proton

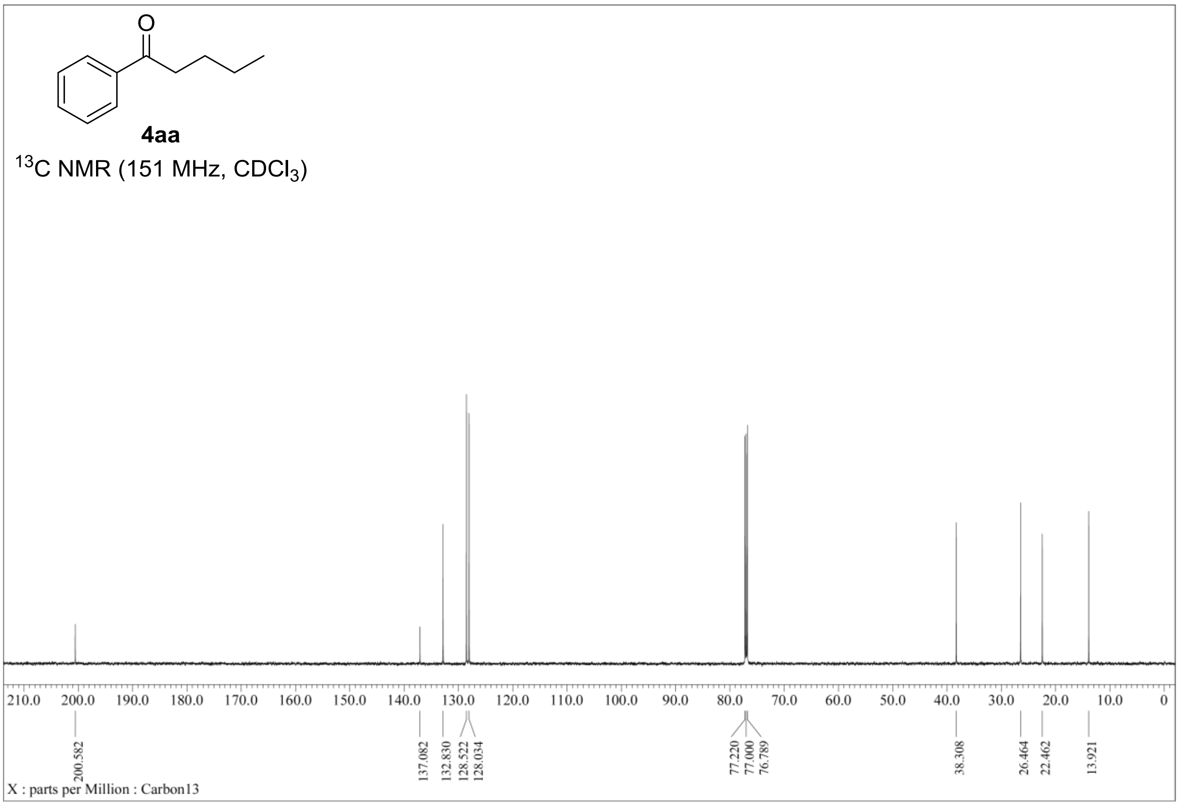
O
4aa
^{13}C NMR (151 MHz, CDCl$_3$)
210.0 200.0 190.0 180.0 170.0 160.0 150.0 140.0 130.0 120.0 110.0 100.0 90.0 80.0 70.0 60.0 50.0 40.0 30.0 20.0 10.0 0
200.582
137.082
132.830
128.522
128.034
77.220
77.000
76.789
38.308
26.464
22.462
13.921
X : parts per Million : Carbon13

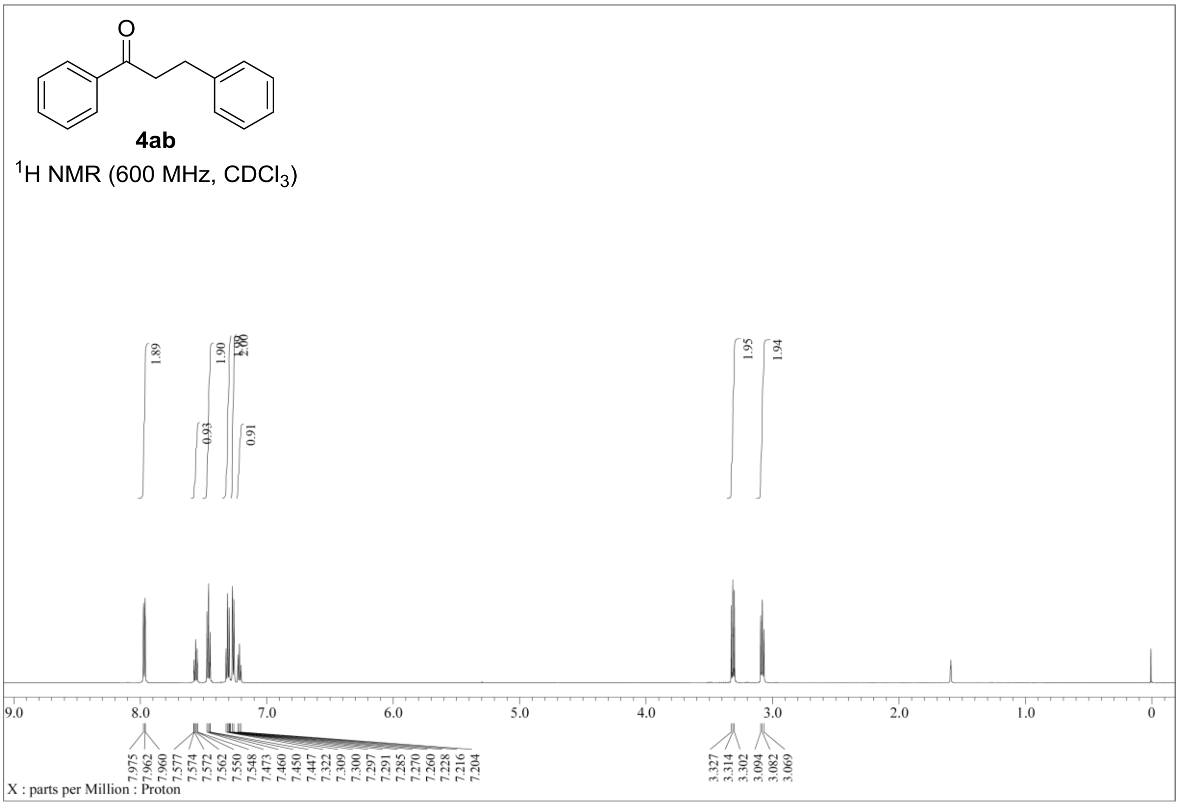
O
4ab
1H NMR (600 MHz, CDCl3)
1.89
1.90
0.93
1.90
2.00
0.91
1.95
1.94
9.0
8.0
7.0
6.0
5.0
4.0
3.0
2.0
1.0
0
7.975
7.962
7.960
7.577
7.574
7.572
7.562
7.550
7.548
7.473
7.460
7.450
7.447
7.322
7.309
7.300
7.297
7.291
7.285
7.270
7.260
7.228
7.216
7.204
3.327
3.314
3.302
3.094
3.082
3.069
X : parts per Million : Proton

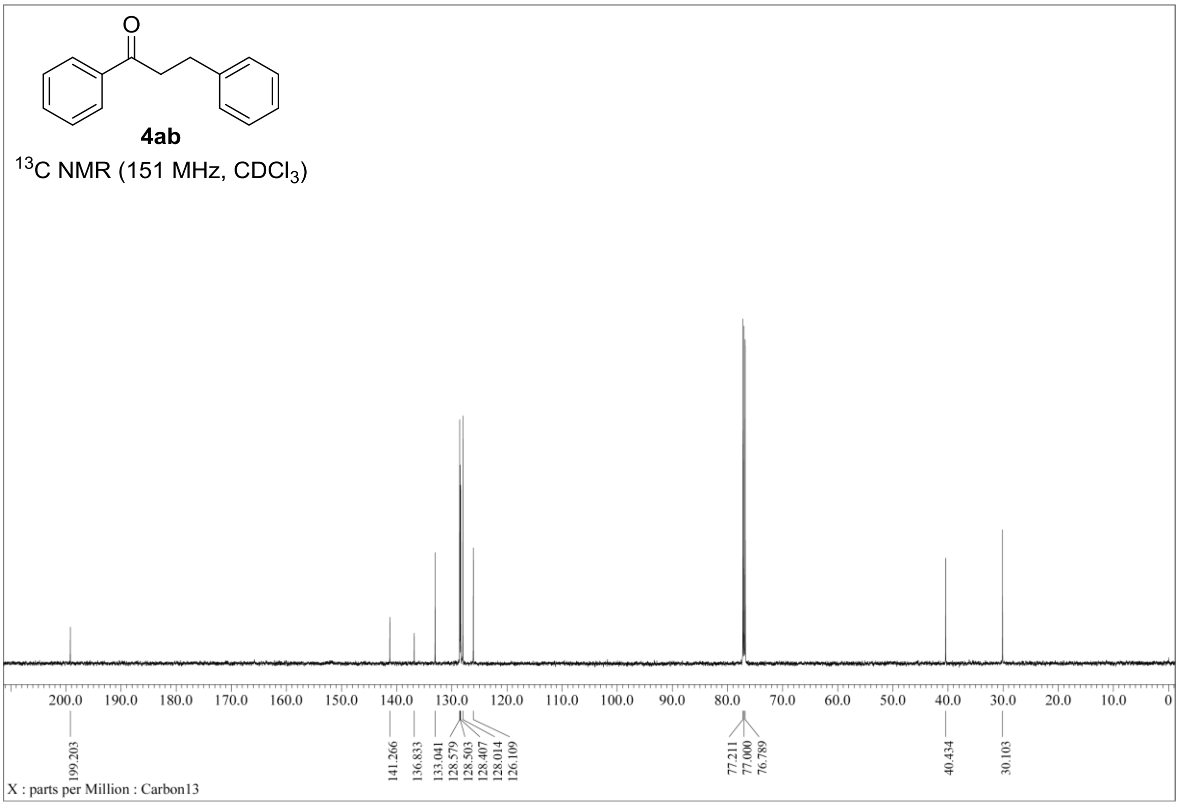
O
4ab
13C NMR (151 MHz, CDCl3)
200.0
190.0
180.0
170.0
160.0
150.0
140.0
130.0
120.0
110.0
100.0
90.0
80.0
70.0
60.0
50.0
40.0
30.0
20.0
10.0
0
199.203
141.266
136.833
133.041
128.579
128.503
128.407
128.014
126.109
77.211
77.000
76.789
40.434
30.103
X : parts per Million : Carbon13

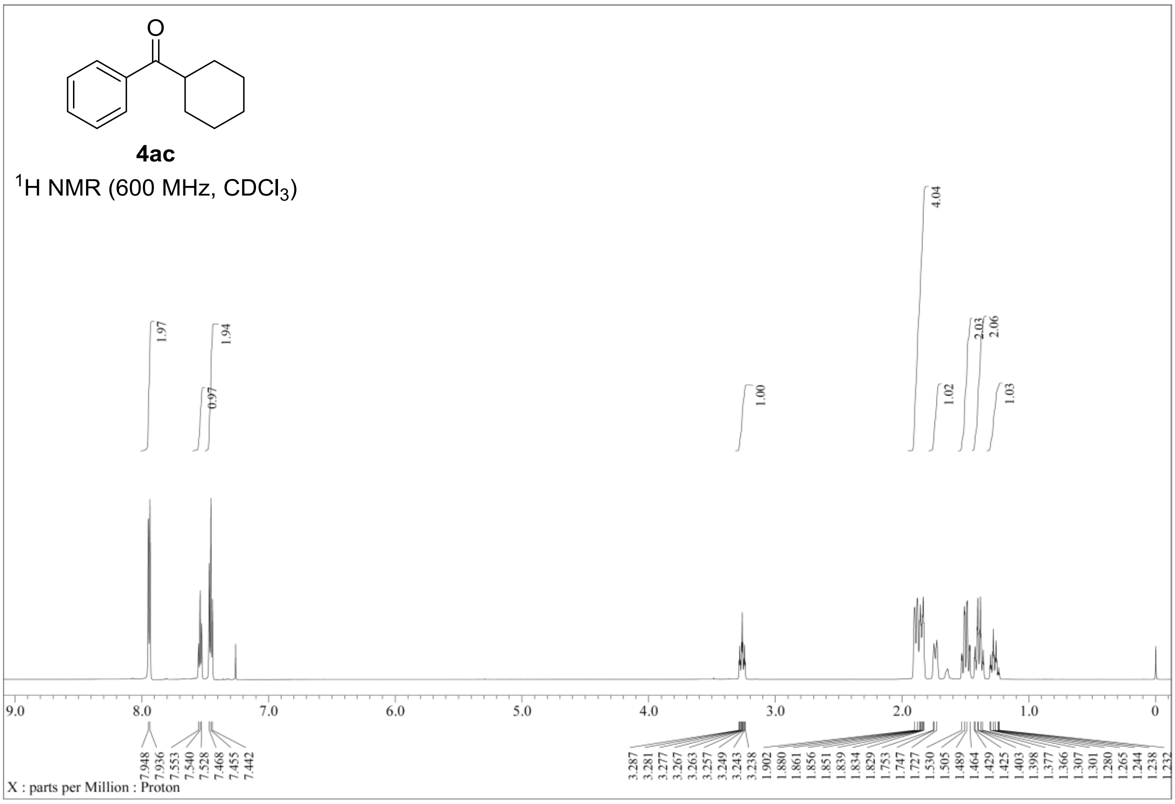
O
4ac
1H NMR (600 MHz, CDCl3)
1.97
0.97
1.94
1.00
4.04
1.02
2.03
2.06
1.03
9.0 8.0 7.0 6.0 5.0 4.0 3.0 2.0 1.0 0
7.948 7.936 7.553 7.540 7.528 7.468 7.455 7.442
3.287 3.281 3.277 3.267 3.263 3.257 3.249 3.243 3.238 1.902 1.880 1.861 1.856 1.851 1.839 1.834 1.829 1.753 1.747 1.727 1.530 1.505 1.489 1.464 1.429 1.425 1.403 1.398 1.377 1.366 1.307 1.301 1.280 1.265 1.244 1.238 1.232
X : parts per Million : Proton

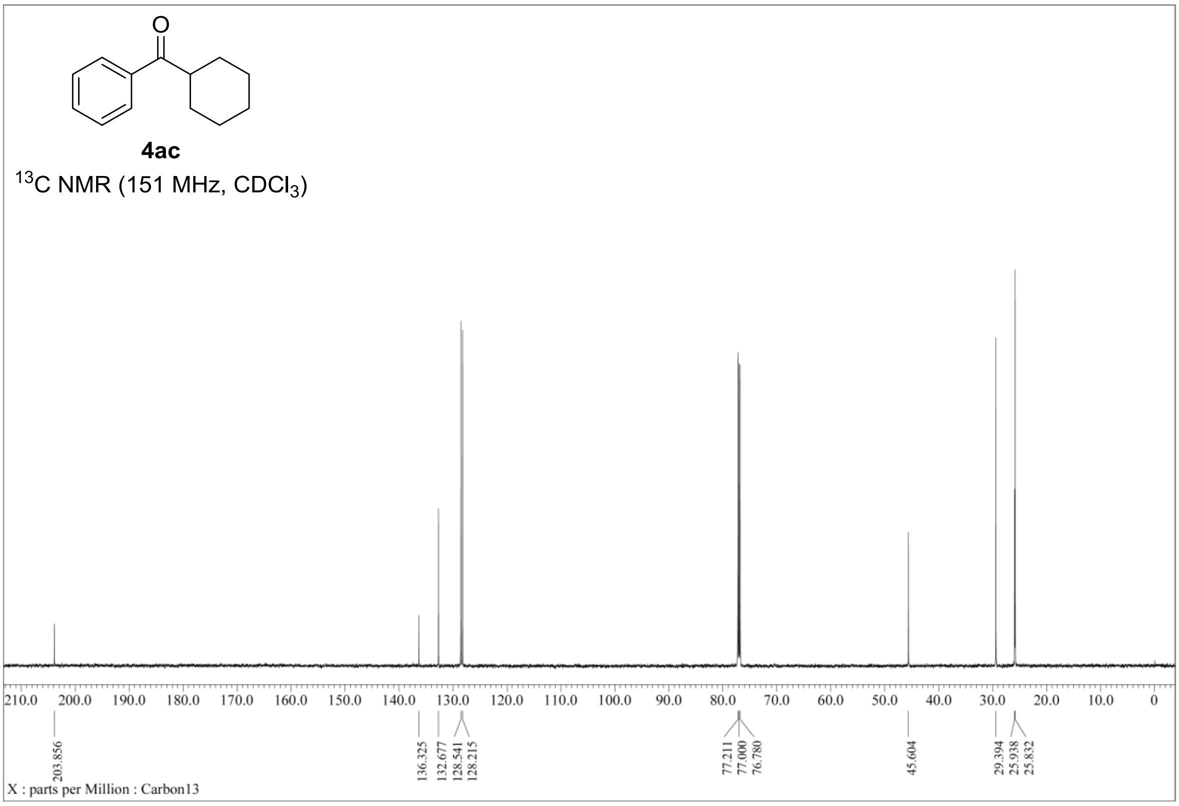
O
4ac
13C NMR (151 MHz, CDCl3)
210.0 200.0 190.0 180.0 170.0 160.0 150.0 140.0 130.0 120.0 110.0 100.0 90.0 80.0 70.0 60.0 50.0 40.0 30.0 20.0 10.0 0
203.856
136.325 132.677 128.541 128.215
77.211 77.000 76.780
45.604
29.394 25.938 25.832
X : parts per Million : Carbon13

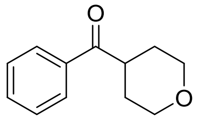

4ad

^{1}H NMR (600 MHz, $CDCl_3$)

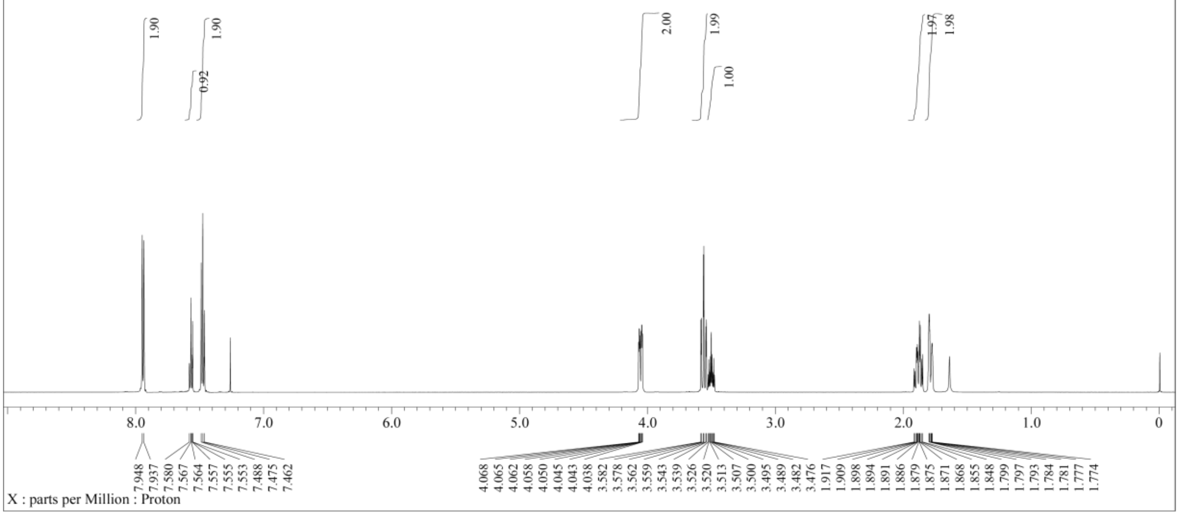

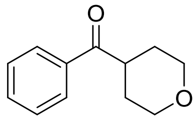

4ad

^{13}C NMR (151 MHz, $CDCl_3$)

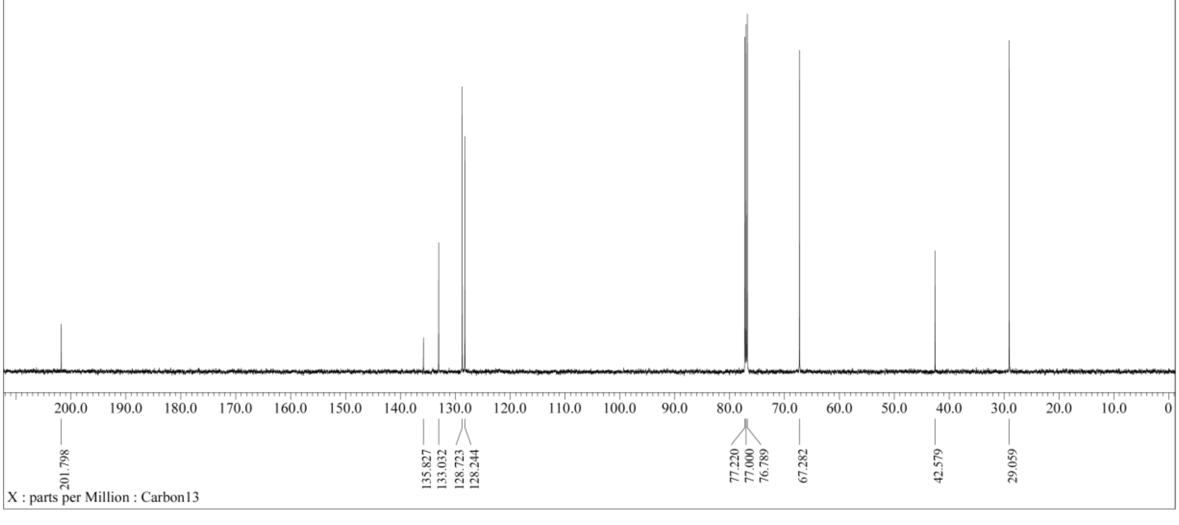

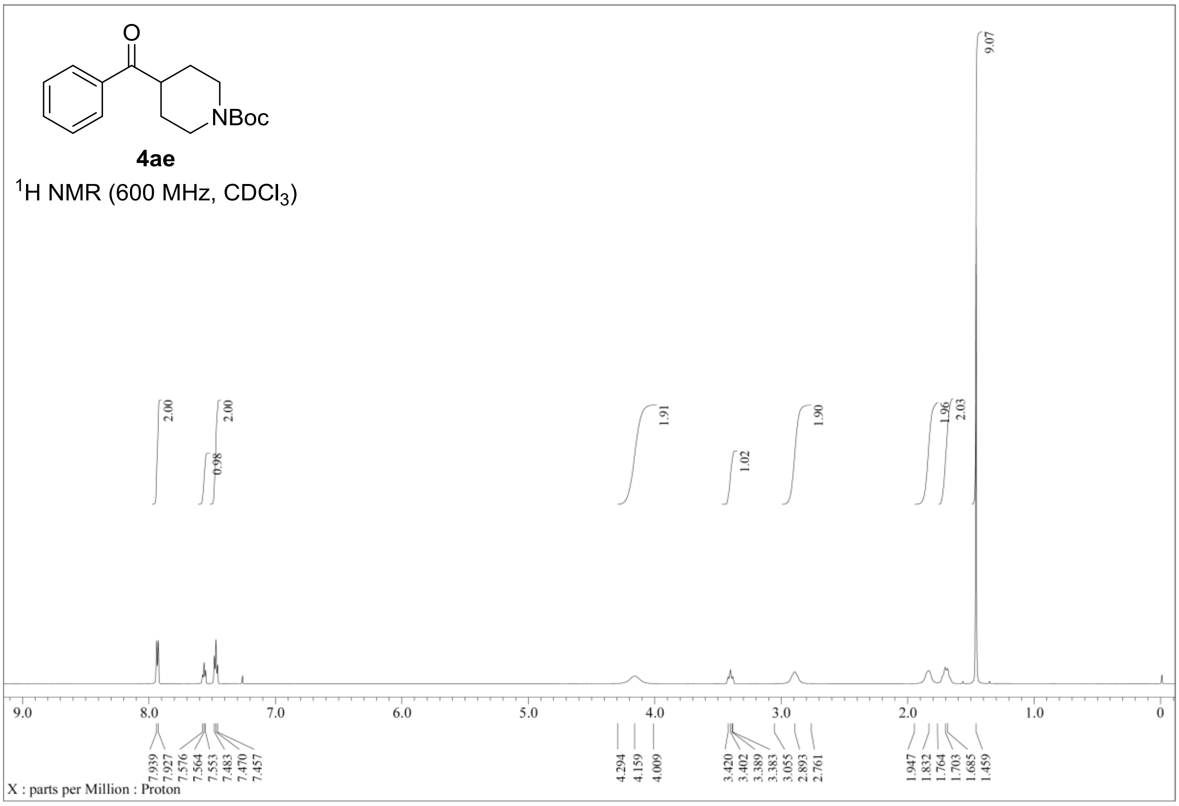
O
NBoc
4ae
^{1}H NMR (600 MHz, CDCl$_3$)
2.00
0.98
2.00
1.91
1.02
1.90
1.96
2.03
9.07
9.0
8.0
7.0
6.0
5.0
4.0
3.0
2.0
1.0
0
7.939
7.927
7.576
7.564
7.553
7.483
7.470
7.457
4.294
4.159
4.009
3.420
3.402
3.389
3.383
3.055
2.893
2.761
1.947
1.832
1.764
1.703
1.685
1.459
X : parts per Million : Proton

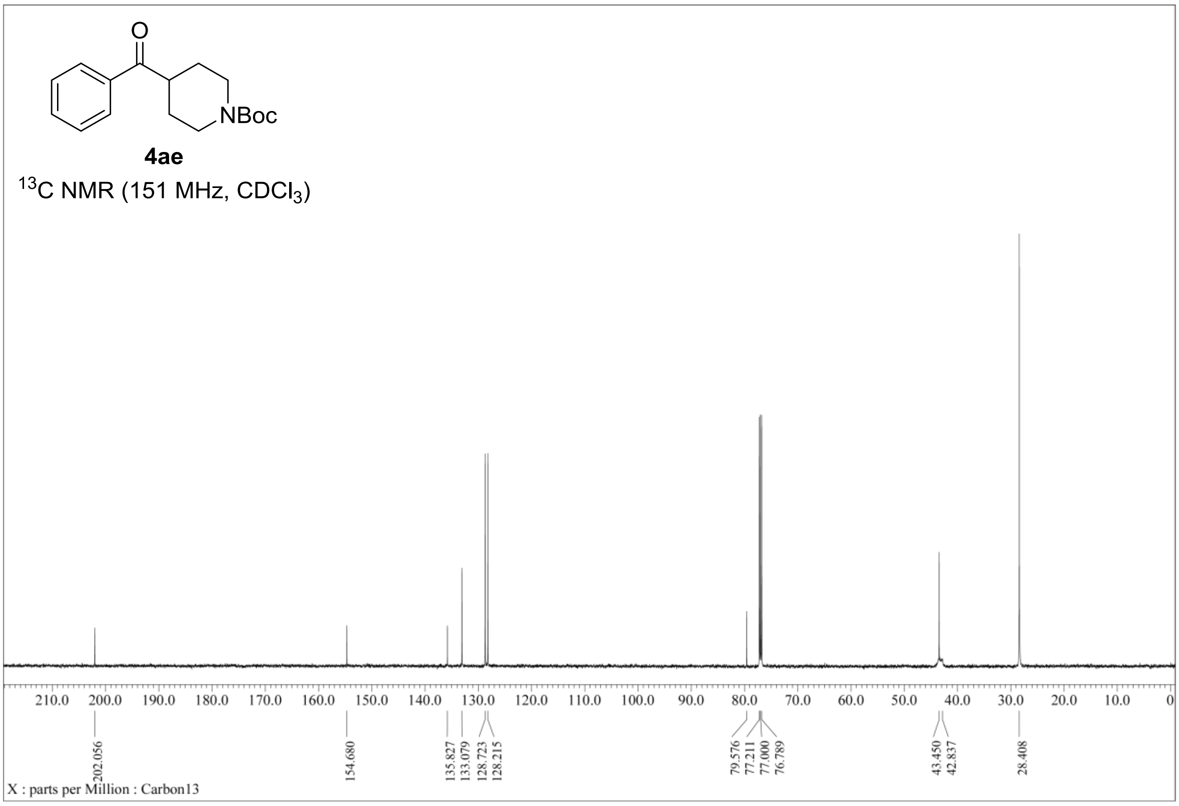
O
NBoc
4ae
^{13}C NMR (151 MHz, CDCl$_3$)
210.0
200.0
190.0
180.0
170.0
160.0
150.0
140.0
130.0
120.0
110.0
100.0
90.0
80.0
70.0
60.0
50.0
40.0
30.0
20.0
10.0
0
202.056
154.680
135.827
133.079
128.723
128.215
79.576
77.211
77.000
76.789
43.450
42.837
28.408
X : parts per Million : Carbon13

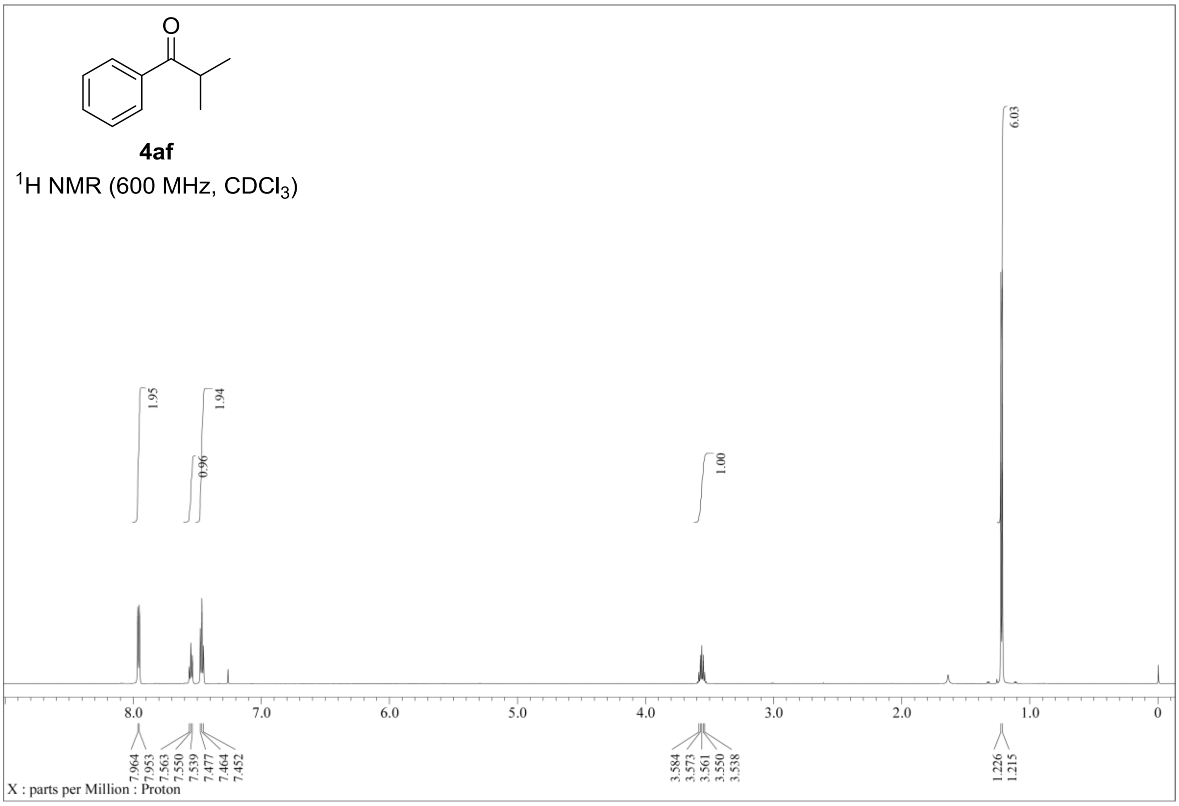
4af
^1H NMR (600 MHz, $CDCl_3$)
1.95
0.96
1.94
1.00
6.03
7.964 7.953 7.563 7.550 7.539 7.477 7.464 7.452
3.584 3.573 3.561 3.550 3.538
1.226 1.215
X : parts per Million : Proton

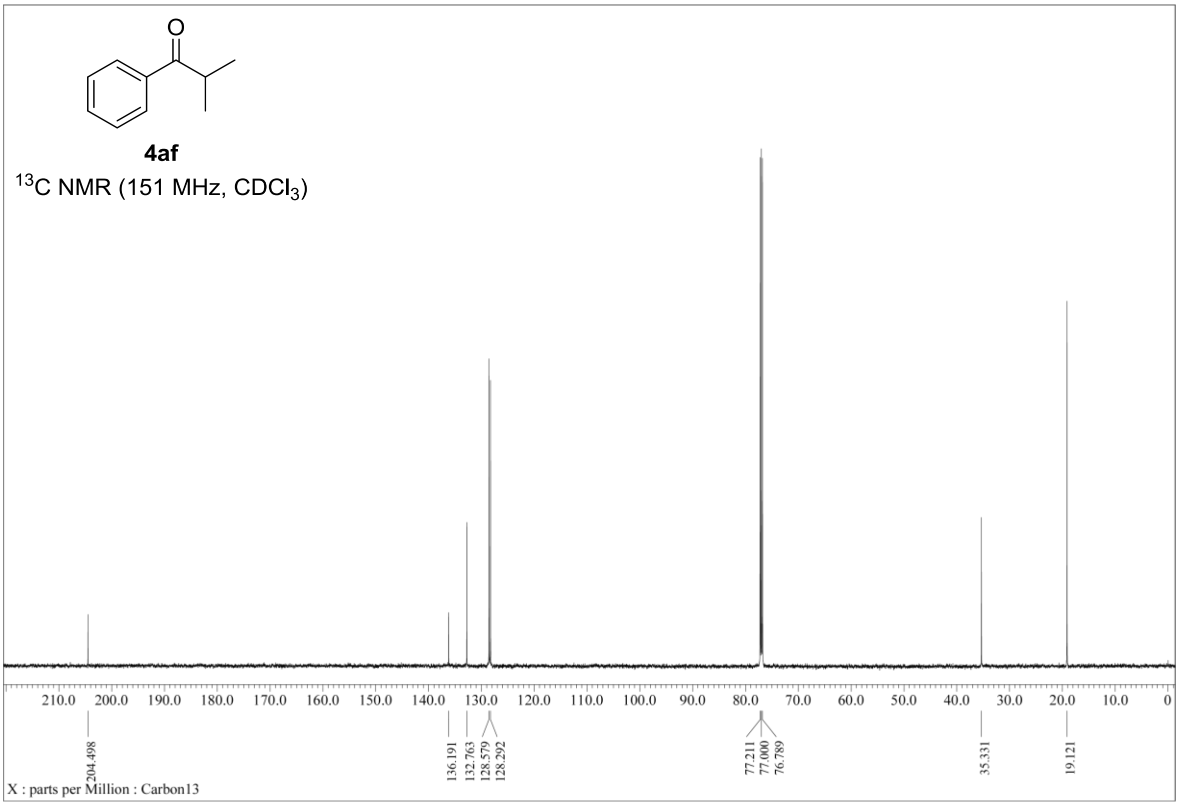
4af
^{13}C NMR (151 MHz, $CDCl_3$)
204.498
136.191 132.763 128.579 128.292
77.211 77.000 76.789
35.331
19.121
X : parts per Million : Carbon13

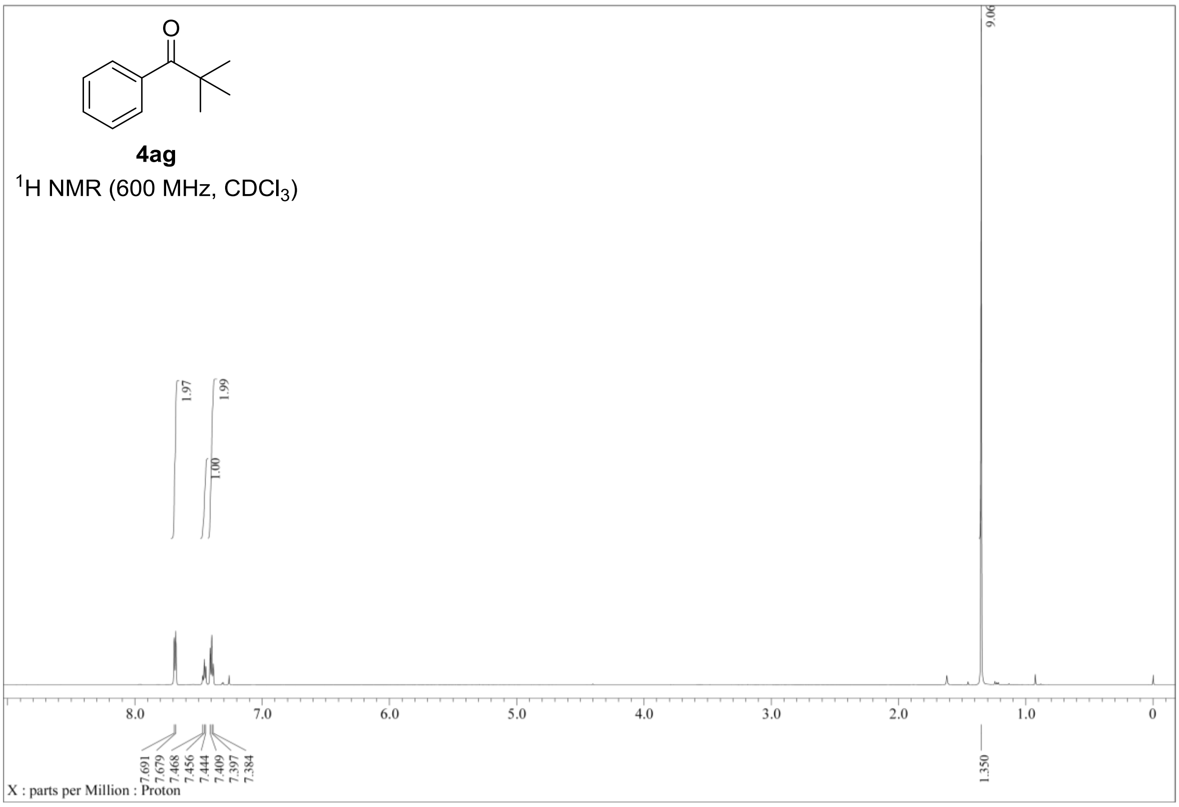
4ag
^{1}H NMR (600 MHz, $CDCl_3$)
1.97
1.00
1.99
9.06
8.0
7.0
6.0
5.0
4.0
3.0
2.0
1.0
0
7.691
7.679
7.468
7.456
7.444
7.409
7.397
7.384
1.350
X : parts per Million : Proton

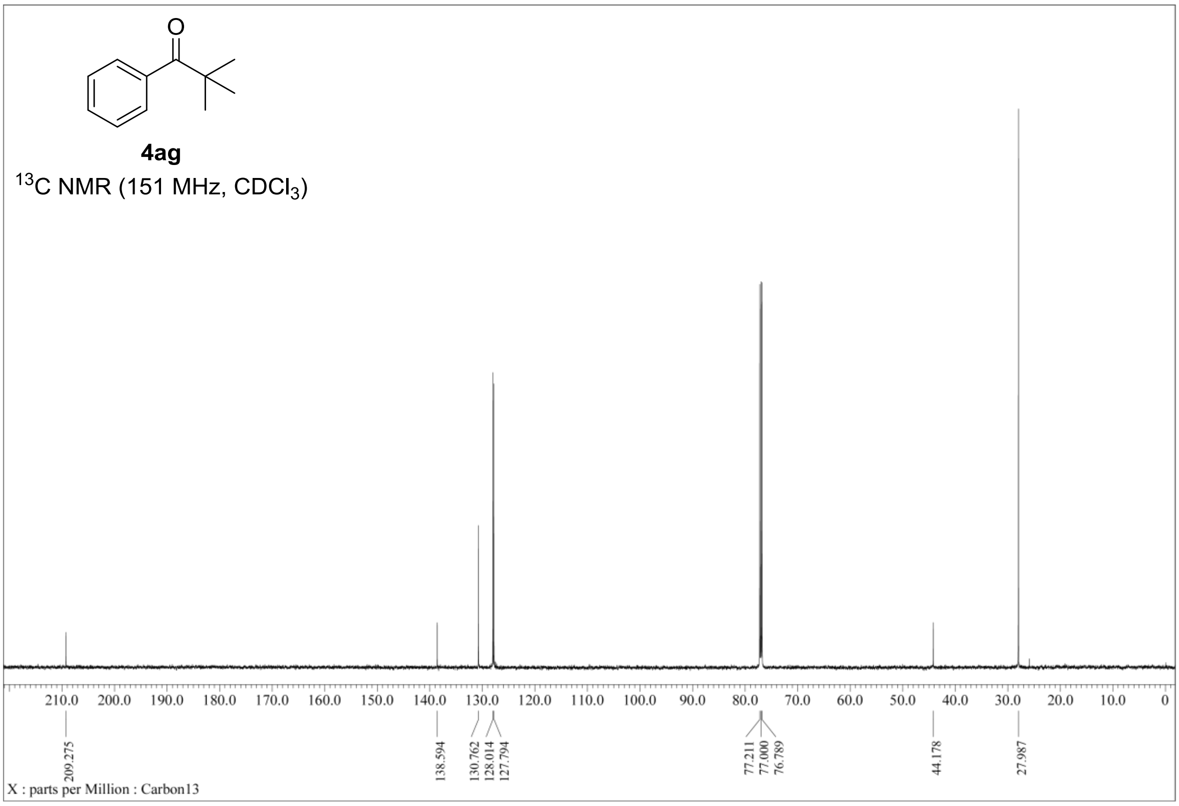
4ag
^{13}C NMR (151 MHz, $CDCl_3$)
210.0
200.0
190.0
180.0
170.0
160.0
150.0
140.0
130.0
120.0
110.0
100.0
90.0
80.0
70.0
60.0
50.0
40.0
30.0
20.0
10.0
0
209.275
138.594
130.762
128.014
127.794
77.211
77.000
76.789
44.178
27.987
X : parts per Million : Carbon13

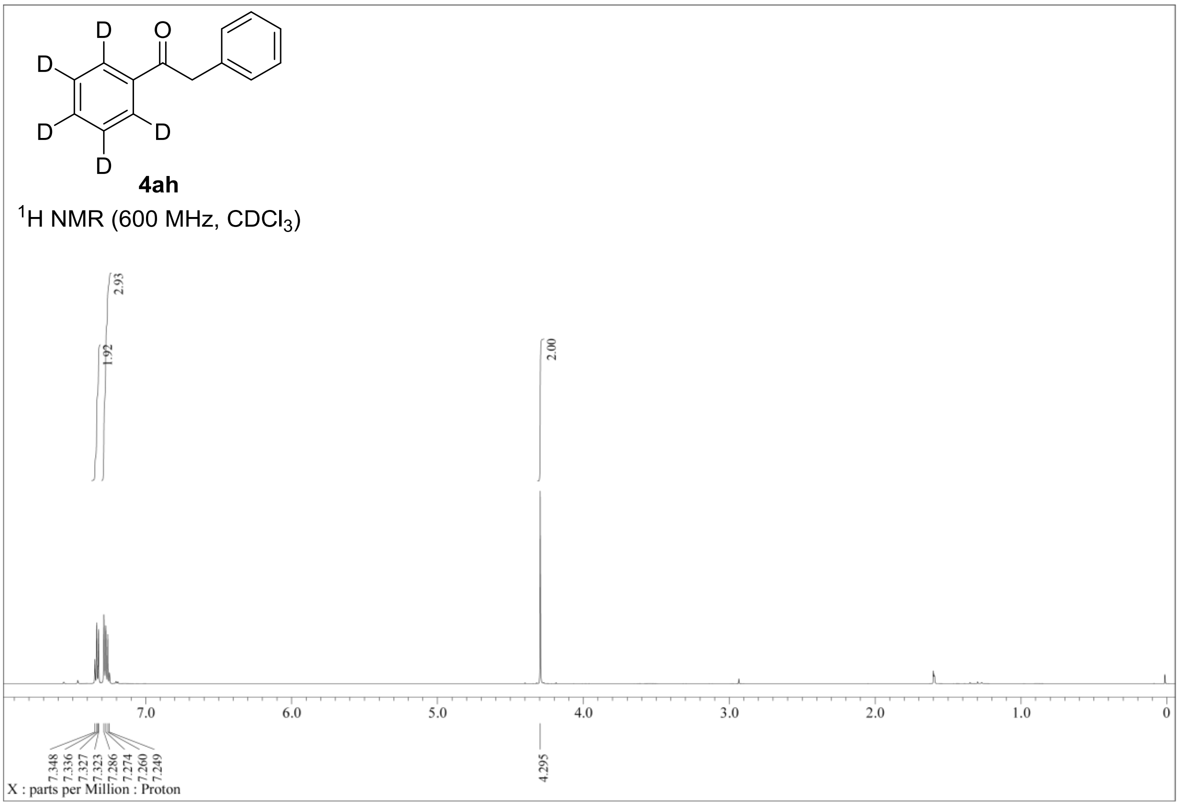
4ah
^{1}H NMR (600 MHz, CDCl$_3$)
1.92
2.93
2.00
7.0
6.0
5.0
4.0
3.0
2.0
1.0
0
7.348 7.336 7.327 7.323 7.286 7.274 7.260 7.249
4.295
X : parts per Million : Proton

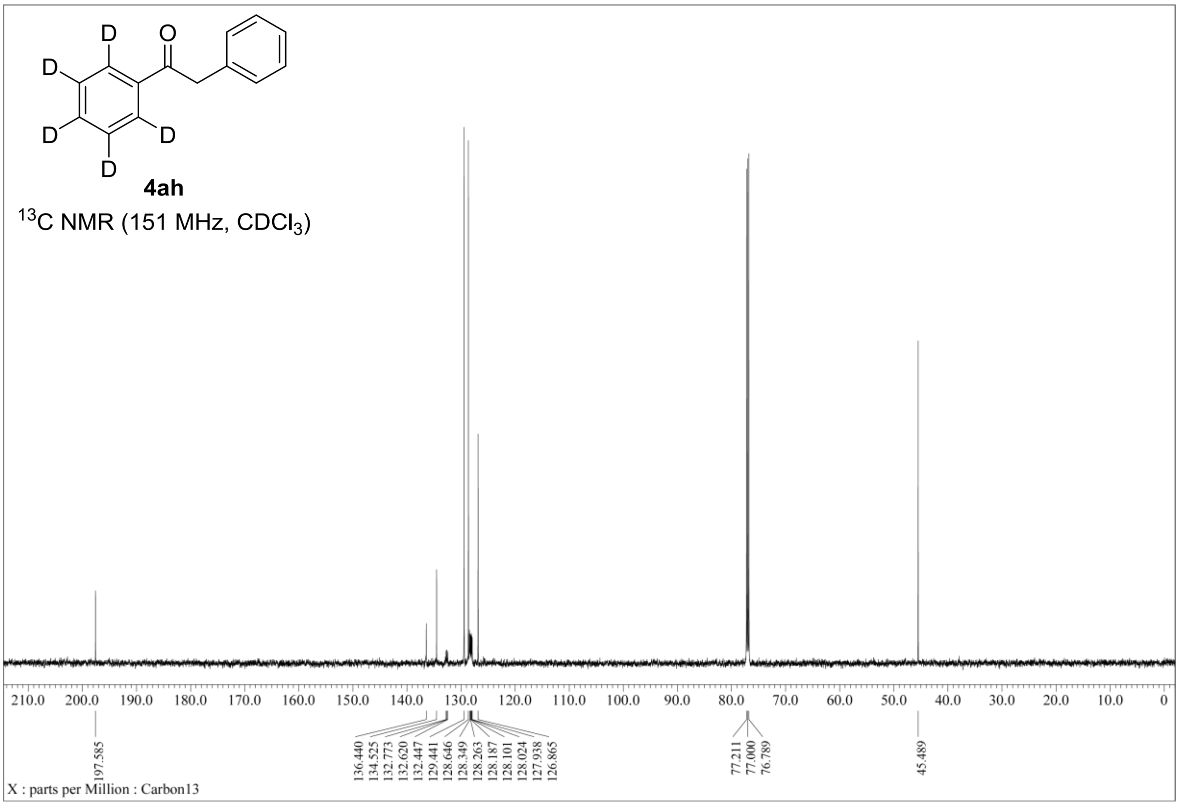
4ah
^{13}C NMR (151 MHz, CDCl$_3$)
210.0 200.0 190.0 180.0 170.0 160.0 150.0 140.0 130.0 120.0 110.0 100.0 90.0 80.0 70.0 60.0 50.0 40.0 30.0 20.0 10.0 0
197.585
136.440 134.525 132.773 132.620 132.447 129.441 128.646 128.349 128.263 128.187 128.101 128.024 127.938 126.865
77.211 77.000 76.789
45.489
X : parts per Million : Carbon13

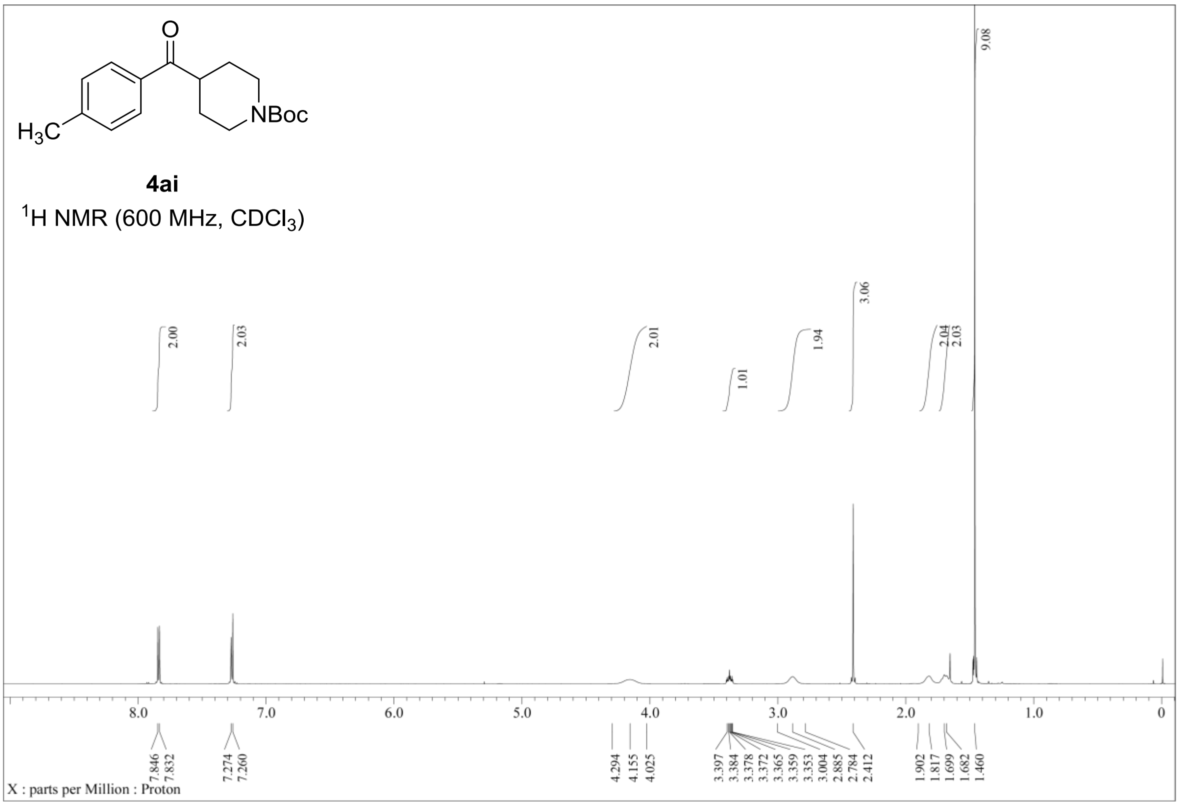
O
NBoc
H3C
4ai
1H NMR (600 MHz, CDCl3)
X : parts per Million : Proton

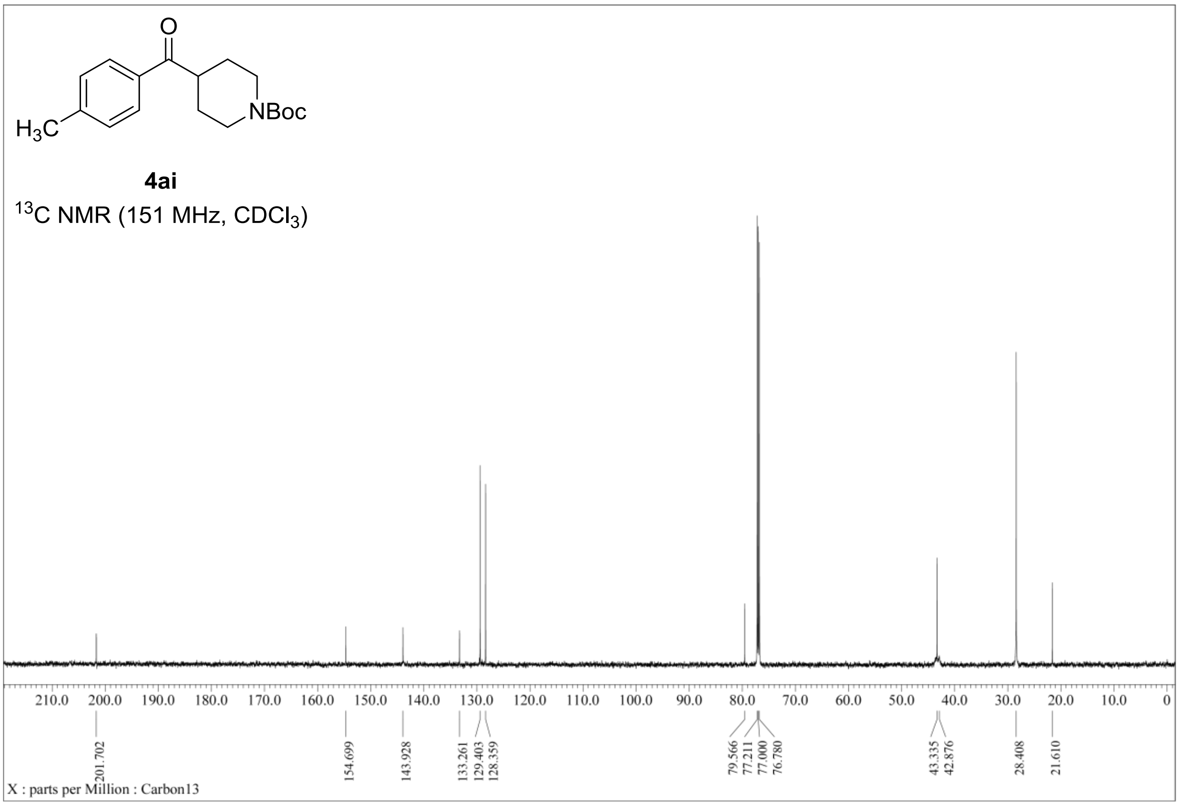
O
NBoc
H3C
4ai
13C NMR (151 MHz, CDCl3)
X : parts per Million : Carbon13

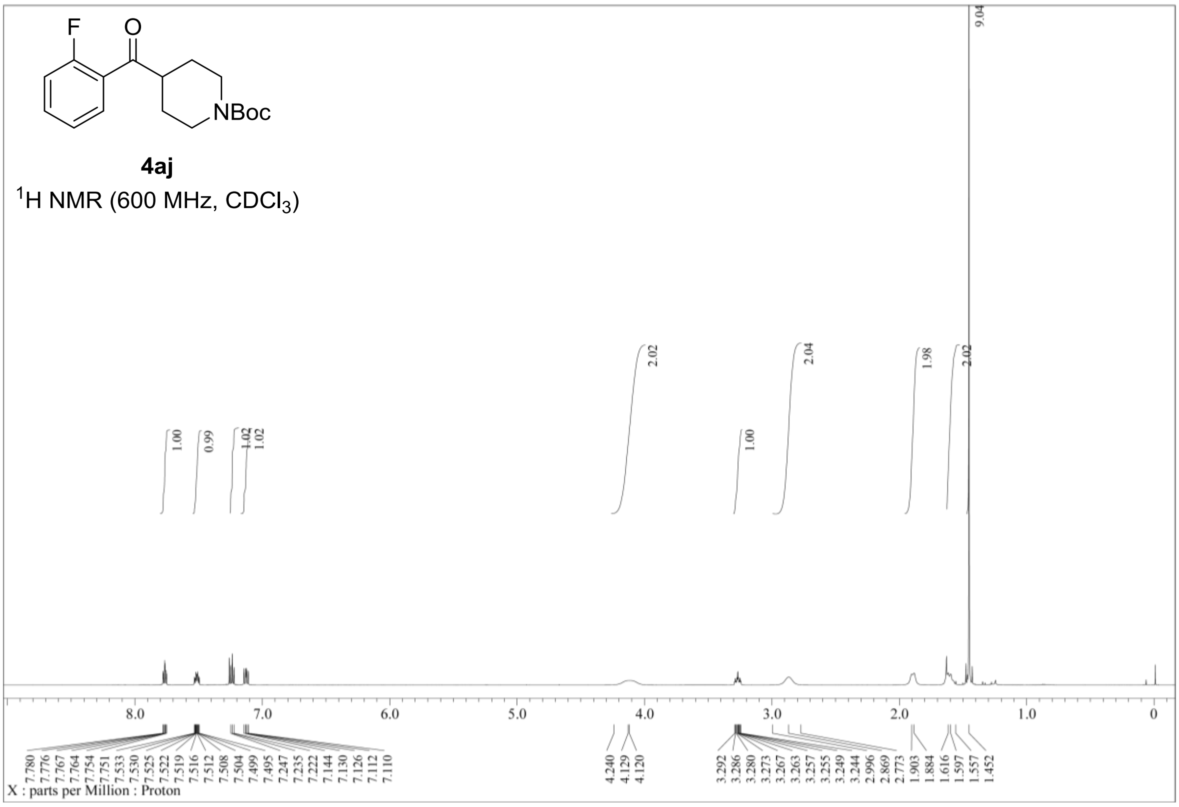
F
O
NBoc
4aj
1H NMR (600 MHz, CDCl3)
X : parts per Million : Proton

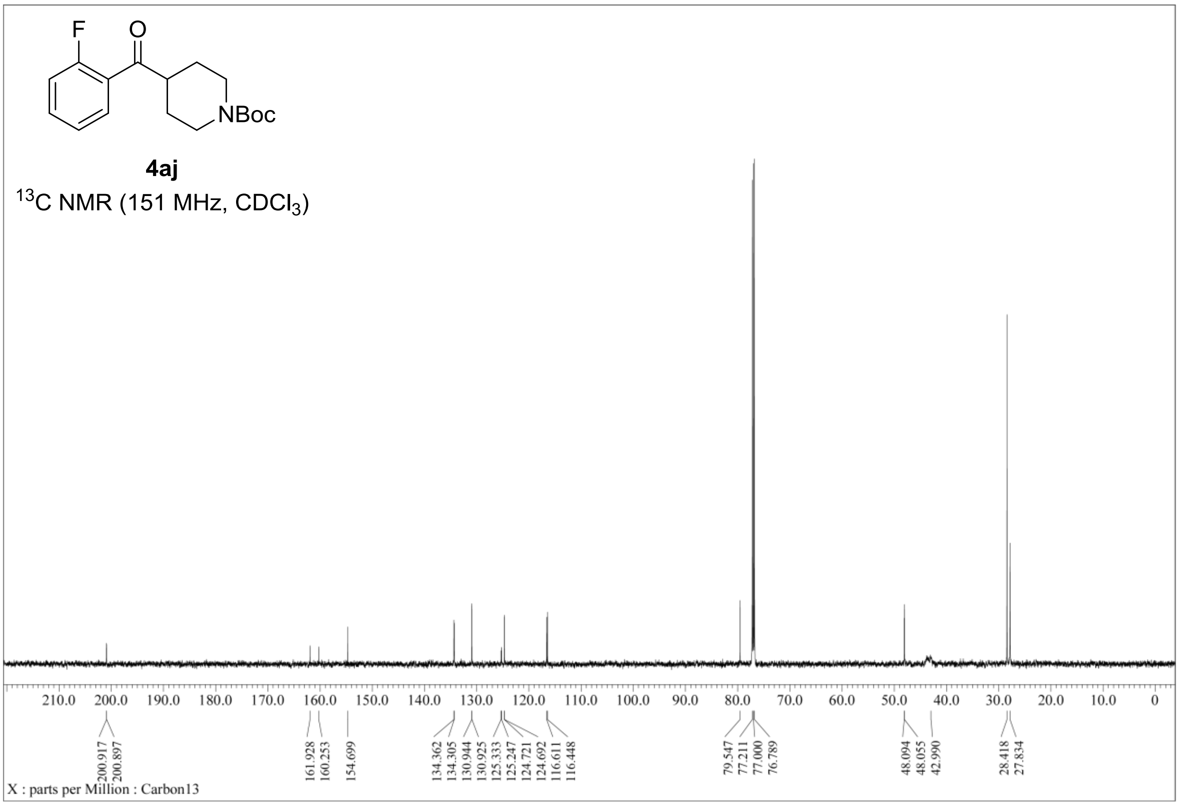
F
O
NBoc
4aj
13C NMR (151 MHz, CDCl3)
X : parts per Million : Carbon13

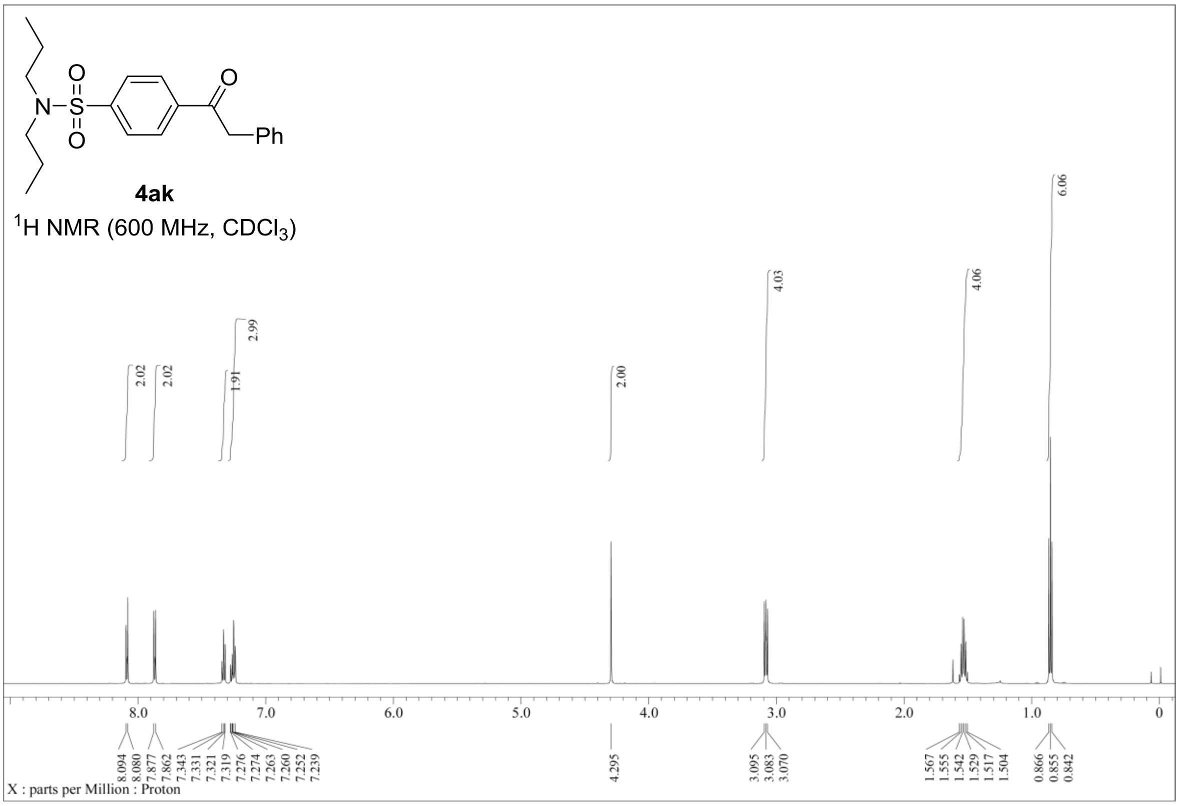

N
S
O
O
O
Ph
4ak
^{1}H NMR (600 MHz, CDCl$_3$)
X : parts per Million : Proton
8.094
8.080
7.877
7.862
7.343
7.331
7.321
7.319
7.276
7.274
7.263
7.260
7.252
7.239
4.295
3.095
3.083
3.070
1.567
1.555
1.542
1.529
1.517
1.504
0.866
0.855
0.842
2.02
2.02
1.91
2.99
2.00
4.03
4.06
6.06

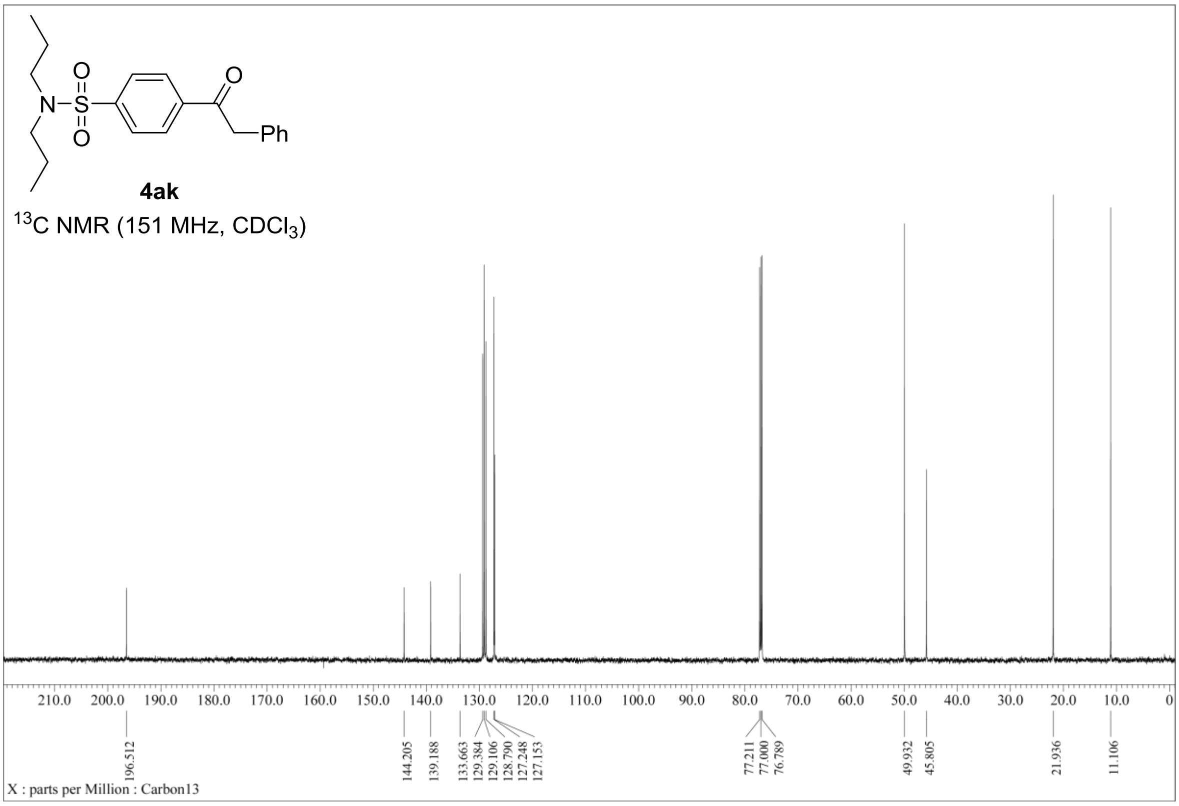

N
S
O
O
O
Ph
4ak
^{13}C NMR (151 MHz, CDCl$_3$)
X : parts per Million : Carbon13
196.512
144.205
139.188
133.663
129.384
129.106
128.790
127.248
127.153
77.211
77.000
76.789
49.932
45.805
21.936
11.106

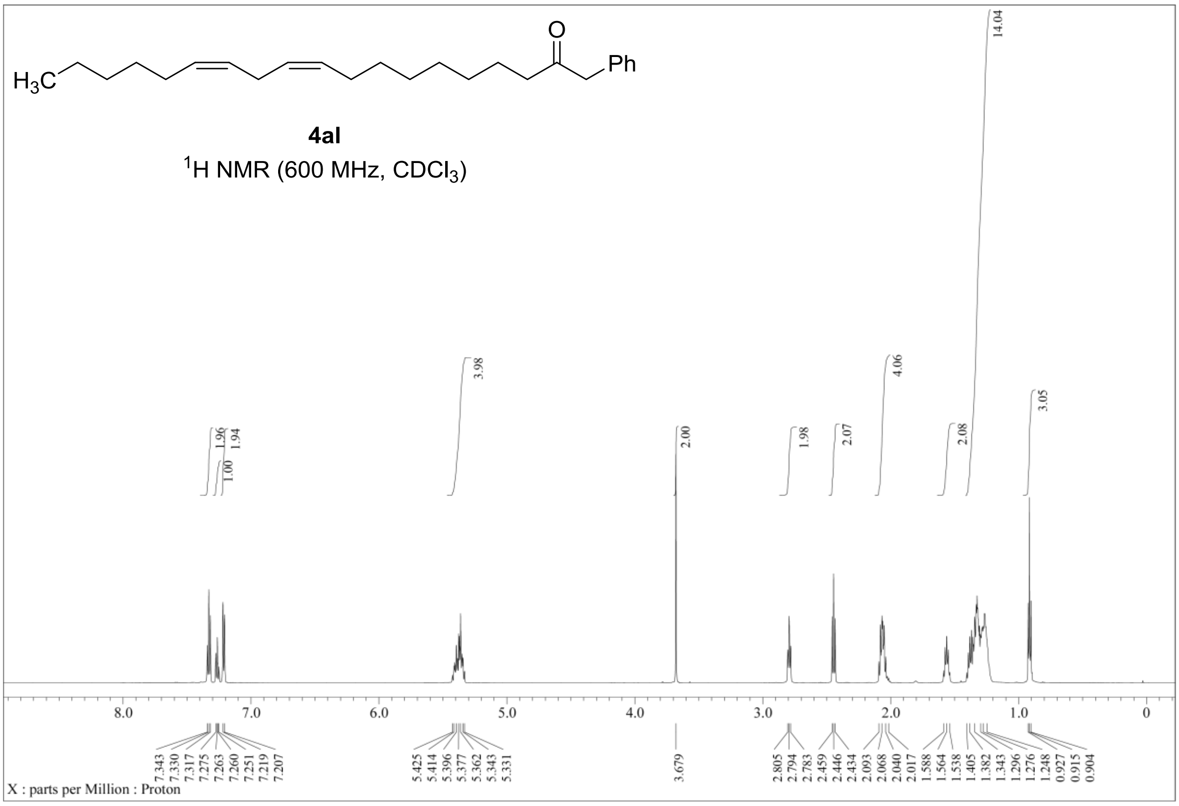
O
Ph
H_3C
4al
^{1}H NMR (600 MHz, $CDCl_3$)
X : parts per Million : Proton

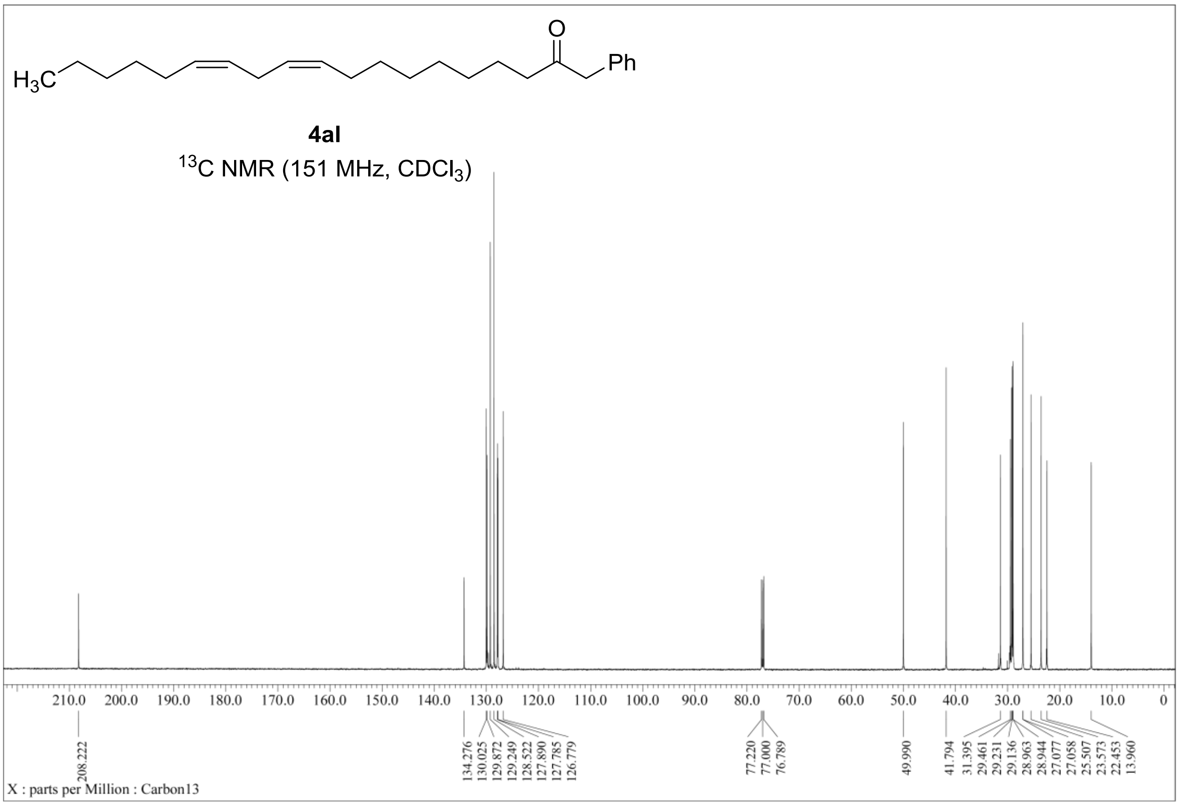
O
Ph
H_3C
4al
^{13}C NMR (151 MHz, $CDCl_3$)
X : parts per Million : Carbon13

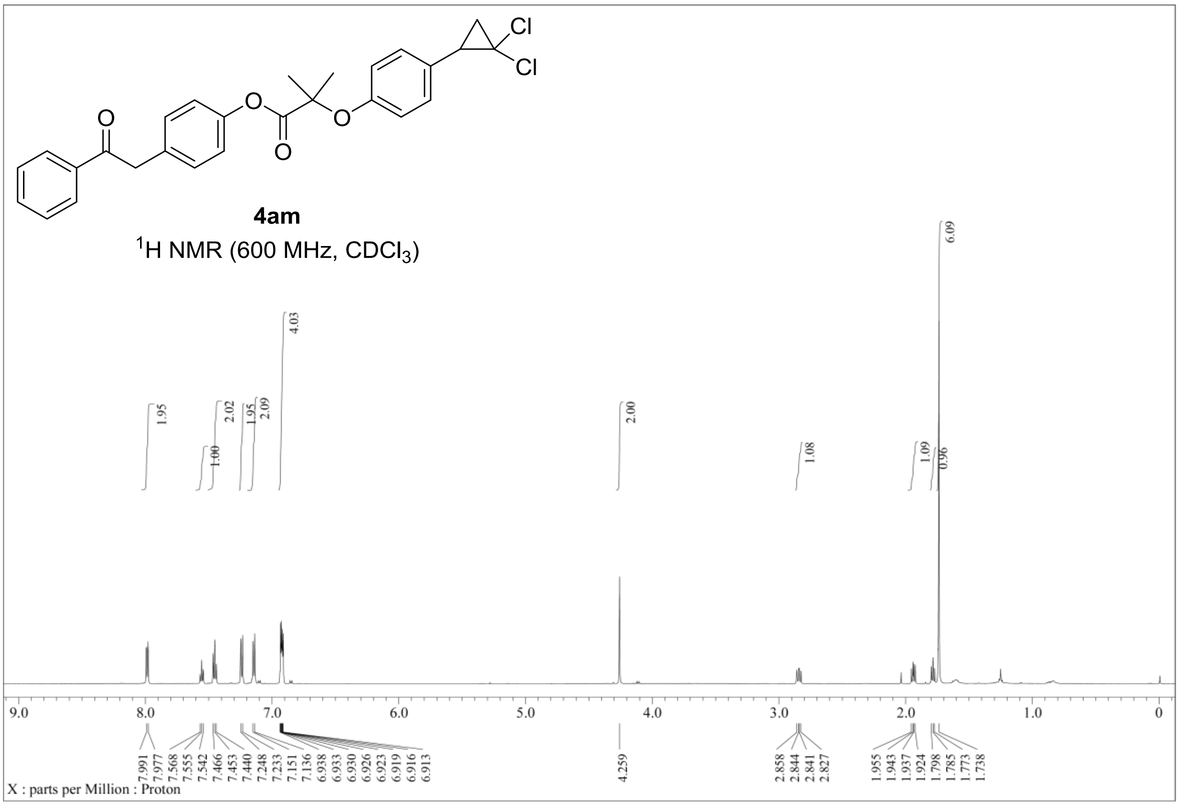
4am
^{1}H NMR (600 MHz, CDCl$_3$)
X : parts per Million : Proton

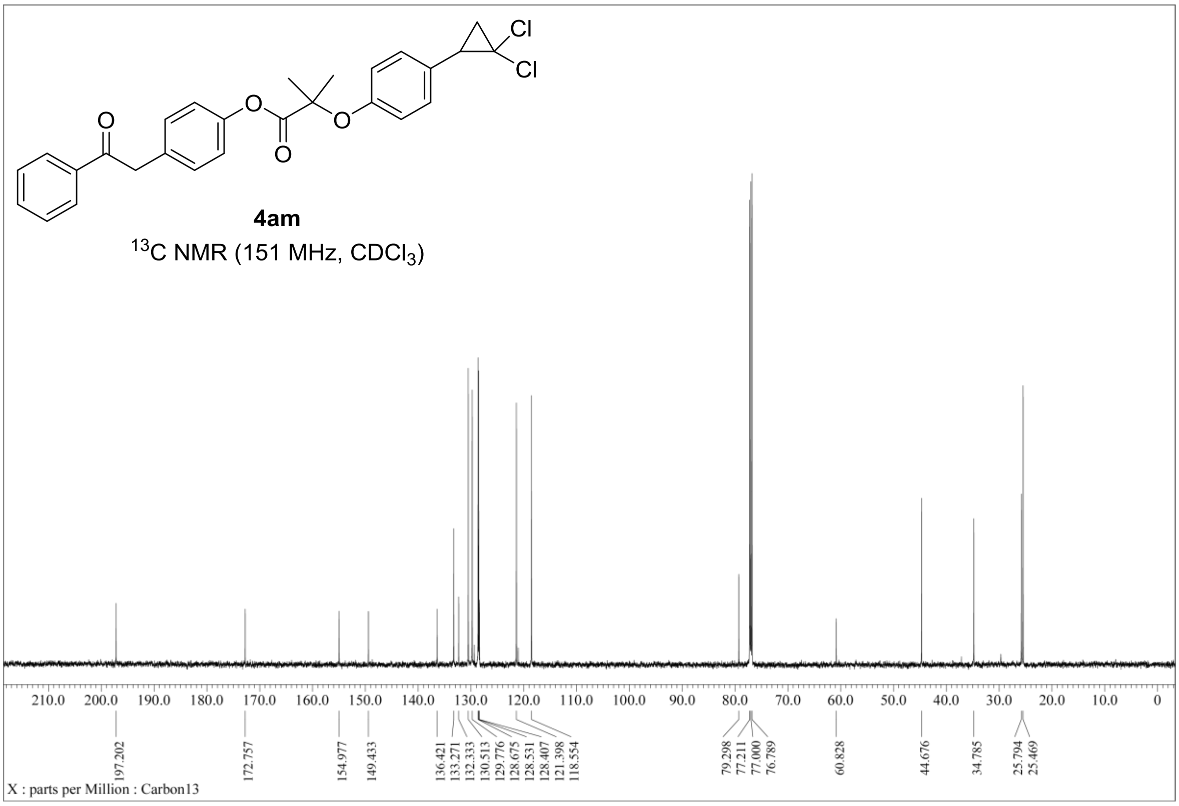
4am
^{13}C NMR (151 MHz, CDCl$_3$)
X : parts per Million : Carbon13

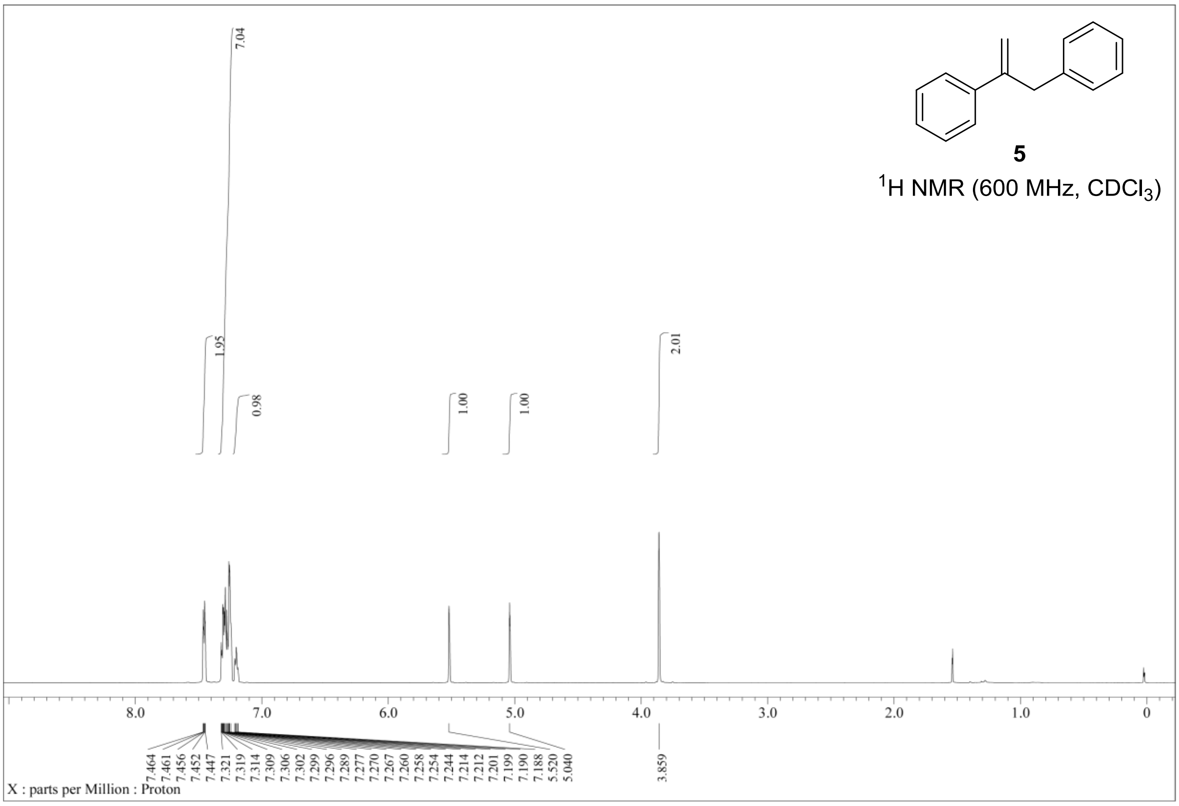
5
1H NMR (600 MHz, CDCl3)
7.04
1.95
0.98
1.00
1.00
2.01
8.0
7.0
6.0
5.0
4.0
3.0
2.0
1.0
0
7.464 7.461 7.456 7.452 7.447 7.321 7.319 7.314 7.309 7.306 7.302 7.299 7.296 7.289 7.277 7.270 7.267 7.260 7.258 7.254 7.244 7.214 7.212 7.201 7.199 7.190 7.188 5.520 5.040
3.859
X : parts per Million : Proton

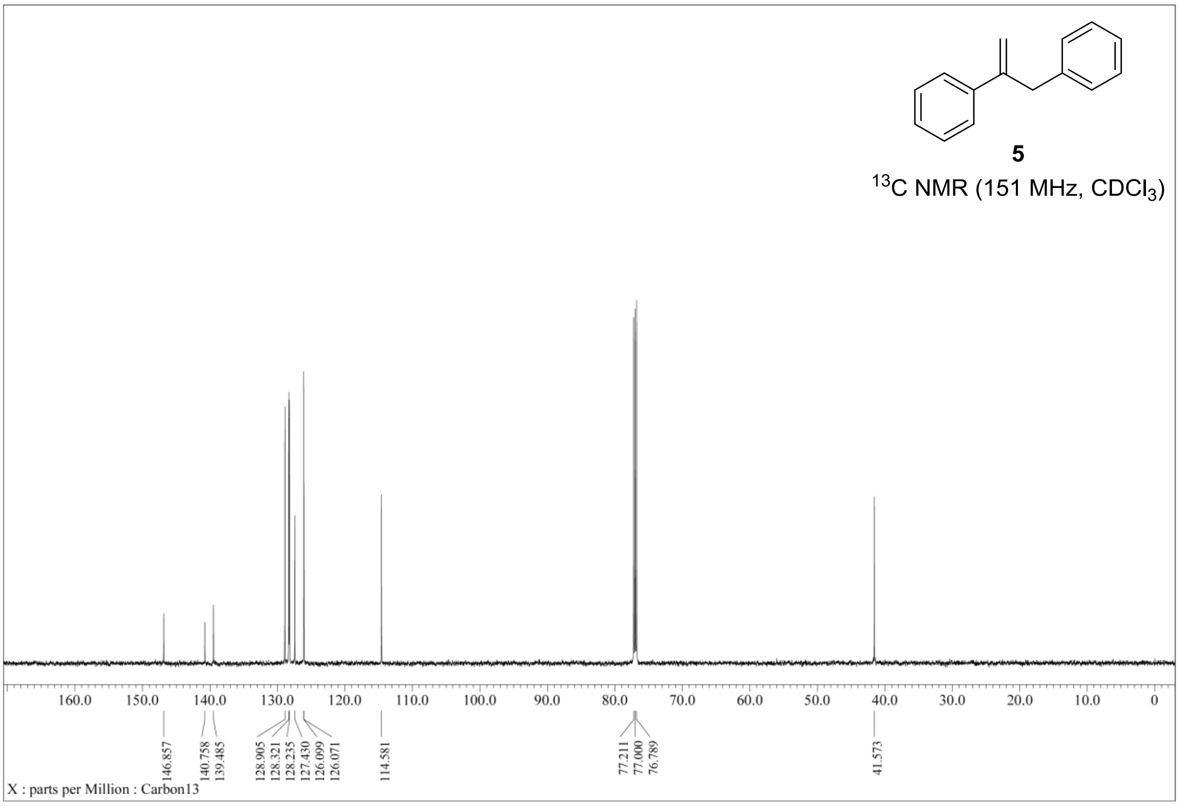
5
13C NMR (151 MHz, CDCl3)
160.0
150.0
140.0
130.0
120.0
110.0
100.0
90.0
80.0
70.0
60.0
50.0
40.0
30.0
20.0
10.0
0
146.857
140.758 139.485
128.905 128.321 128.235 127.430 126.099 126.071
114.581
77.211 77.000 76.789
41.573
X : parts per Million : Carbon13

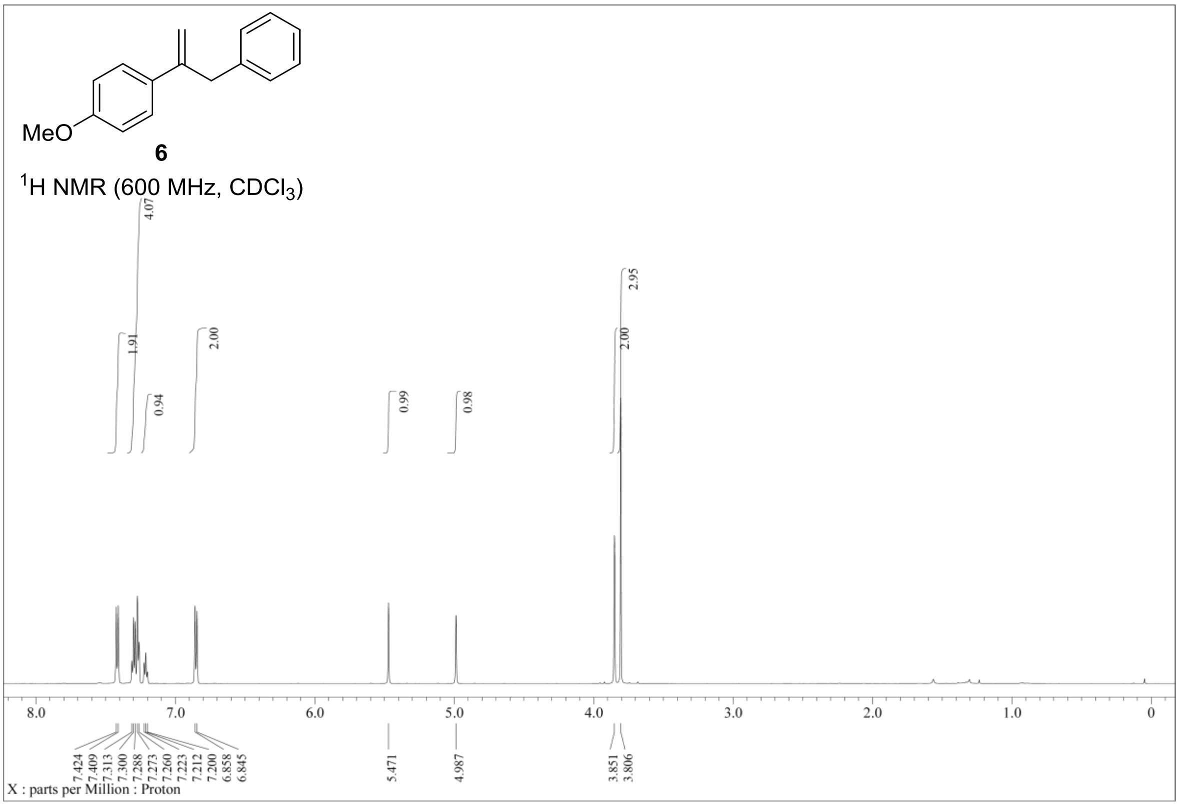

MeO
6
^{1}H NMR (600 MHz, CDCl$_3$)
4.07
1.91
0.94
2.00
0.99
0.98
2.95
2.00
8.0
7.0
6.0
5.0
4.0
3.0
2.0
1.0
0
7.424
7.409
7.313
7.300
7.288
7.273
7.260
7.223
7.212
7.200
6.858
6.845
5.471
4.987
3.851
3.806
X : parts per Million : Proton

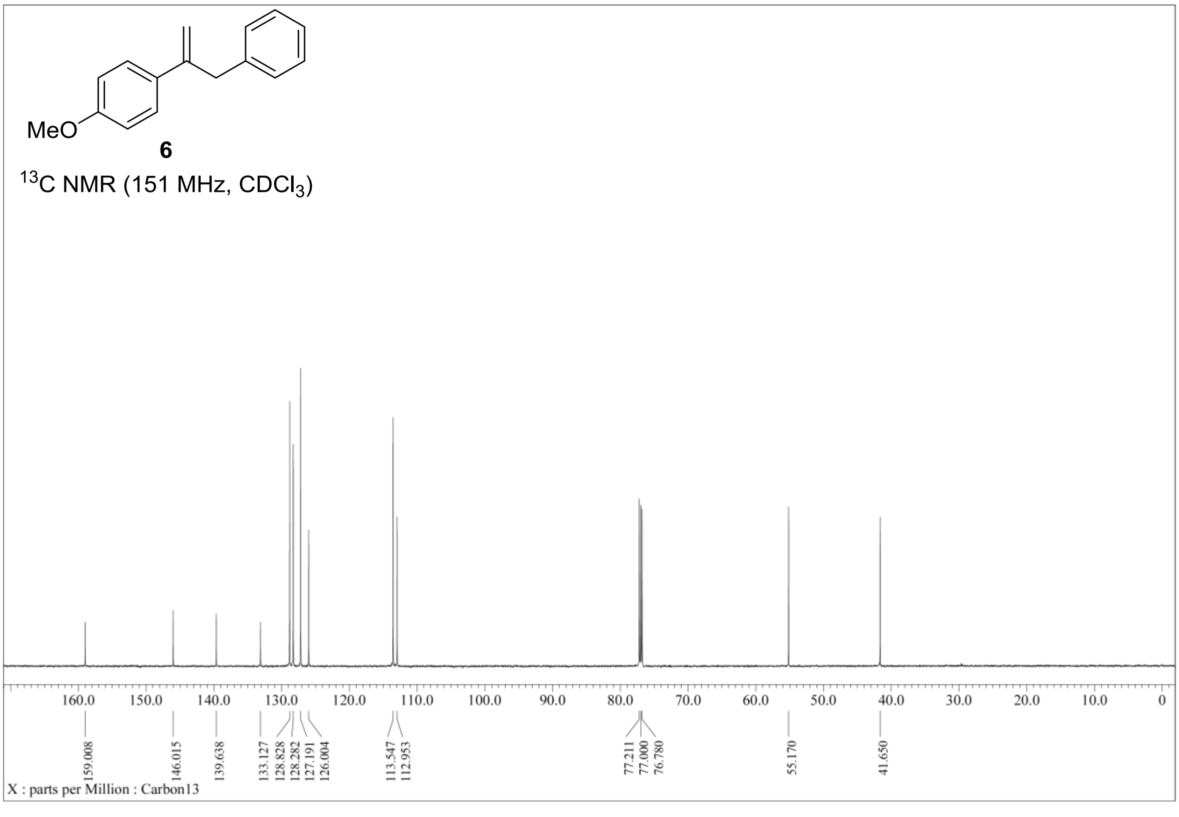

MeO
6
^{13}C NMR (151 MHz, CDCl$_3$)
160.0
150.0
140.0
130.0
120.0
110.0
100.0
90.0
80.0
70.0
60.0
50.0
40.0
30.0
20.0
10.0
0
159.008
146.015
139.638
133.127
128.828
128.282
127.191
126.004
113.547
112.953
77.211
77.000
76.780
55.170
41.650
X : parts per Million : Carbon13

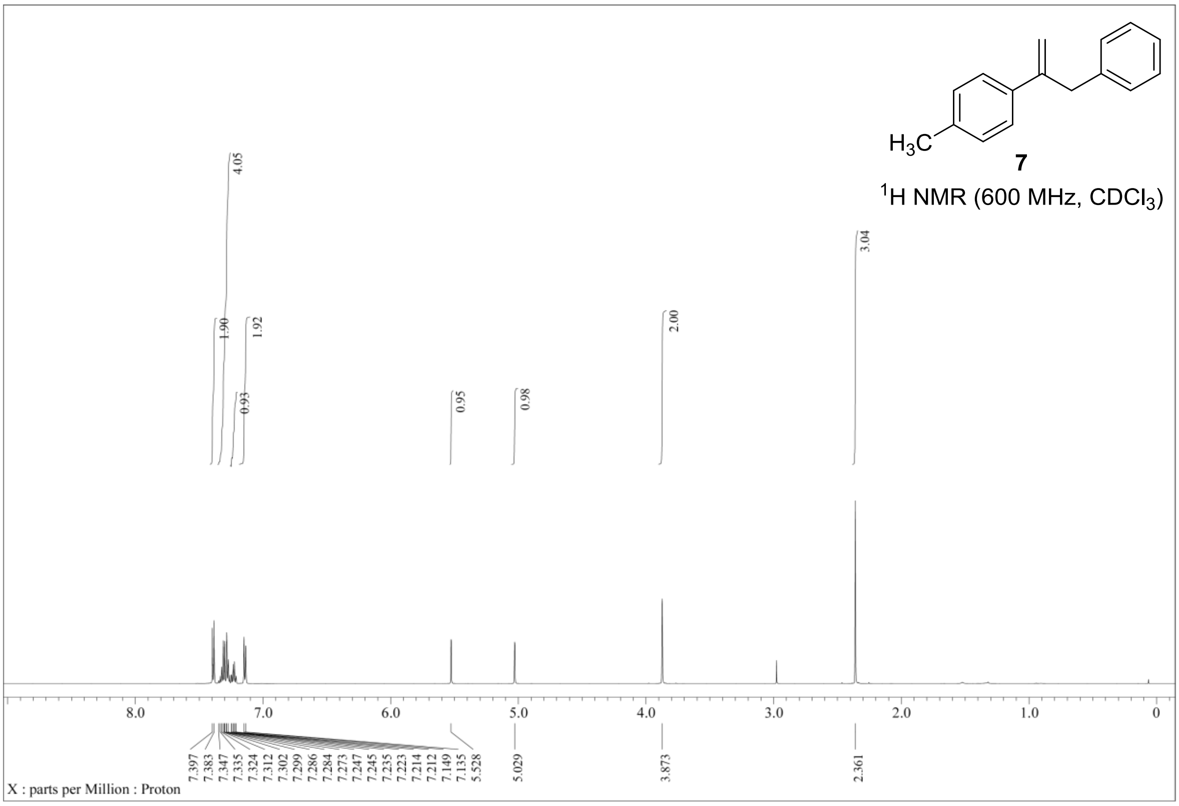
H3C
7
1H NMR (600 MHz, CDCl3)
X : parts per Million : Proton

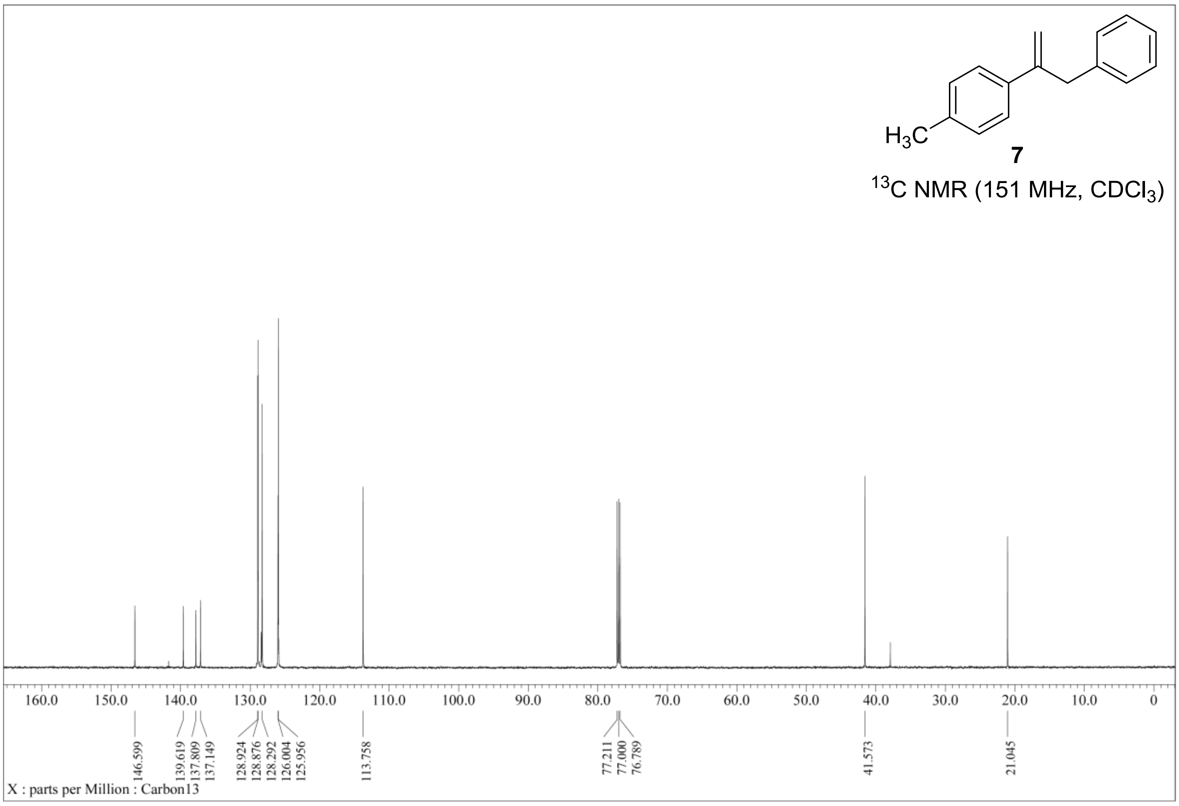
H3C
7
13C NMR (151 MHz, CDCl3)
X : parts per Million : Carbon13

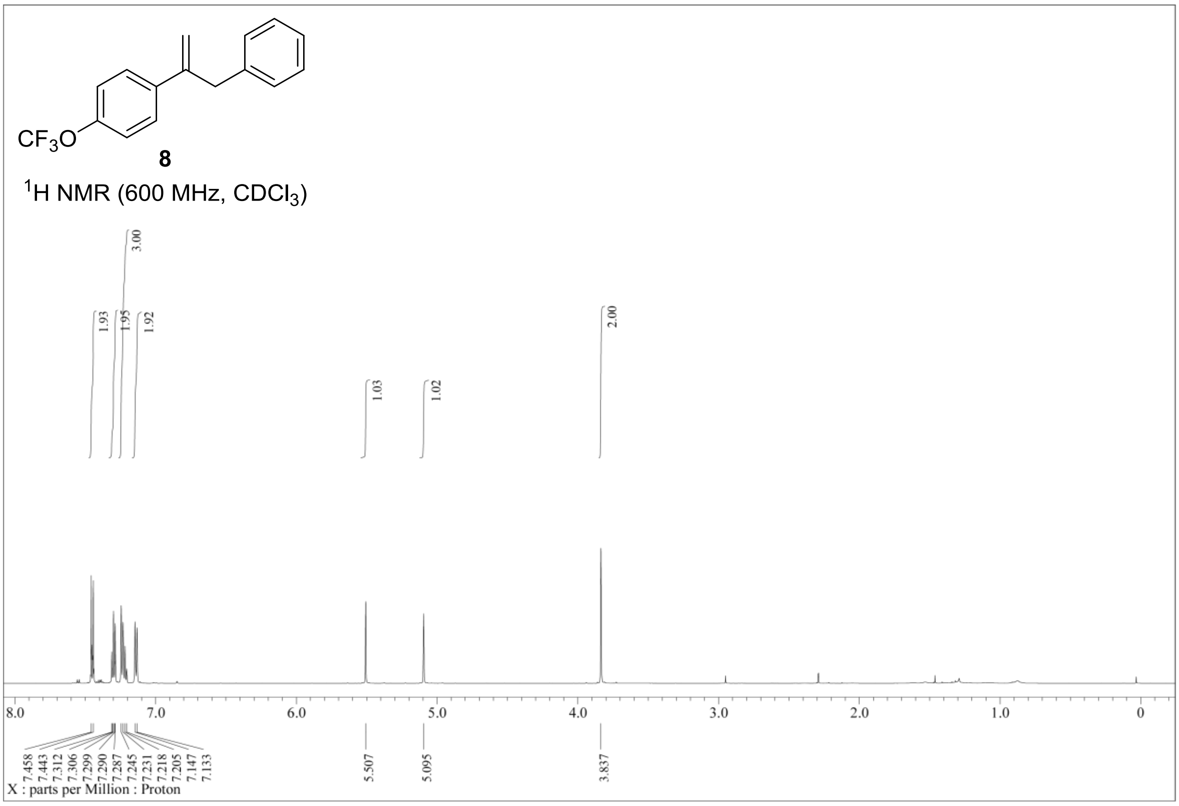
CF3O
8
1H NMR (600 MHz, CDCl3)
1.93
1.95
3.00
1.92
1.03
1.02
2.00
8.0
7.0
6.0
5.0
4.0
3.0
2.0
1.0
0
7.458
7.443
7.312
7.306
7.299
7.290
7.287
7.245
7.231
7.218
7.205
7.147
7.133
5.507
5.095
3.837
X : parts per Million : Proton

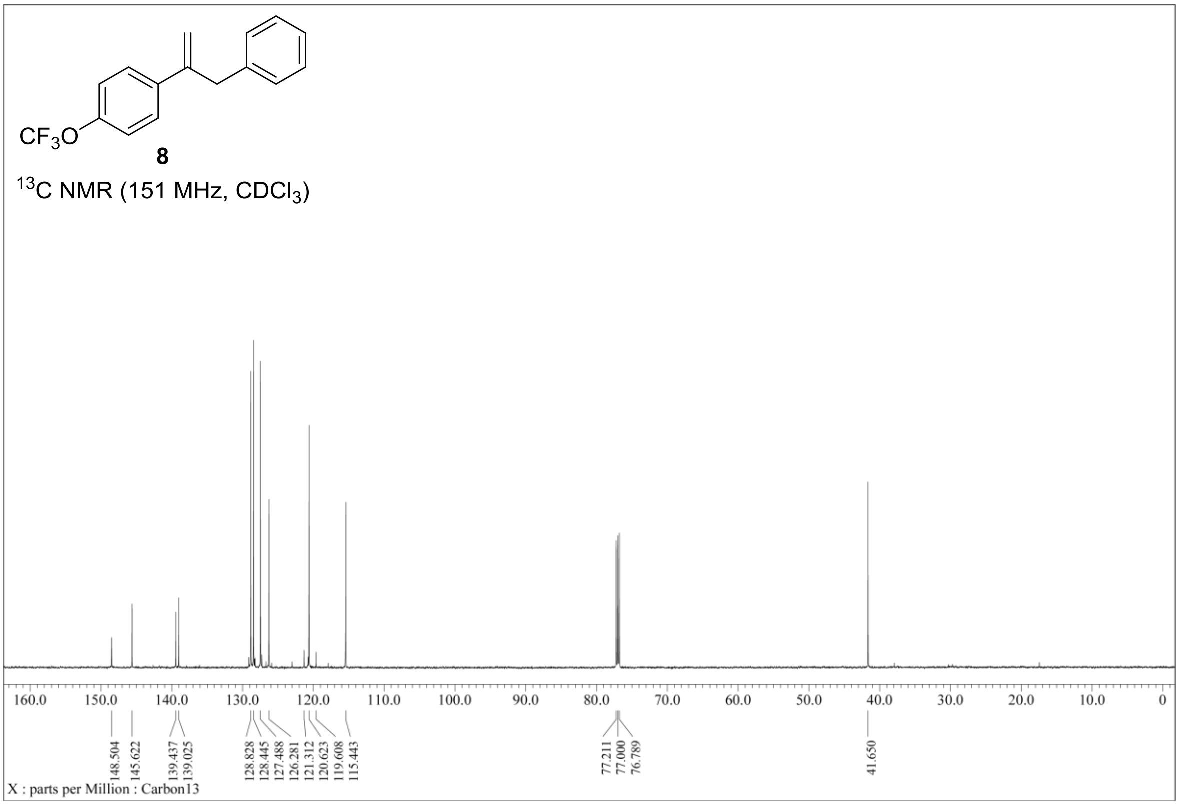
CF3O
8
13C NMR (151 MHz, CDCl3)
160.0
150.0
140.0
130.0
120.0
110.0
100.0
90.0
80.0
70.0
60.0
50.0
40.0
30.0
20.0
10.0
0
148.504
145.622
139.437
139.025
128.828
128.445
127.488
126.281
121.312
120.623
119.608
115.443
77.211
77.000
76.789
41.650
X : parts per Million : Carbon13

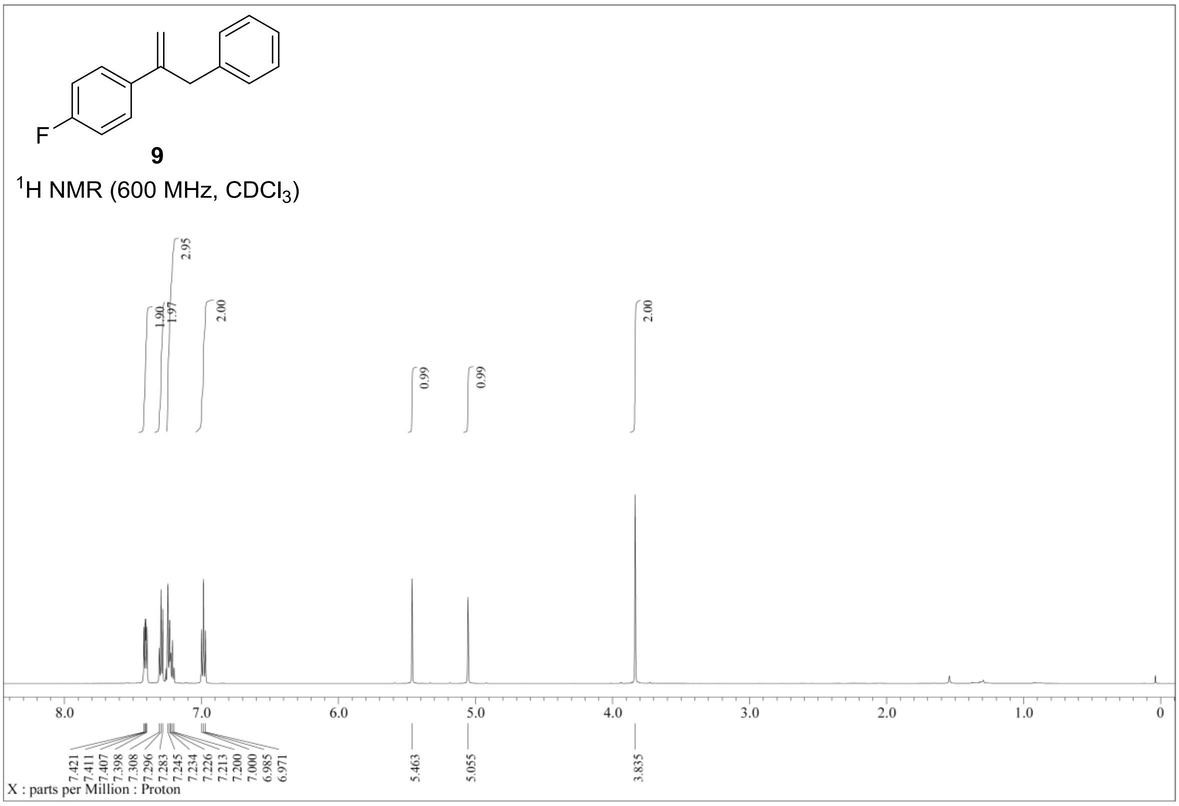
F
9
1H NMR (600 MHz, CDCl3)
2.95
1.90
1.97
2.00
0.99
0.99
2.00
8.0 7.0 6.0 5.0 4.0 3.0 2.0 1.0 0
7.421 7.411 7.407 7.398 7.308 7.296 7.283 7.245 7.234 7.226 7.213 7.200 7.000 6.985 6.971
5.463
5.055
3.835
X : parts per Million : Proton

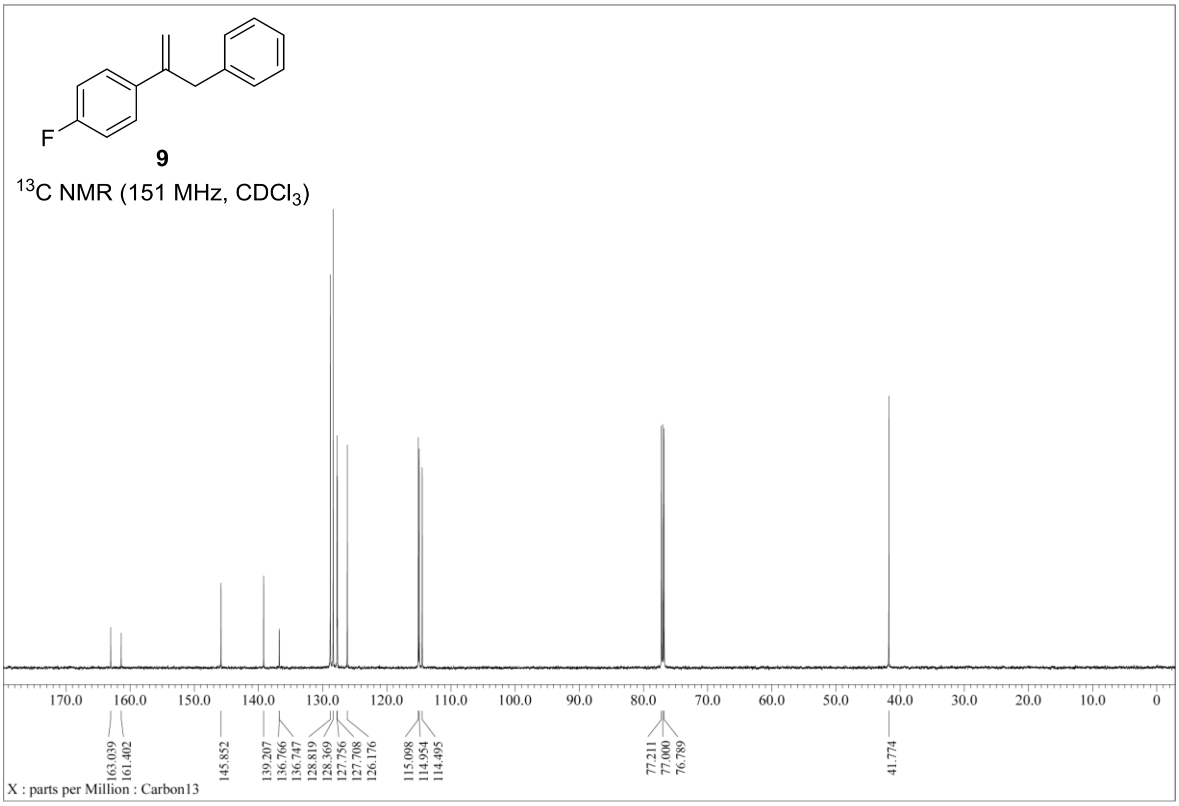
F
9
13C NMR (151 MHz, CDCl3)
170.0 160.0 150.0 140.0 130.0 120.0 110.0 100.0 90.0 80.0 70.0 60.0 50.0 40.0 30.0 20.0 10.0 0
163.039 161.402
145.852
139.207 136.766 136.747 128.819 128.369 127.756 127.708 126.176
115.098 114.954 114.495
77.211 77.000 76.789
41.774
X : parts per Million : Carbon13

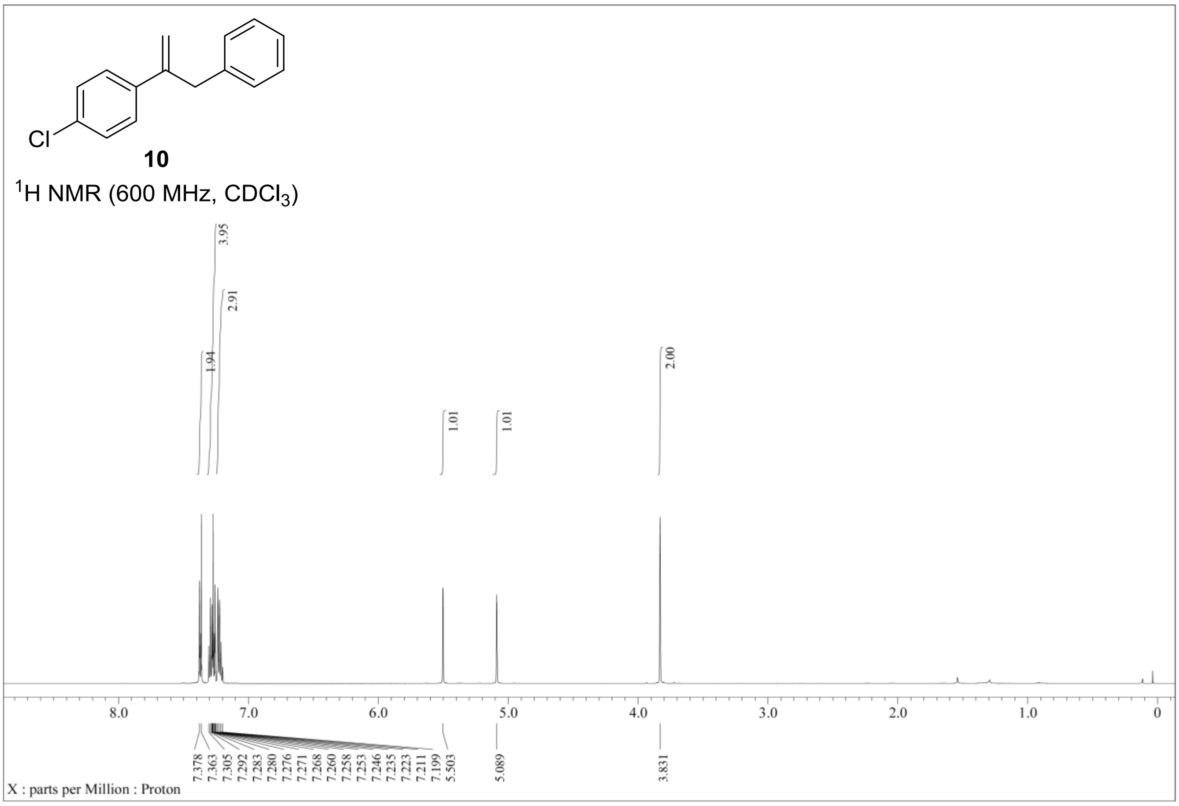
Cl
10
1H NMR (600 MHz, CDCl3)
3.95
2.91
1.94
1.01
1.01
2.00
8.0
7.0
6.0
5.0
4.0
3.0
2.0
1.0
0
7.378
7.363
7.305
7.292
7.283
7.280
7.276
7.271
7.268
7.260
7.258
7.253
7.246
7.235
7.223
7.211
7.199
5.503
5.089
3.831
X : parts per Million : Proton

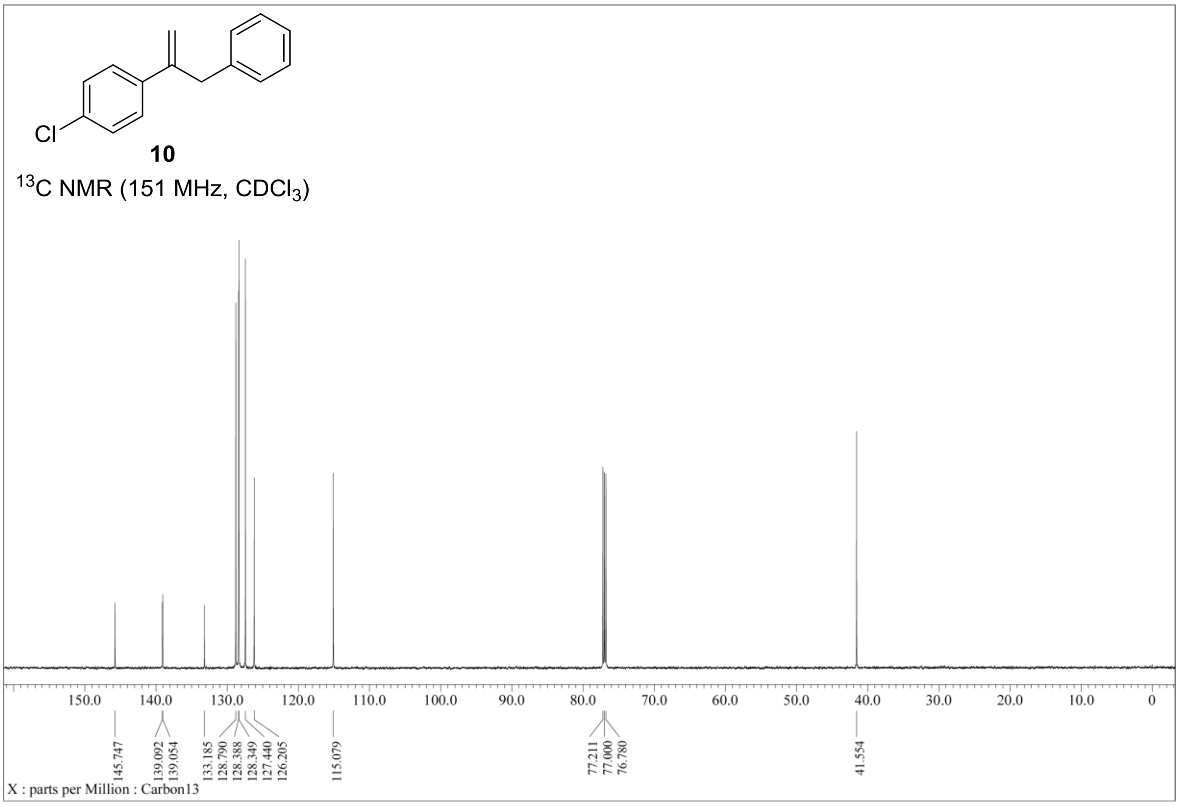
Cl
10
13C NMR (151 MHz, CDCl3)
150.0
140.0
130.0
120.0
110.0
100.0
90.0
80.0
70.0
60.0
50.0
40.0
30.0
20.0
10.0
0
145.747
139.092
139.054
133.185
128.790
128.388
128.349
127.440
126.205
115.079
77.211
77.000
76.780
41.554
X : parts per Million : Carbon13

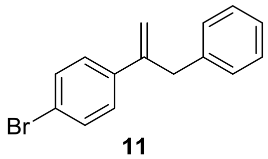

11

^{1}H NMR (600 MHz, $CDCl_3$)

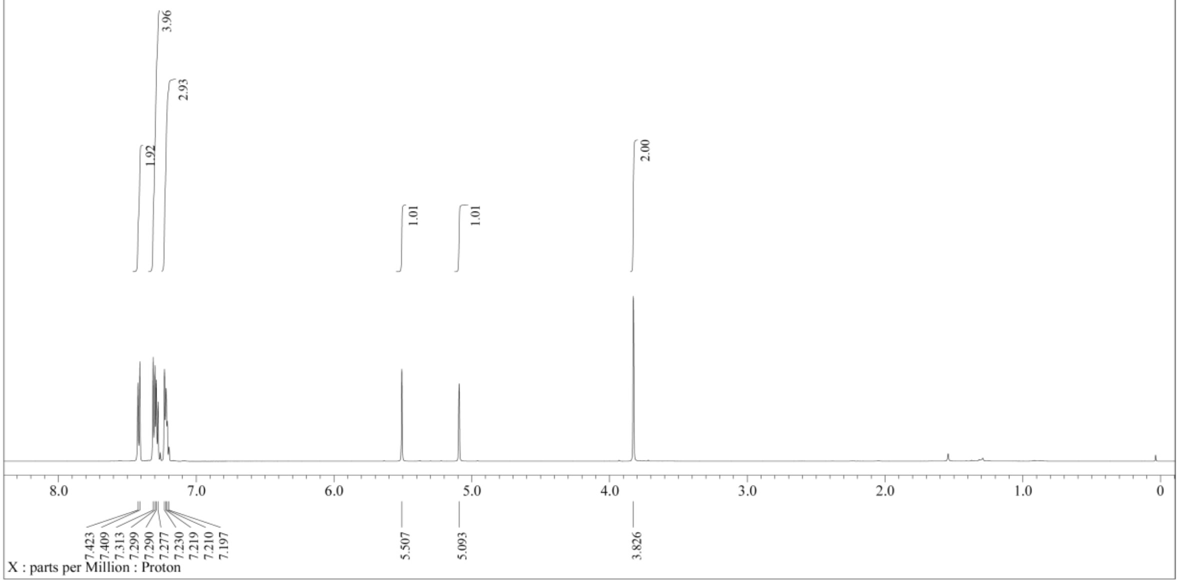

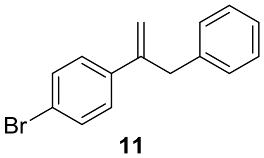

11

^{13}C NMR (151 MHz, $CDCl_3$)

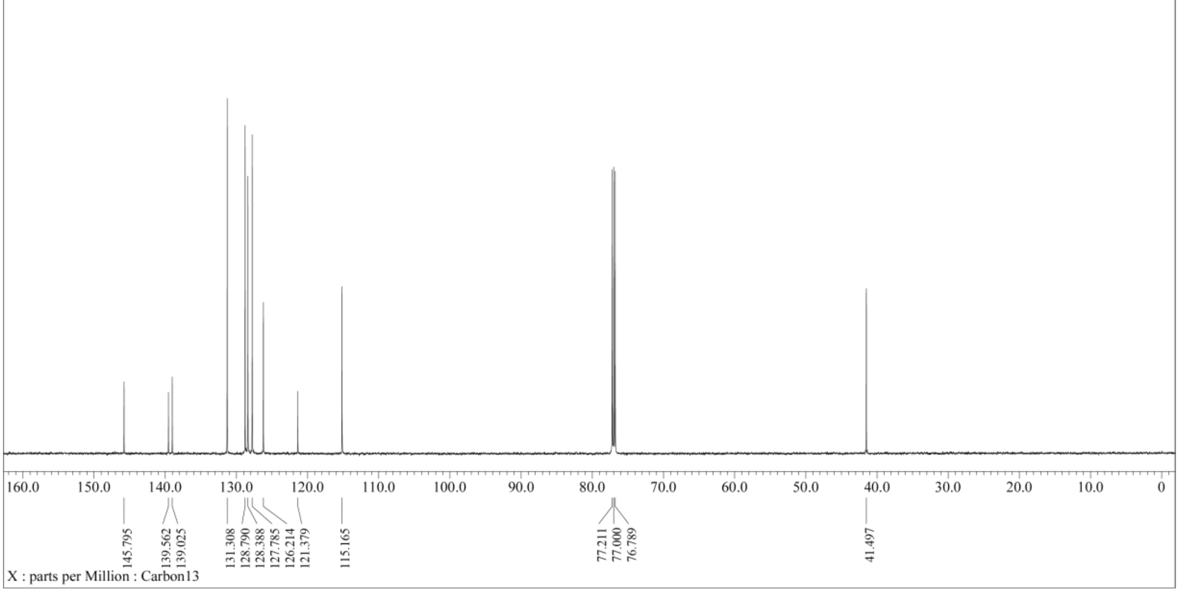

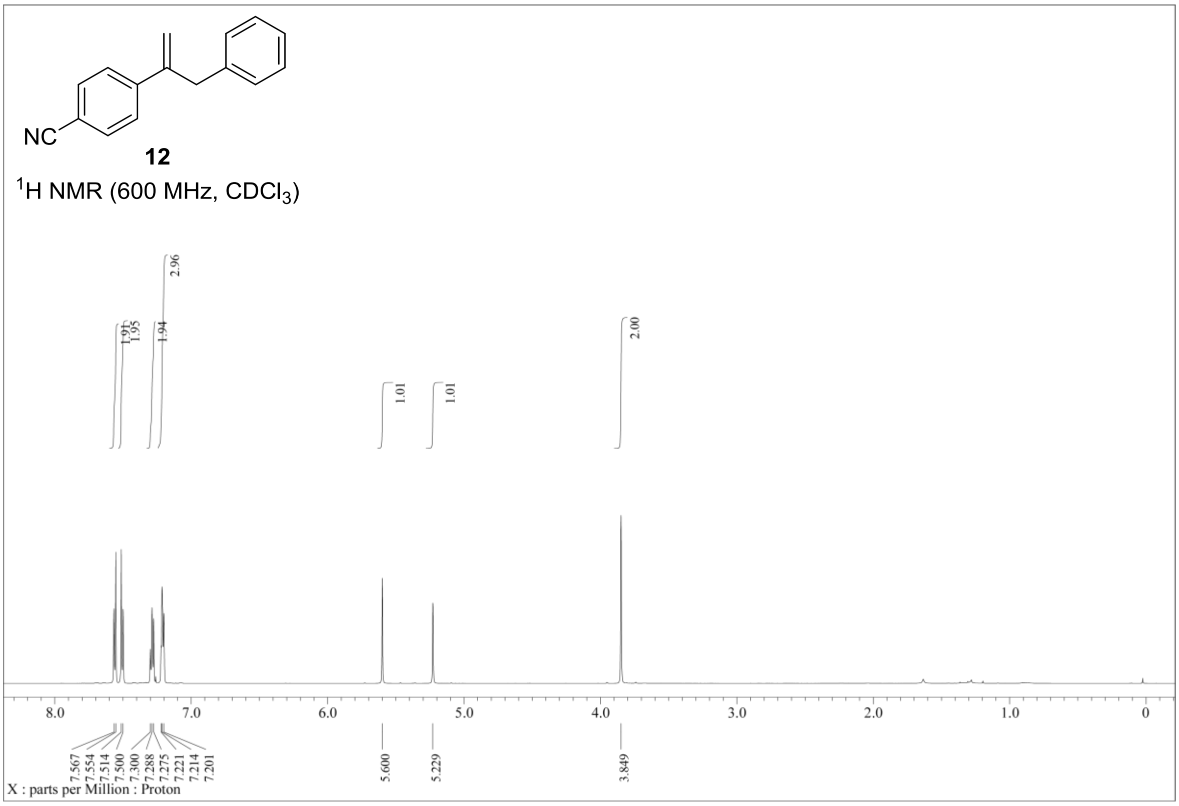
NC
12
1H NMR (600 MHz, CDCl3)
2.96
1.91
1.95
1.94
1.01
1.01
2.00
8.0
7.0
6.0
5.0
4.0
3.0
2.0
1.0
0
7.567
7.554
7.514
7.500
7.300
7.288
7.275
7.221
7.214
7.201
5.600
5.229
3.849
X : parts per Million : Proton

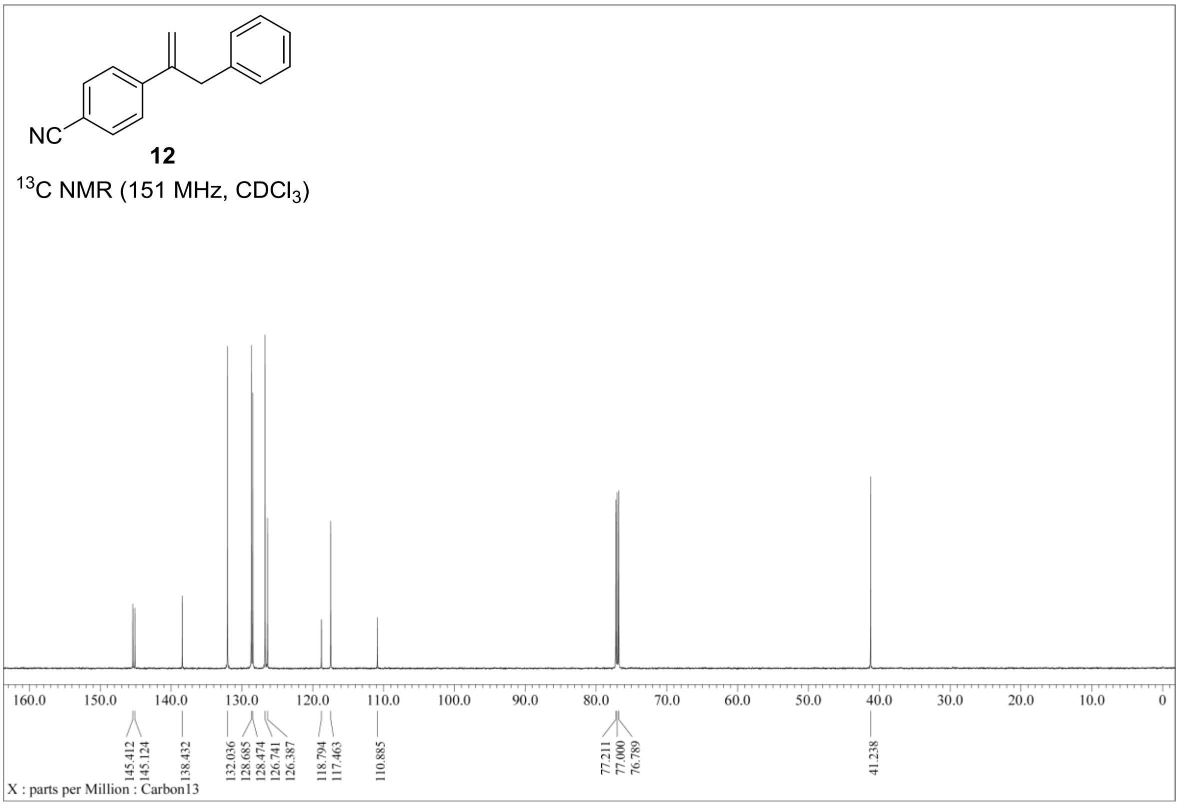
NC
12
13C NMR (151 MHz, CDCl3)
160.0
150.0
140.0
130.0
120.0
110.0
100.0
90.0
80.0
70.0
60.0
50.0
40.0
30.0
20.0
10.0
0
145.412
145.124
138.432
132.036
128.685
128.474
126.741
126.387
118.794
117.463
110.885
77.211
77.000
76.789
41.238
X : parts per Million : Carbon13

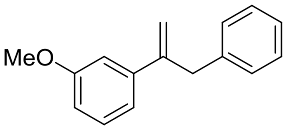

13

^{1}H NMR (600 MHz, $CDCl_3$)

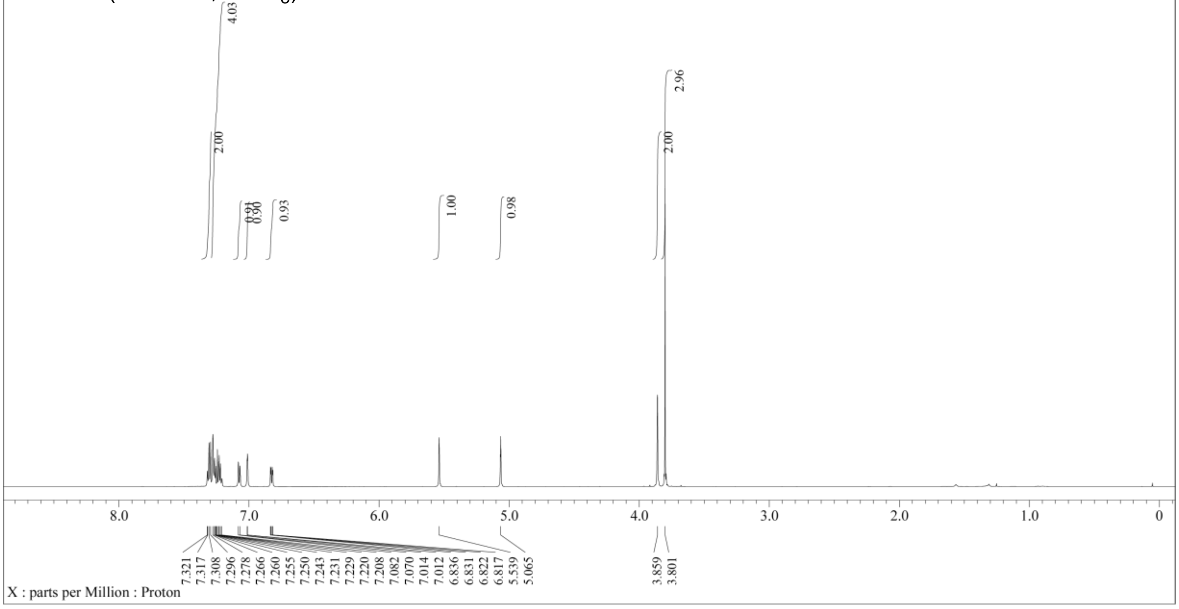

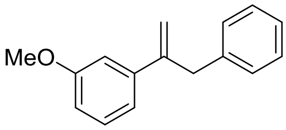

13

^{13}C NMR (151 MHz, $CDCl_3$)

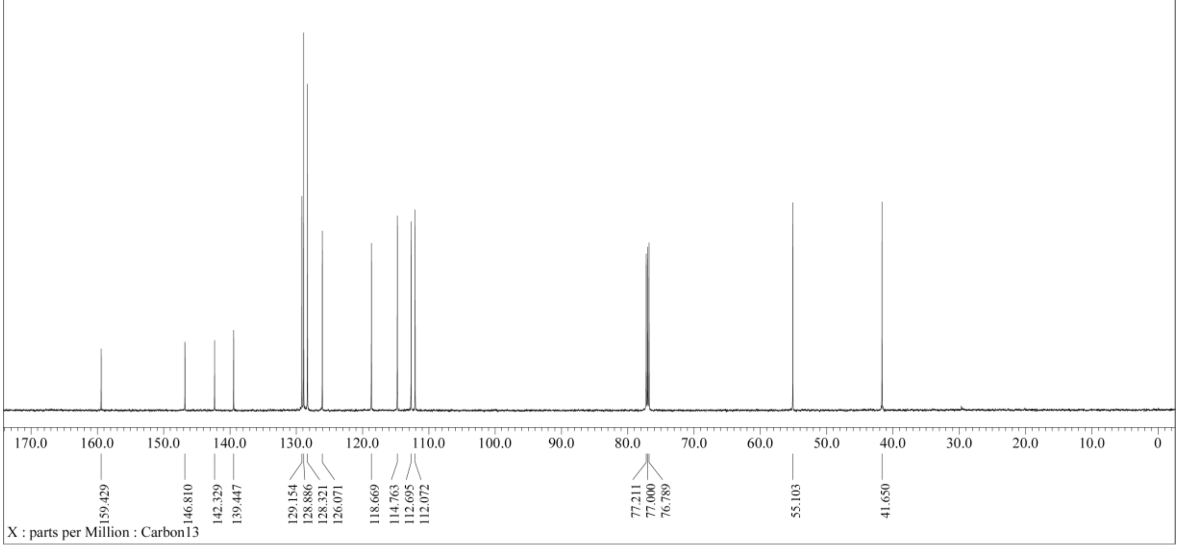

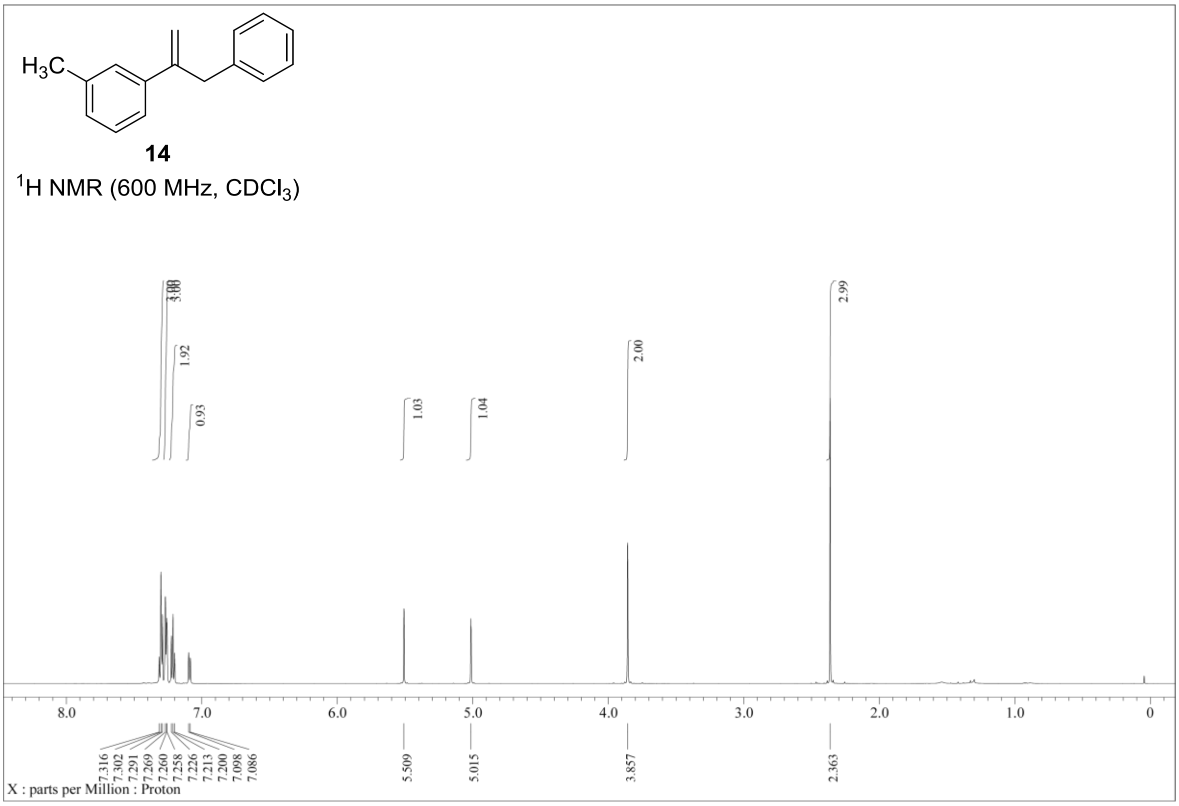
H3C
14
1H NMR (600 MHz, CDCl3)
3.00
3.00
1.92
0.93
1.03
1.04
2.00
2.99
8.0
7.0
6.0
5.0
4.0
3.0
2.0
1.0
0
7.316
7.302
7.291
7.269
7.260
7.258
7.226
7.213
7.200
7.098
7.086
5.509
5.015
3.857
2.363
X : parts per Million : Proton

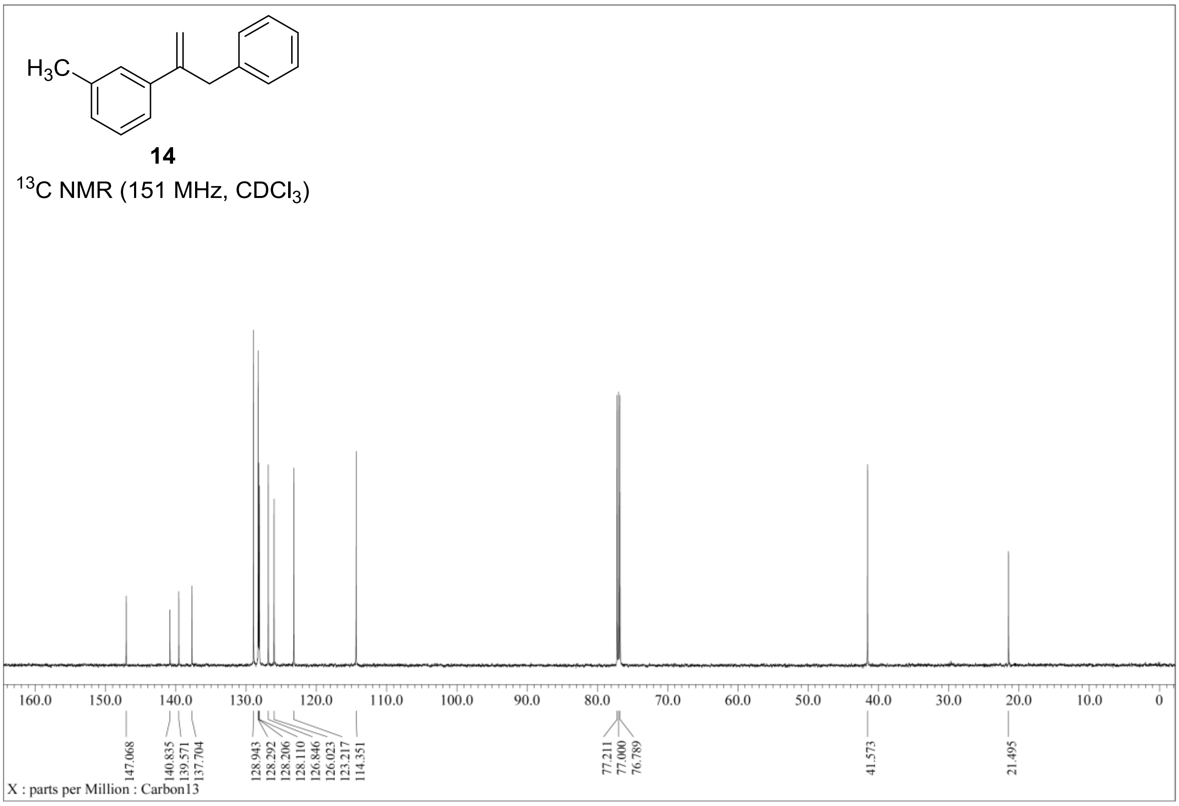
H3C
14
13C NMR (151 MHz, CDCl3)
160.0
150.0
140.0
130.0
120.0
110.0
100.0
90.0
80.0
70.0
60.0
50.0
40.0
30.0
20.0
10.0
0
147.068
140.835
139.571
137.704
128.943
128.292
128.206
128.110
126.846
126.023
123.217
114.351
77.211
77.000
76.789
41.573
21.495
X : parts per Million : Carbon13

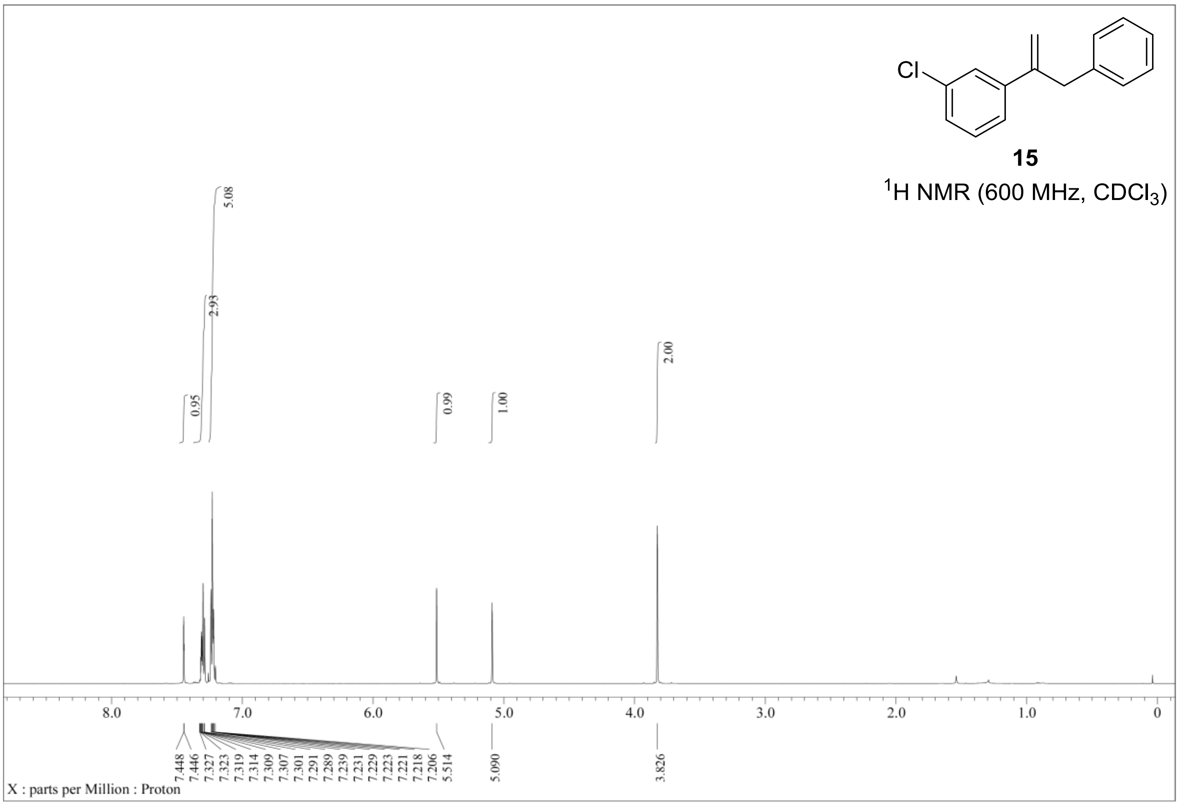
Cl
15
^{1}H NMR (600 MHz, $CDCl_3$)
0.95
2.93
5.08
0.99
1.00
2.00
8.0
7.0
6.0
5.0
4.0
3.0
2.0
1.0
0
7.448
7.446
7.327
7.323
7.319
7.314
7.309
7.307
7.301
7.291
7.289
7.239
7.231
7.229
7.223
7.221
7.218
7.206
5.514
5.090
3.826
X : parts per Million : Proton

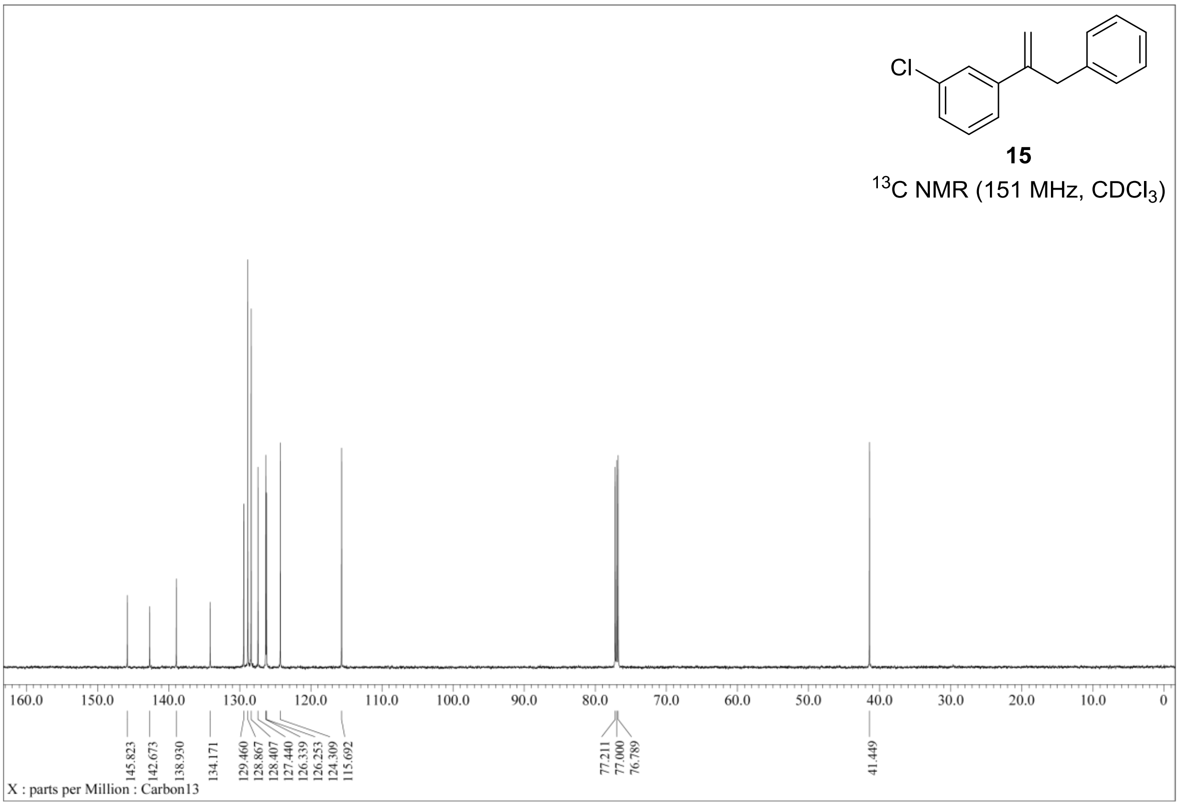
Cl
15
^{13}C NMR (151 MHz, $CDCl_3$)
160.0
150.0
140.0
130.0
120.0
110.0
100.0
90.0
80.0
70.0
60.0
50.0
40.0
30.0
20.0
10.0
0
145.823
142.673
138.930
134.171
129.460
128.867
128.407
127.440
126.339
126.253
124.309
115.692
77.211
77.000
76.789
41.449
X : parts per Million : Carbon13

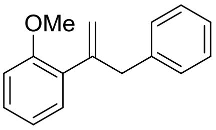

16

^{1}H NMR (600 MHz, $CDCl_3$)

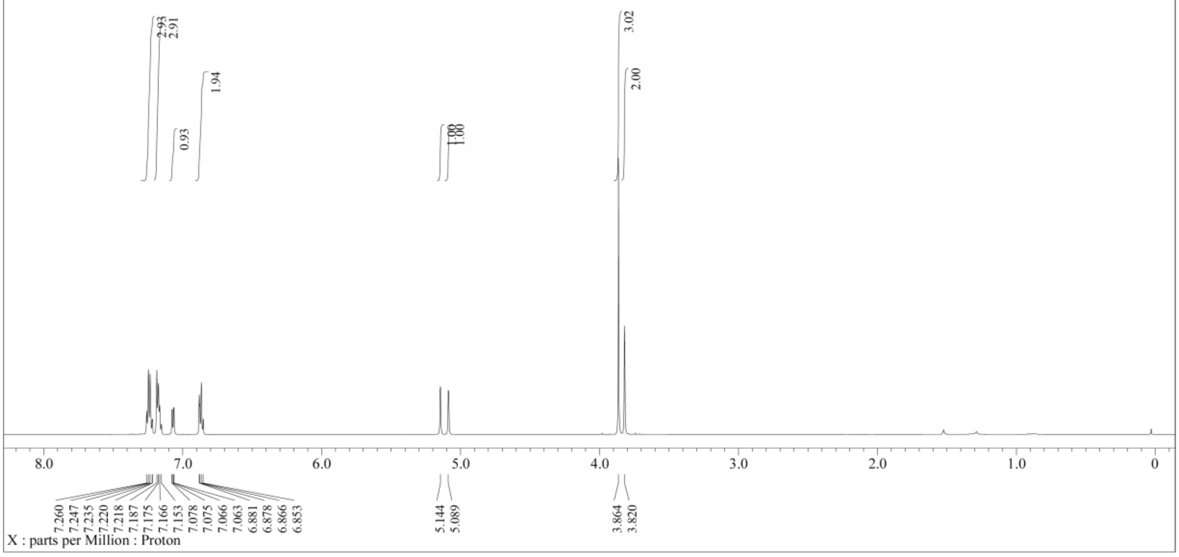

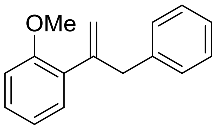

16

^{13}C NMR (151 MHz, $CDCl_3$)

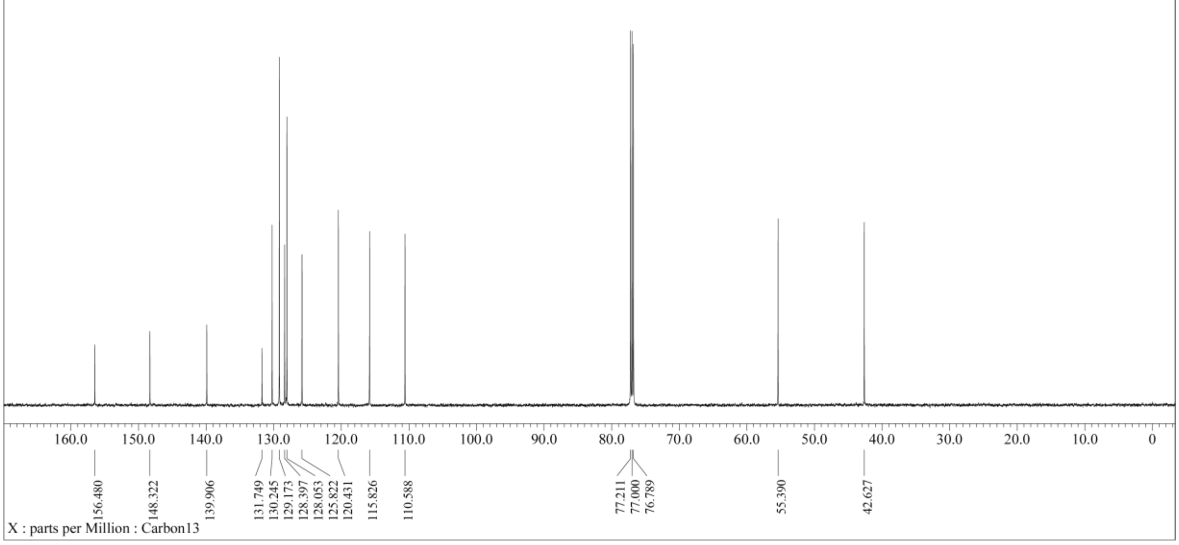

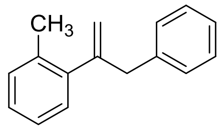

17

^1H NMR (600 MHz, $CDCl_3$)

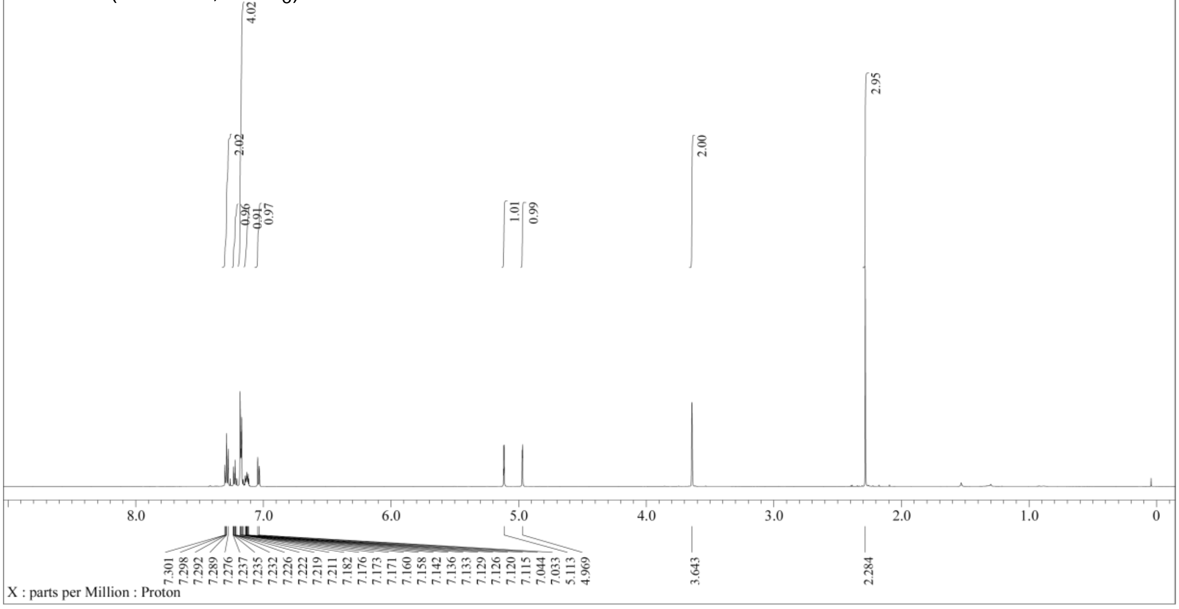

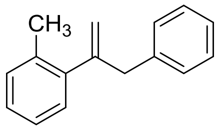

17

^{13}C NMR (151 MHz, $CDCl_3$)

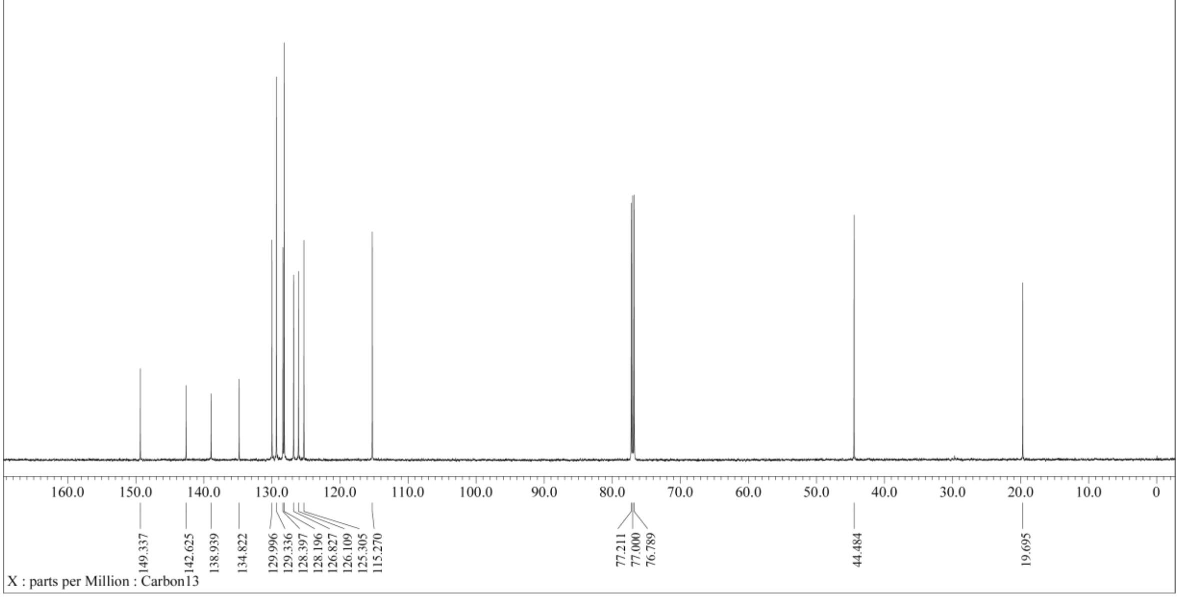

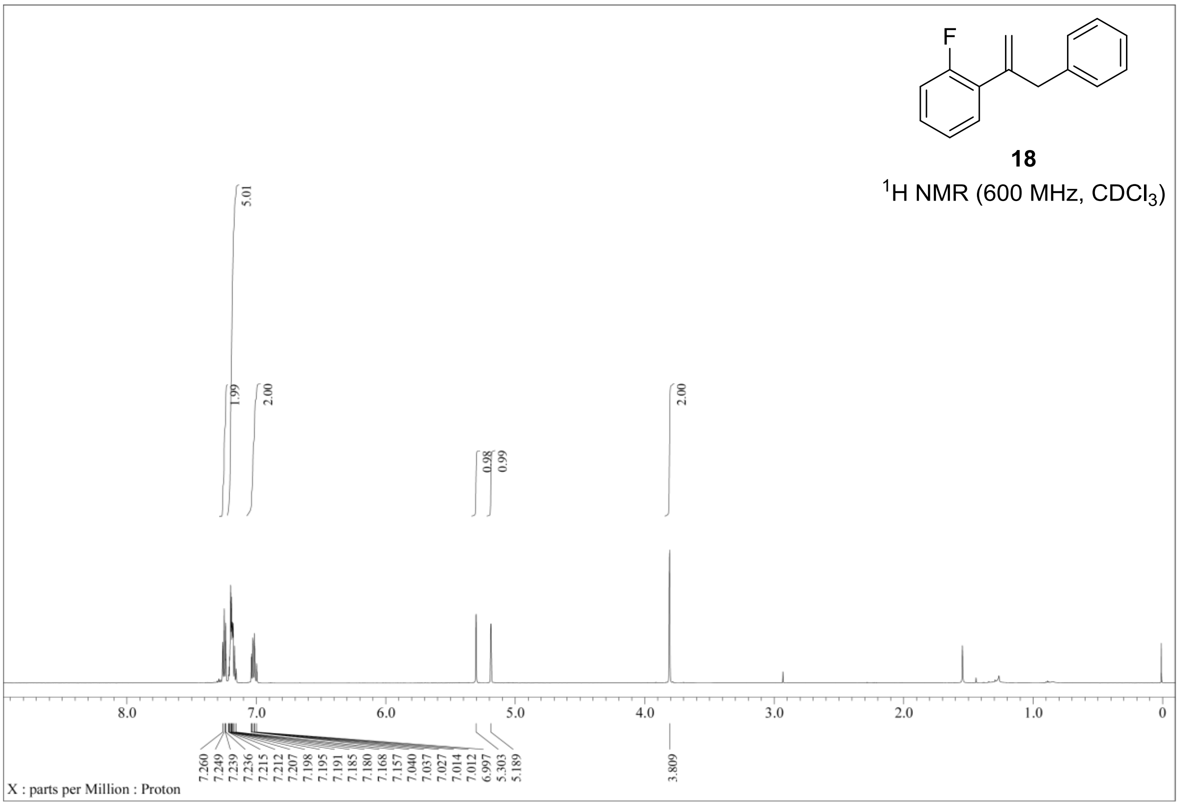
18
^{1}H NMR (600 MHz, CDCl$_3$)
X : parts per Million : Proton

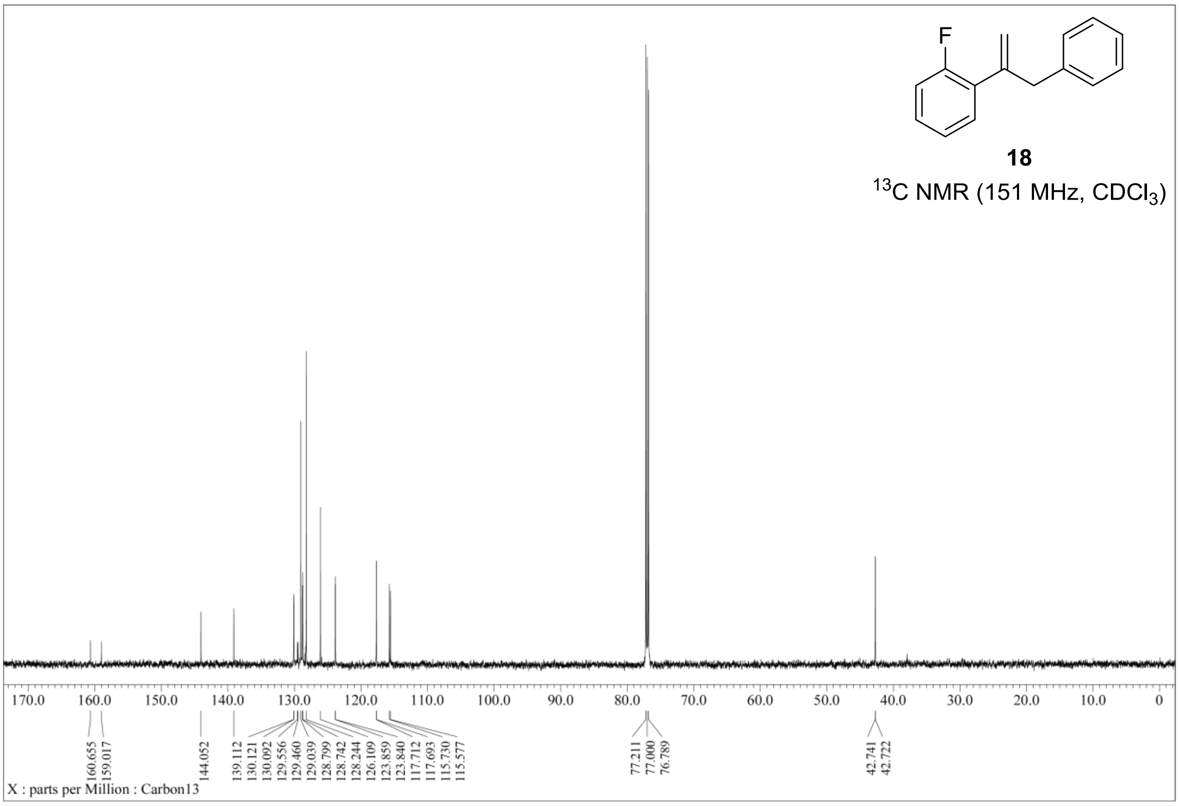
18
^{13}C NMR (151 MHz, CDCl$_3$)
X : parts per Million : Carbon13

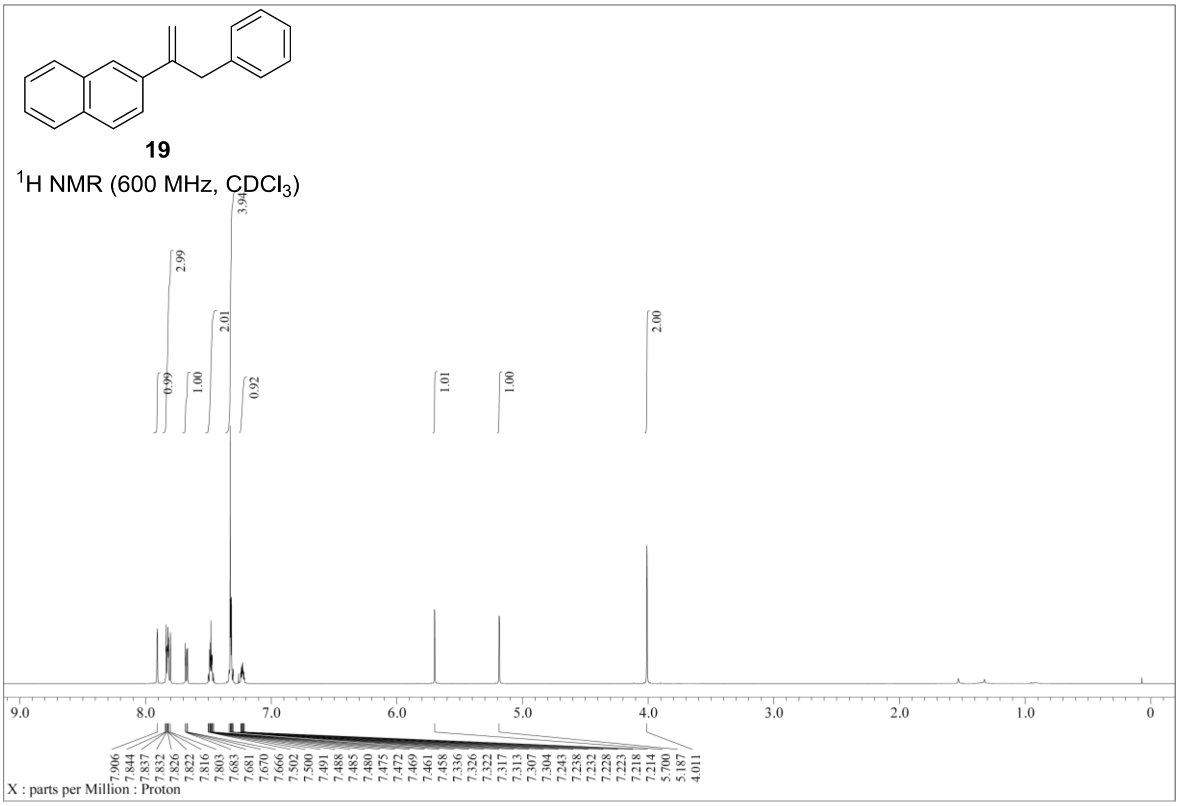
19
^{1}H NMR (600 MHz, $CDCl_3$)
X : parts per Million : Proton

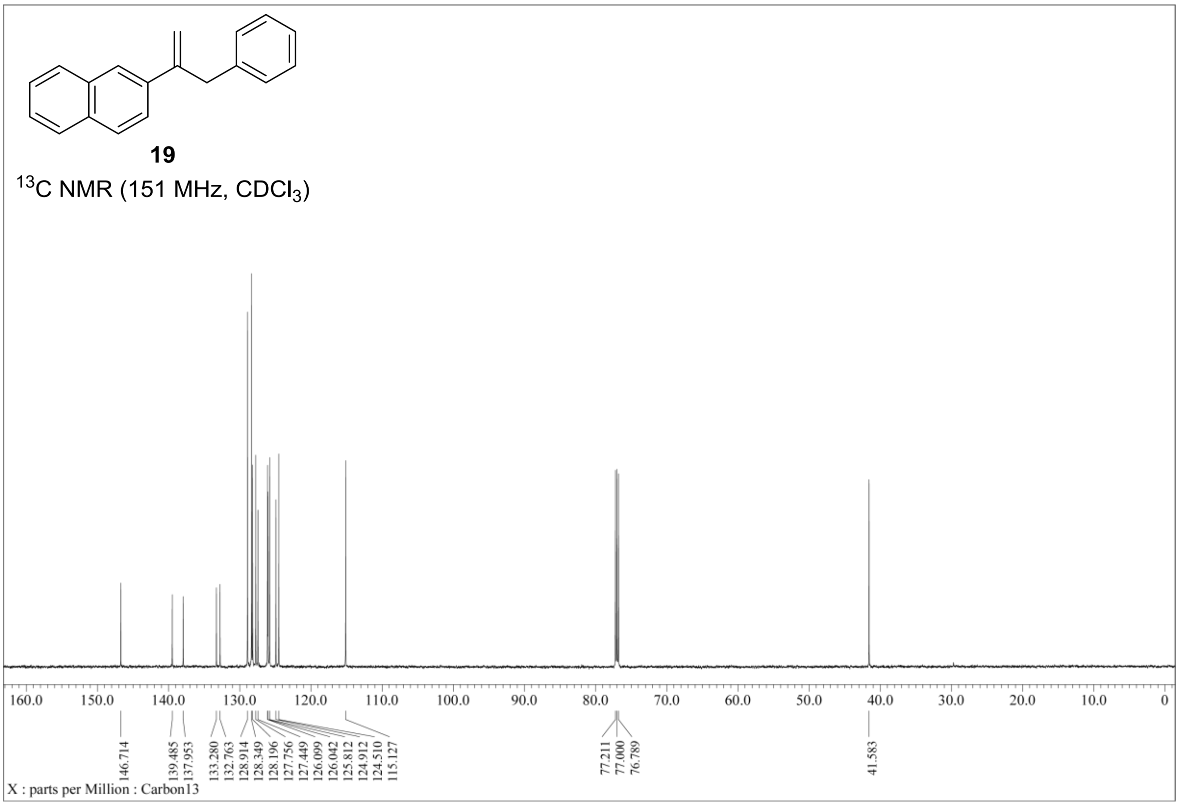
19
^{13}C NMR (151 MHz, $CDCl_3$)
X : parts per Million : Carbon13

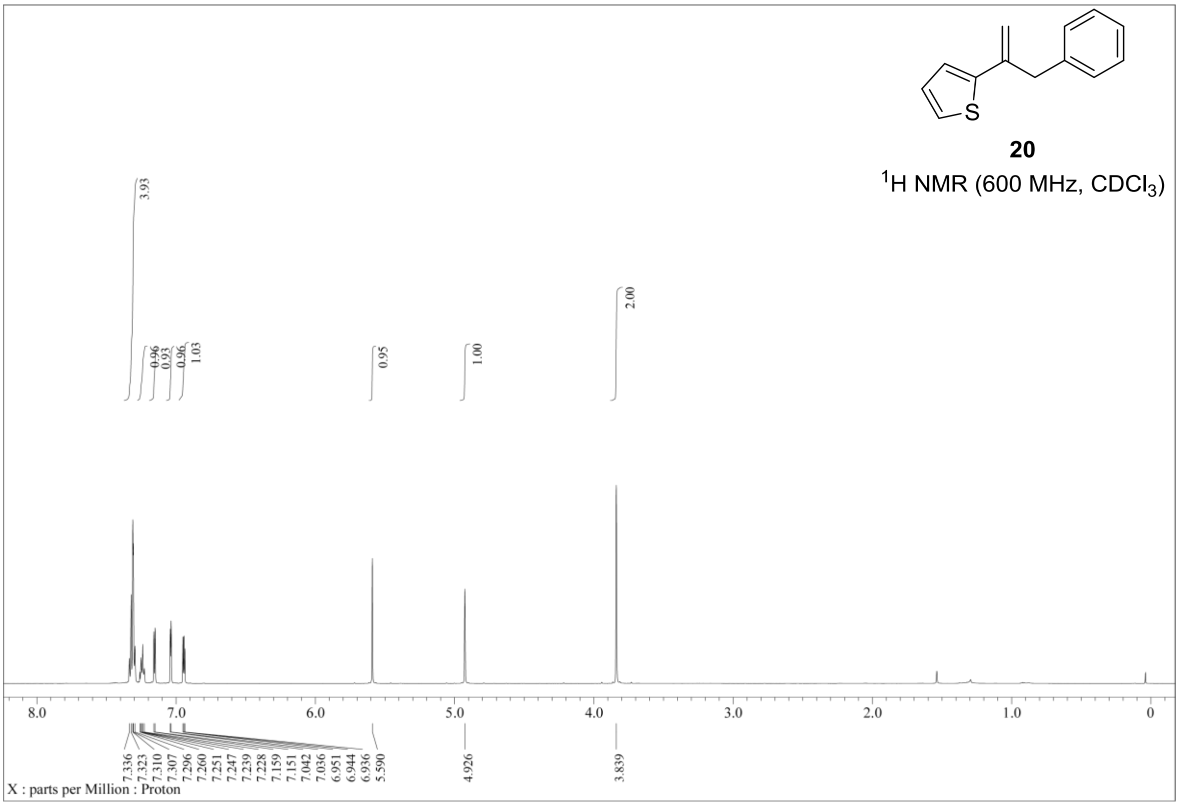
S
20
1H NMR (600 MHz, CDCl3)
3.93
0.96
0.93
0.96
1.03
0.95
1.00
2.00
8.0
7.0
6.0
5.0
4.0
3.0
2.0
1.0
0
7.336
7.323
7.310
7.307
7.296
7.260
7.251
7.247
7.239
7.228
7.159
7.151
7.042
7.036
6.951
6.944
6.936
5.590
4.926
3.839
X : parts per Million : Proton

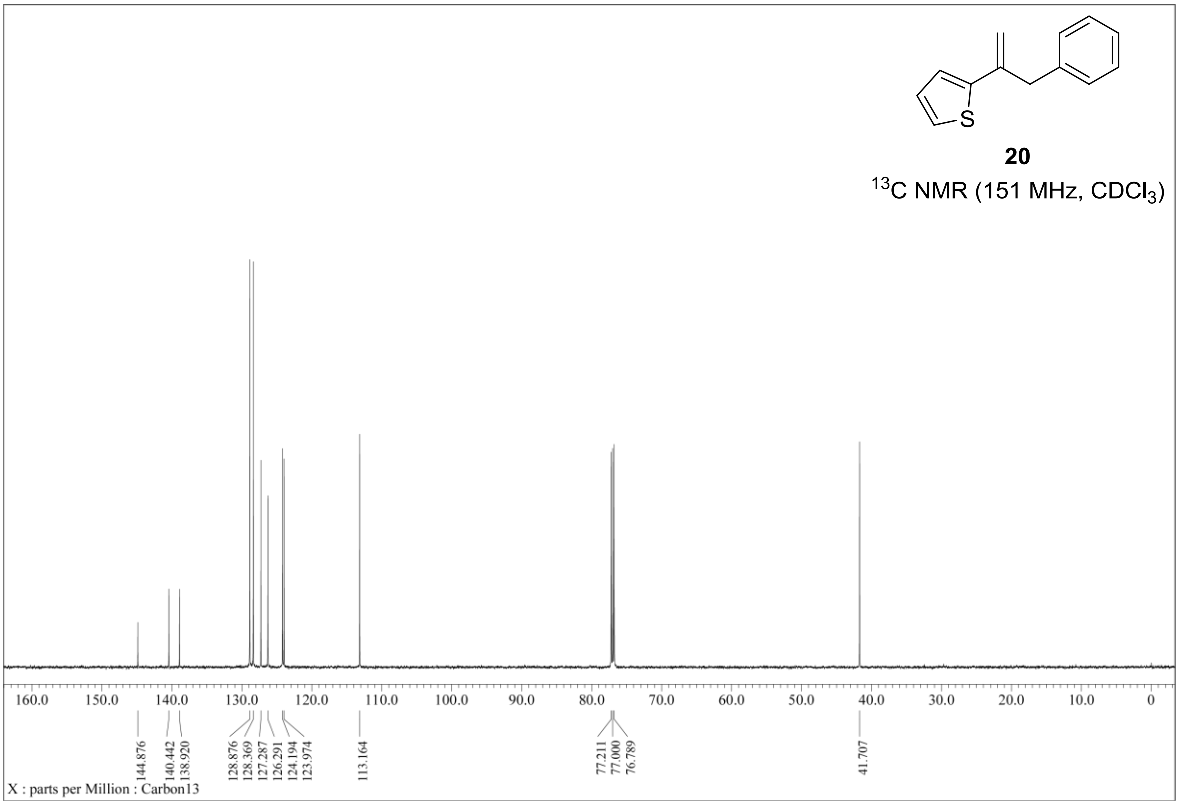
S
20
13C NMR (151 MHz, CDCl3)
160.0
150.0
140.0
130.0
120.0
110.0
100.0
90.0
80.0
70.0
60.0
50.0
40.0
30.0
20.0
10.0
0
144.876
140.442
138.920
128.876
128.369
127.287
126.291
124.194
123.974
113.164
77.211
77.000
76.789
41.707
X : parts per Million : Carbon13

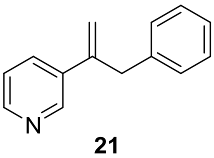

21

^{1}H NMR (600 MHz, $CDCl_3$)

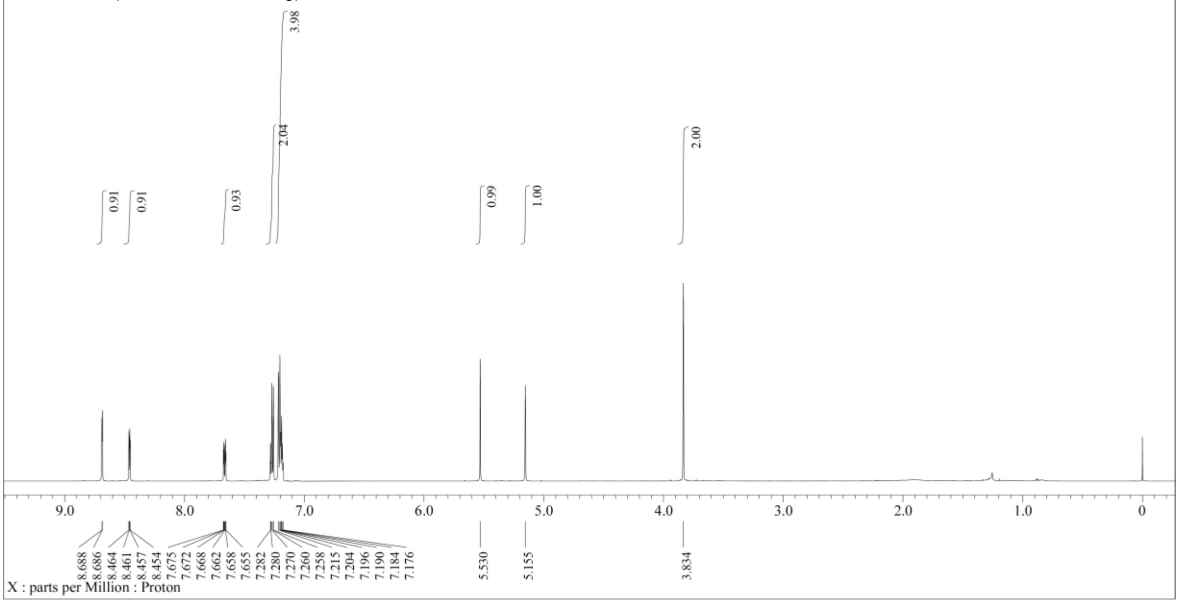

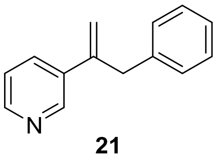

21

^{13}C NMR (151 MHz, $CDCl_3$)

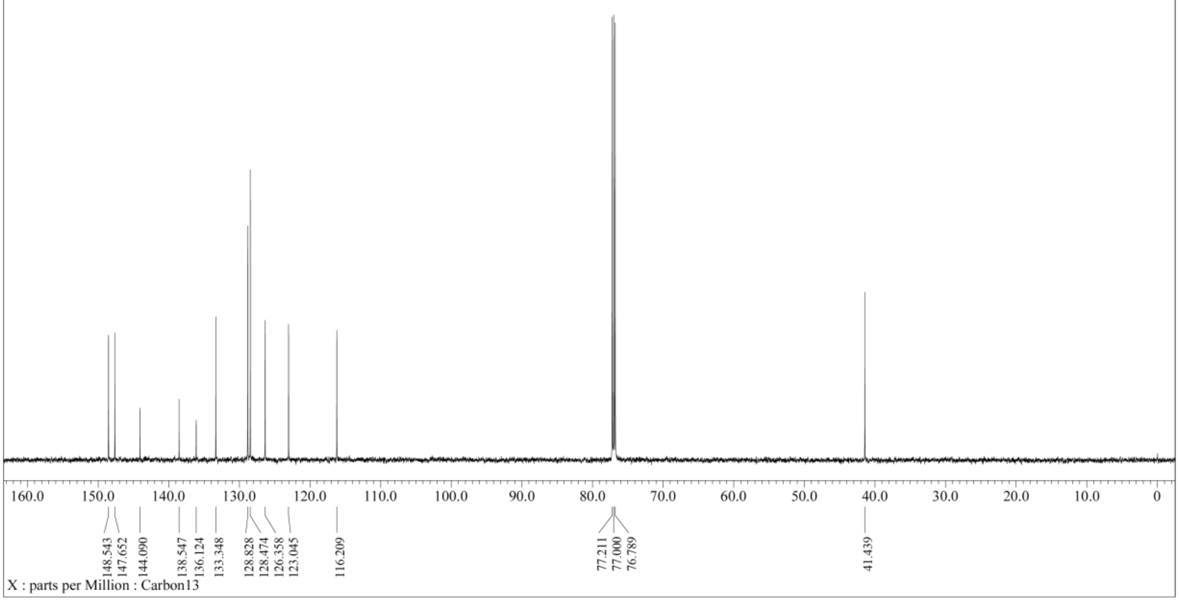

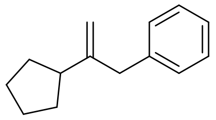

22

^{1}H NMR (600 MHz, $CDCl_3$)

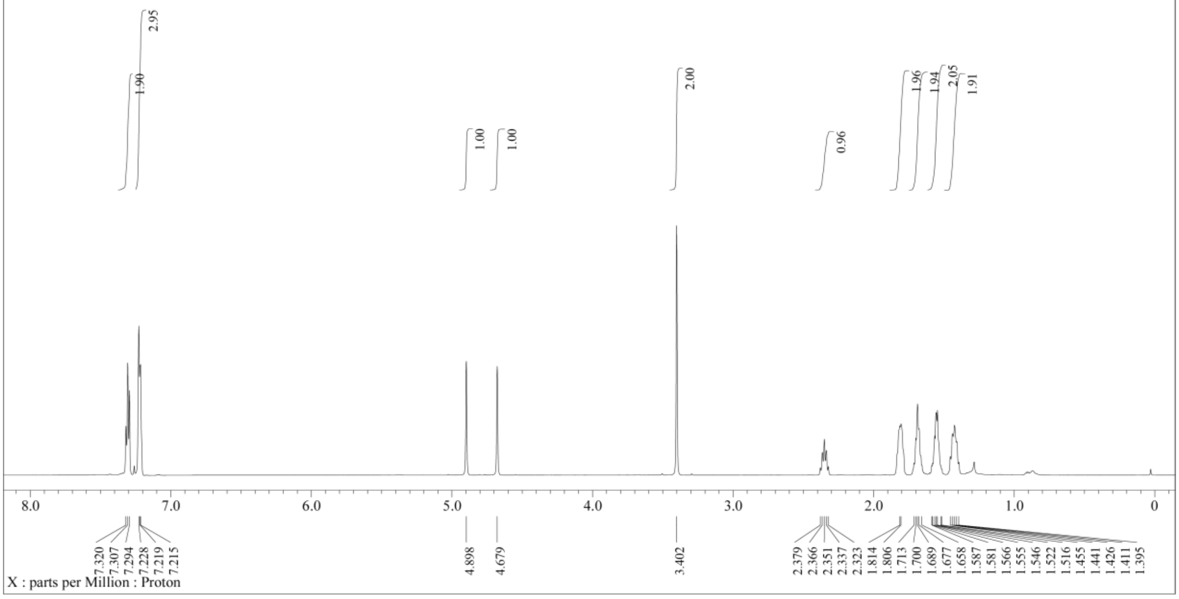

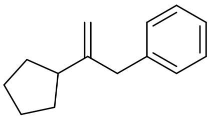

22

^{13}C NMR (151 MHz, $CDCl_3$)

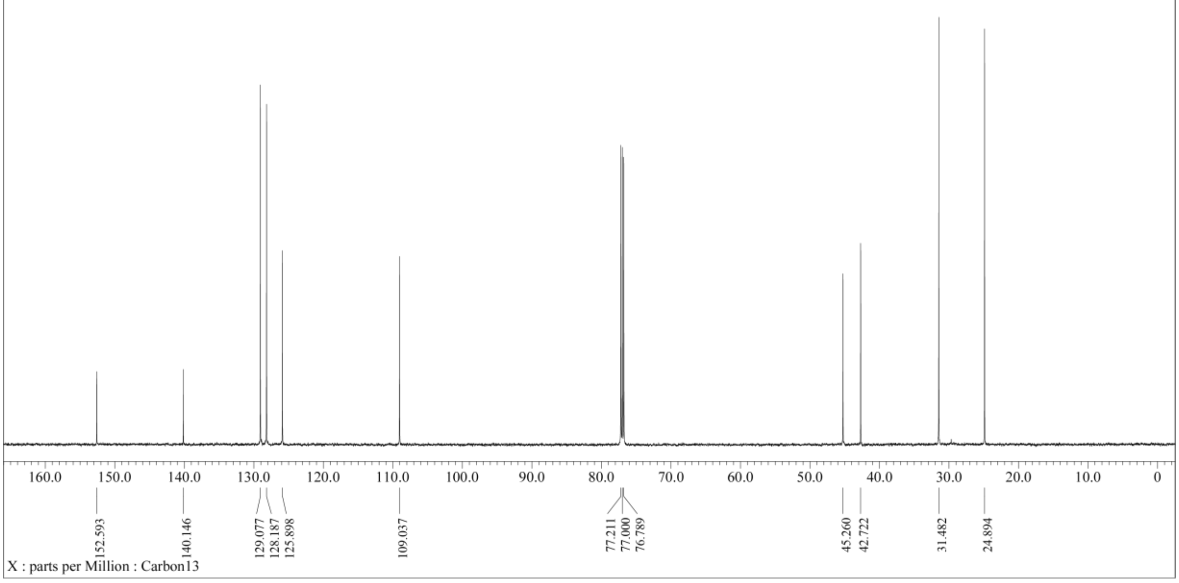

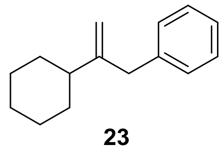

23

^{1}H NMR (600 MHz, $CDCl_3$)

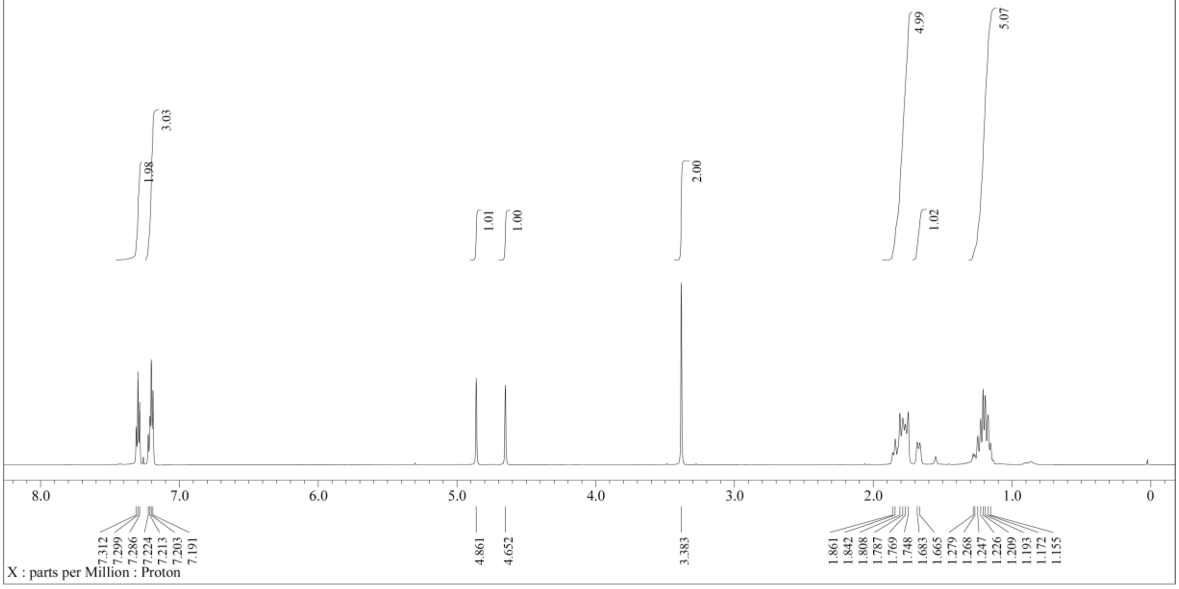

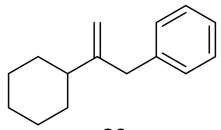

23

^{13}C NMR (151 MHz, $CDCl_3$)

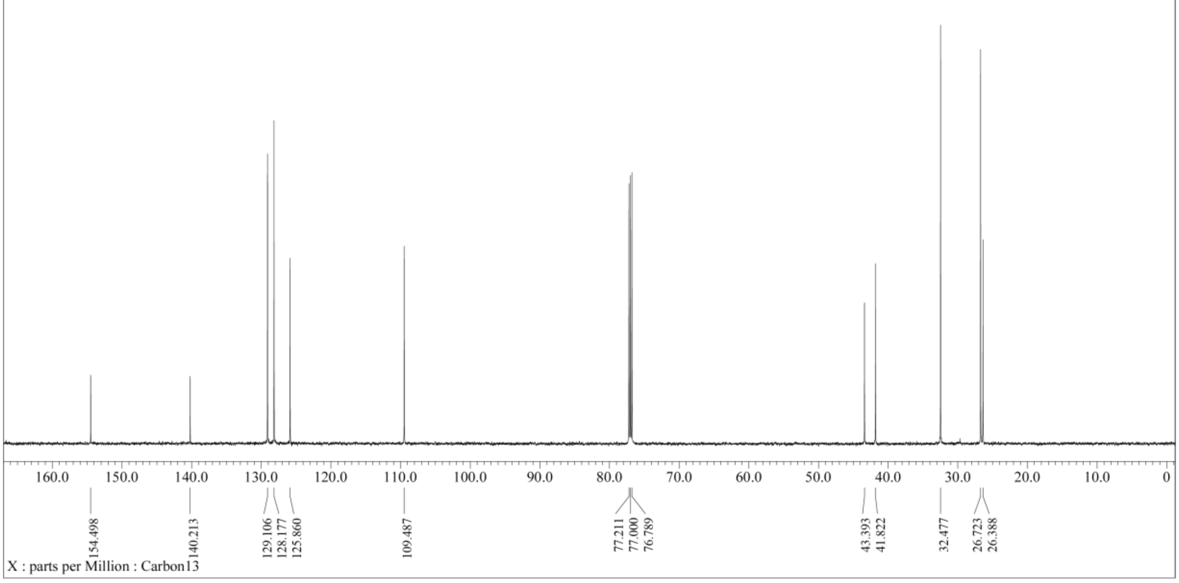

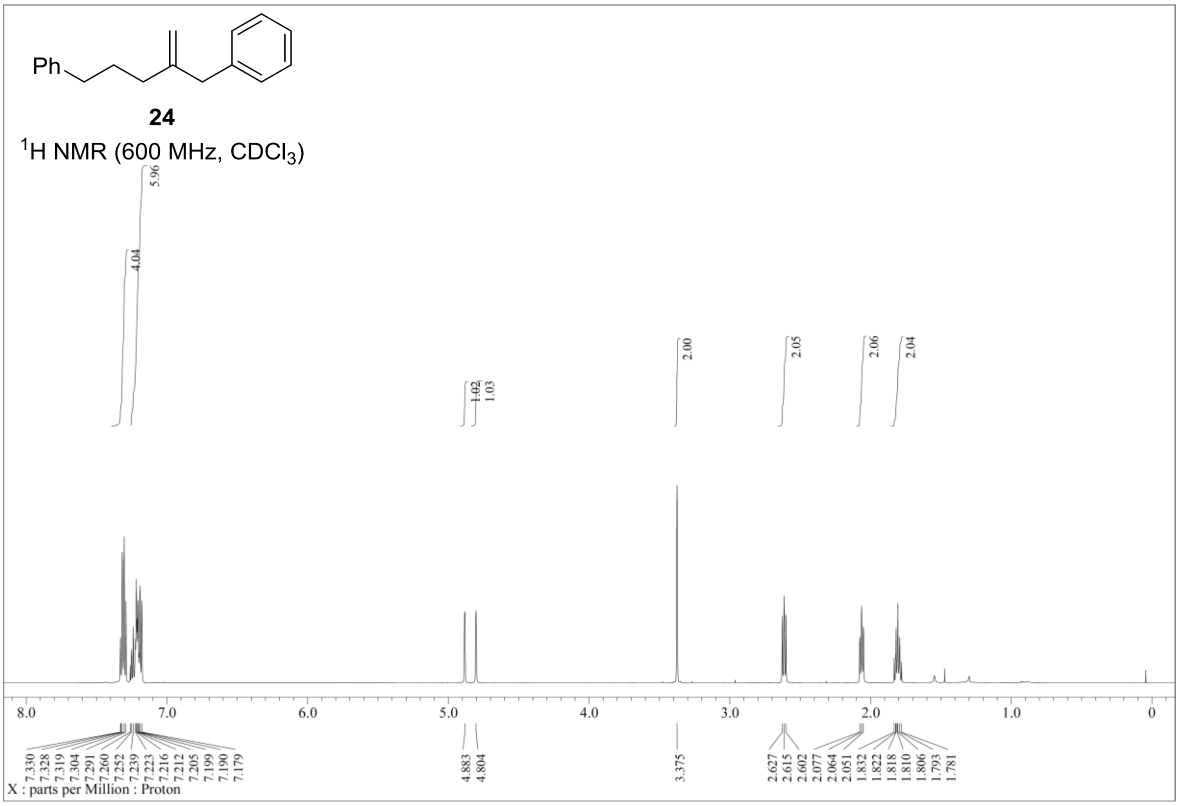
Ph
24
1H NMR (600 MHz, CDCl3)
5.96
4.04
1.02
1.03
2.00
2.05
2.06
2.04
8.0
7.0
6.0
5.0
4.0
3.0
2.0
1.0
0
7.330 7.328 7.319 7.304 7.291 7.260 7.252 7.239 7.223 7.216 7.212 7.205 7.199 7.190 7.179
4.883 4.804
3.375
2.627 2.615 2.602 2.077 2.064 2.051 1.832 1.822 1.818 1.810 1.806 1.793 1.781
X : parts per Million : Proton

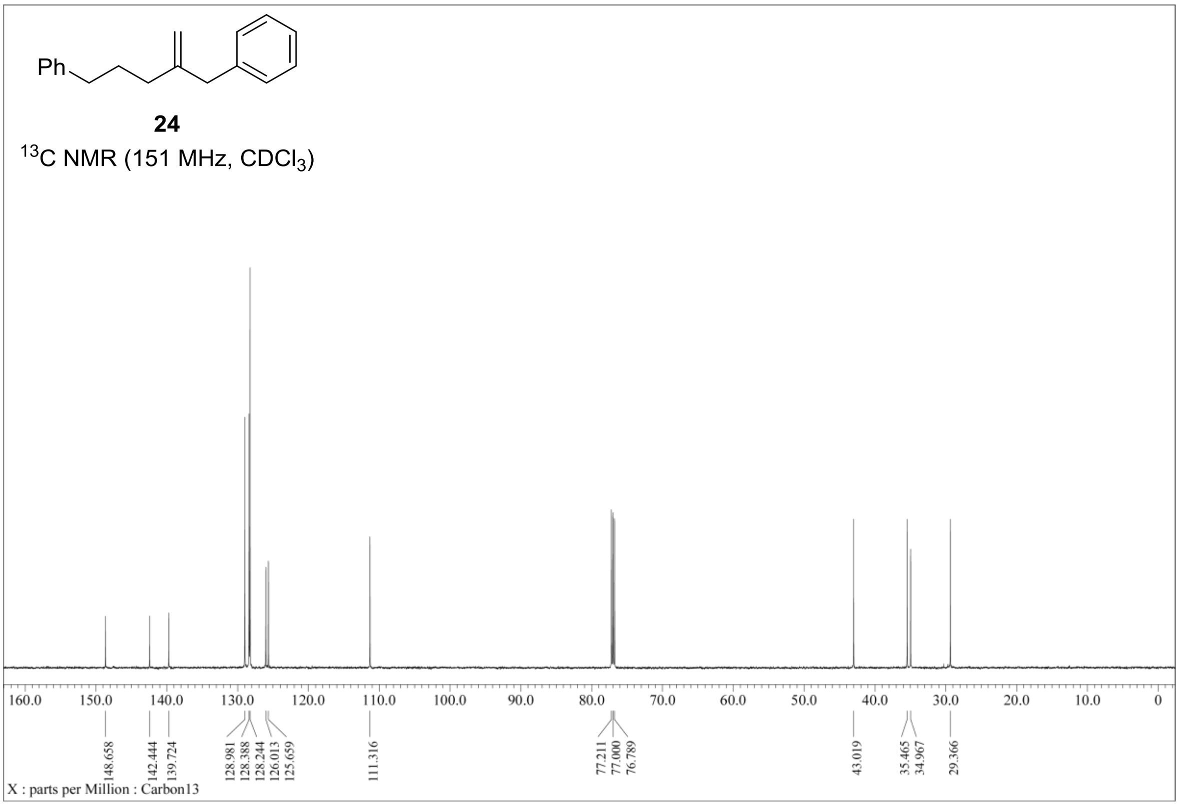
Ph
24
13C NMR (151 MHz, CDCl3)
160.0
150.0
140.0
130.0
120.0
110.0
100.0
90.0
80.0
70.0
60.0
50.0
40.0
30.0
20.0
10.0
0
148.658
142.444 139.724
128.981 128.388 128.244 126.013 125.659
111.316
77.211 77.000 76.789
43.019
35.465 34.967
29.366
X : parts per Million : Carbon13

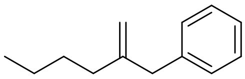

25

^{1}H NMR (600 MHz, $CDCl_3$)

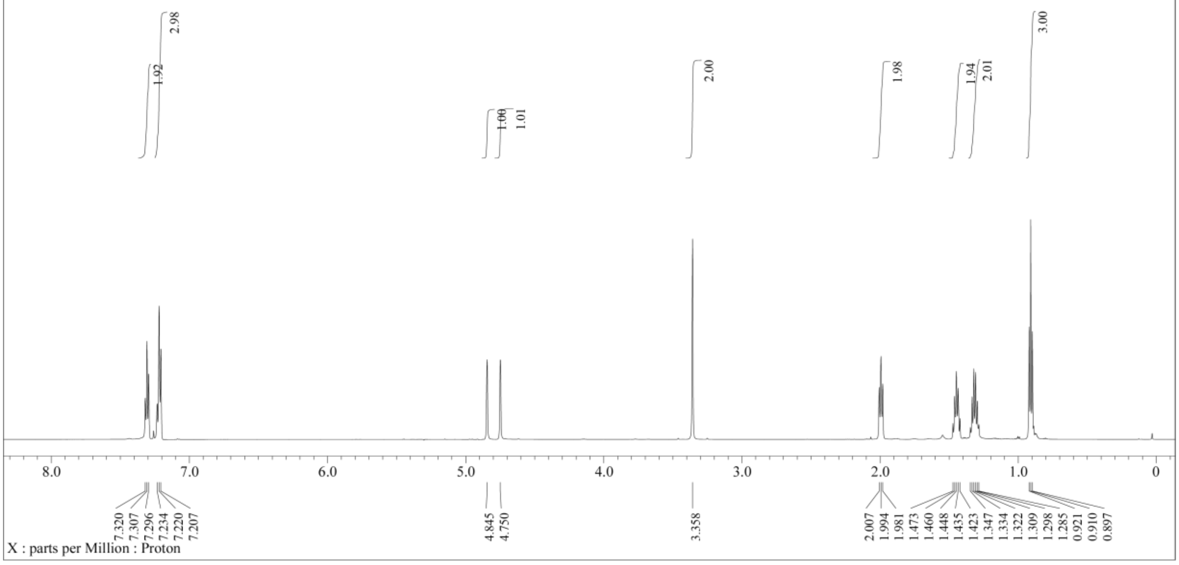

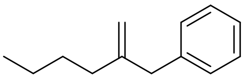

25

^{13}C NMR (151 MHz, $CDCl_3$)

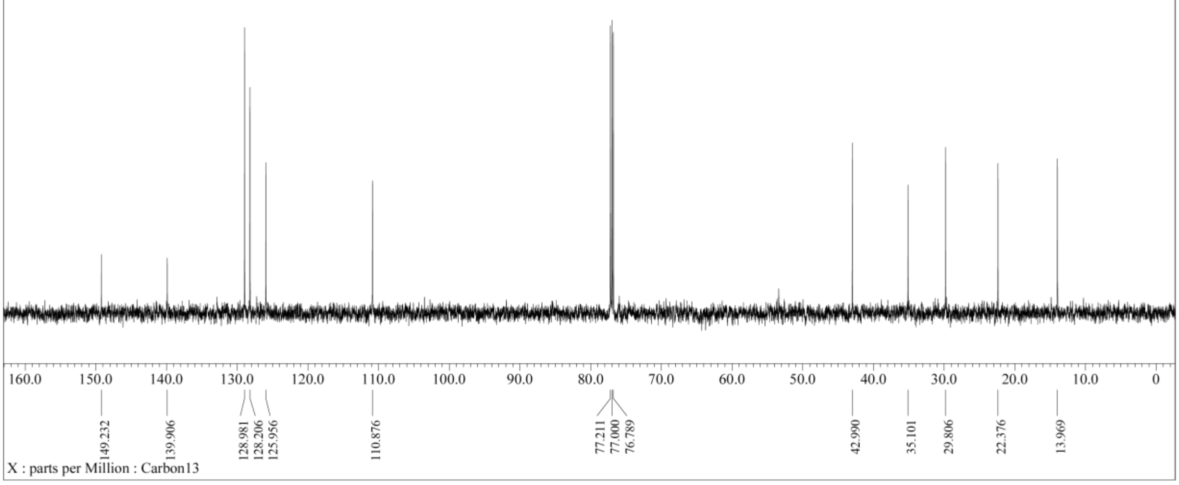

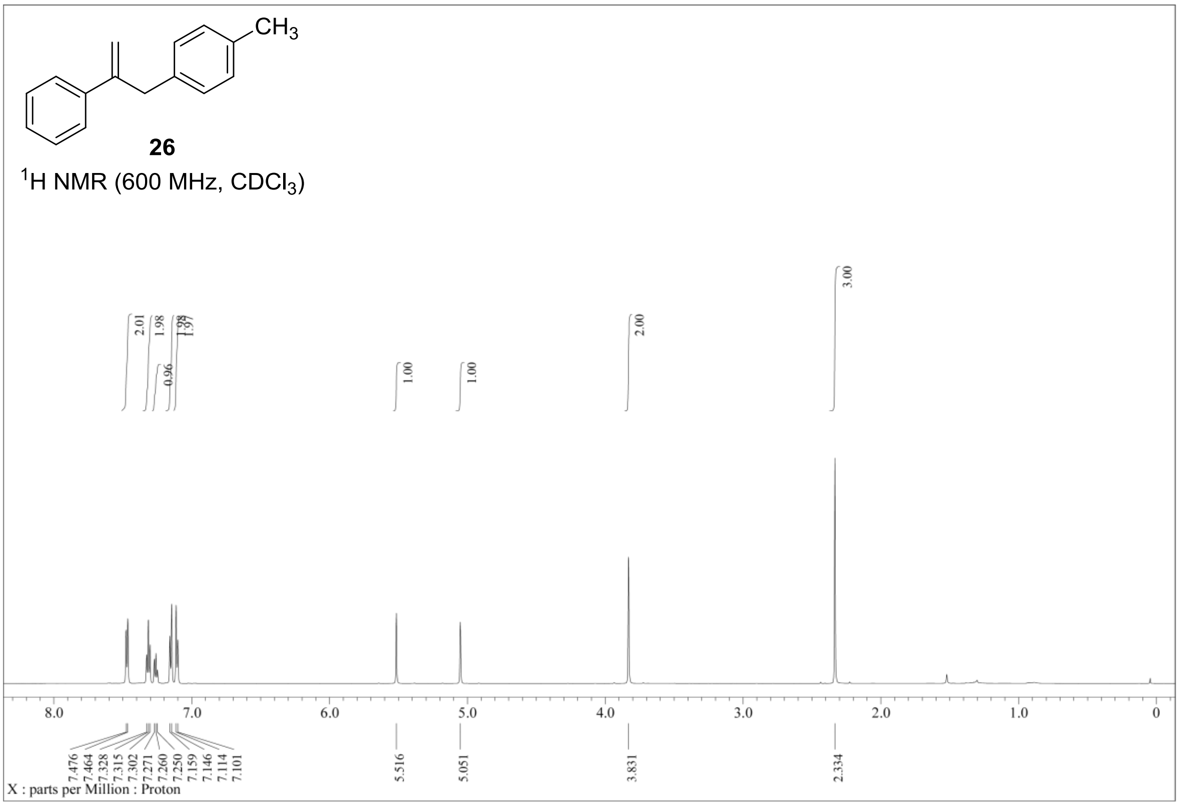
CH3
26
1H NMR (600 MHz, CDCl3)
2.01
1.98
0.96
1.98
1.97
1.00
1.00
2.00
3.00
8.0
7.0
6.0
5.0
4.0
3.0
2.0
1.0
0
7.476
7.464
7.328
7.315
7.302
7.271
7.260
7.250
7.159
7.146
7.114
7.101
5.516
5.051
3.831
2.334
X : parts per Million : Proton

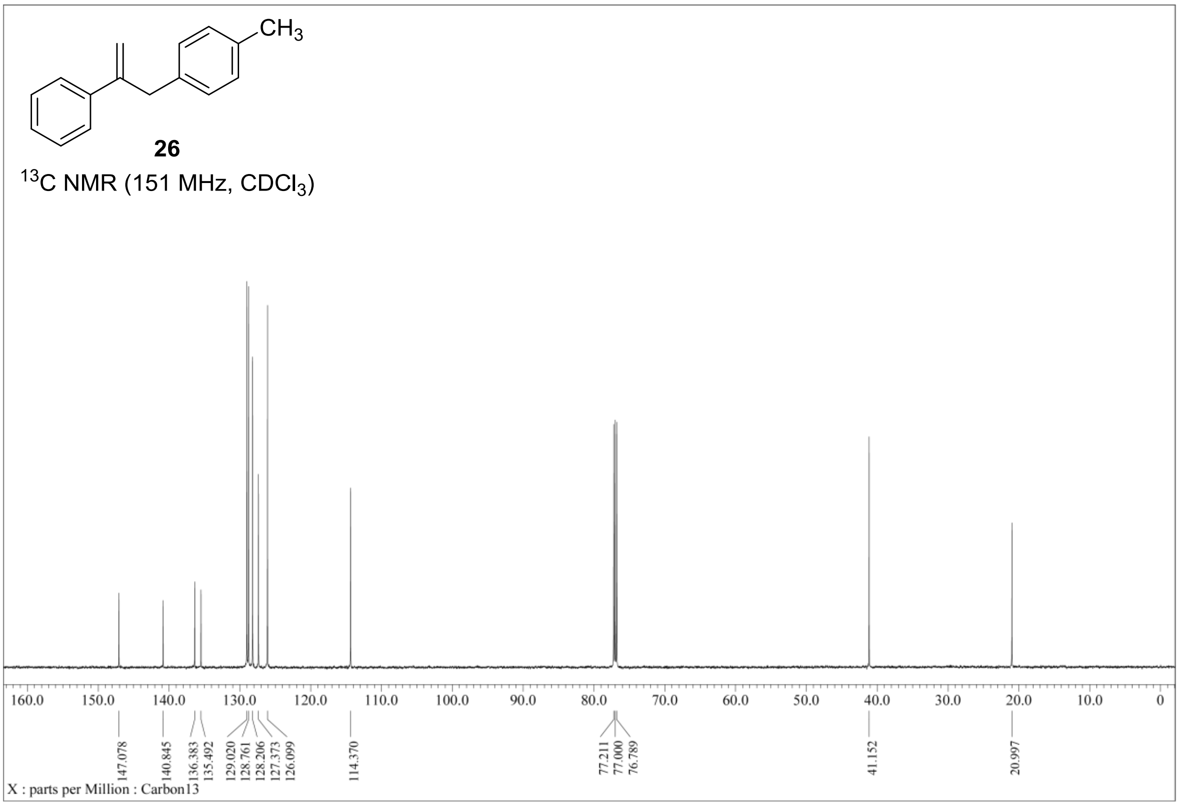
CH3
26
13C NMR (151 MHz, CDCl3)
160.0
150.0
140.0
130.0
120.0
110.0
100.0
90.0
80.0
70.0
60.0
50.0
40.0
30.0
20.0
10.0
0
147.078
140.845
136.383
135.492
129.020
128.761
128.206
127.373
126.099
114.370
77.211
77.000
76.789
41.152
20.997
X : parts per Million : Carbon13

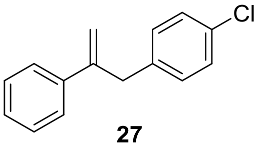

27

^{1}H NMR (600 MHz, $CDCl_3$)

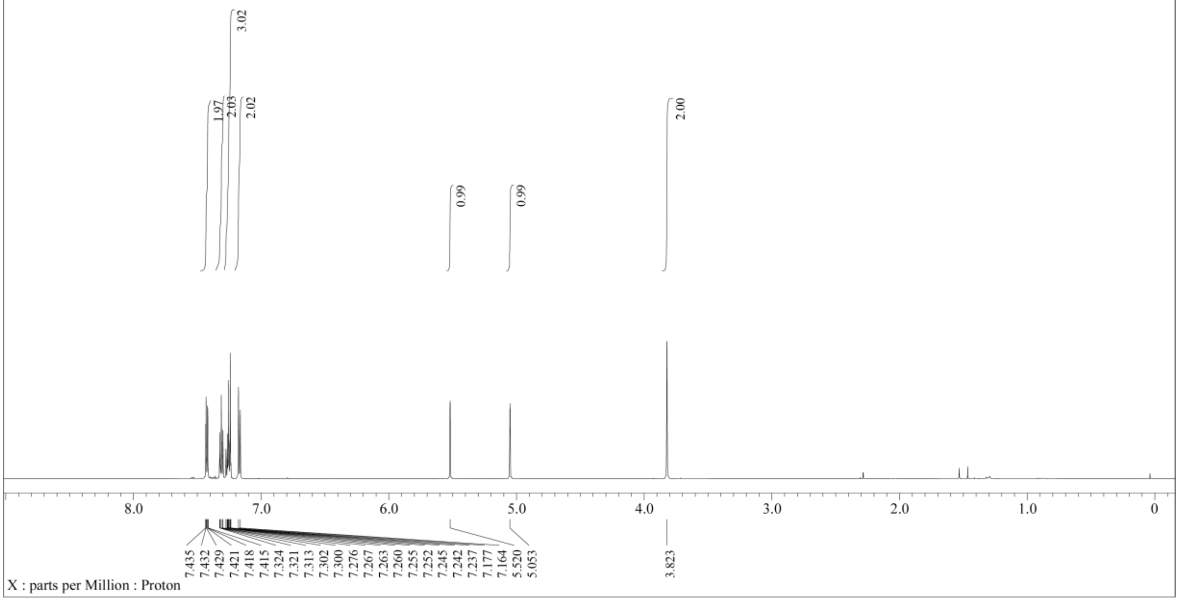

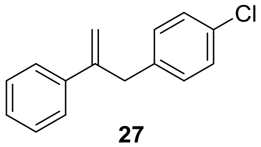

27

^{13}C NMR (151 MHz, $CDCl_3$)

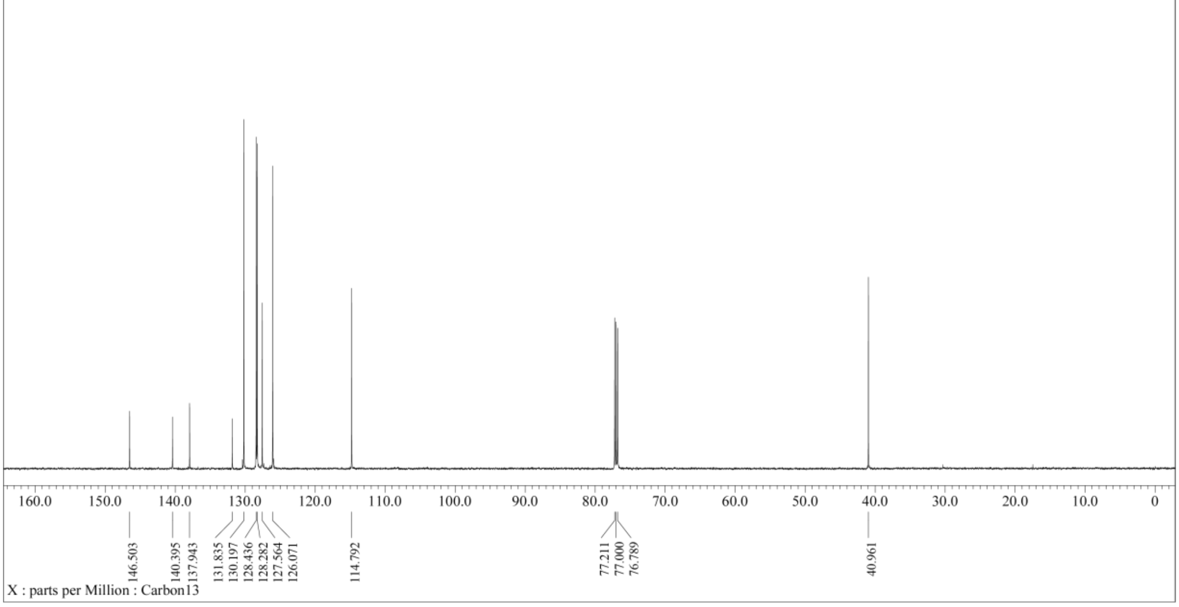

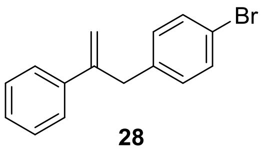

28

^{1}H NMR (600 MHz, $CDCl_3$)

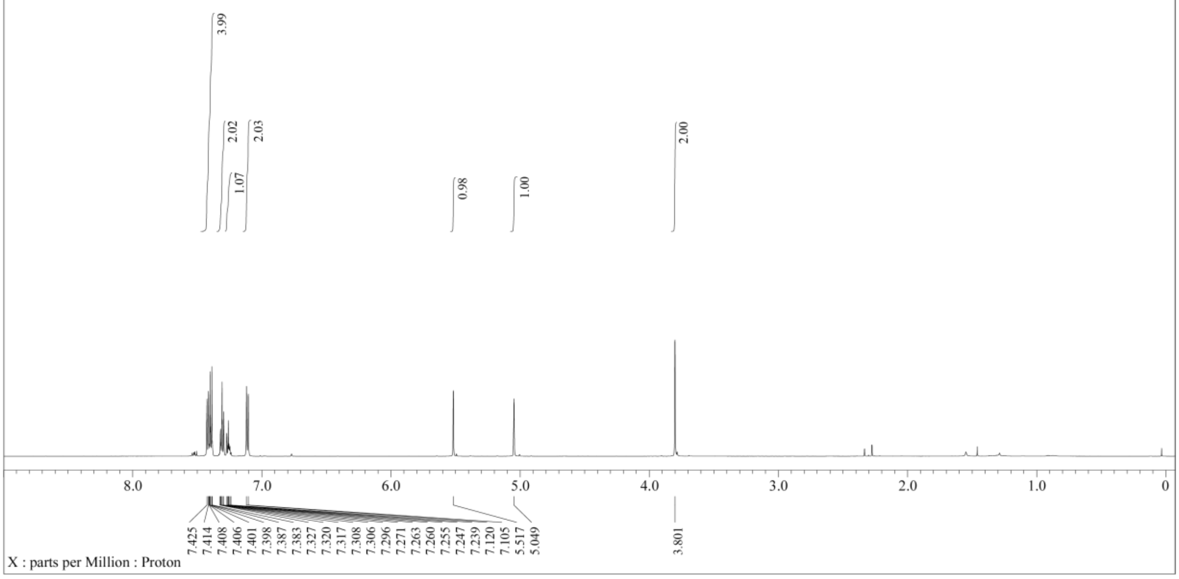

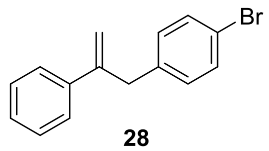

28

^{13}C NMR (151 MHz, $CDCl_3$)

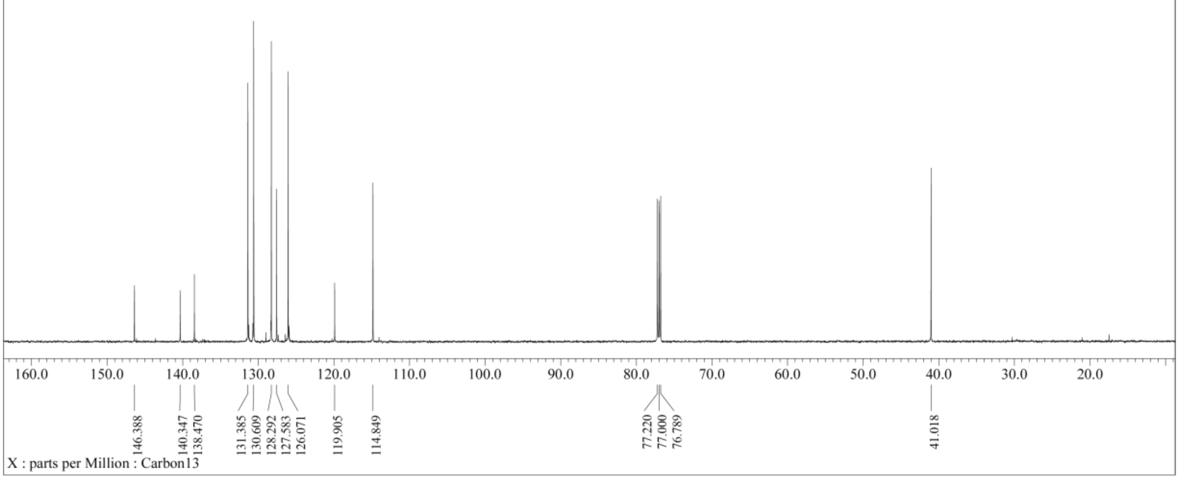

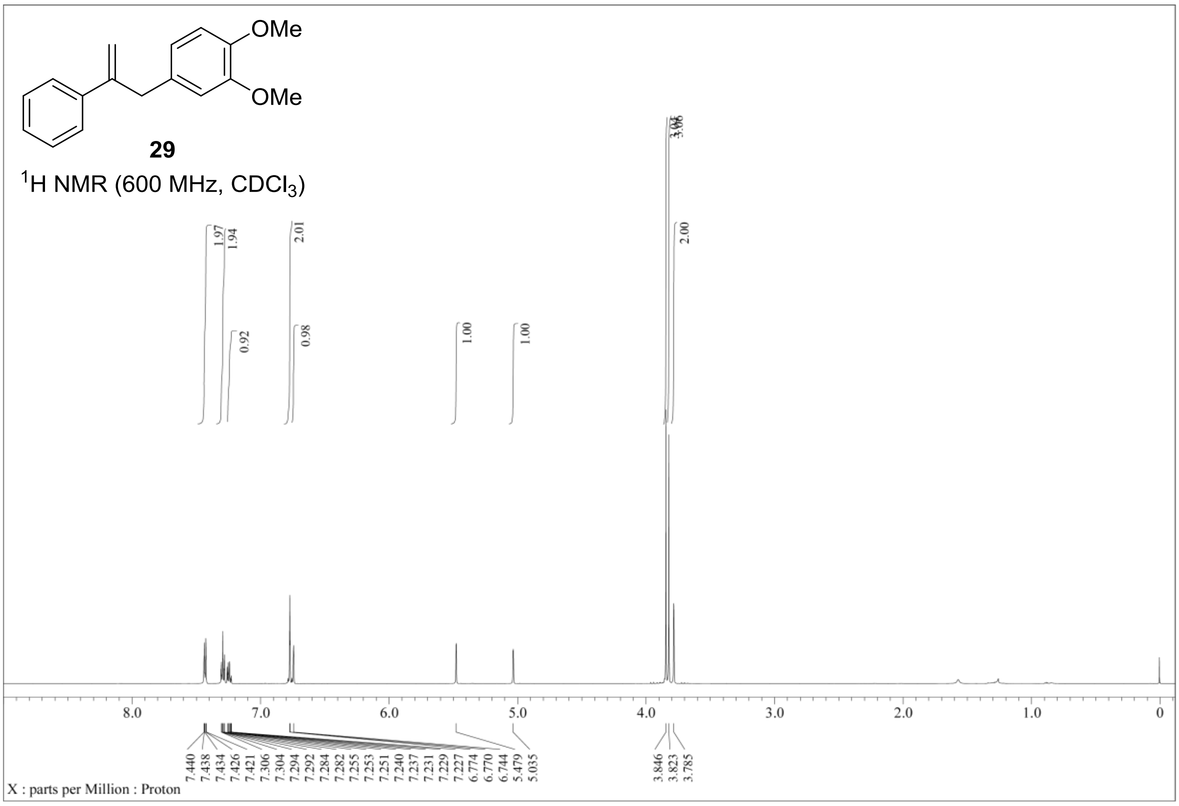
OMe
OMe
29
1H NMR (600 MHz, CDCl3)
1.97
1.94
0.92
2.01
0.98
1.00
1.00
2.00
8.0
7.0
6.0
5.0
4.0
3.0
2.0
1.0
0
7.440 7.438 7.434 7.426 7.421 7.306 7.304 7.294 7.292 7.284 7.282 7.255 7.253 7.251 7.240 7.237 7.231 7.229 7.227 6.774 6.770 6.744 5.479 5.035
3.846 3.823 3.785
X : parts per Million : Proton

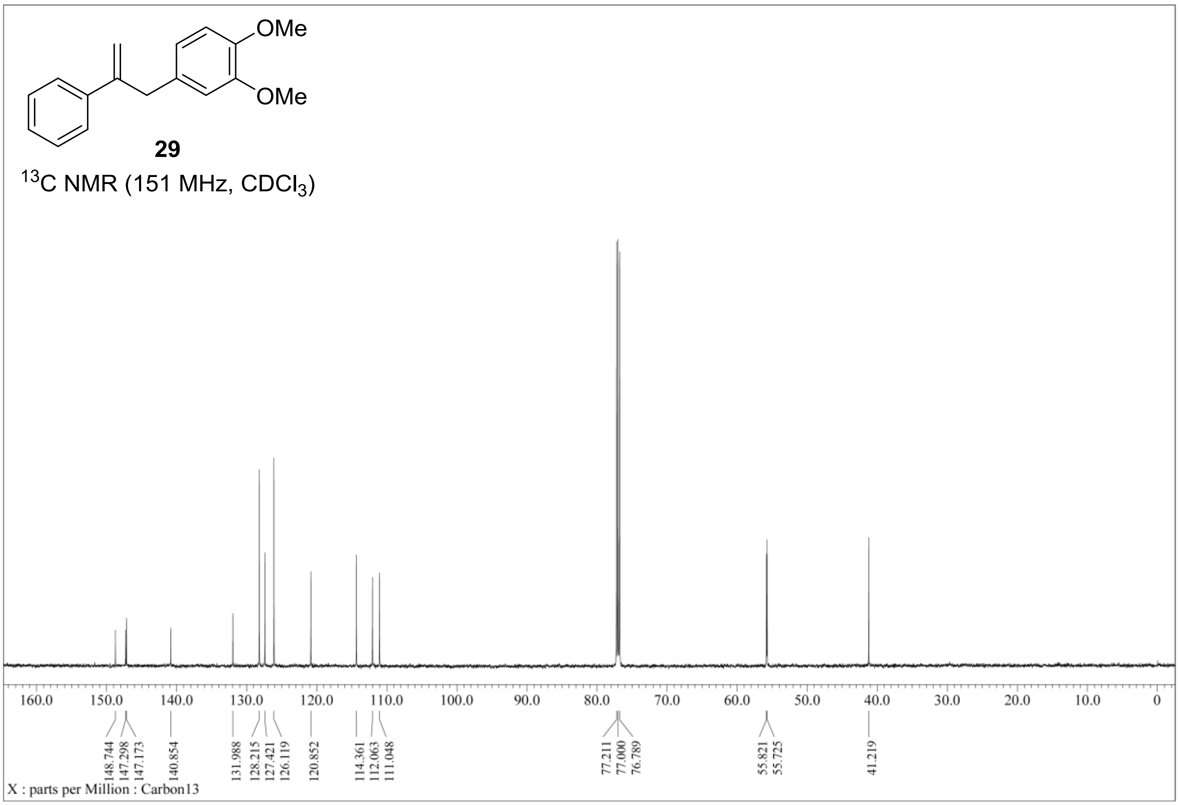
OMe
OMe
29
13C NMR (151 MHz, CDCl3)
160.0
150.0
140.0
130.0
120.0
110.0
100.0
90.0
80.0
70.0
60.0
50.0
40.0
30.0
20.0
10.0
0
148.744 147.298 147.173
140.854
131.988 128.215 127.421 126.119
120.852
114.361 112.063 111.048
77.211 77.000 76.789
55.821 55.725
41.219
X : parts per Million : Carbon13

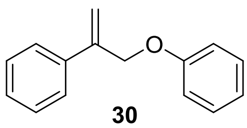

30

^{1}H NMR (600 MHz, $CDCl_3$)

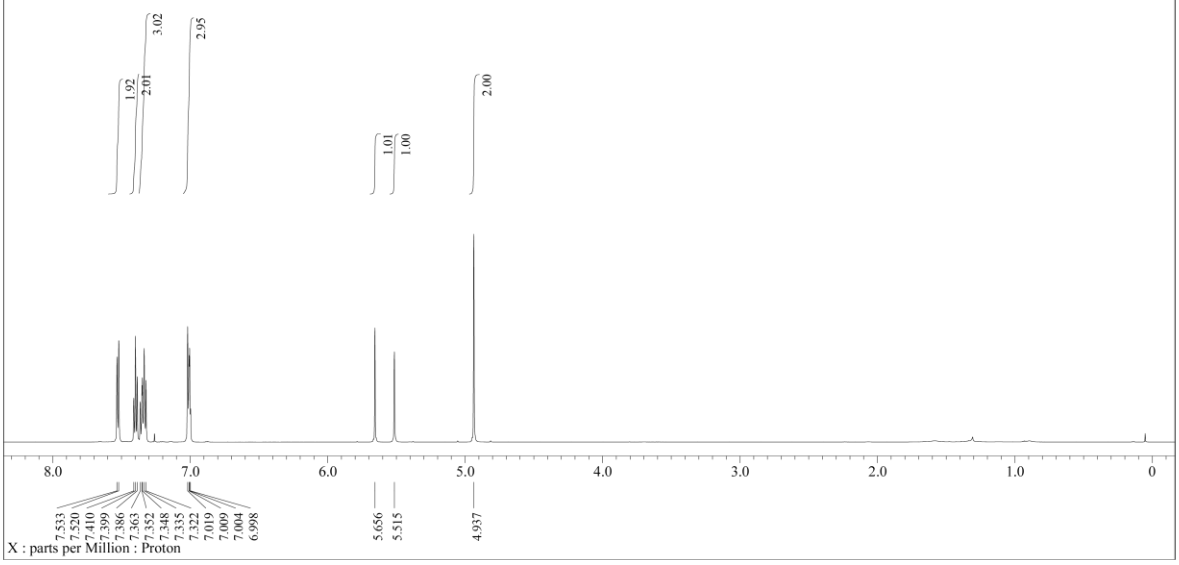

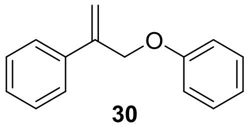

30

^{13}C NMR (151 MHz, $CDCl_3$)

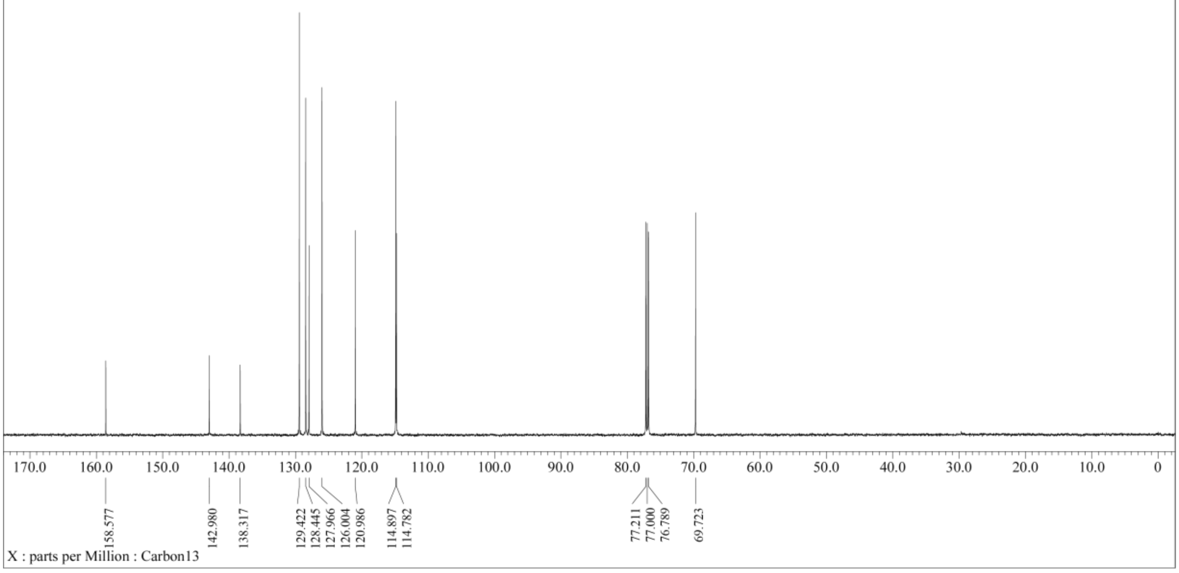

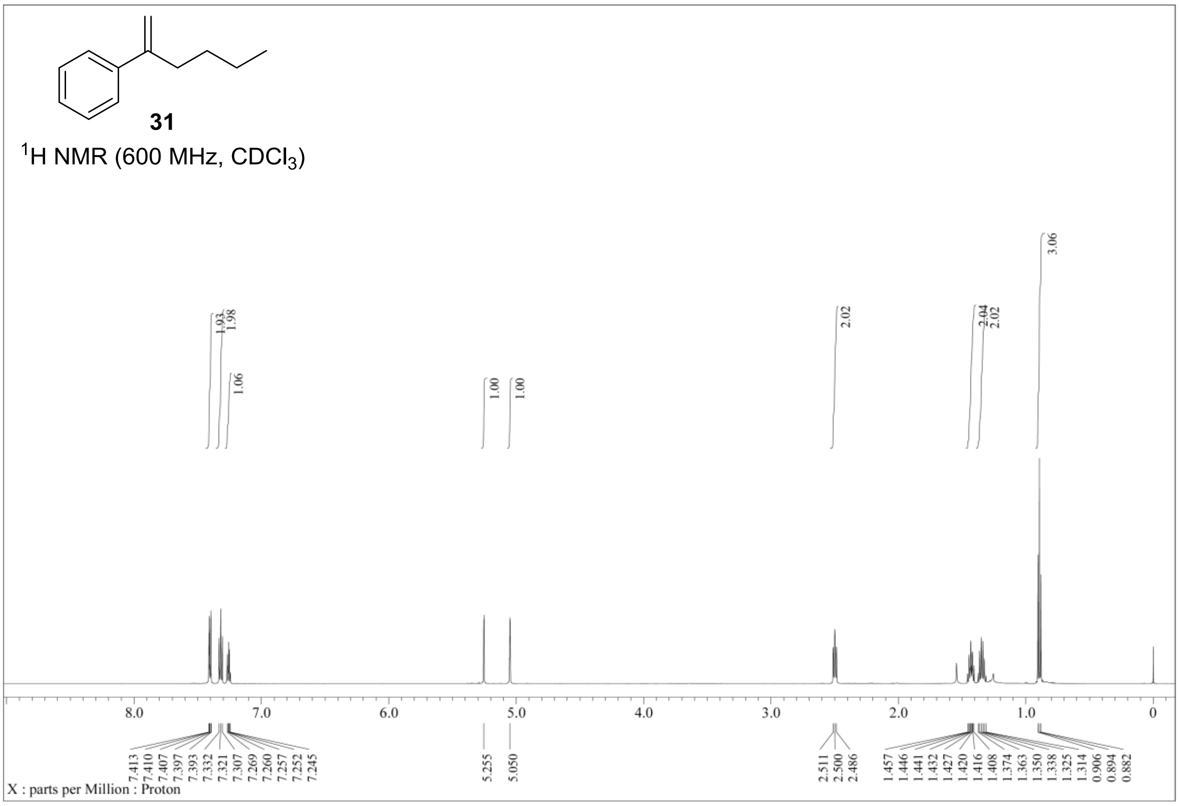
31
^{1}H NMR (600 MHz, $CDCl_3$)
1.93
1.98
1.06
1.00
1.00
2.02
2.04
2.02
3.06
8.0 7.0 6.0 5.0 4.0 3.0 2.0 1.0 0
7.413 7.410 7.407 7.397 7.393 7.332 7.321 7.307 7.269 7.260 7.257 7.252 7.245
5.255
5.050
2.511 2.500 2.486
1.457 1.446 1.441 1.432 1.427 1.420 1.416 1.408 1.374 1.363 1.350 1.338 1.325 1.314 0.906 0.894 0.882
X : parts per Million : Proton

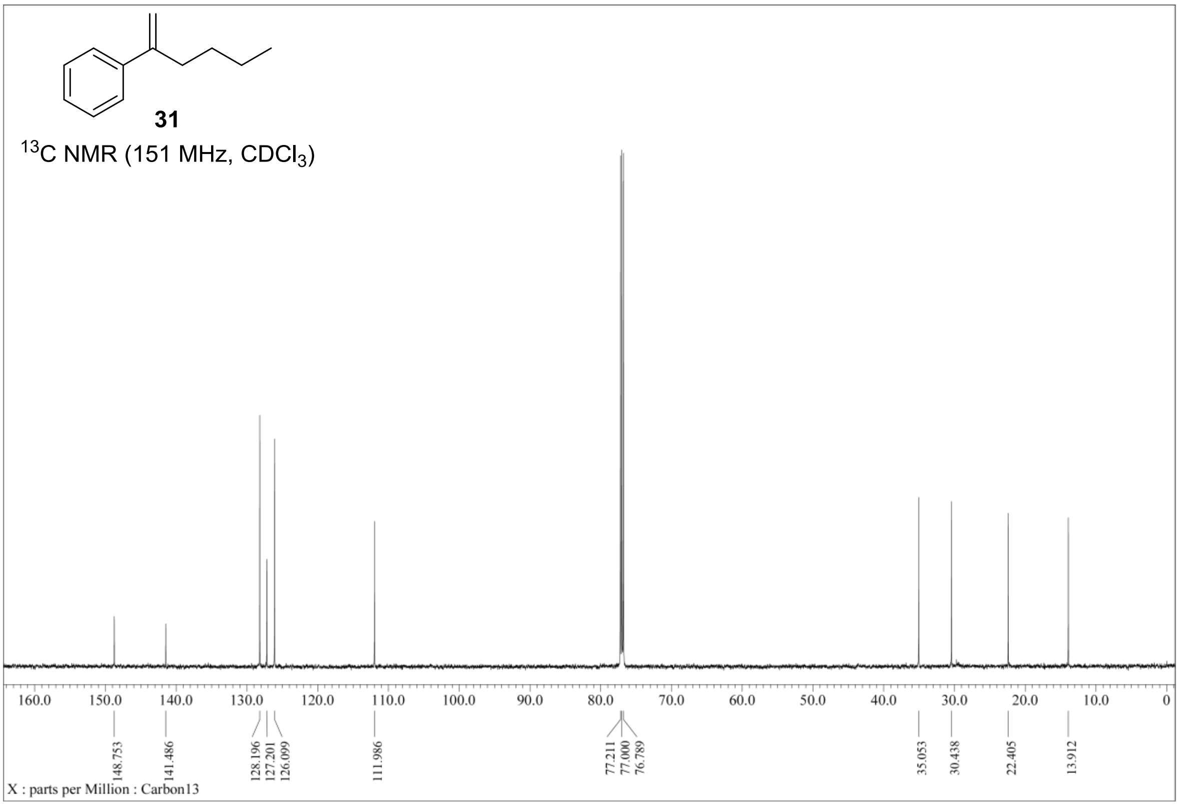
31
^{13}C NMR (151 MHz, $CDCl_3$)
160.0 150.0 140.0 130.0 120.0 110.0 100.0 90.0 80.0 70.0 60.0 50.0 40.0 30.0 20.0 10.0 0
148.753
141.486
128.196 127.201 126.099
111.986
77.211 77.000 76.789
35.053
30.438
22.405
13.912
X : parts per Million : Carbon13

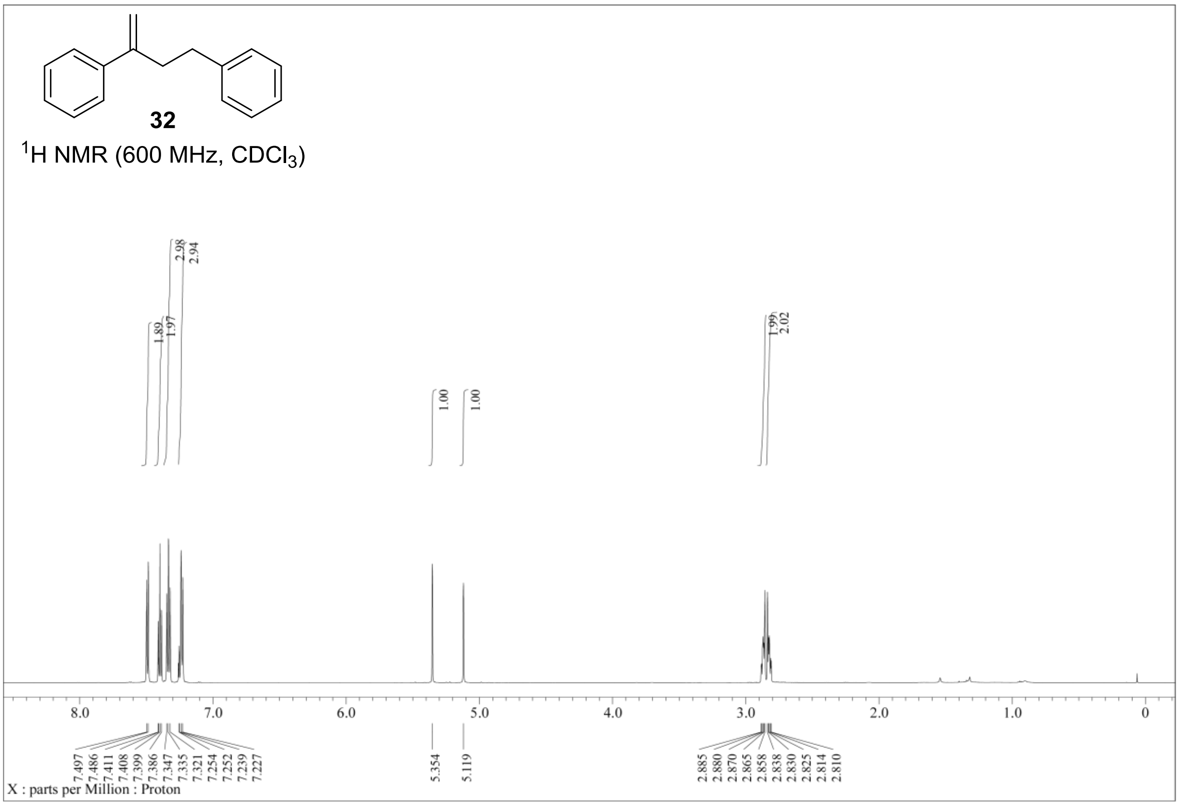

32

^{1}H NMR (600 MHz, $CDCl_3$)

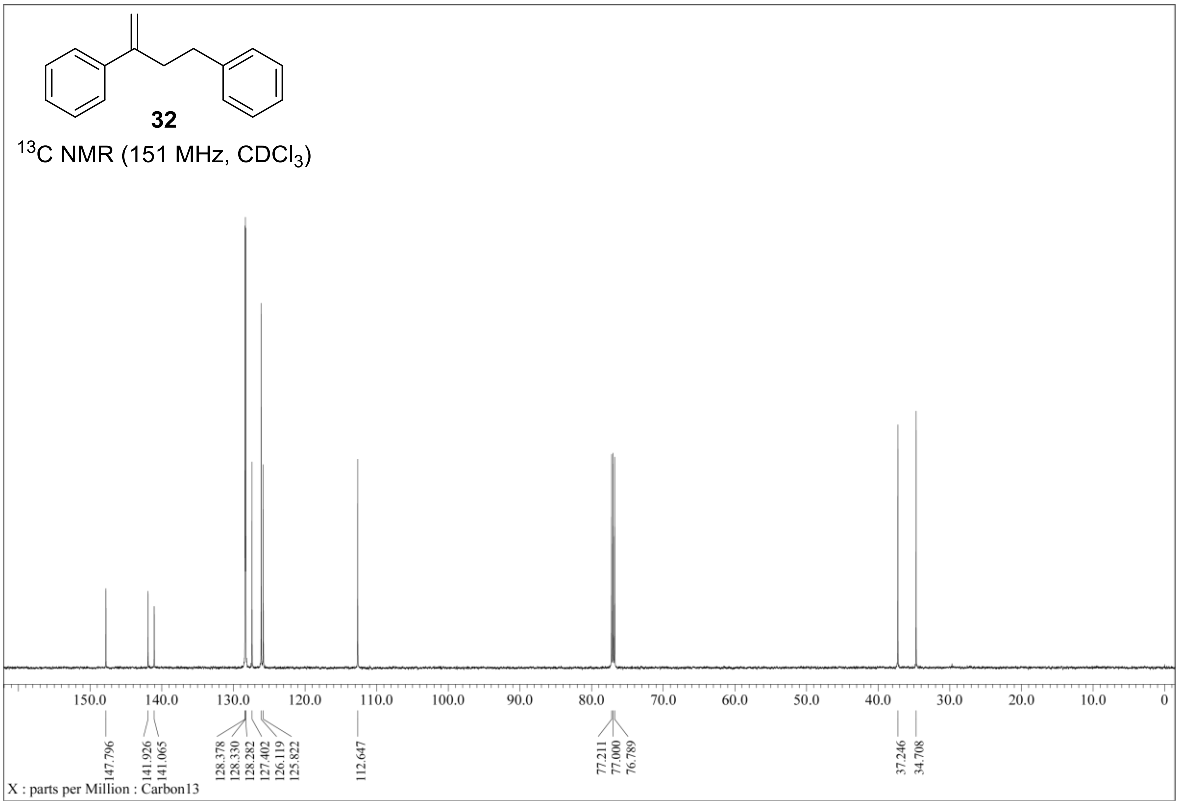

32

^{13}C NMR (151 MHz, $CDCl_3$)

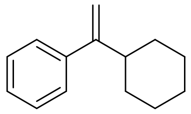

33

^{1}H NMR (600 MHz, $CDCl_3$)

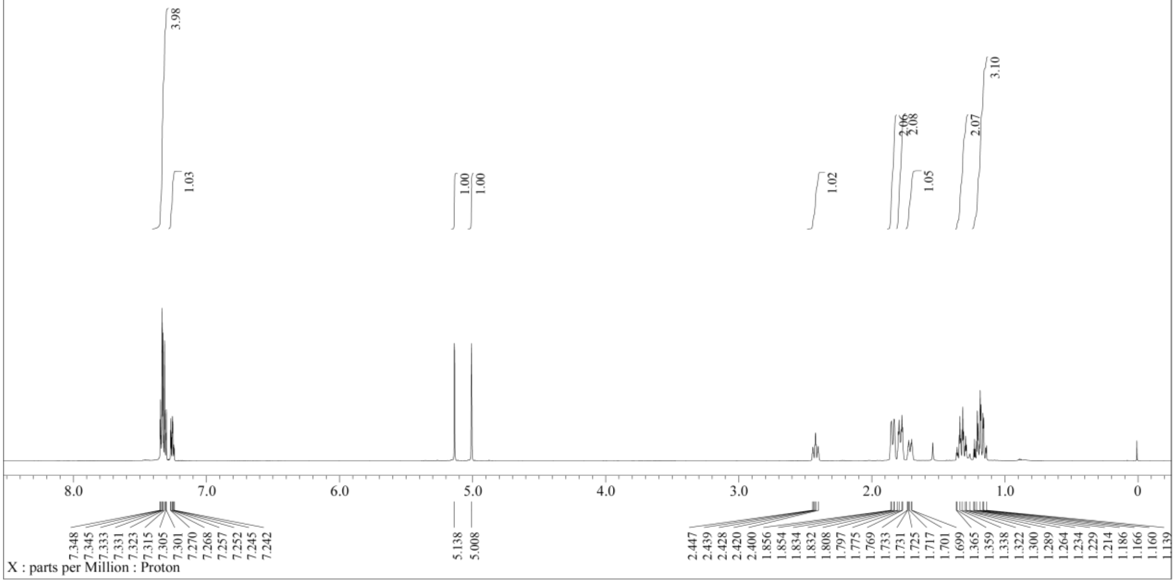

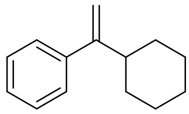

33

^{13}C NMR (151 MHz, $CDCl_3$)

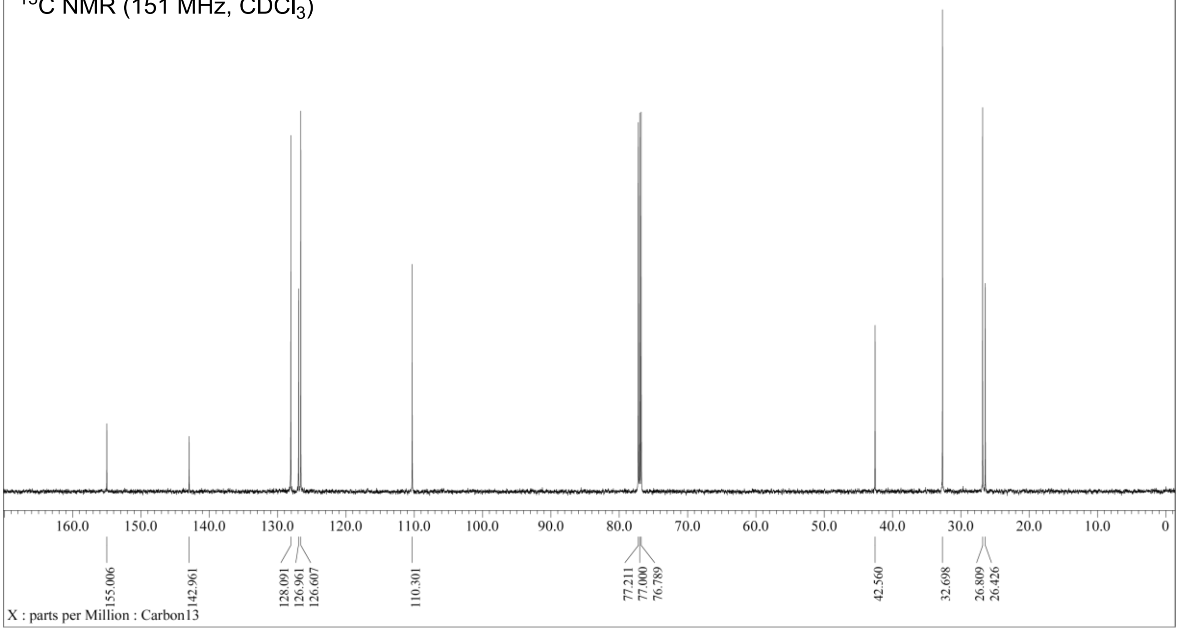

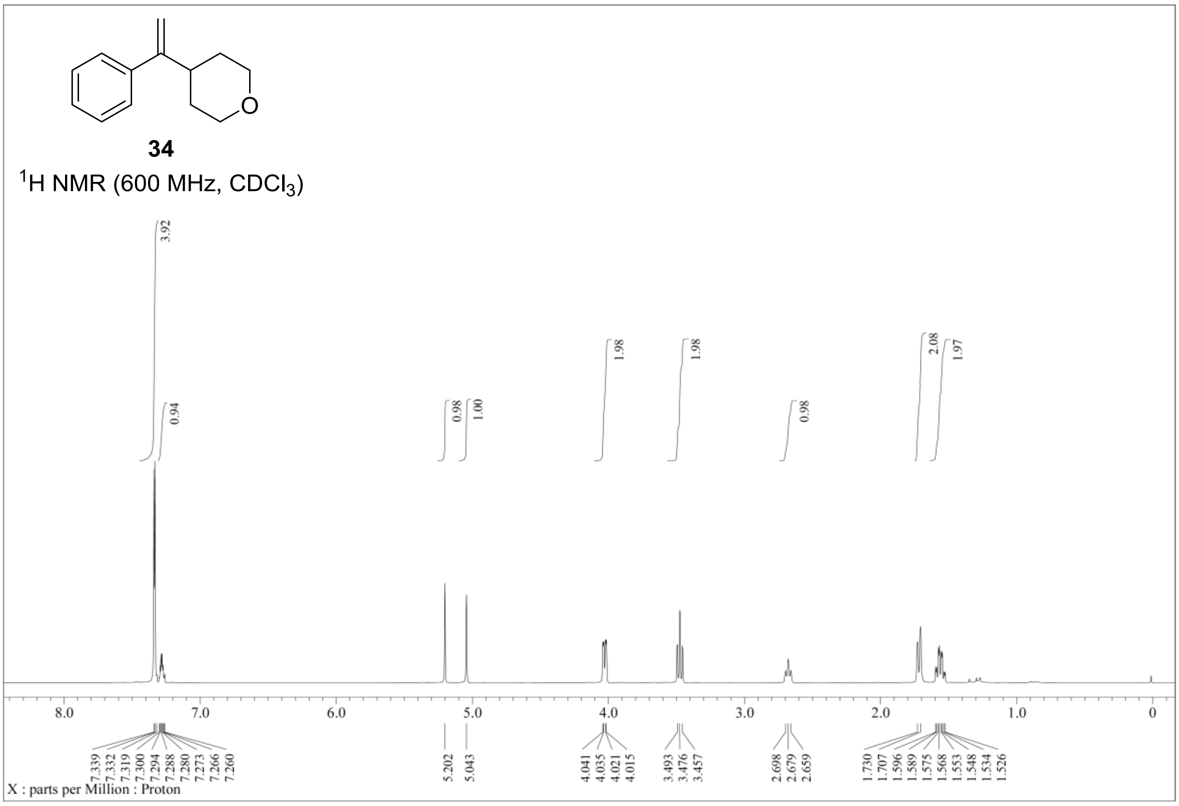
34
^{1}H NMR (600 MHz, CDCl$_3$)
X : parts per Million : Proton

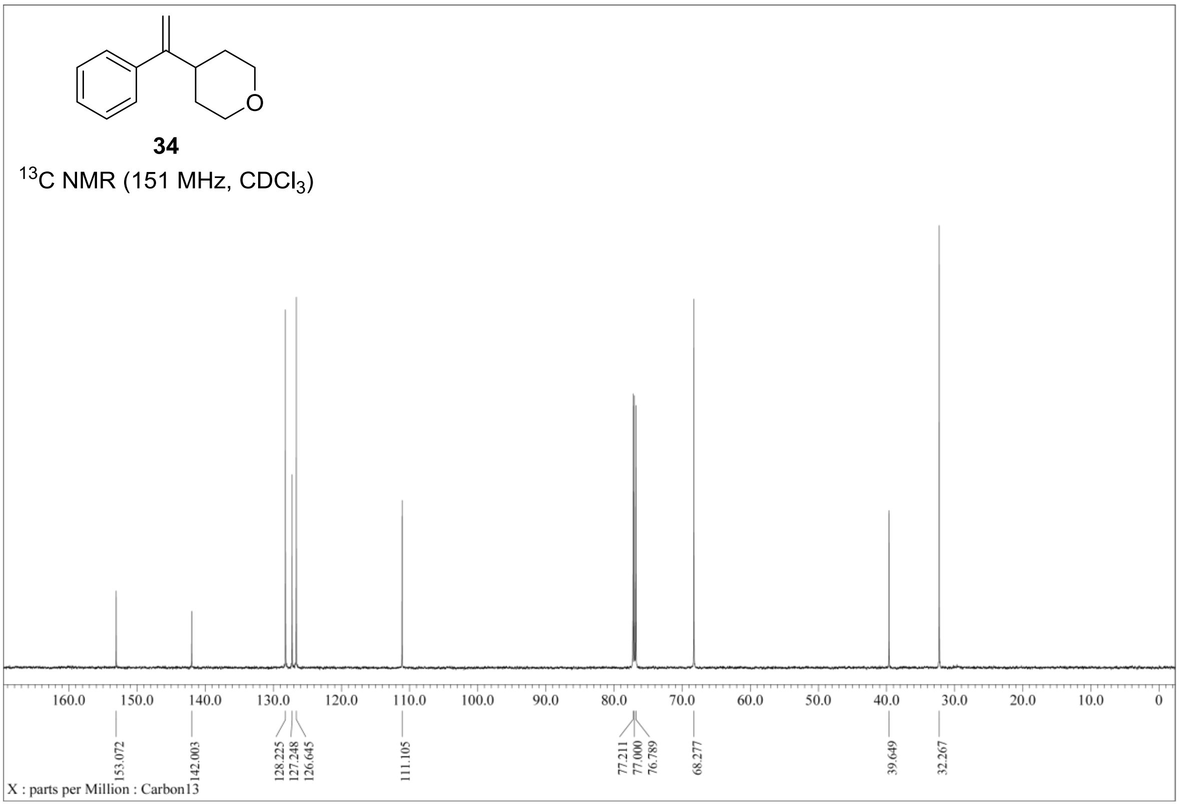
34
^{13}C NMR (151 MHz, CDCl$_3$)
X : parts per Million : Carbon13

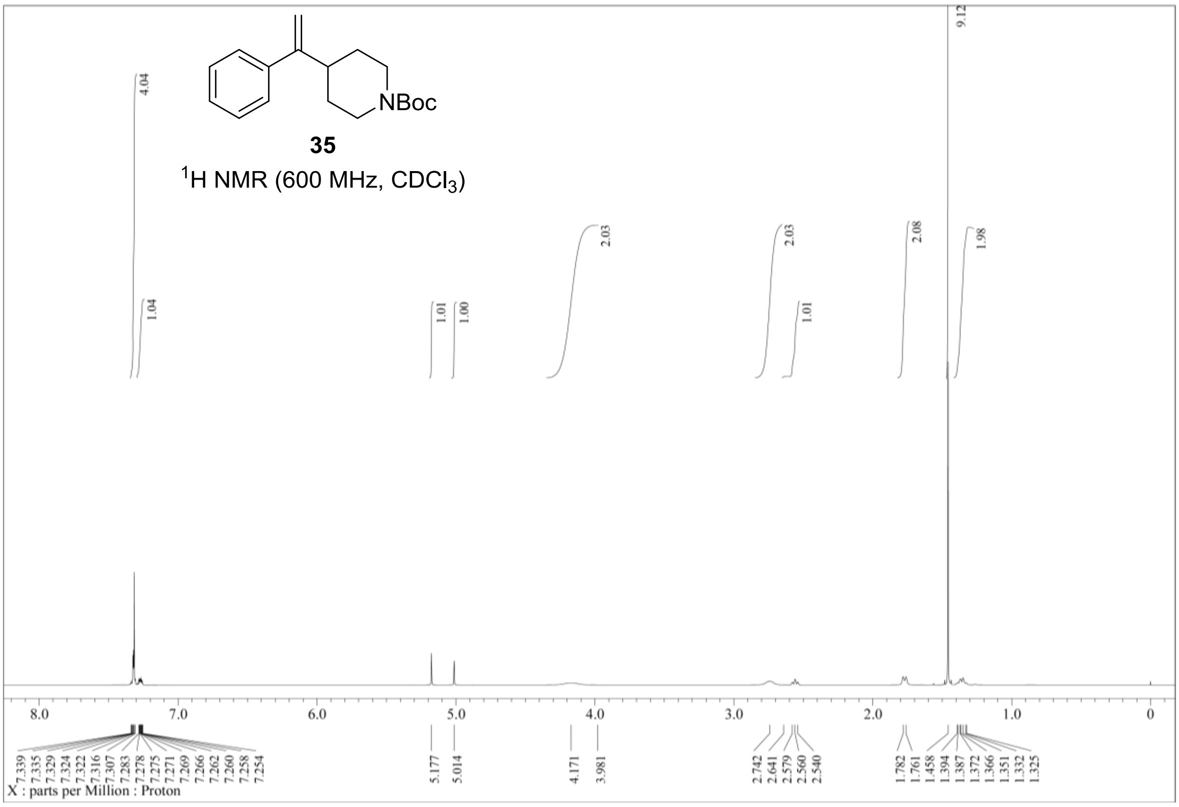
NBoc
35
1H NMR (600 MHz, CDCl3)
X : parts per Million : Proton

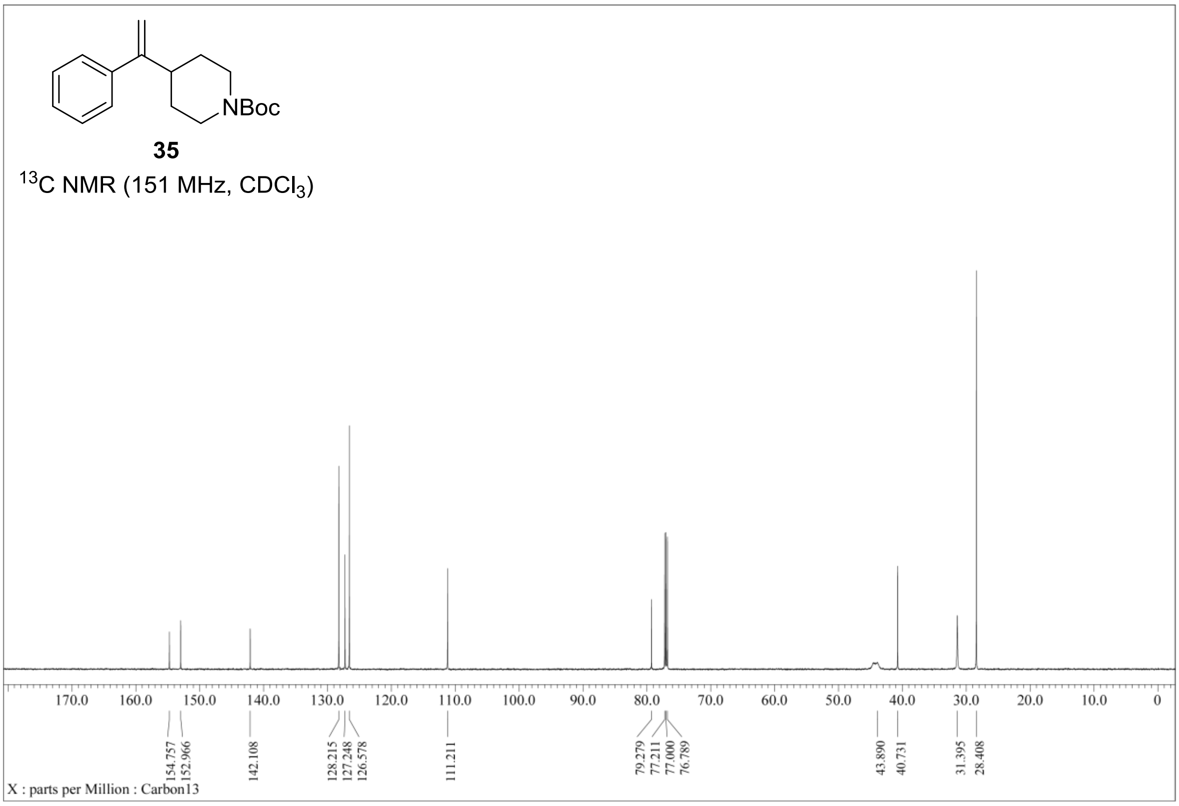
NBoc
35
13C NMR (151 MHz, CDCl3)
X : parts per Million : Carbon13

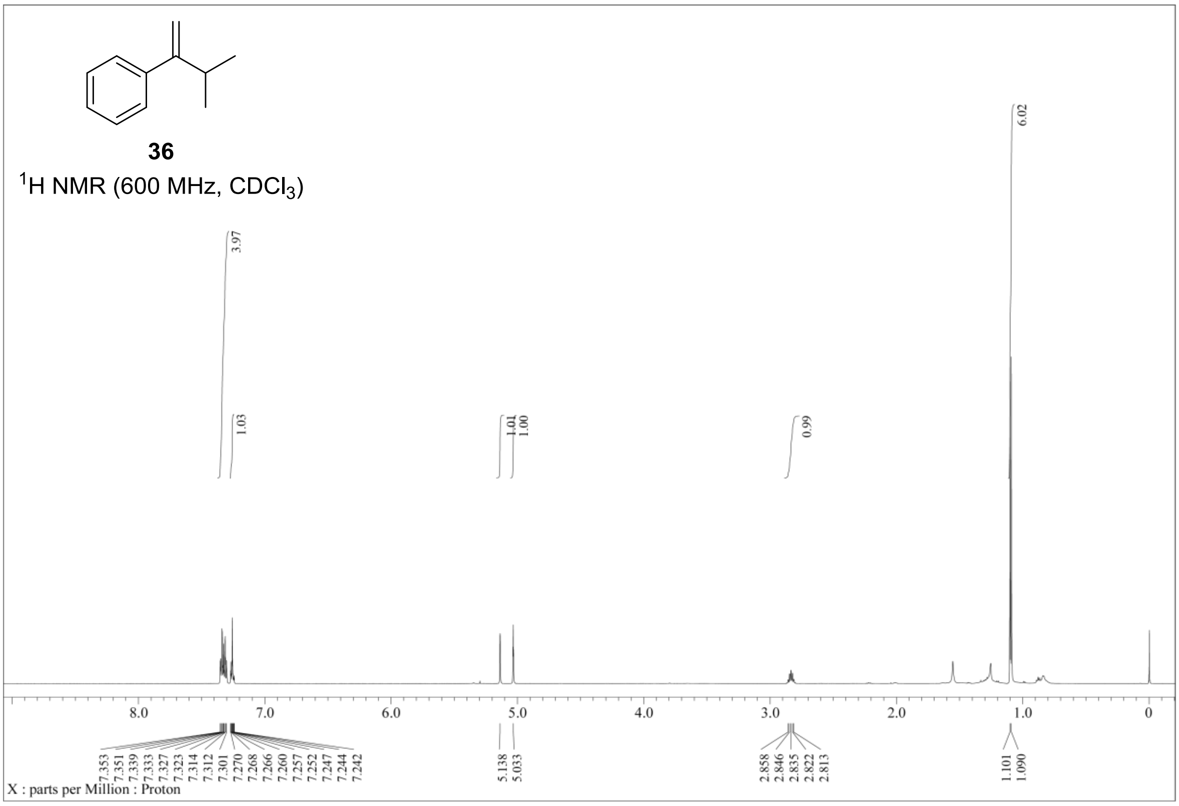
36
1H NMR (600 MHz, CDCl3)
3.97
1.03
1.01
1.00
0.99
6.02
8.0
7.0
6.0
5.0
4.0
3.0
2.0
1.0
0
7.353
7.351
7.339
7.333
7.327
7.323
7.314
7.312
7.301
7.270
7.268
7.266
7.260
7.257
7.252
7.247
7.244
7.242
5.138
5.033
2.858
2.846
2.835
2.822
2.813
1.101
1.090
X : parts per Million : Proton

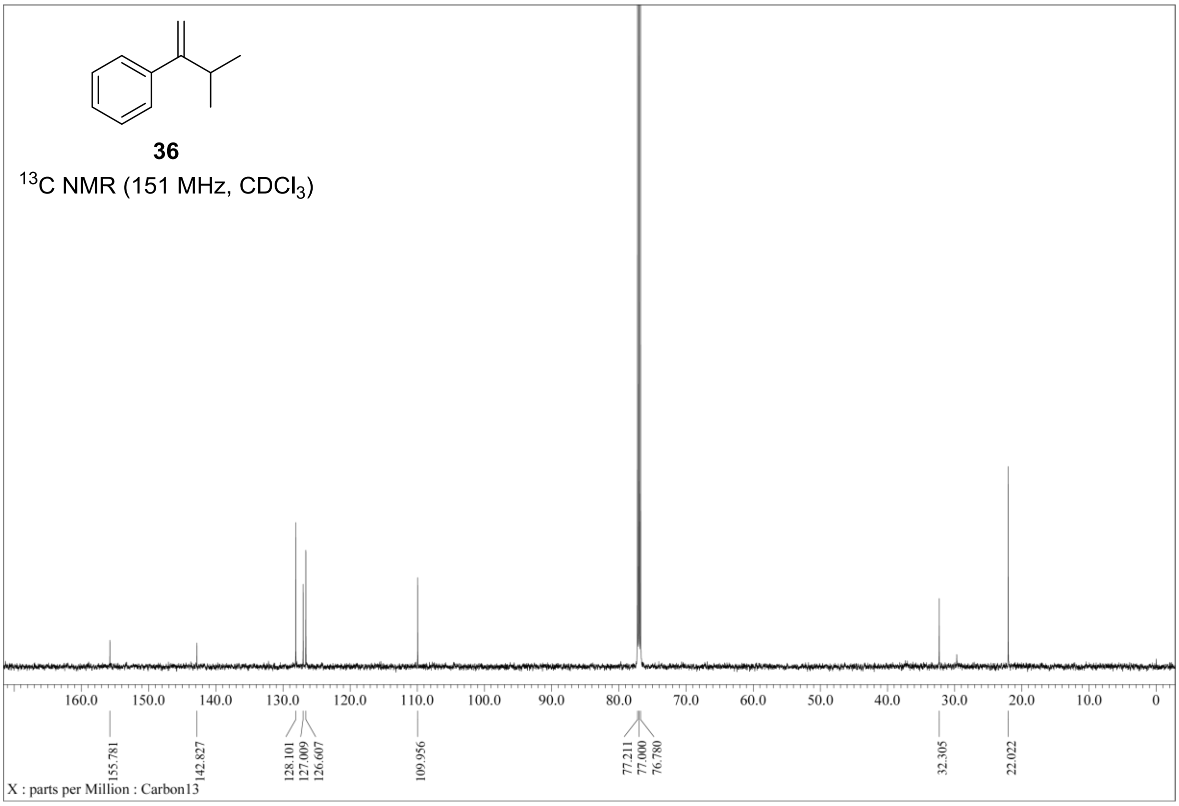
36
13C NMR (151 MHz, CDCl3)
160.0
150.0
140.0
130.0
120.0
110.0
100.0
90.0
80.0
70.0
60.0
50.0
40.0
30.0
20.0
10.0
0
155.781
142.827
128.101
127.009
126.607
109.956
77.211
77.000
76.780
32.305
22.022
X : parts per Million : Carbon13

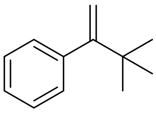

37

^{1}H NMR (600 MHz, $CDCl_3$)

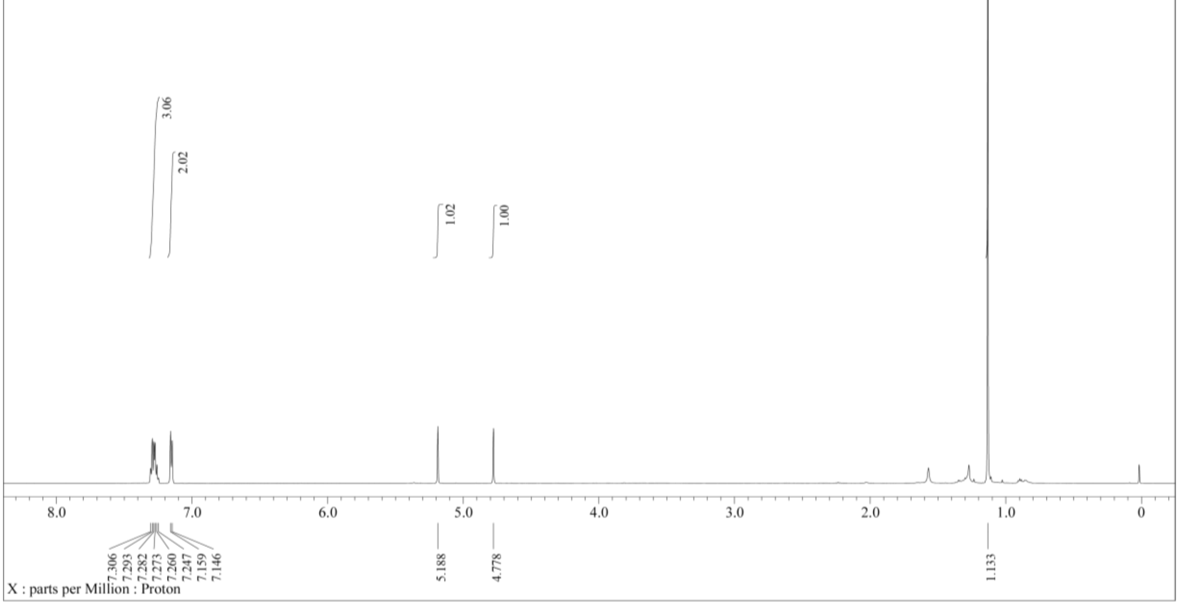

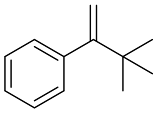

37

^{13}C NMR (151 MHz, $CDCl_3$)

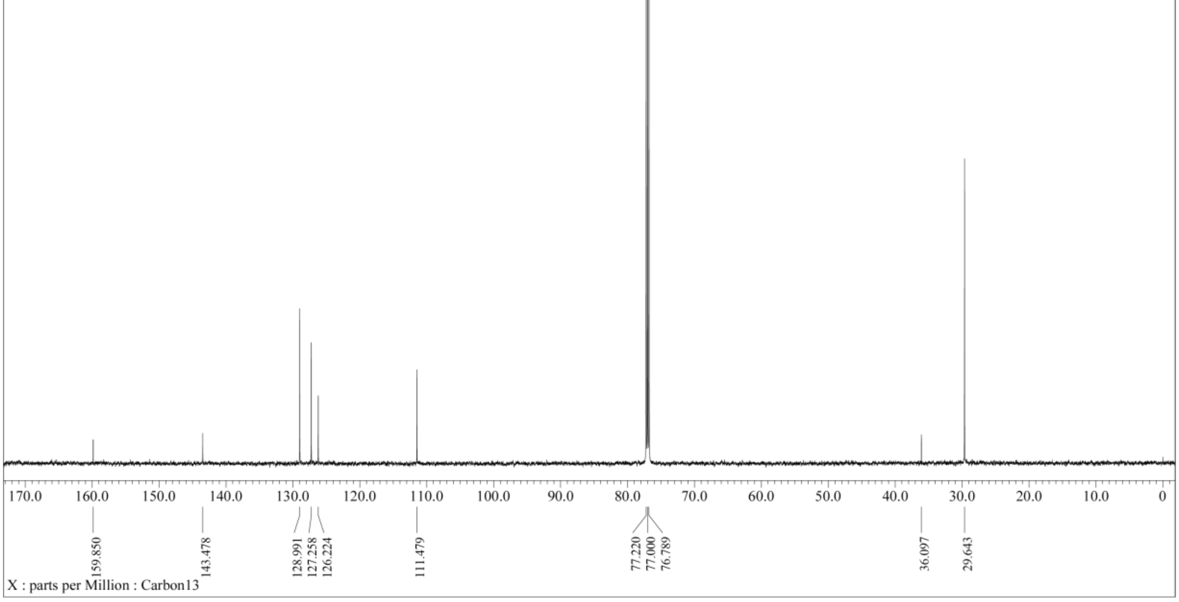

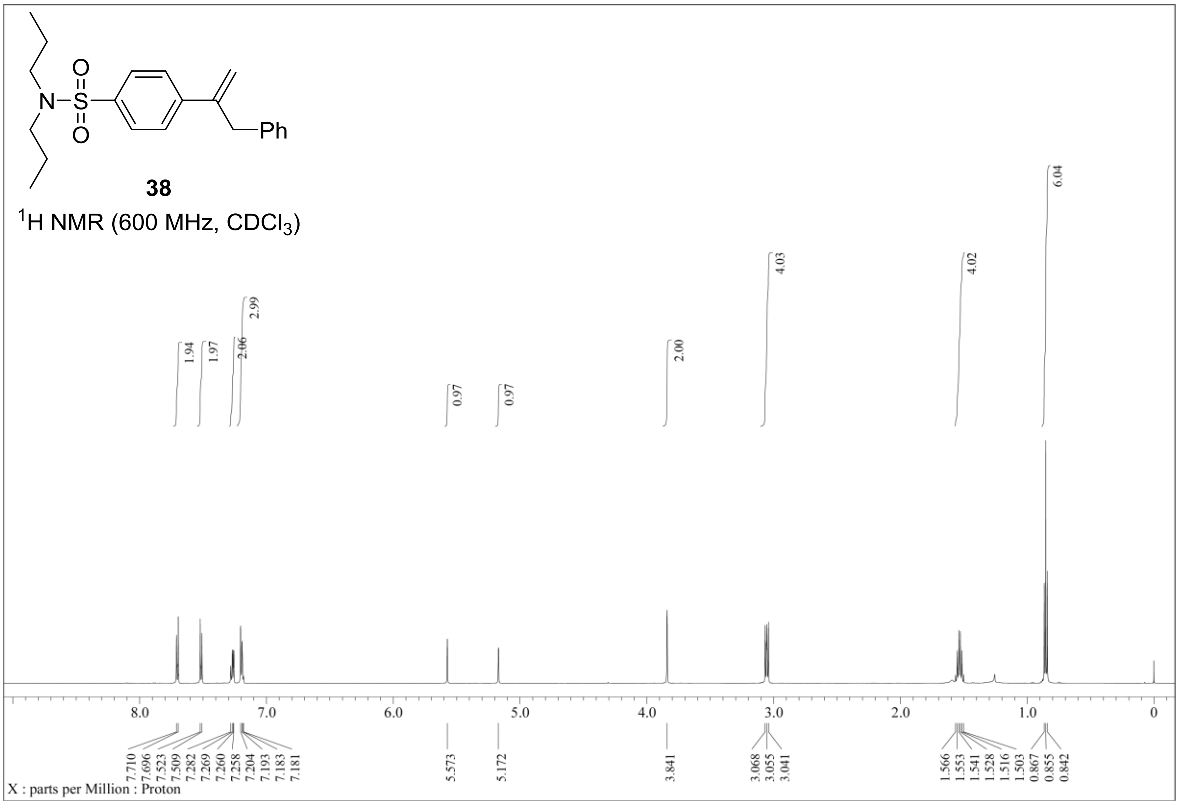
Ph
38
1H NMR (600 MHz, CDCl3)
1.94 1.97 2.06 2.99 0.97 0.97 2.00 4.03 4.02 6.04
7.710 7.696 7.523 7.509 7.282 7.269 7.260 7.258 7.204 7.193 7.183 7.181
5.573
5.172
3.841
3.068 3.055 3.041
1.566 1.553 1.541 1.528 1.516 1.503 0.867 0.855 0.842
X : parts per Million : Proton

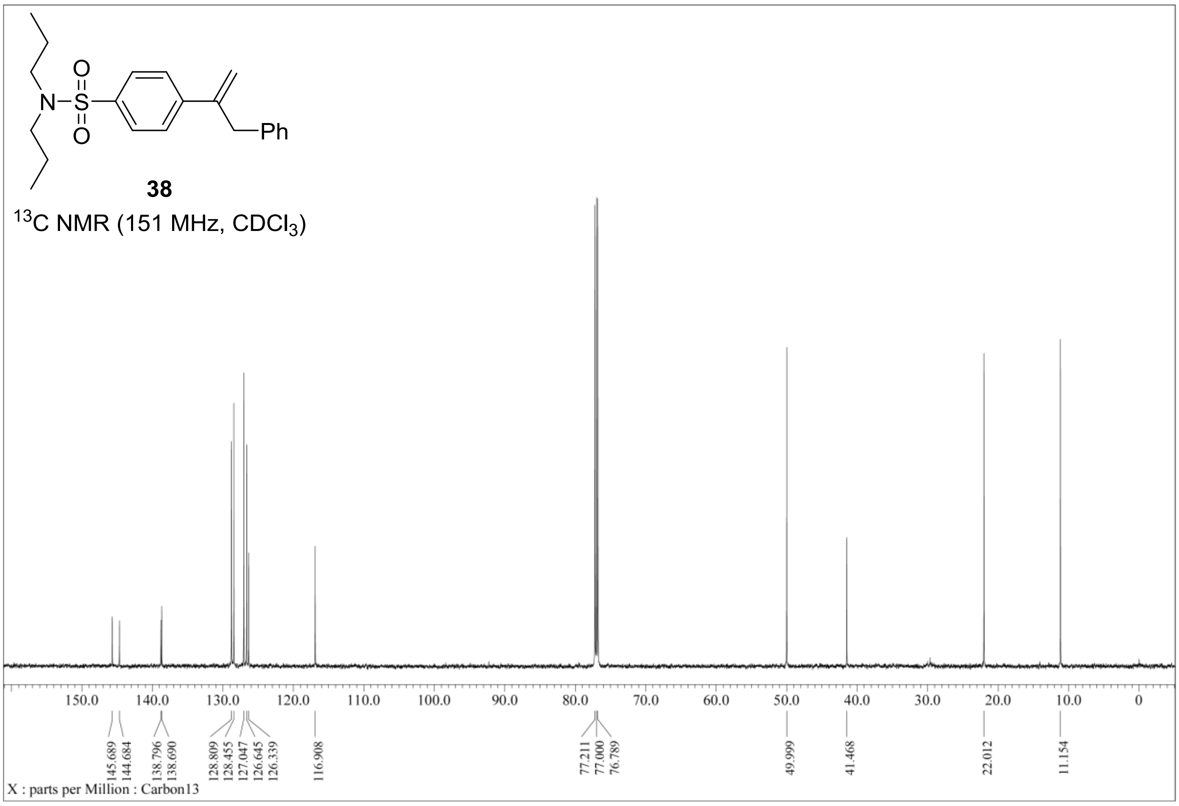
Ph
38
13C NMR (151 MHz, CDCl3)
145.689 144.684 138.796 138.690 128.809 128.455 127.047 126.645 126.339 116.908
77.211 77.000 76.789
49.999 41.468 22.012 11.154
X : parts per Million : Carbon13

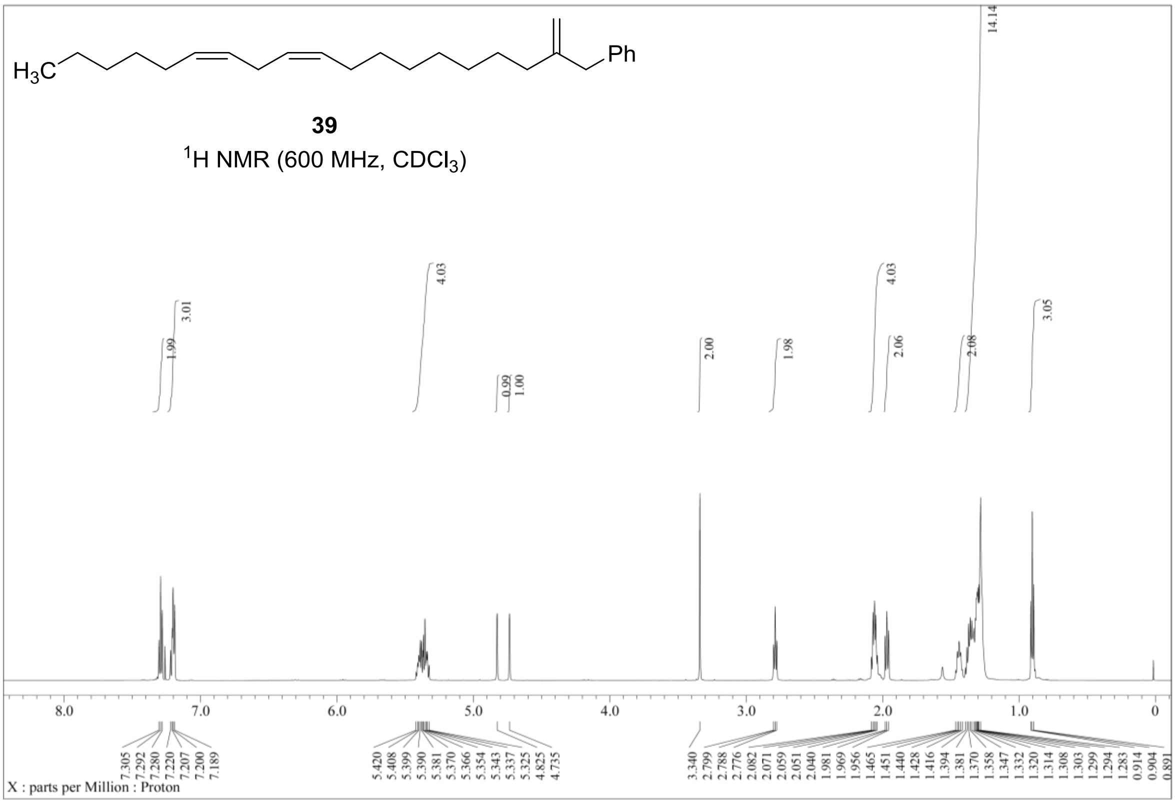
H3C
Ph
39
1H NMR (600 MHz, CDCl3)
X : parts per Million : Proton

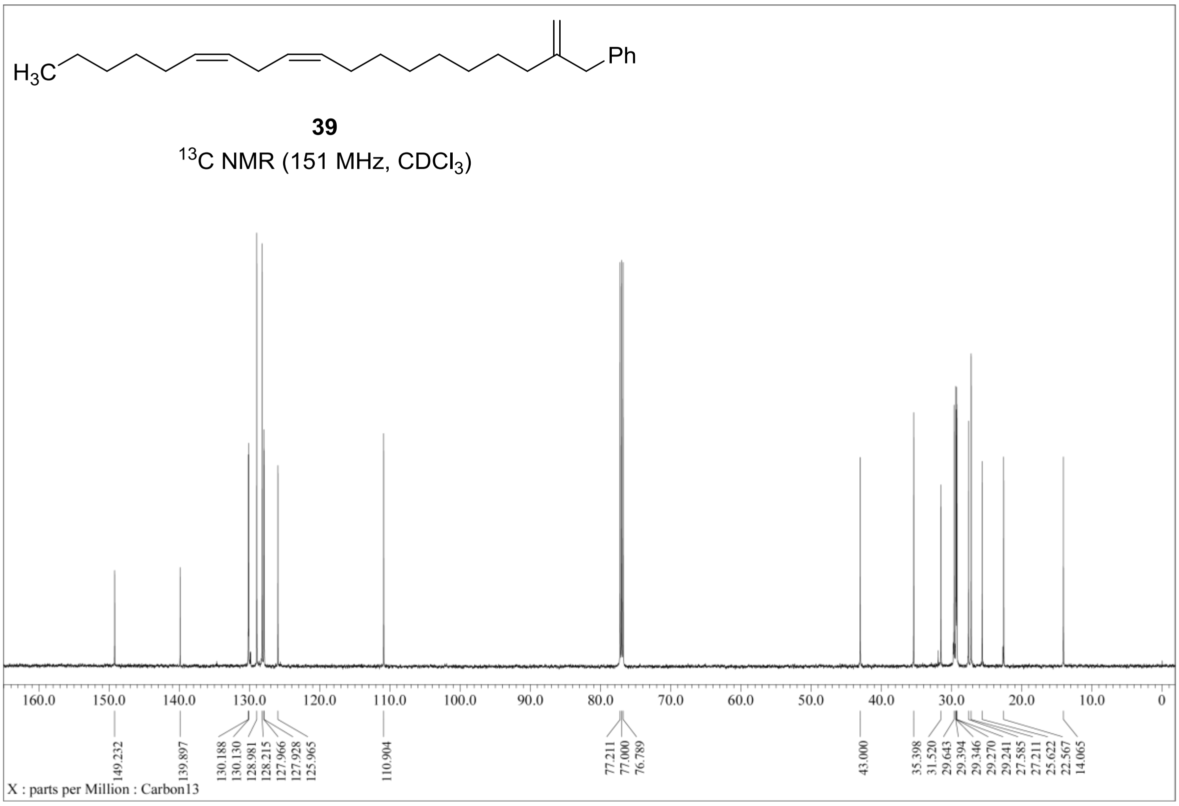
H3C
Ph
39
13C NMR (151 MHz, CDCl3)
X : parts per Million : Carbon13

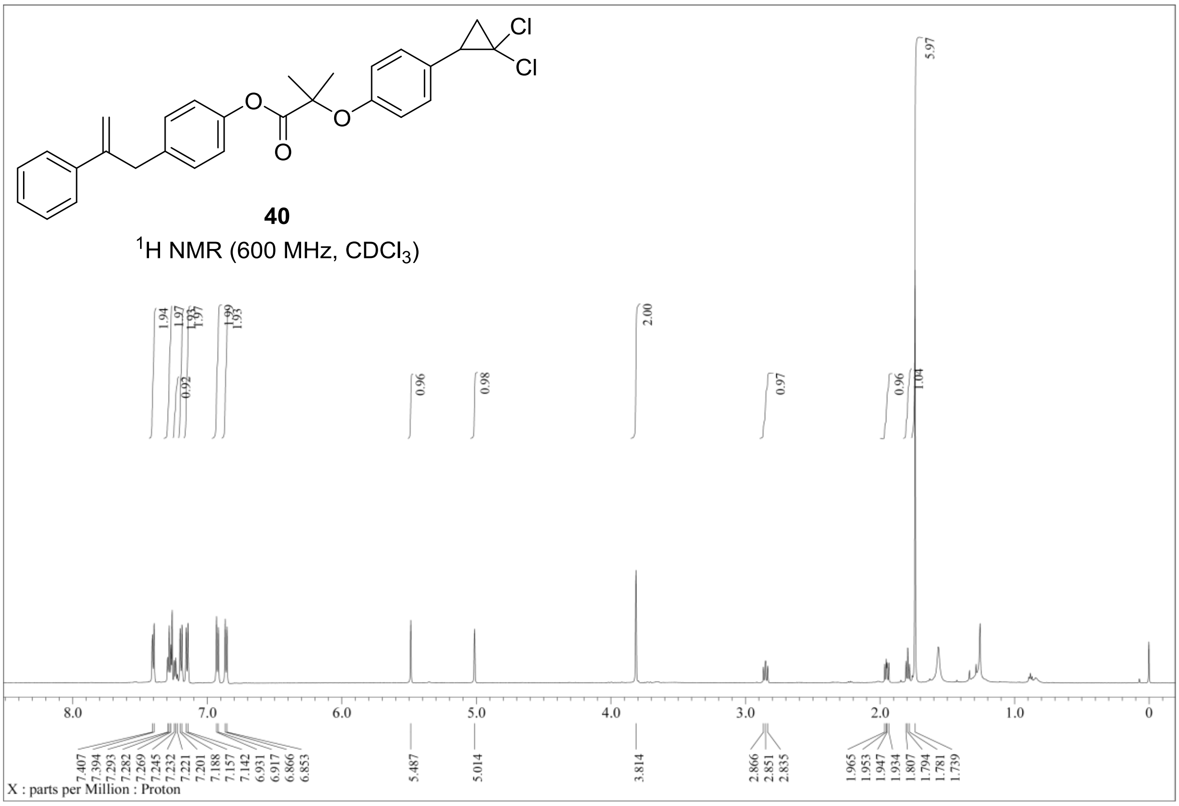
Cl
Cl
O
O
O
40
1H NMR (600 MHz, CDCl3)
5.97
1.94
1.97
1.93
1.97
0.92
1.99
1.93
0.96
0.98
2.00
0.97
0.96
1.04
8.0
7.0
6.0
5.0
4.0
3.0
2.0
1.0
0
7.407
7.394
7.293
7.282
7.269
7.245
7.232
7.221
7.201
7.188
7.157
7.142
6.931
6.917
6.866
6.853
5.487
5.014
3.814
2.866
2.851
2.835
1.965
1.953
1.947
1.934
1.807
1.794
1.781
1.739
X : parts per Million : Proton

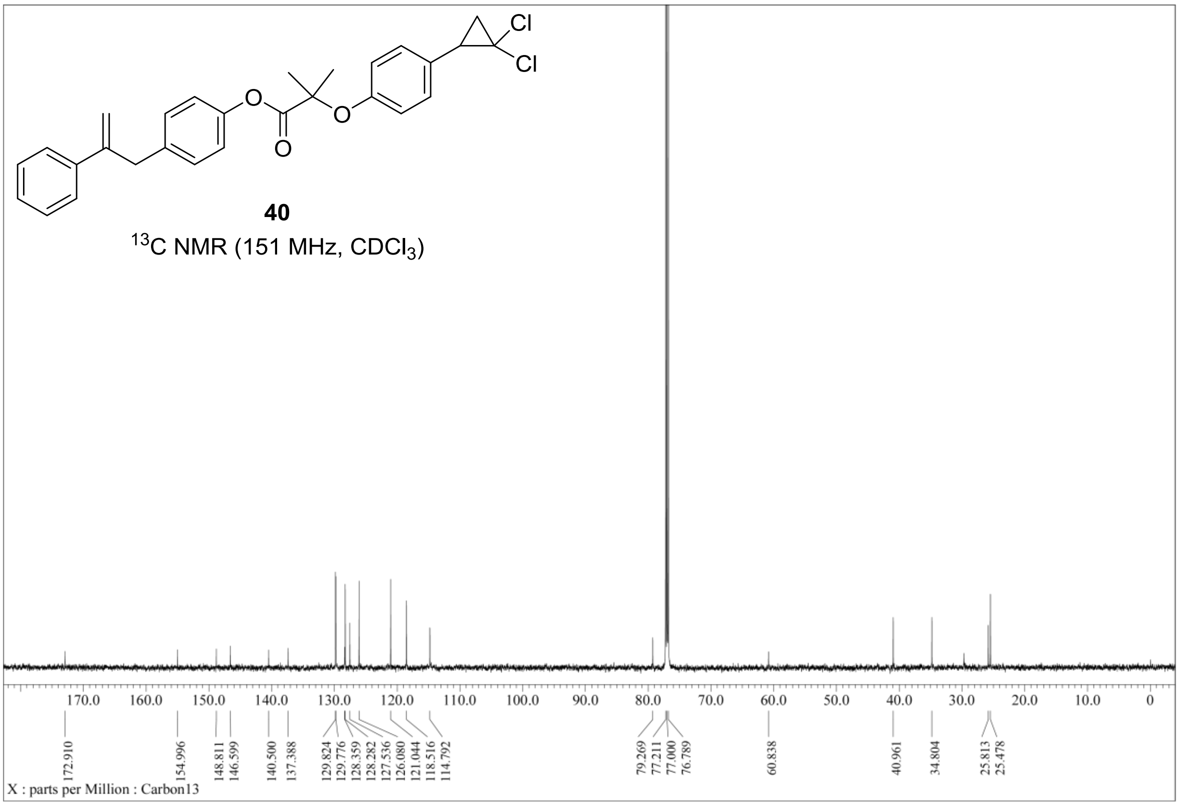
Cl
Cl
O
O
O
40
13C NMR (151 MHz, CDCl3)
170.0
160.0
150.0
140.0
130.0
120.0
110.0
100.0
90.0
80.0
70.0
60.0
50.0
40.0
30.0
20.0
10.0
0
172.910
154.996
148.811
146.599
140.500
137.388
129.824
129.776
128.359
128.282
127.536
126.080
121.044
118.516
114.792
79.269
77.211
77.000
76.789
60.838
40.961
34.804
25.813
25.478
X : parts per Million : Carbon13

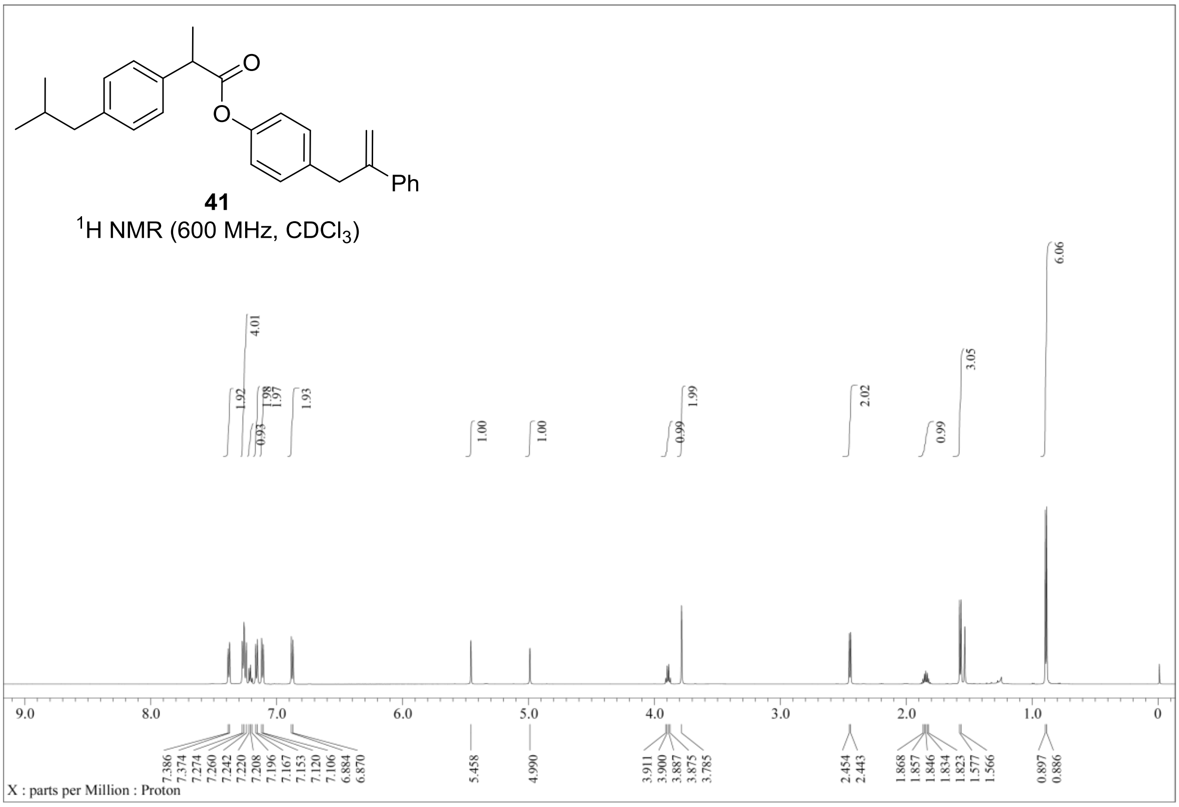
O
O
Ph
41
1H NMR (600 MHz, CDCl3)
1.92 4.01 0.93 1.98 1.97 1.93 1.00 1.00 0.99 1.99 2.02 0.99 3.05 6.06
9.0 8.0 7.0 6.0 5.0 4.0 3.0 2.0 1.0 0
7.386 7.374 7.274 7.260 7.242 7.220 7.208 7.196 7.167 7.153 7.120 7.106 6.884 6.870
5.458
4.990
3.911 3.900 3.887 3.875 3.785
2.454 2.443
1.868 1.857 1.846 1.834 1.823 1.577 1.566
0.897 0.886
X : parts per Million : Proton

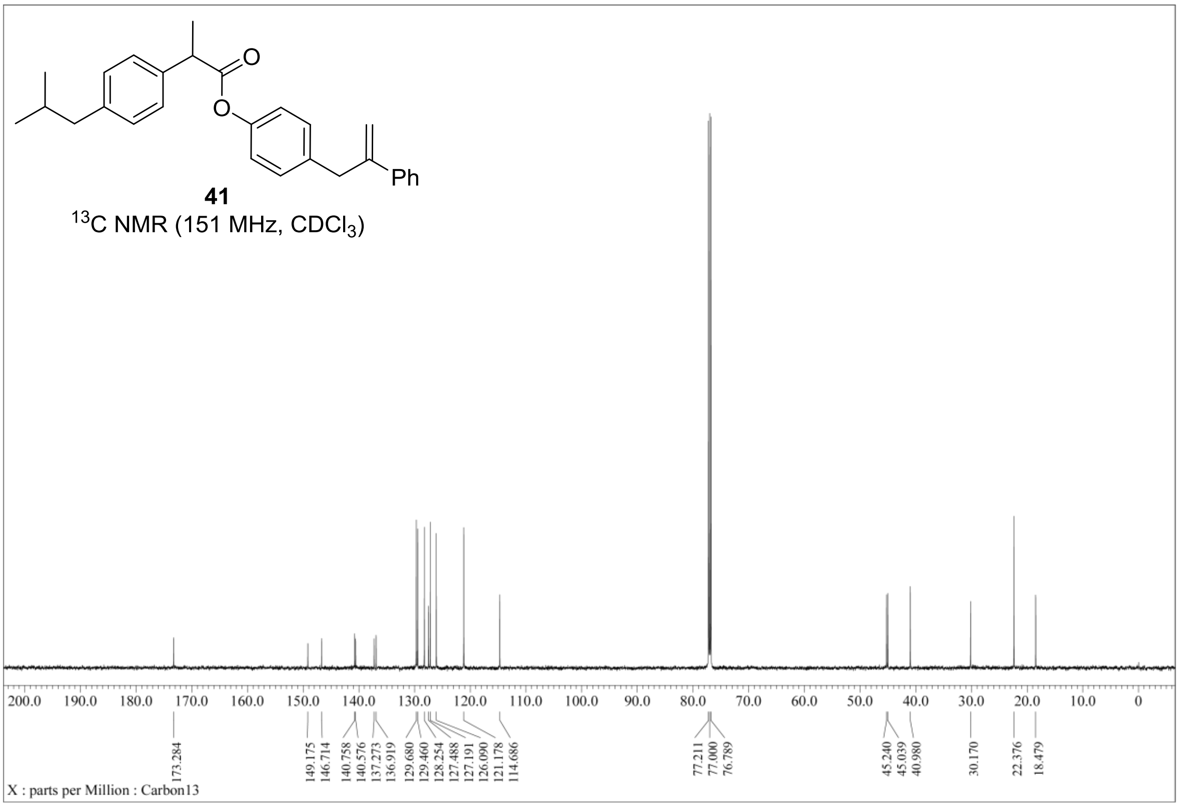
O
O
Ph
41
13C NMR (151 MHz, CDCl3)
200.0 190.0 180.0 170.0 160.0 150.0 140.0 130.0 120.0 110.0 100.0 90.0 80.0 70.0 60.0 50.0 40.0 30.0 20.0 10.0 0
173.284
149.175 146.714 140.758 140.576 137.273 136.919 129.680 129.460 128.254 127.488 127.191 126.090 121.178 114.686
77.211 77.000 76.789
45.240 45.039 40.980
30.170
22.376 18.479
X : parts per Million : Carbon13

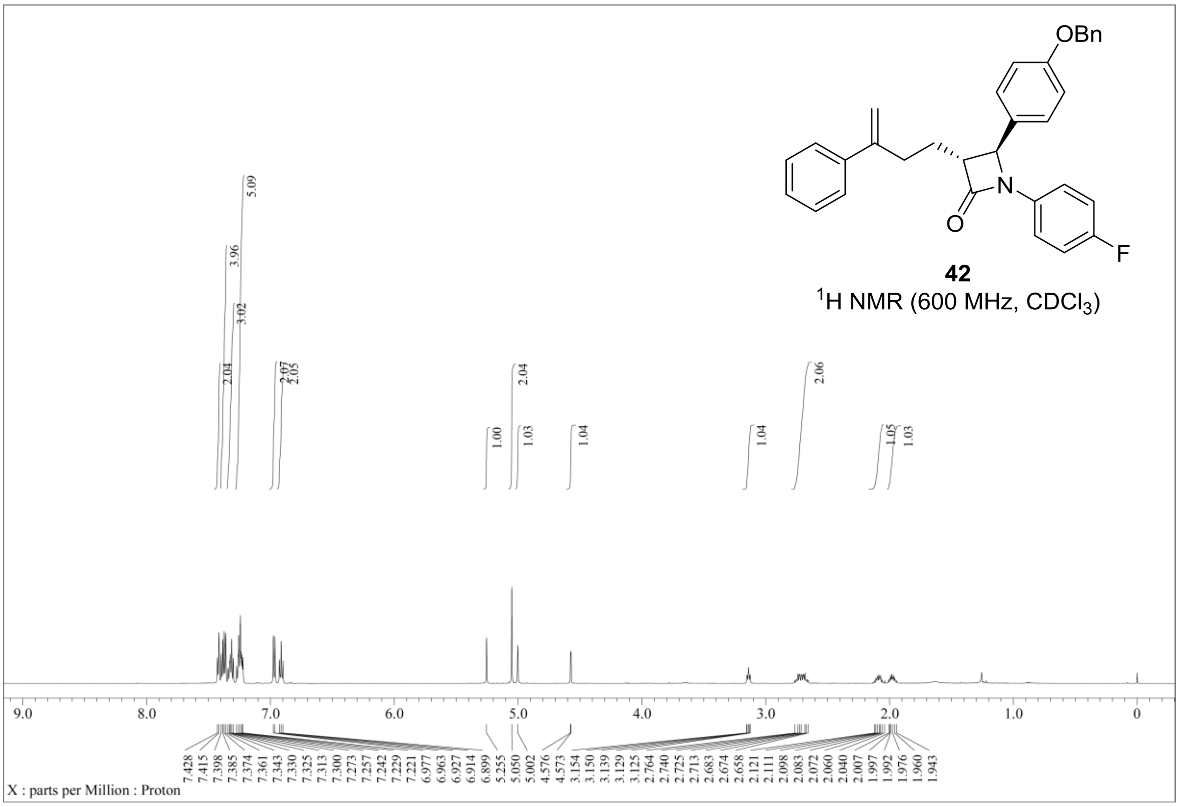
OBn
N
O
F
42
^{1}H NMR (600 MHz, $CDCl_3$)
5.09
3.96
3.02
2.04
2.07
2.05
1.00
2.04
1.03
1.04
1.04
2.06
1.05
1.03
9.0 8.0 7.0 6.0 5.0 4.0 3.0 2.0 1.0 0
7.428 7.415 7.398 7.385 7.374 7.361 7.343 7.330 7.325 7.313 7.300 7.273 7.257 7.242 7.229 7.221 6.977 6.963 6.927 6.914 6.899 5.255 5.050 5.002 4.576 4.573 3.154 3.150 3.139 3.129 3.125 2.764 2.740 2.725 2.713 2.683 2.674 2.658 2.121 2.111 2.098 2.083 2.072 2.060 2.040 2.007 1.997 1.992 1.976 1.960 1.943
X : parts per Million : Proton

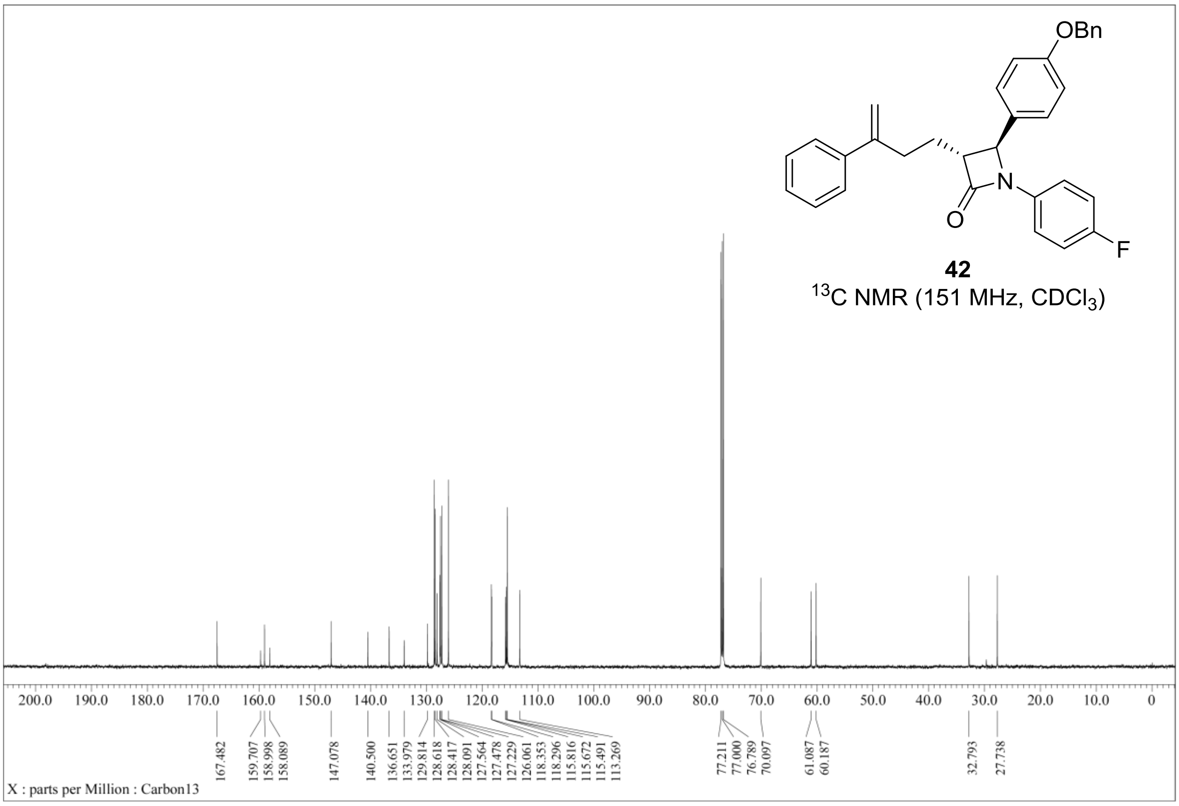
OBn
N
O
F
42
^{13}C NMR (151 MHz, $CDCl_3$)
200.0 190.0 180.0 170.0 160.0 150.0 140.0 130.0 120.0 110.0 100.0 90.0 80.0 70.0 60.0 50.0 40.0 30.0 20.0 10.0 0
167.482 159.707 158.998 158.089 147.078 140.500 136.651 133.979 129.814 128.618 128.417 128.091 127.564 127.478 127.229 126.061 118.353 118.296 115.816 115.672 115.491 113.269 77.211 77.000 76.789 70.097 61.087 60.187 32.793 27.738
X : parts per Million : Carbon13

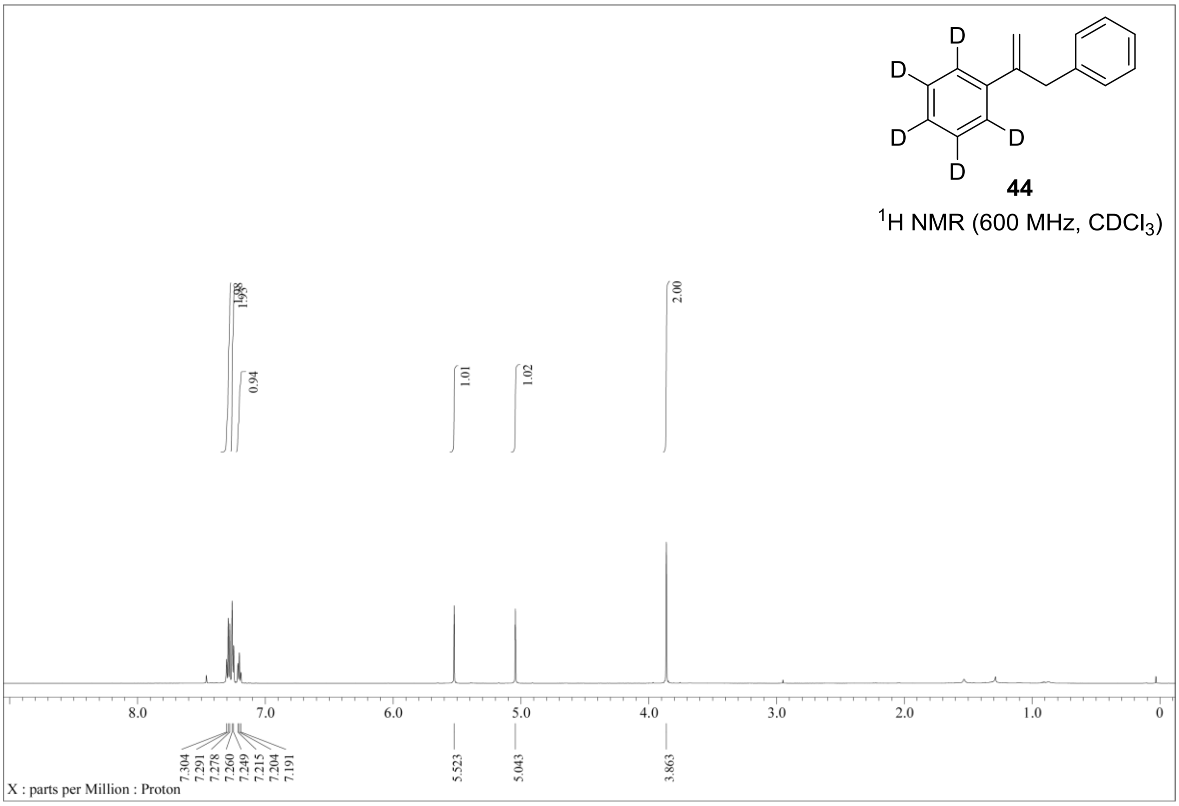
D
44
^{1}H NMR (600 MHz, $CDCl_3$)
0.94
1.01
1.02
2.00
8.0
7.0
6.0
5.0
4.0
3.0
2.0
1.0
0
7.304
7.291
7.278
7.260
7.249
7.215
7.204
7.191
5.523
5.043
3.863
X : parts per Million : Proton

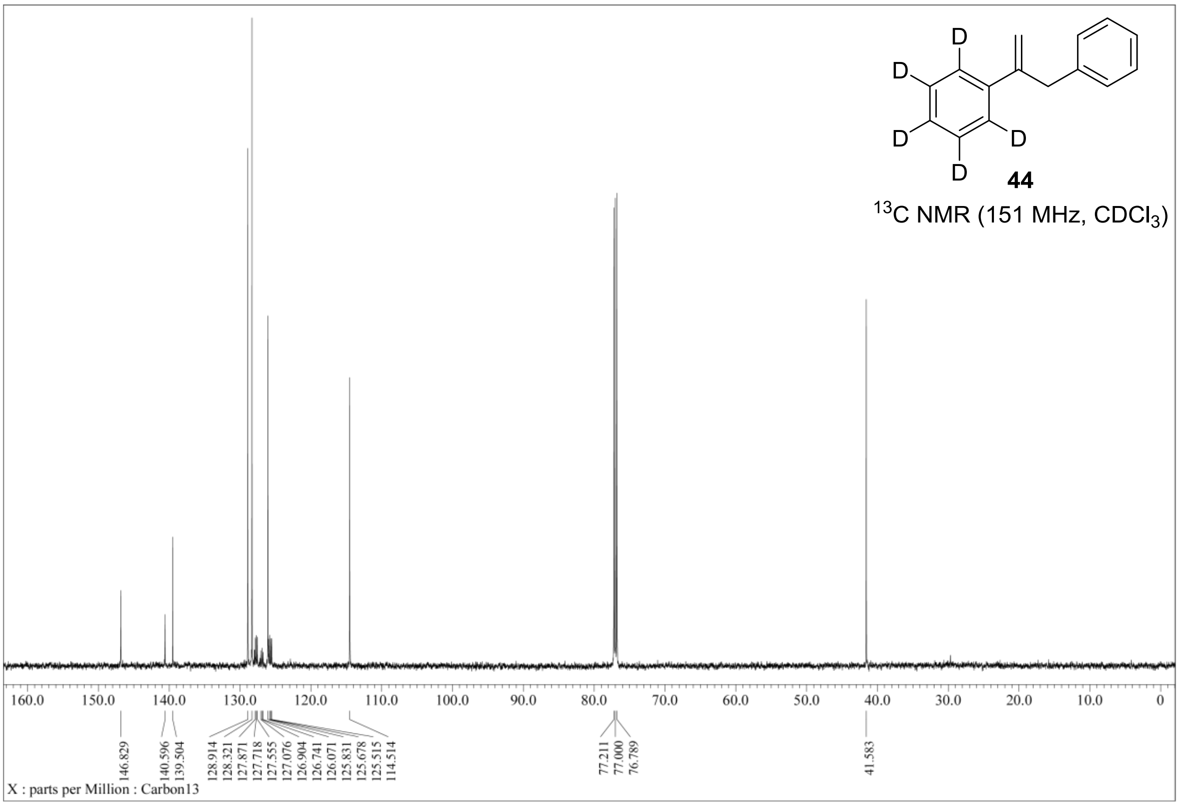
D
44
^{13}C NMR (151 MHz, $CDCl_3$)
160.0
150.0
140.0
130.0
120.0
110.0
100.0
90.0
80.0
70.0
60.0
50.0
40.0
30.0
20.0
10.0
0
146.829
140.596
139.504
128.914
128.321
127.871
127.718
127.555
127.076
126.904
126.741
126.071
125.831
125.678
125.515
114.514
77.211
77.000
76.789
41.583
X : parts per Million : Carbon13

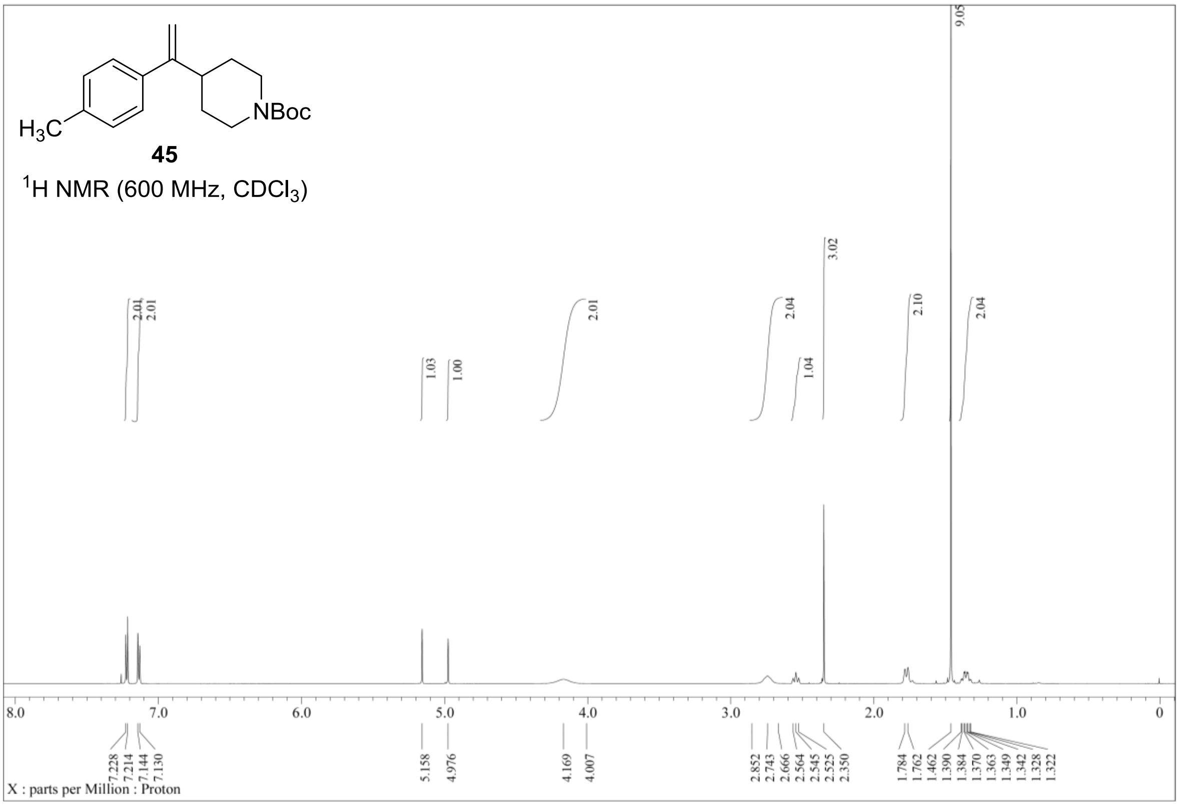

H3C
NBoc
45
1H NMR (600 MHz, CDCl3)
X : parts per Million : Proton

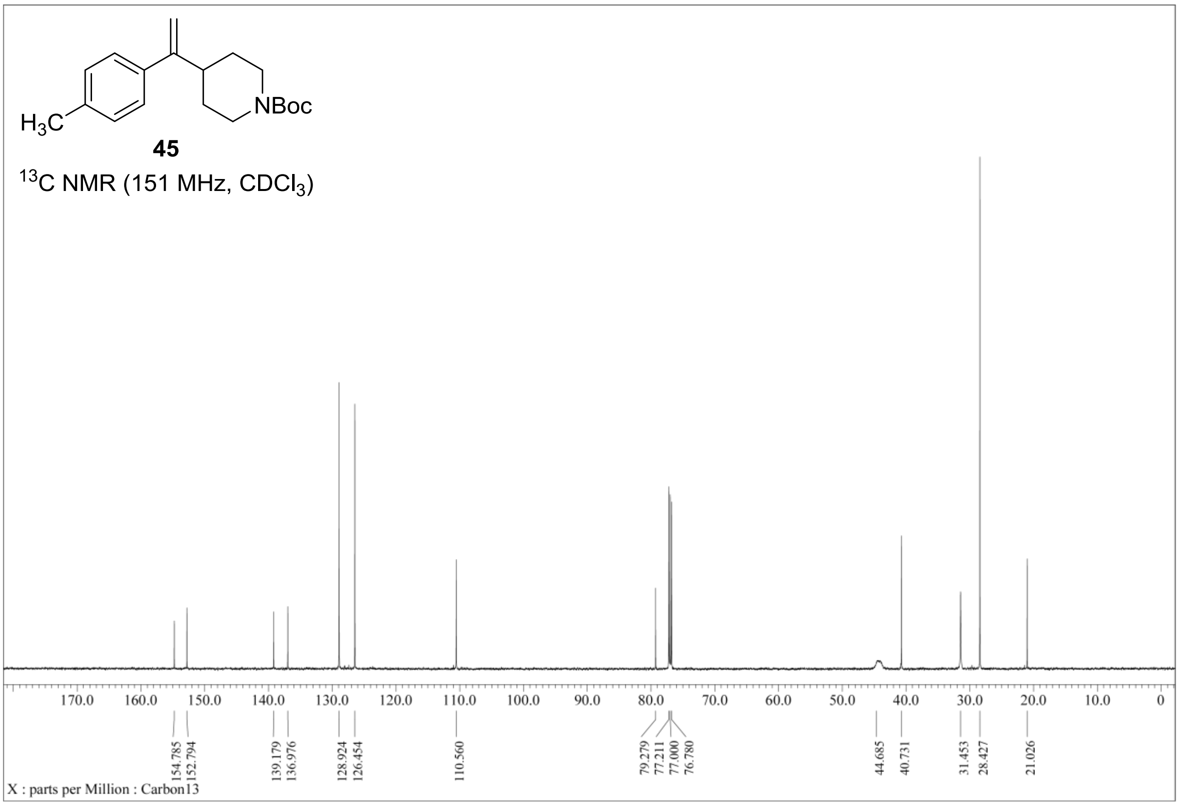

H3C
NBoc
45
13C NMR (151 MHz, CDCl3)
X : parts per Million : Carbon13

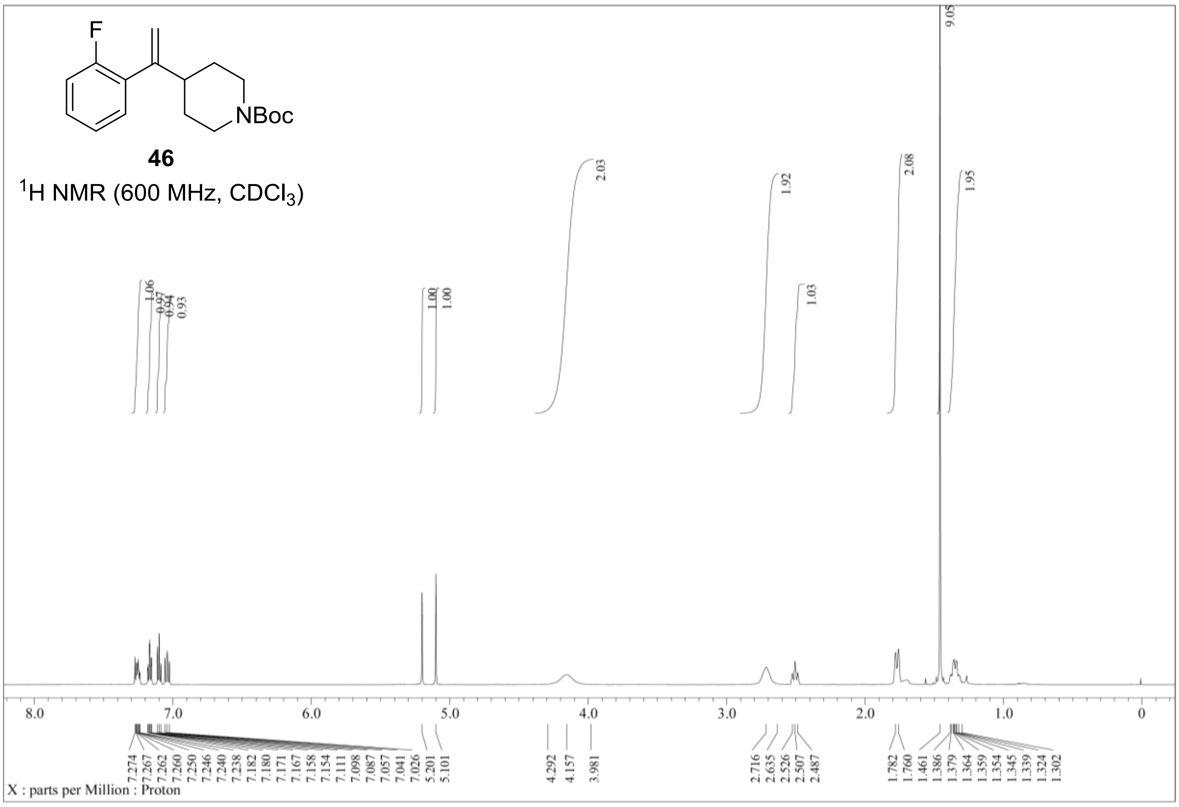
F
NBoc
46
1H NMR (600 MHz, CDCl3)
1.06 0.97 0.94 0.93
1.00 1.00
2.03
1.92
1.03
2.08
9.05
1.95
8.0 7.0 6.0 5.0 4.0 3.0 2.0 1.0 0
7.274 7.267 7.262 7.260 7.250 7.246 7.240 7.238 7.182 7.180 7.171 7.167 7.158 7.154 7.111 7.098 7.087 7.057 7.041 7.026 5.201 5.101
4.292 4.157 3.981
2.716 2.635 2.526 2.507 2.487
1.782 1.760 1.461 1.386 1.379 1.364 1.359 1.354 1.345 1.339 1.324 1.302
X : parts per Million : Proton

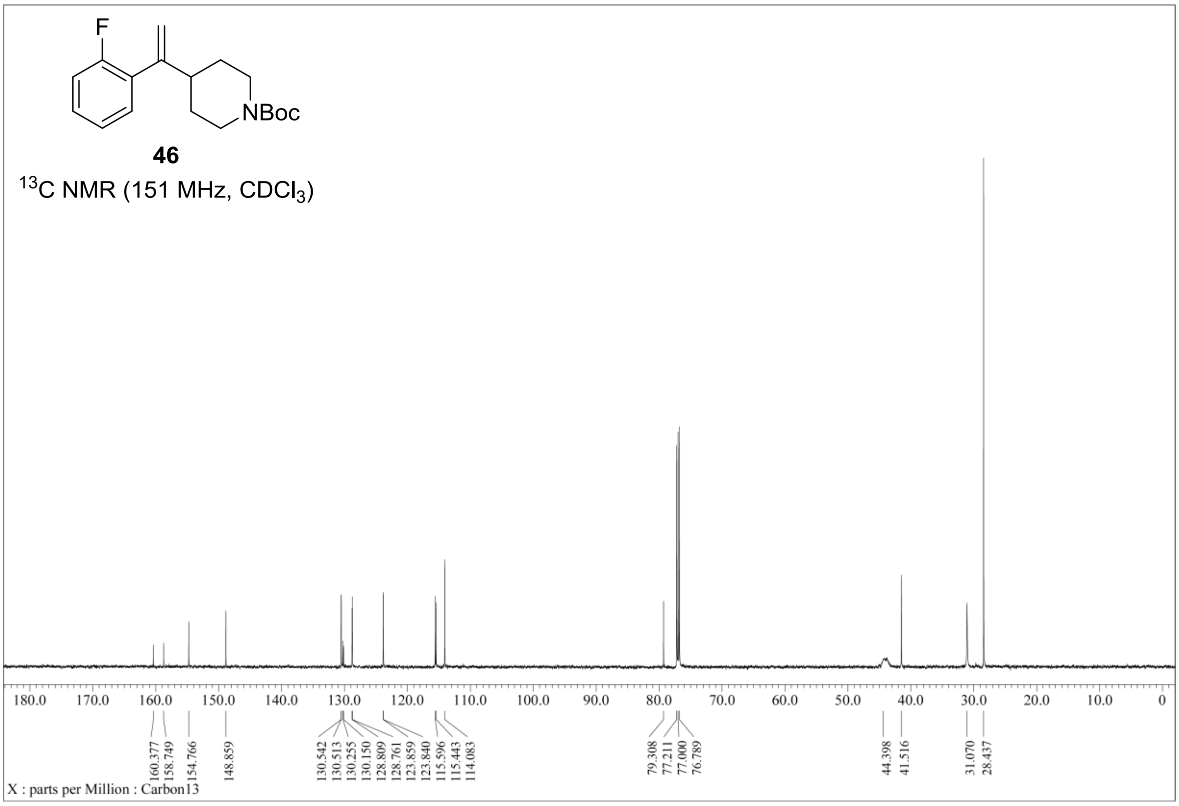
F
NBoc
46
13C NMR (151 MHz, CDCl3)
180.0 170.0 160.0 150.0 140.0 130.0 120.0 110.0 100.0 90.0 80.0 70.0 60.0 50.0 40.0 30.0 20.0 10.0 0
160.377 158.749 154.766 148.859
130.542 130.513 130.255 130.150 128.809 128.761 123.859 123.840 115.596 115.443 114.083
79.308 77.211 77.000 76.789
44.398 41.516
31.070 28.437
X : parts per Million : Carbon13

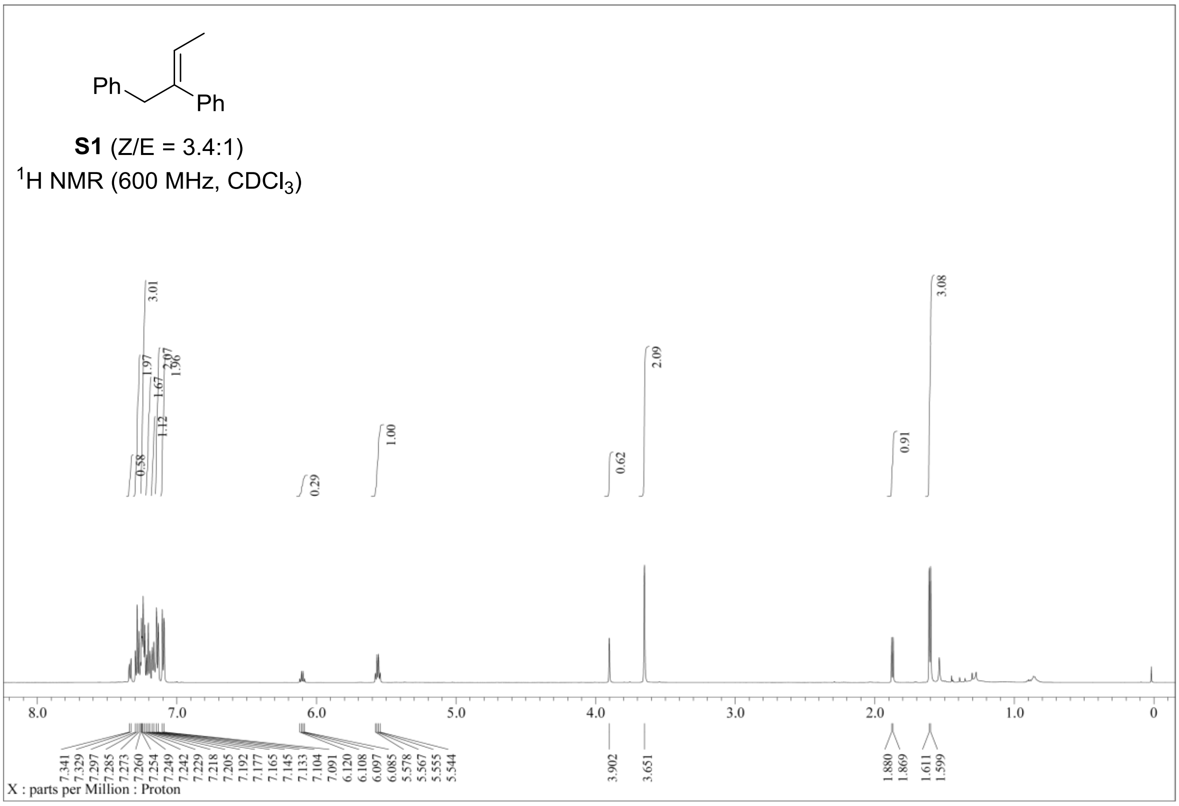
Ph
Ph
S1 (Z/E = 3.4:1)
1H NMR (600 MHz, CDCl3)
3.01
1.97
1.67
2.07
1.96
1.12
0.58
0.29
1.00
0.62
2.09
0.91
3.08
8.0 7.0 6.0 5.0 4.0 3.0 2.0 1.0 0
7.341 7.329 7.297 7.285 7.273 7.260 7.254 7.249 7.242 7.229 7.218 7.205 7.192 7.177 7.165 7.145 7.133 7.104 7.091 6.120 6.108 6.097 6.085 5.578 5.567 5.555 5.544
3.902
3.651
1.880 1.869
1.611 1.599
X : parts per Million : Proton

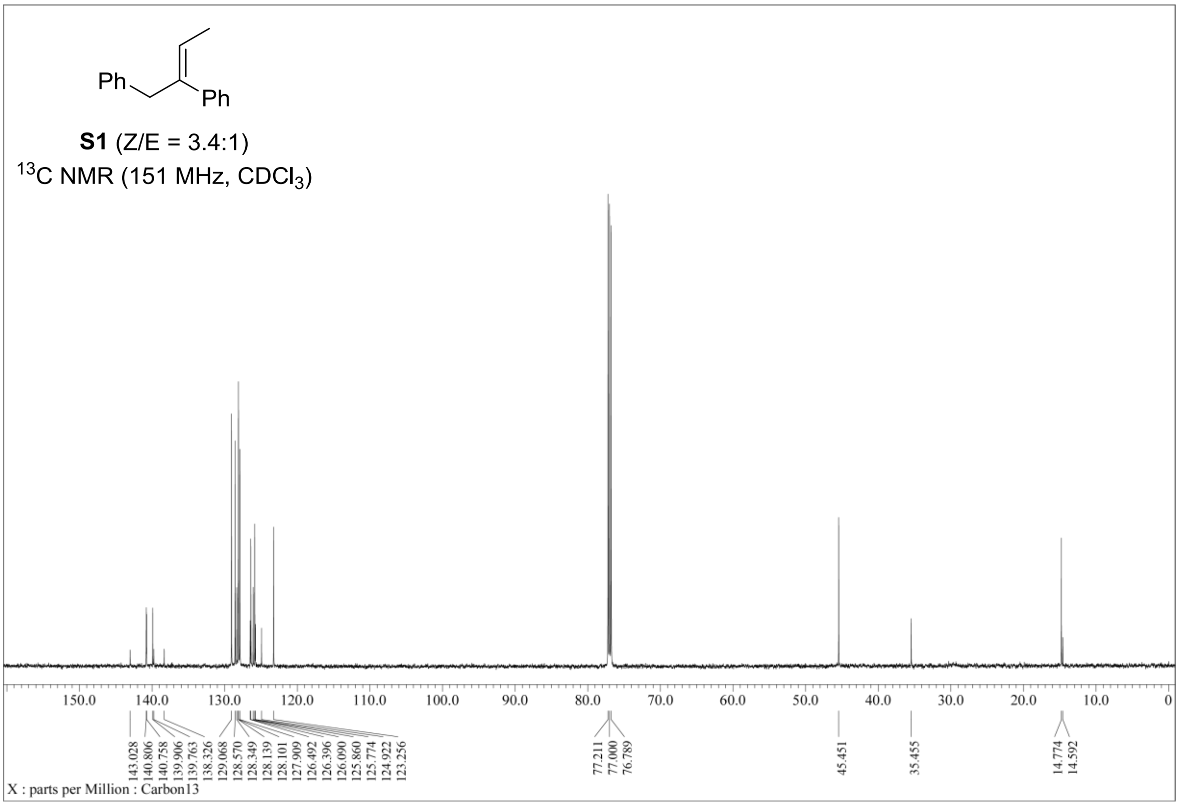
Ph
Ph
S1 (Z/E = 3.4:1)
13C NMR (151 MHz, CDCl3)
150.0 140.0 130.0 120.0 110.0 100.0 90.0 80.0 70.0 60.0 50.0 40.0 30.0 20.0 10.0 0
143.028 140.806 140.758 139.906 139.763 138.326 129.068 128.570 128.349 128.139 128.101 127.909 126.492 126.396 126.090 125.860 125.774 124.922 123.256
77.211 77.000 76.789
45.451
35.455
14.774 14.592
X : parts per Million : Carbon13

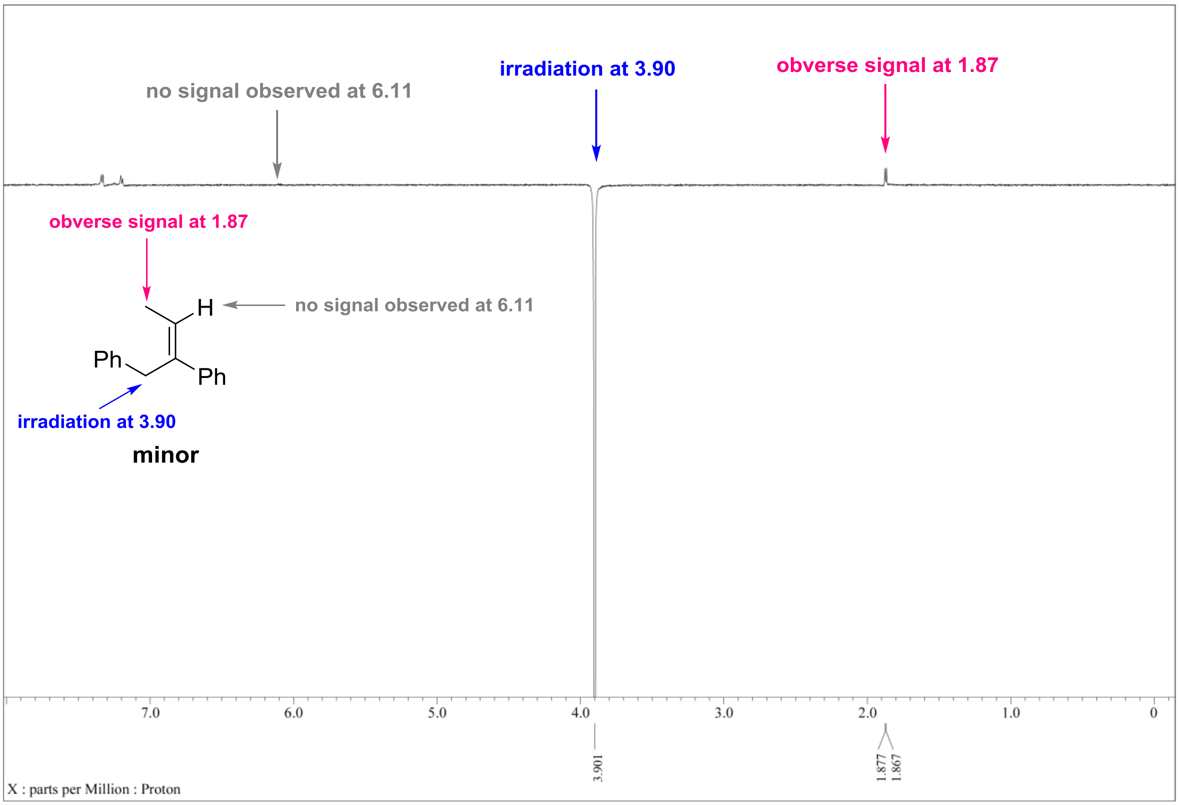
no signal observed at 6.11
irradiation at 3.90
obverse signal at 1.87
obverse signal at 1.87
H
no signal observed at 6.11
Ph
Ph
irradiation at 3.90
minor
7.0
6.0
5.0
4.0
3.0
2.0
1.0
0
3.901
1.877
1.867
X : parts per Million : Proton

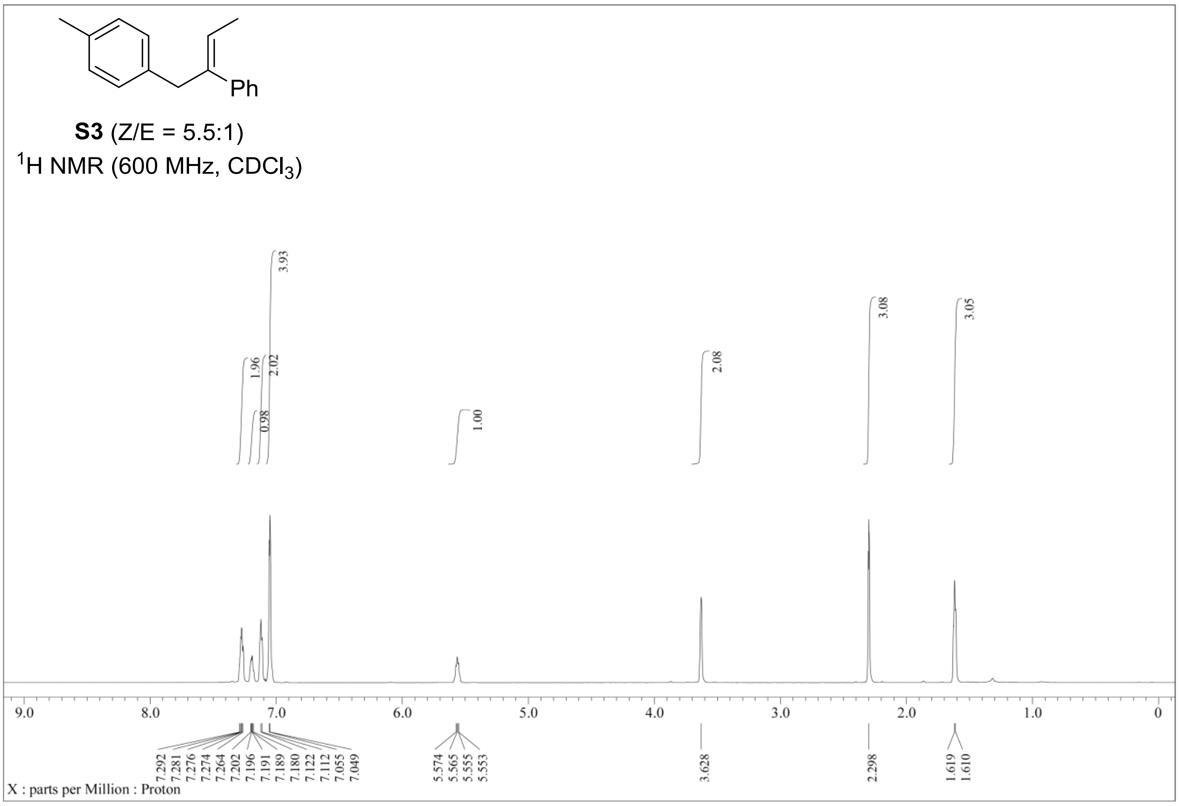
Ph
S3 (Z/E = 5.5:1)
^{1}H NMR (600 MHz, $CDCl_3$)
3.93
1.96
2.02
0.98
1.00
2.08
3.08
3.05
9.0
8.0
7.0
6.0
5.0
4.0
3.0
2.0
1.0
0
7.292
7.281
7.276
7.274
7.264
7.202
7.196
7.191
7.189
7.180
7.122
7.112
7.055
7.049
5.574
5.565
5.555
5.553
3.628
2.298
1.619
1.610
X : parts per Million : Proton

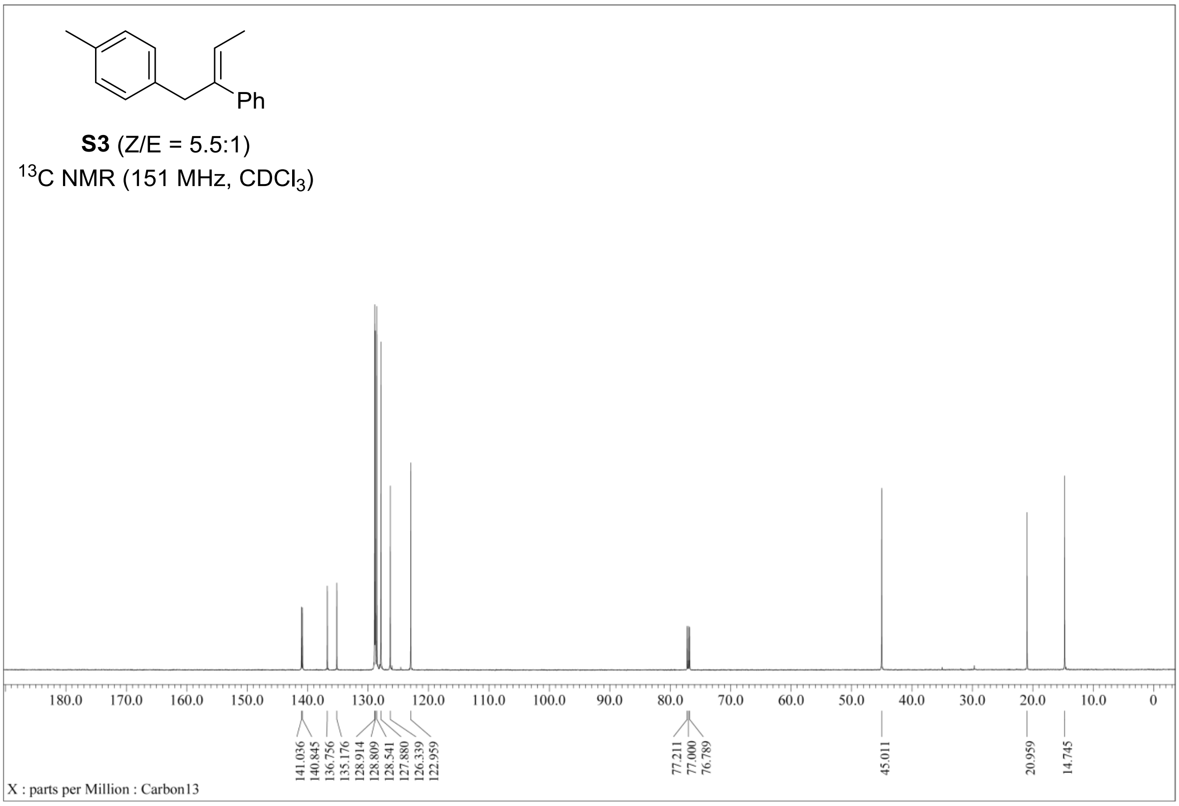
Ph
S3 (Z/E = 5.5:1)
13C NMR (151 MHz, CDCl3)
180.0 170.0 160.0 150.0 140.0 130.0 120.0 110.0 100.0 90.0 80.0 70.0 60.0 50.0 40.0 30.0 20.0 10.0 0
141.036 140.845 136.756 135.176 128.914 128.809 128.541 127.880 126.339 122.959
77.211 77.000 76.789
45.011
20.959
14.745
X : parts per Million : Carbon13

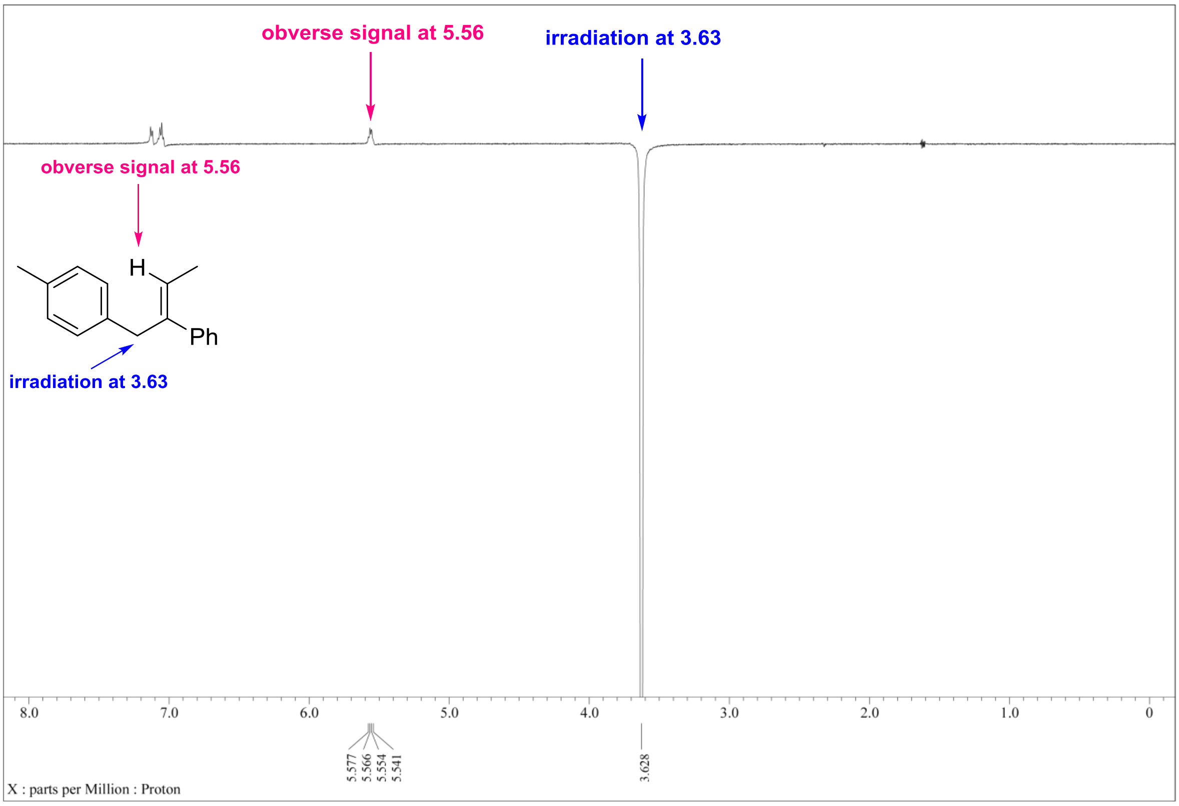
obverse signal at 5.56
irradiation at 3.63
obverse signal at 5.56
H
Ph
irradiation at 3.63
8.0 7.0 6.0 5.0 4.0 3.0 2.0 1.0 0
5.577 5.566 5.554 5.541
3.628
X : parts per Million : Proton

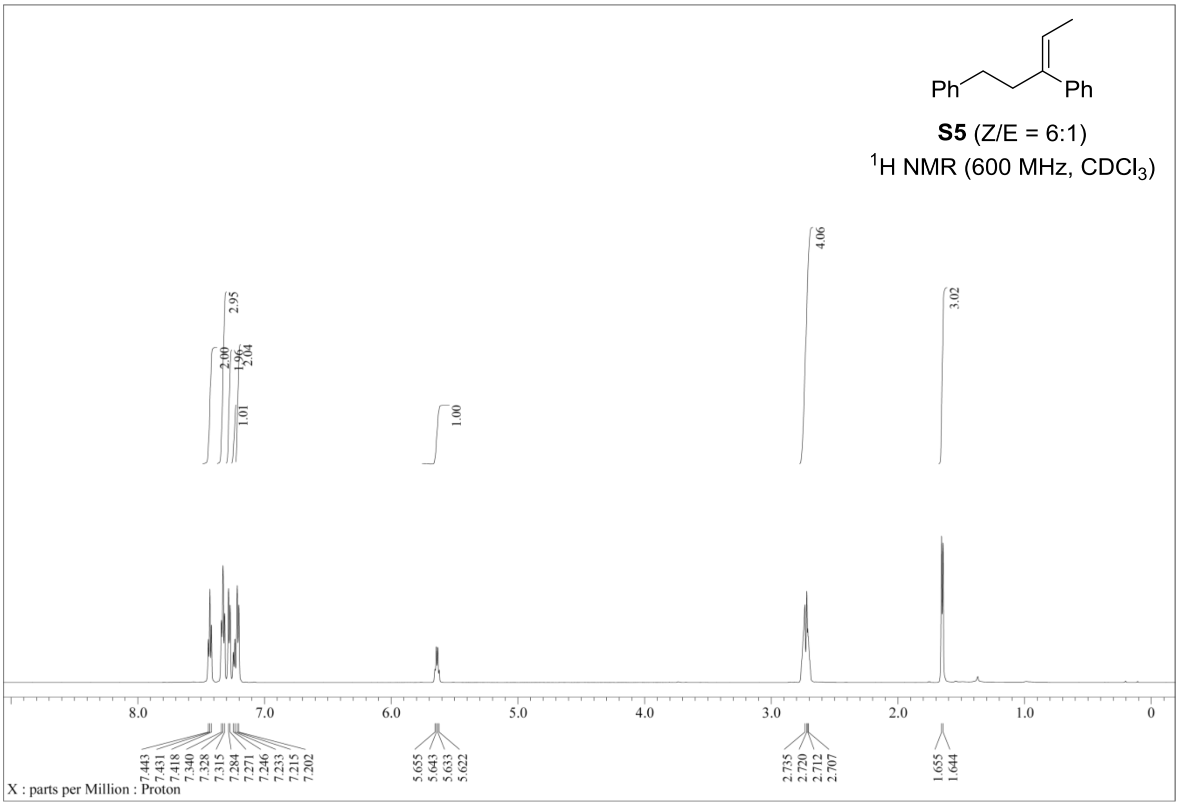
Ph
Ph
S5 (Z/E = 6:1)
1H NMR (600 MHz, CDCl3)
X : parts per Million : Proton

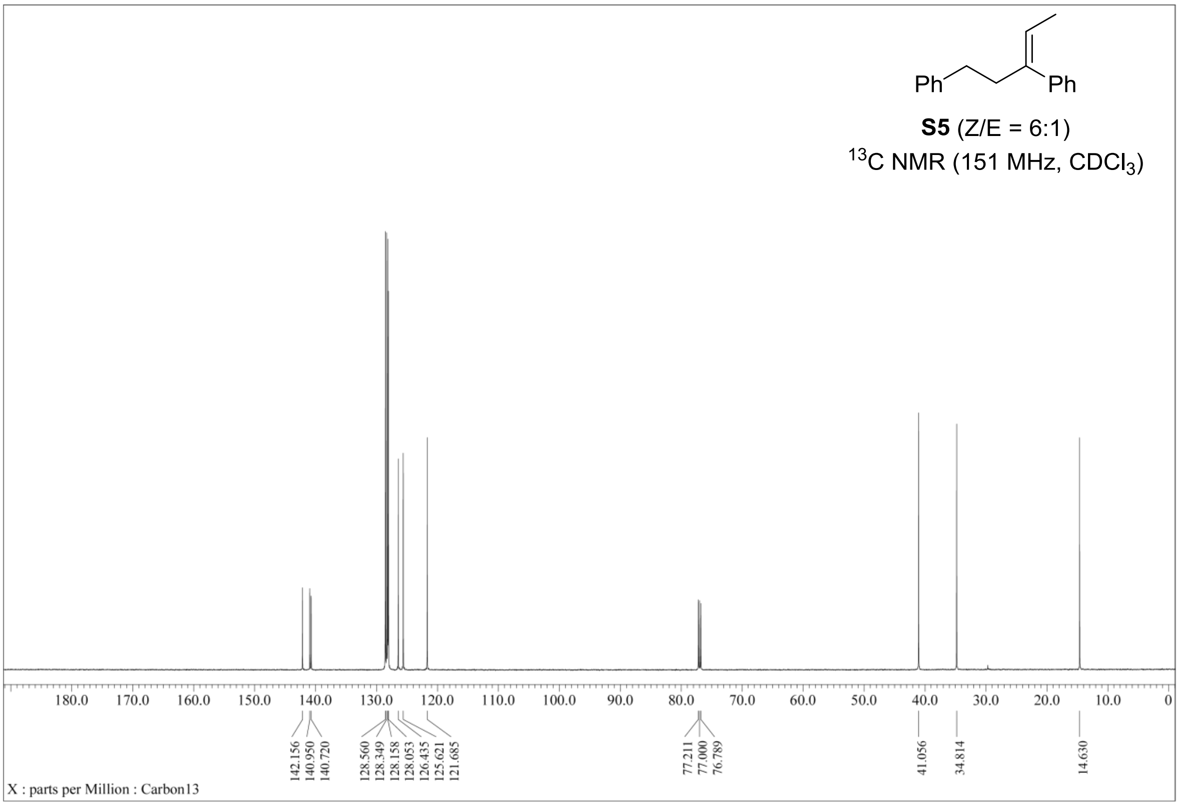
Ph
Ph
S5 (Z/E = 6:1)
13C NMR (151 MHz, CDCl3)
X : parts per Million : Carbon13

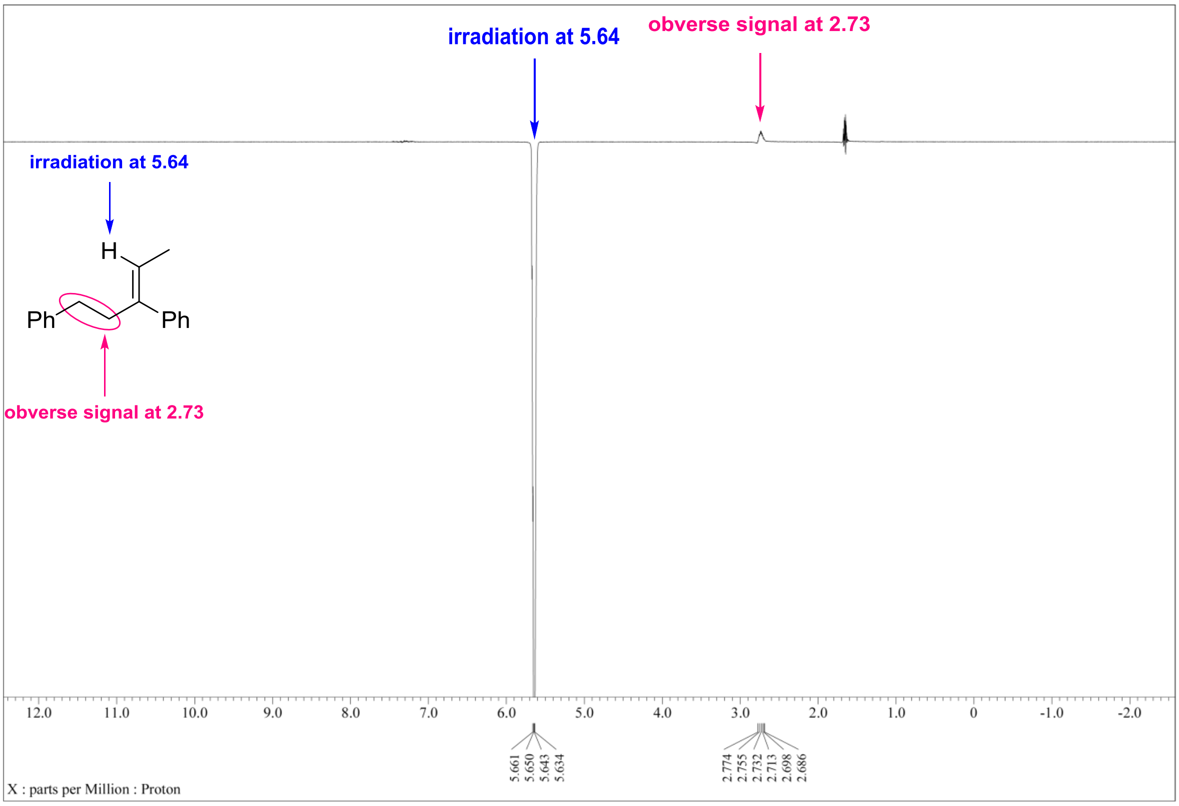
irradiation at 5.64
obverse signal at 2.73
irradiation at 5.64
H
Ph
Ph
obverse signal at 2.73
12.0 11.0 10.0 9.0 8.0 7.0 6.0 5.0 4.0 3.0 2.0 1.0 0 -1.0 -2.0
5.661 5.650 5.643 5.634
2.774 2.755 2.732 2.713 2.698 2.686
X : parts per Million : Proton

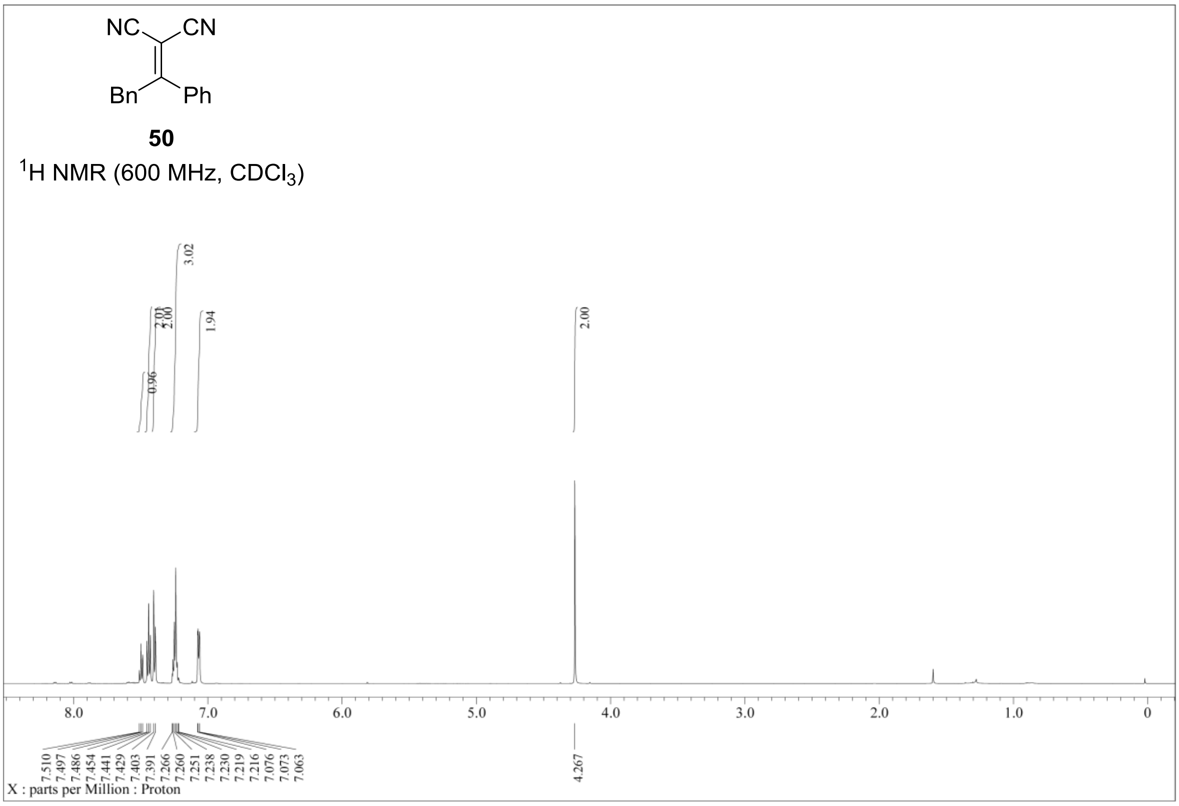
NC CN
Bn Ph
50
^{1}H NMR (600 MHz, CDCl$_3$)
0.96
2.01
2.00
3.02
1.94
2.00
8.0
7.0
6.0
5.0
4.0
3.0
2.0
1.0
0
7.510
7.497
7.486
7.454
7.441
7.429
7.403
7.391
7.266
7.260
7.251
7.238
7.230
7.219
7.216
7.076
7.073
7.063
4.267
X : parts per Million : Proton

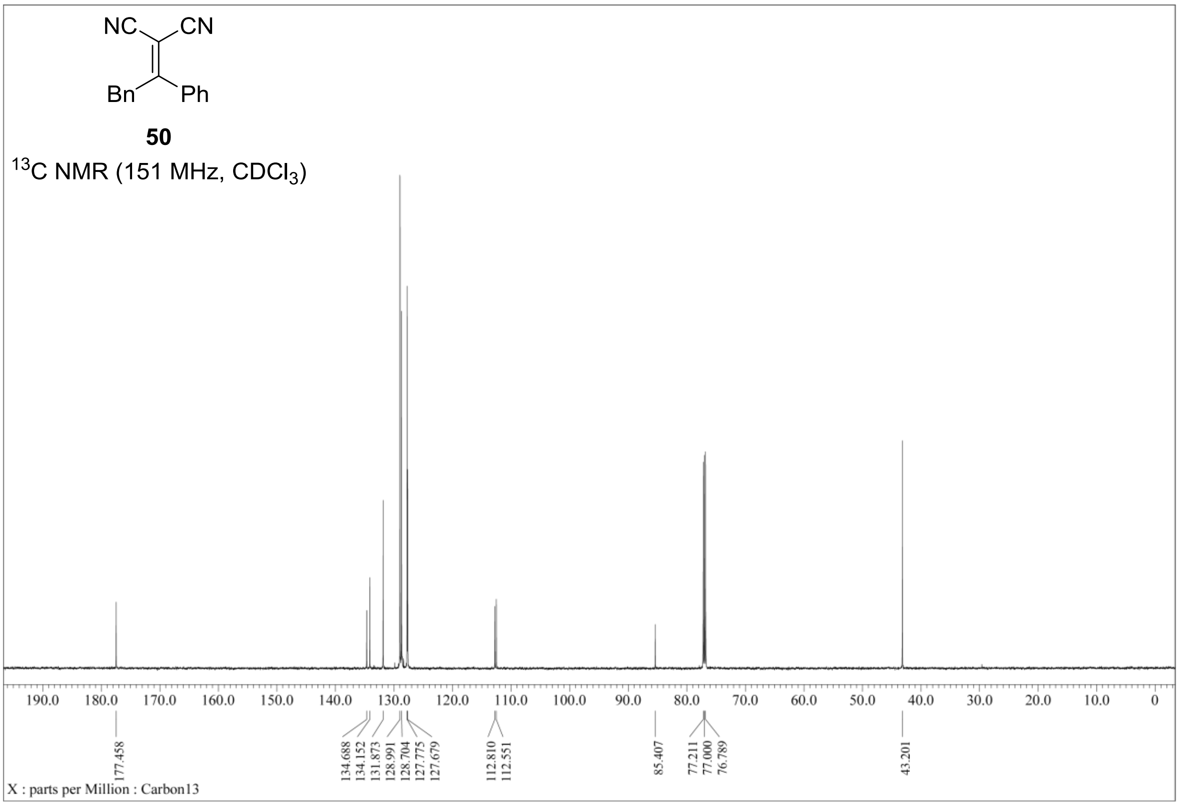
NC CN
Bn Ph
50
^{13}C NMR (151 MHz, CDCl$_3$)
190.0
180.0
170.0
160.0
150.0
140.0
130.0
120.0
110.0
100.0
90.0
80.0
70.0
60.0
50.0
40.0
30.0
20.0
10.0
0
177.458
134.688
134.152
131.873
128.991
128.704
127.775
127.679
112.810
112.551
85.407
77.211
77.000
76.789
43.201
X : parts per Million : Carbon13

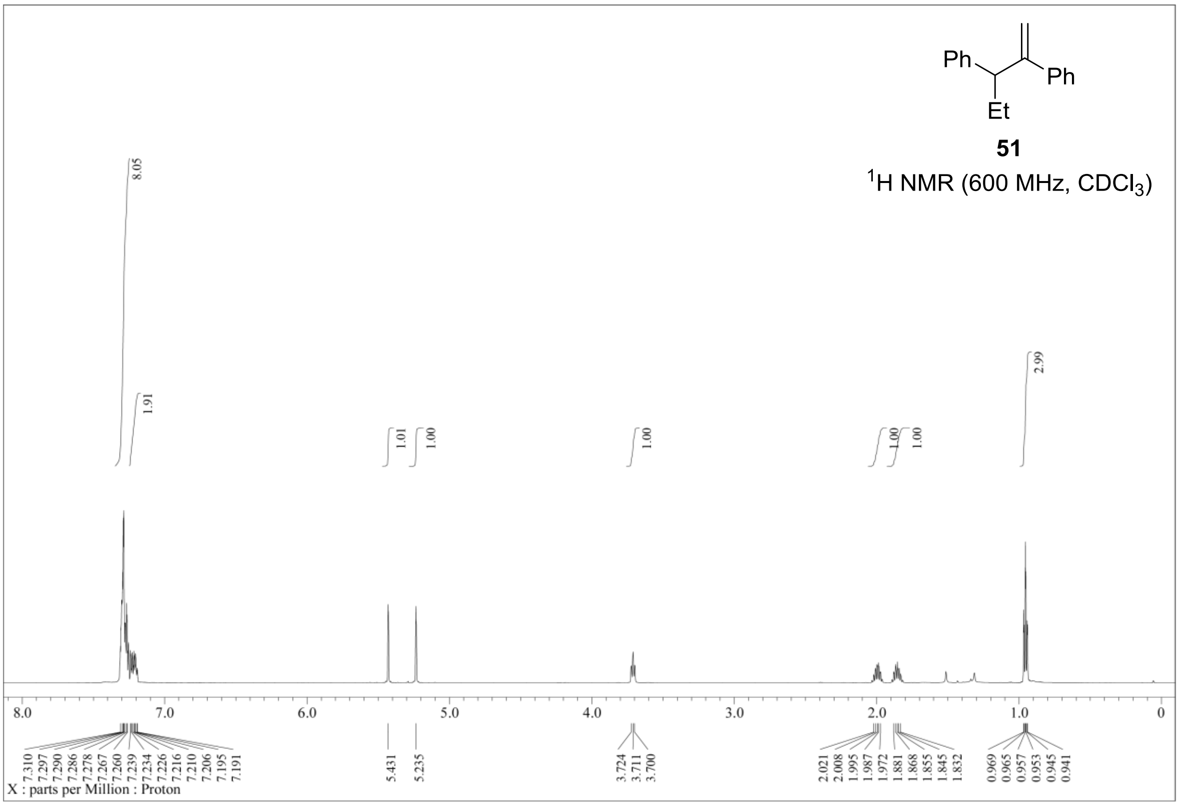
51
^{1}H NMR (600 MHz, CDCl$_3$)
X : parts per Million : Proton

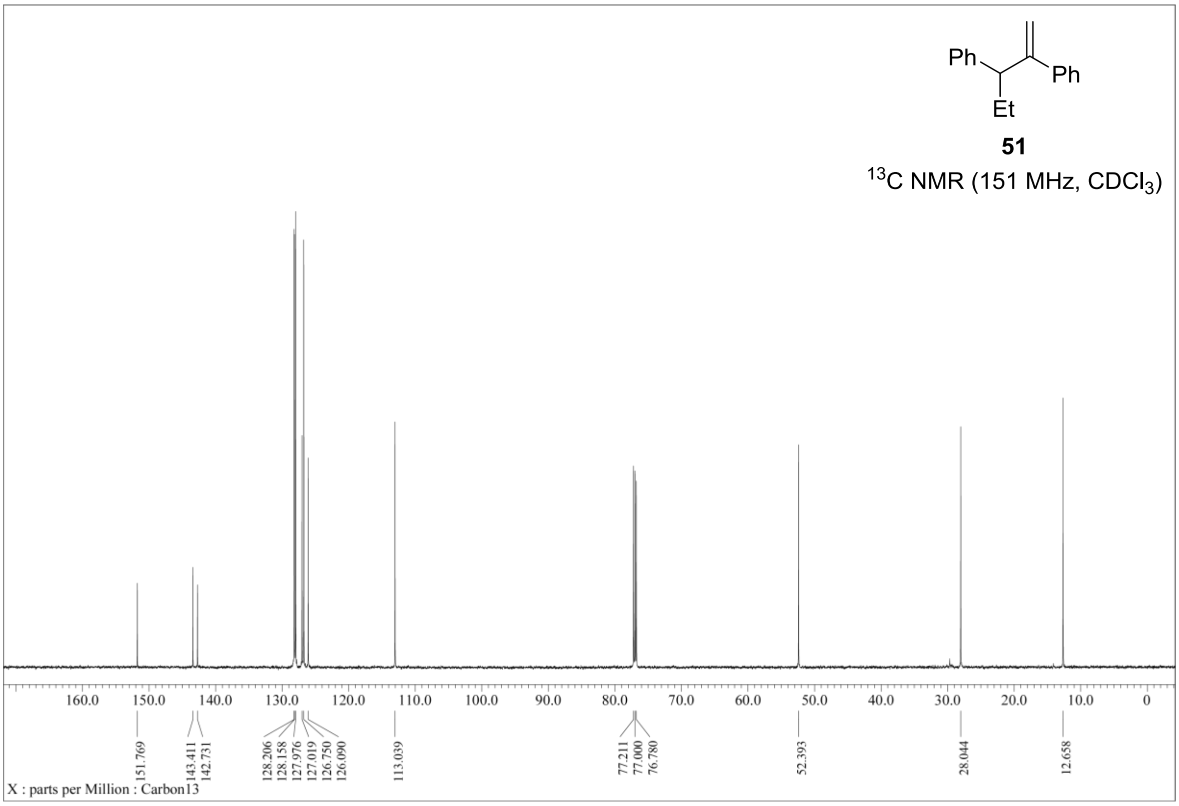
51
^{13}C NMR (151 MHz, CDCl$_3$)
X : parts per Million : Carbon13

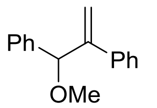

52

^{1}H NMR (600 MHz, $CDCl_3$)

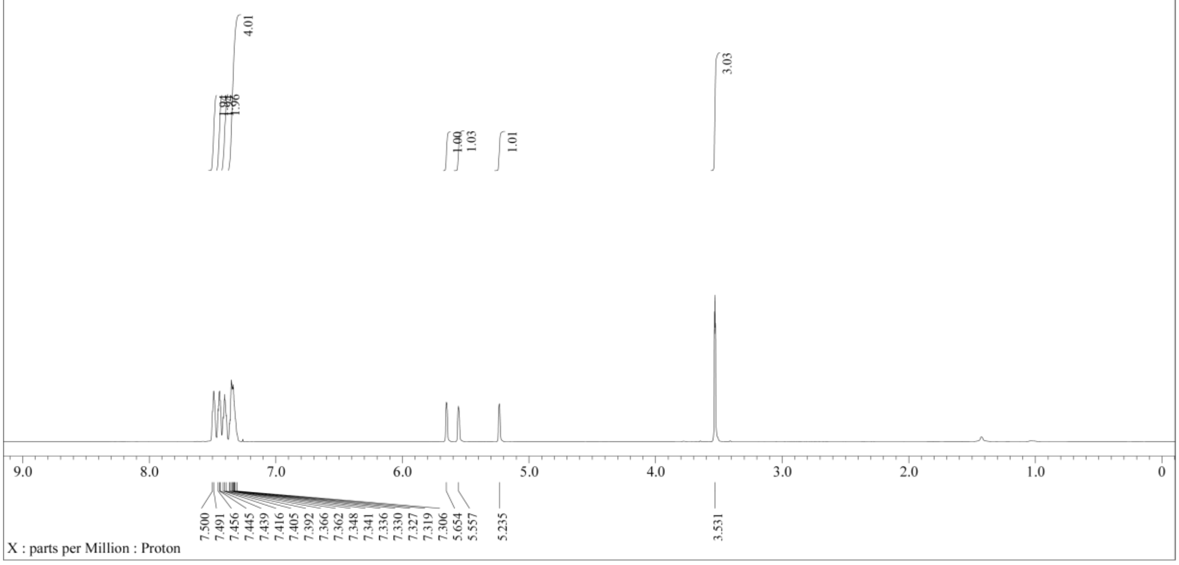

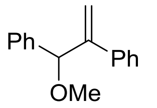

52

^{13}C NMR (151 MHz, $CDCl_3$)

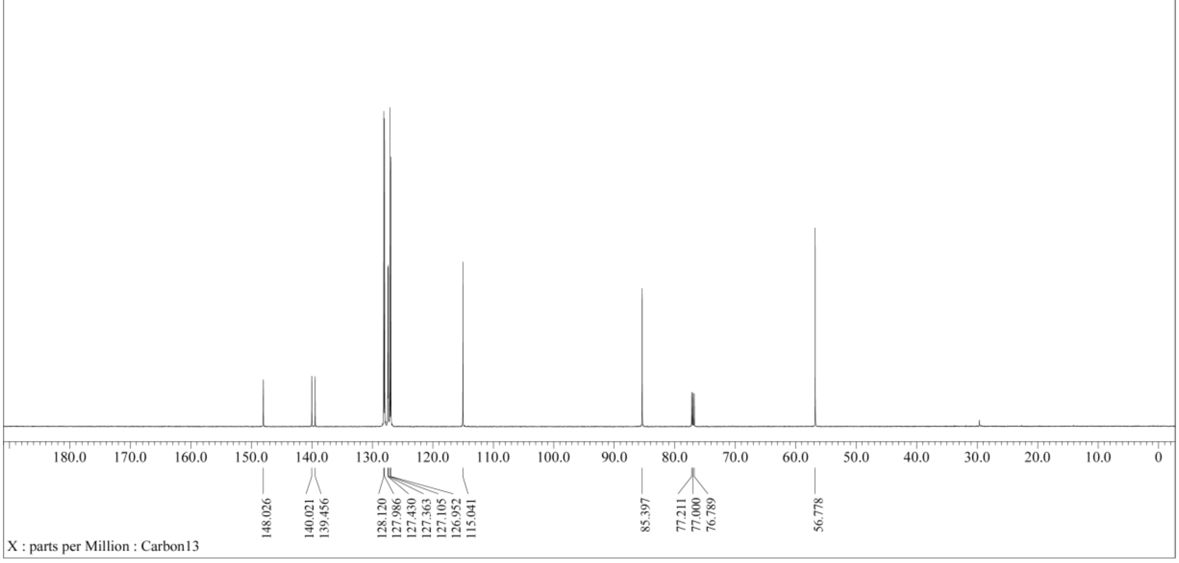

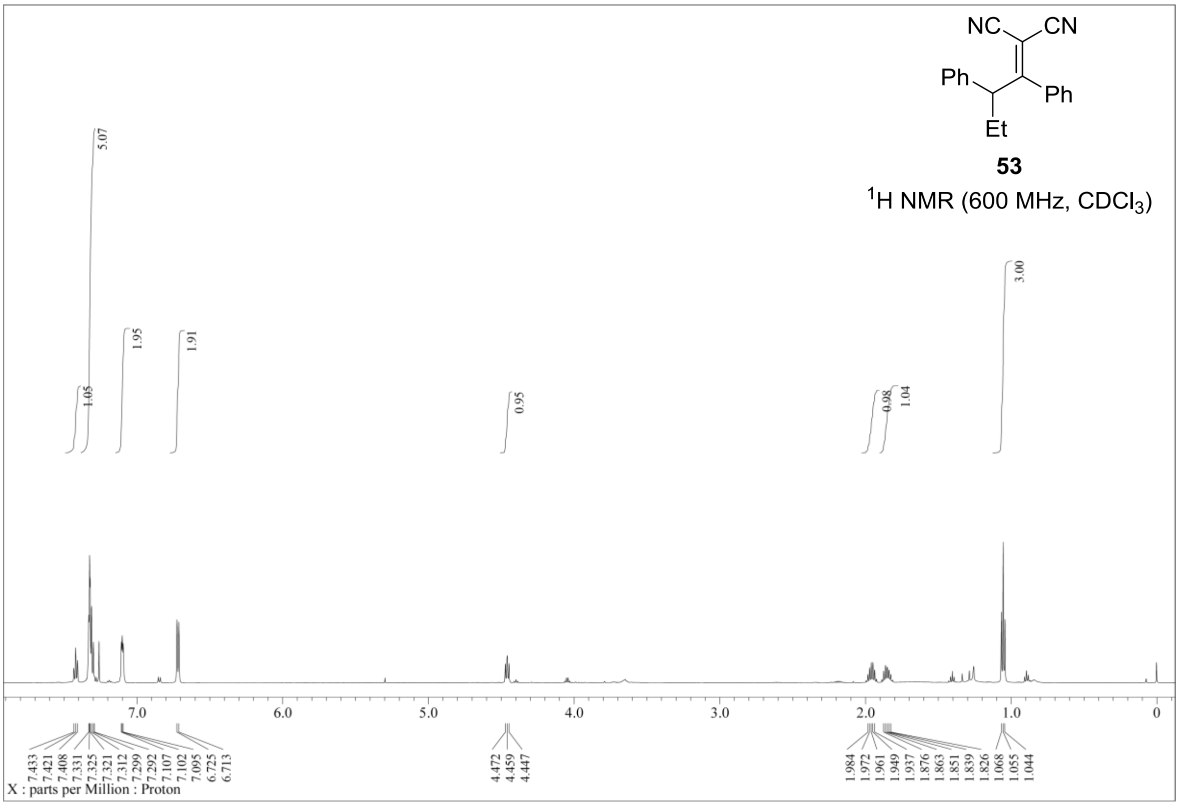
NC CN
Ph Ph
Et
53
1H NMR (600 MHz, CDCl3)
5.07
1.05
1.95
1.91
0.95
0.98
1.04
3.00
7.0
6.0
5.0
4.0
3.0
2.0
1.0
0
7.433 7.421 7.408 7.331 7.325 7.321 7.312 7.299 7.292 7.107 7.102 7.095 6.725 6.713
4.472 4.459 4.447
1.984 1.972 1.961 1.949 1.937 1.876 1.863 1.851 1.839 1.826 1.068 1.055 1.044
X : parts per Million : Proton

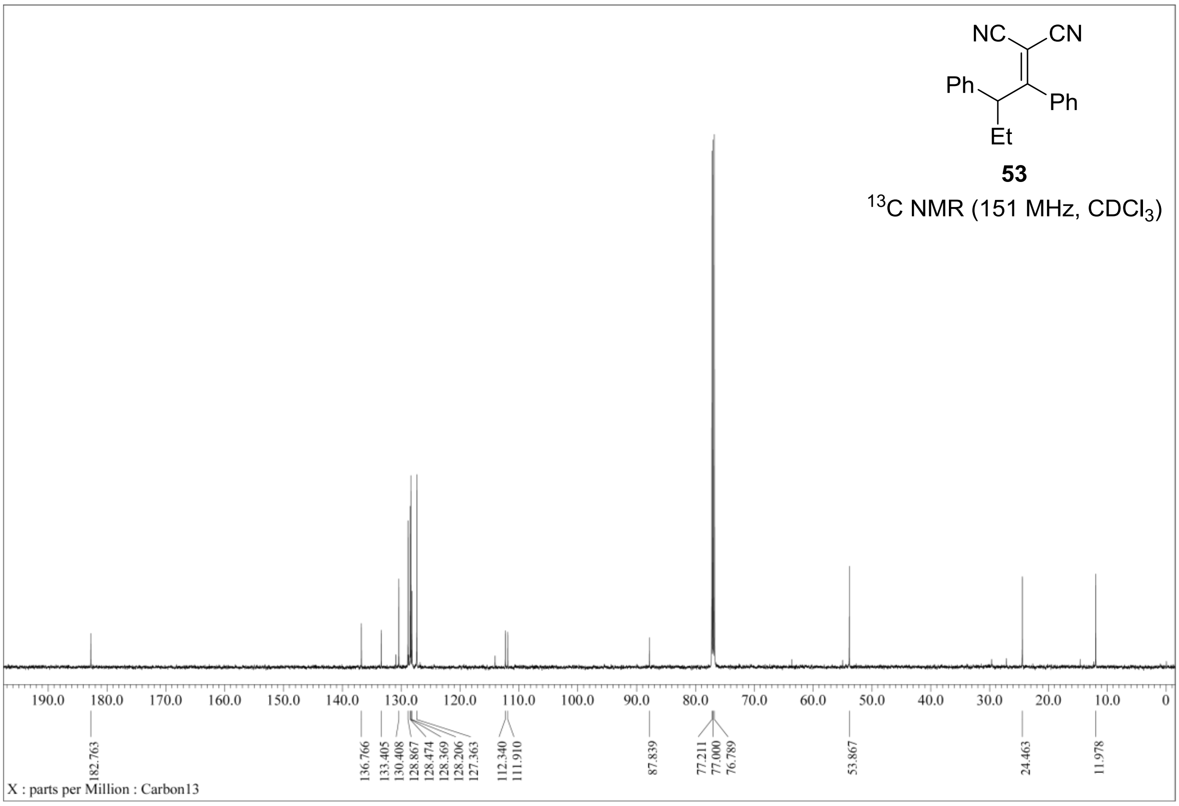
NC CN
Ph Ph
Et
53
13C NMR (151 MHz, CDCl3)
190.0
180.0
170.0
160.0
150.0
140.0
130.0
120.0
110.0
100.0
90.0
80.0
70.0
60.0
50.0
40.0
30.0
20.0
10.0
0
182.763
136.766 133.405 130.408 128.867 128.474 128.369 128.206 127.363
112.340 111.910
87.839
77.211 77.000 76.789
53.867
24.463
11.978
X : parts per Million : Carbon13

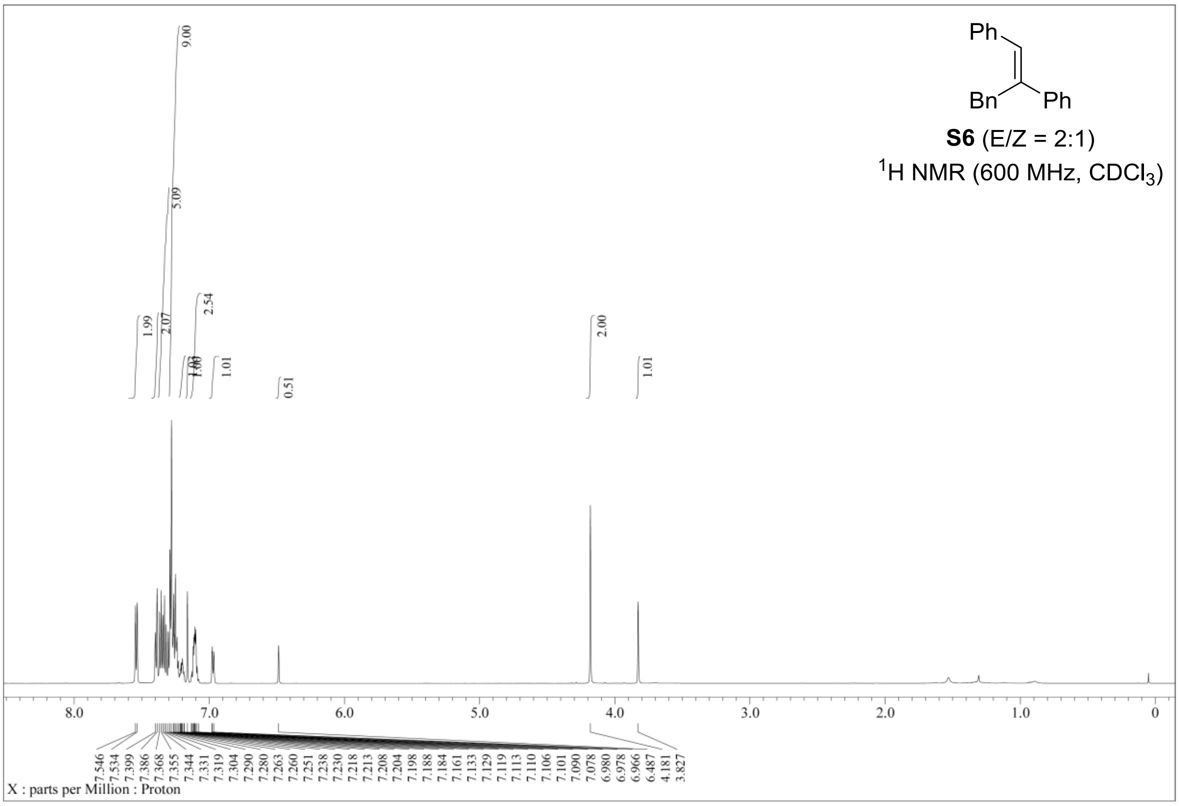
Ph
Bn
Ph
S6 (E/Z = 2:1)
1H NMR (600 MHz, CDCl3)
9.00
5.09
1.99
2.07
2.54
1.01
0.51
2.00
1.01
8.0 7.0 6.0 5.0 4.0 3.0 2.0 1.0 0
7.546 7.534 7.399 7.386 7.368 7.355 7.344 7.331 7.319 7.304 7.290 7.280 7.263 7.260 7.251 7.238 7.230 7.218 7.213 7.208 7.204 7.198 7.188 7.184 7.161 7.133 7.129 7.119 7.113 7.110 7.106 7.101 7.090 7.078 6.980 6.978 6.966 6.487 4.181 3.827
X : parts per Million : Proton

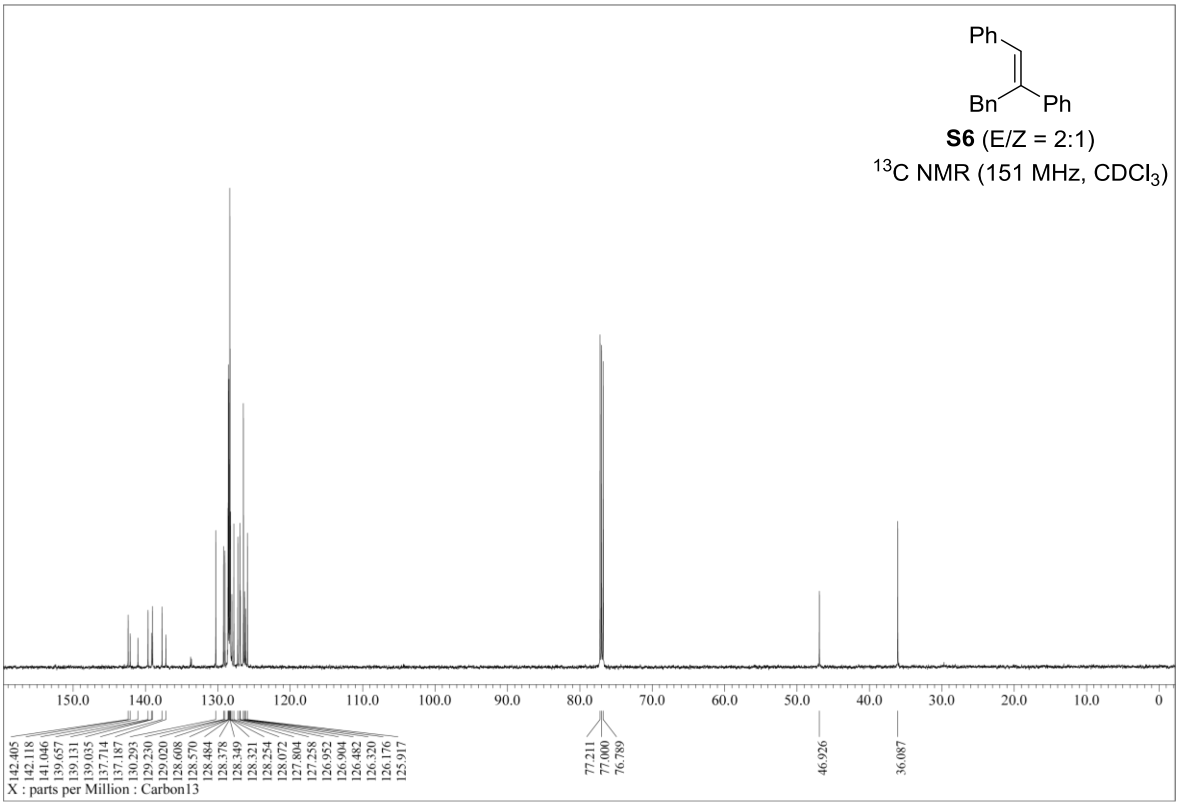
Ph
Bn
Ph
S6 (E/Z = 2:1)
13C NMR (151 MHz, CDCl3)
150.0 140.0 130.0 120.0 110.0 100.0 90.0 80.0 70.0 60.0 50.0 40.0 30.0 20.0 10.0 0
142.405 142.118 141.046 139.657 139.131 139.035 137.714 137.187 130.293 129.230 129.020 128.608 128.570 128.484 128.378 128.349 128.321 128.254 128.072 127.804 127.258 126.952 126.904 126.482 126.320 126.176 125.917
77.211 77.000 76.789
46.926
36.087
X : parts per Million : Carbon13

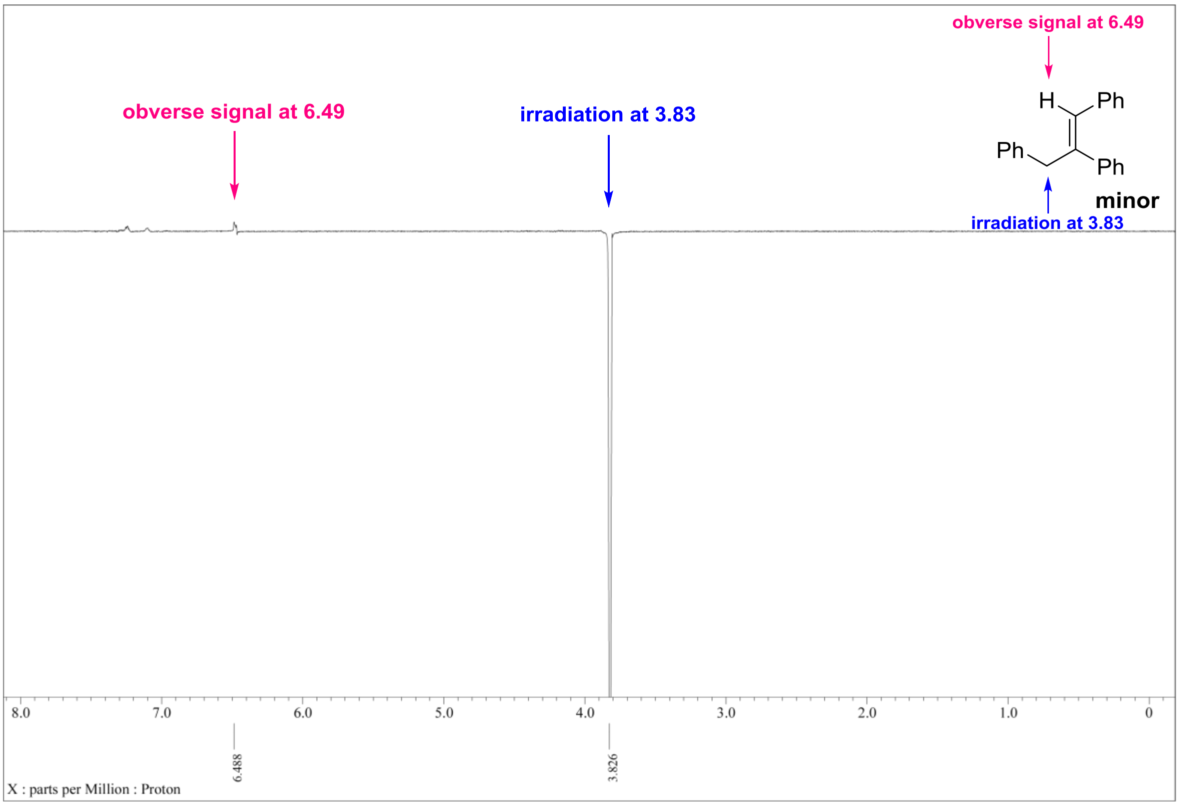

obverse signal at 6.49
irradiation at 3.83
obverse signal at 6.49
H
Ph
Ph
Ph
minor
irradiation at 3.83
8.0
7.0
6.0
5.0
4.0
3.0
2.0
1.0
0
6.488
3.826
X : parts per Million : Proton

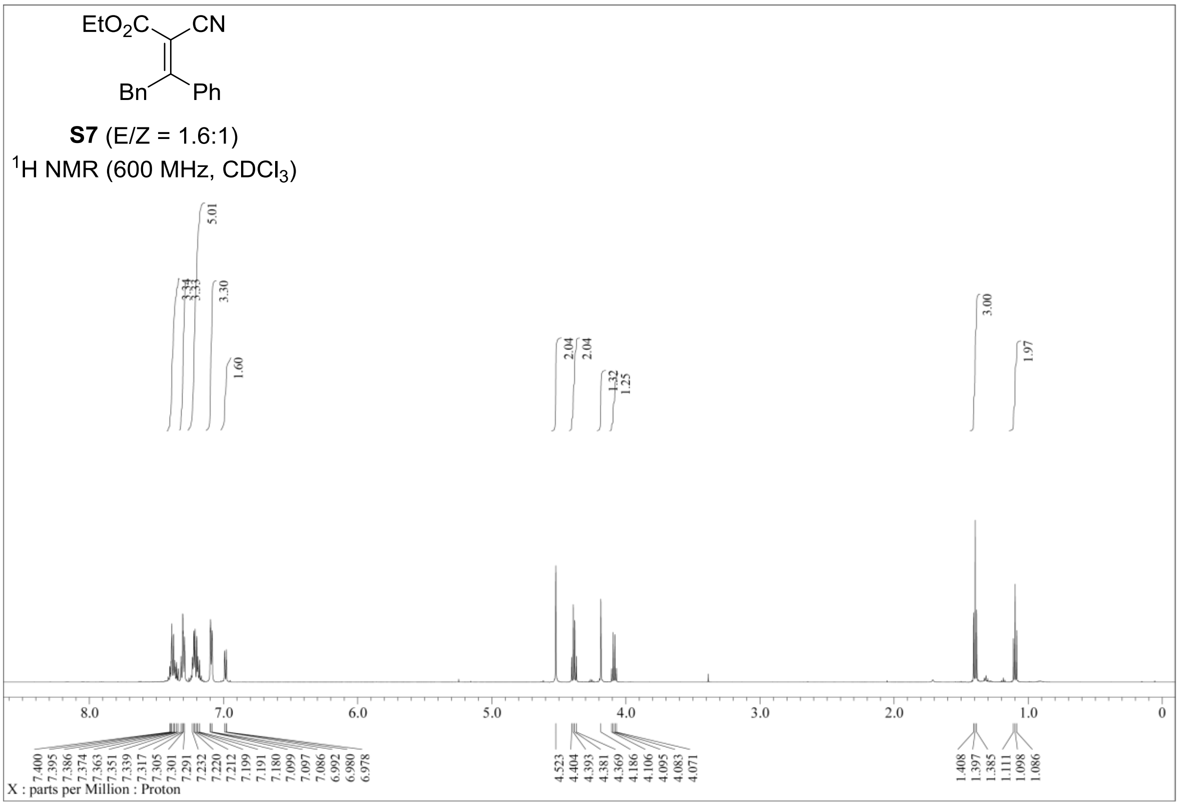

EtO2C
CN
Bn
Ph
S7 (E/Z = 1.6:1)
1H NMR (600 MHz, CDCl3)
5.01
3.34
3.33
3.30
1.60
2.04
2.04
1.32
1.25
3.00
1.97
8.0
7.0
6.0
5.0
4.0
3.0
2.0
1.0
0
7.400 7.395 7.386 7.374 7.363 7.351 7.339 7.317 7.305 7.301 7.291 7.232 7.220 7.212 7.199 7.191 7.180 7.099 7.097 7.086 6.992 6.980 6.978
4.523 4.404 4.393 4.381 4.369 4.186 4.106 4.095 4.083 4.071
1.408 1.397 1.385 1.111 1.098 1.086
X : parts per Million : Proton

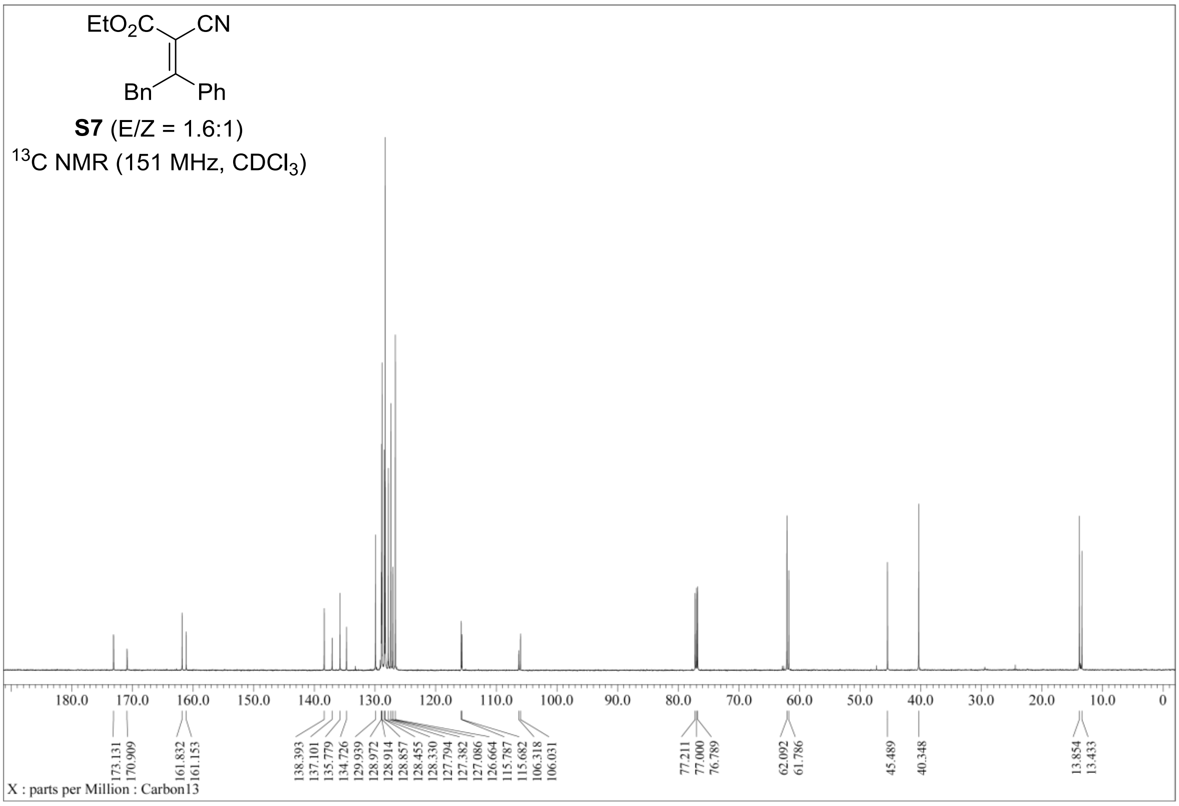
EtO2C CN
Bn Ph
S7 (E/Z = 1.6:1)
13C NMR (151 MHz, CDCl3)
173.131
170.909
161.832
161.153
138.393
137.101
135.779
134.726
129.939
128.972
128.914
128.857
128.455
128.330
127.794
127.382
127.086
126.664
115.787
115.682
106.318
106.031
77.211
77.000
76.789
62.092
61.786
45.489
40.348
13.854
13.433
X : parts per Million : Carbon13

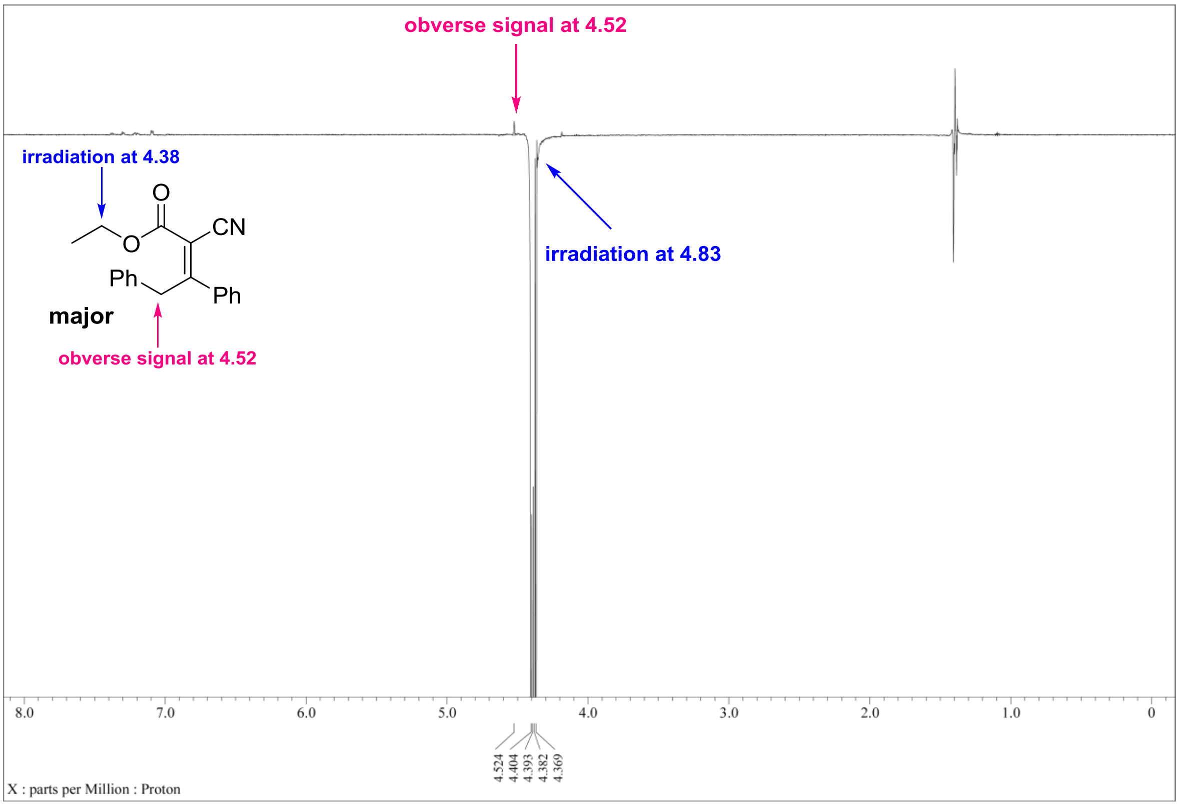
obverse signal at 4.52
irradiation at 4.38
irradiation at 4.83
O
CN
O
Ph
Ph
major
obverse signal at 4.52
4.524
4.404
4.393
4.382
4.369
X : parts per Million : Proton

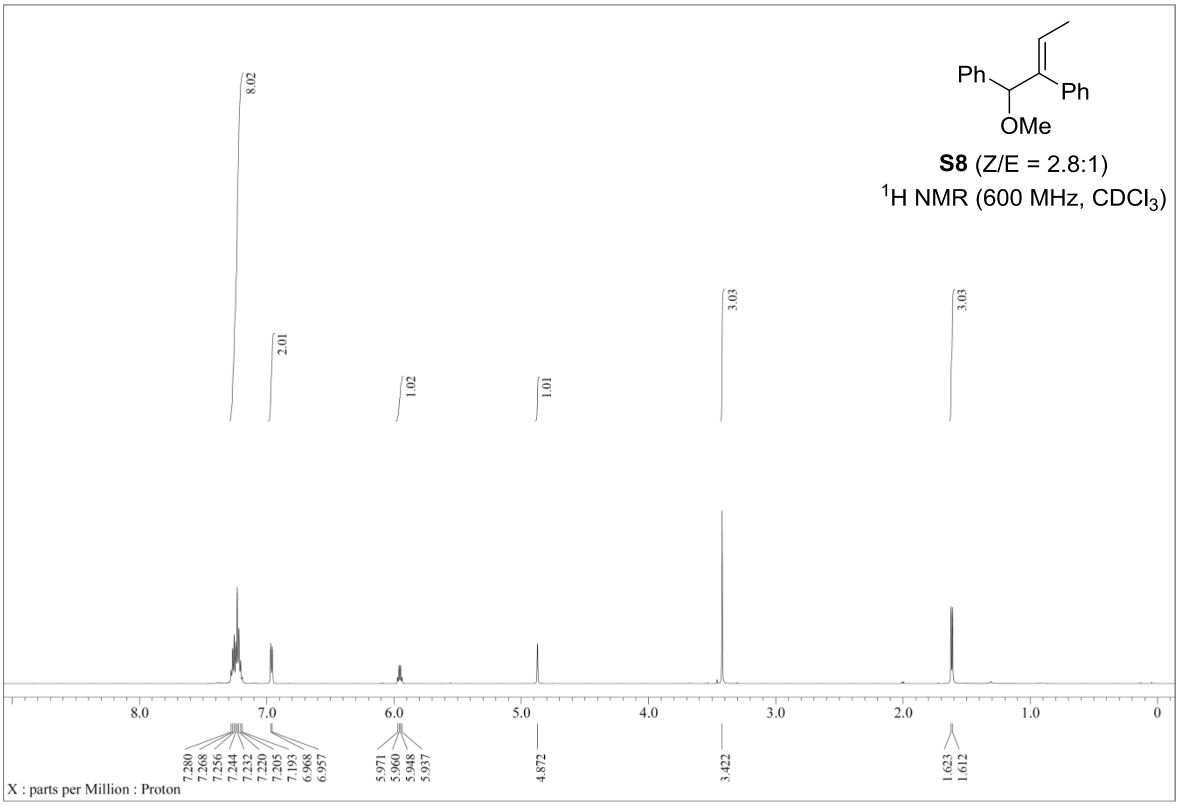

Ph
Ph
OMe
S8 (Z/E = 2.8:1)
1H NMR (600 MHz, CDCl3)
8.02
2.01
1.02
1.01
3.03
3.03
8.0
7.0
6.0
5.0
4.0
3.0
2.0
1.0
0
7.280
7.268
7.256
7.244
7.232
7.220
7.205
7.193
6.968
6.957
5.971
5.960
5.948
5.937
4.872
3.422
1.623
1.612
X : parts per Million : Proton

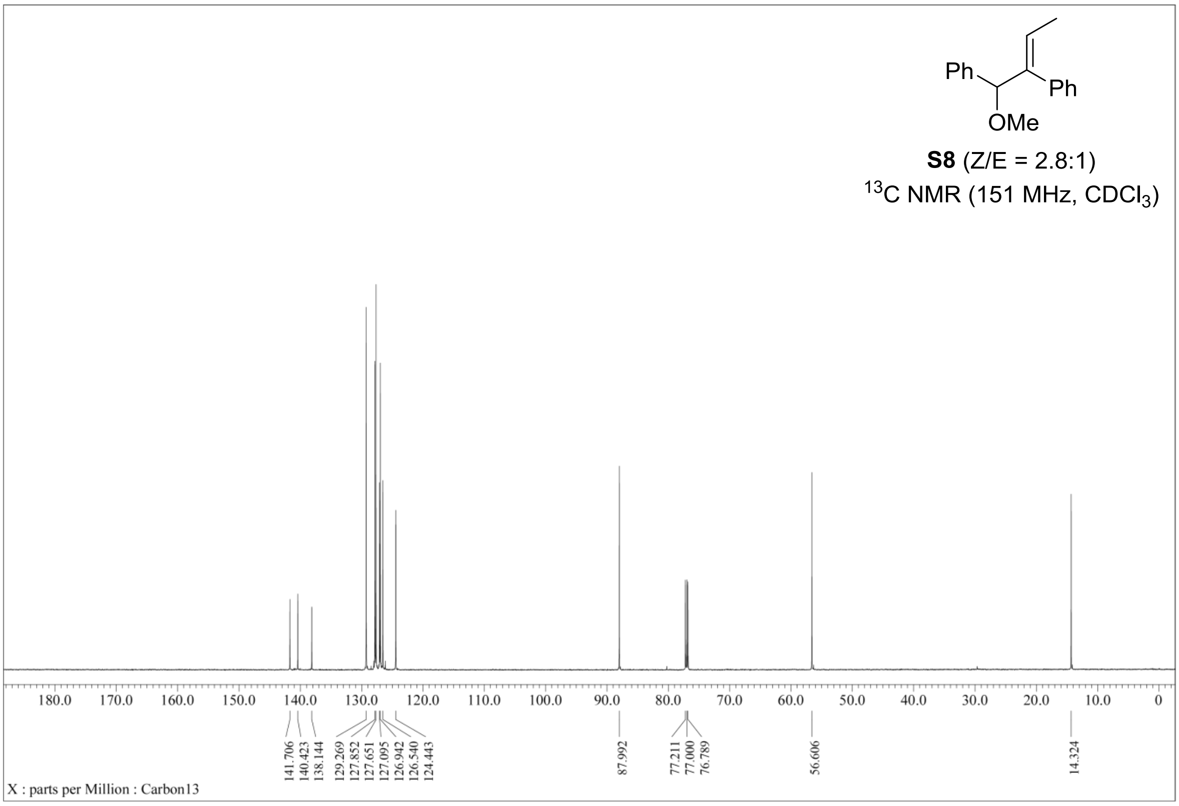

Ph
Ph
OMe
S8 (Z/E = 2.8:1)
13C NMR (151 MHz, CDCl3)
180.0
170.0
160.0
150.0
140.0
130.0
120.0
110.0
100.0
90.0
80.0
70.0
60.0
50.0
40.0
30.0
20.0
10.0
0
141.706
140.423
138.144
129.269
127.852
127.651
127.095
126.942
126.540
124.443
87.992
77.211
77.000
76.789
56.606
14.324
X : parts per Million : Carbon13

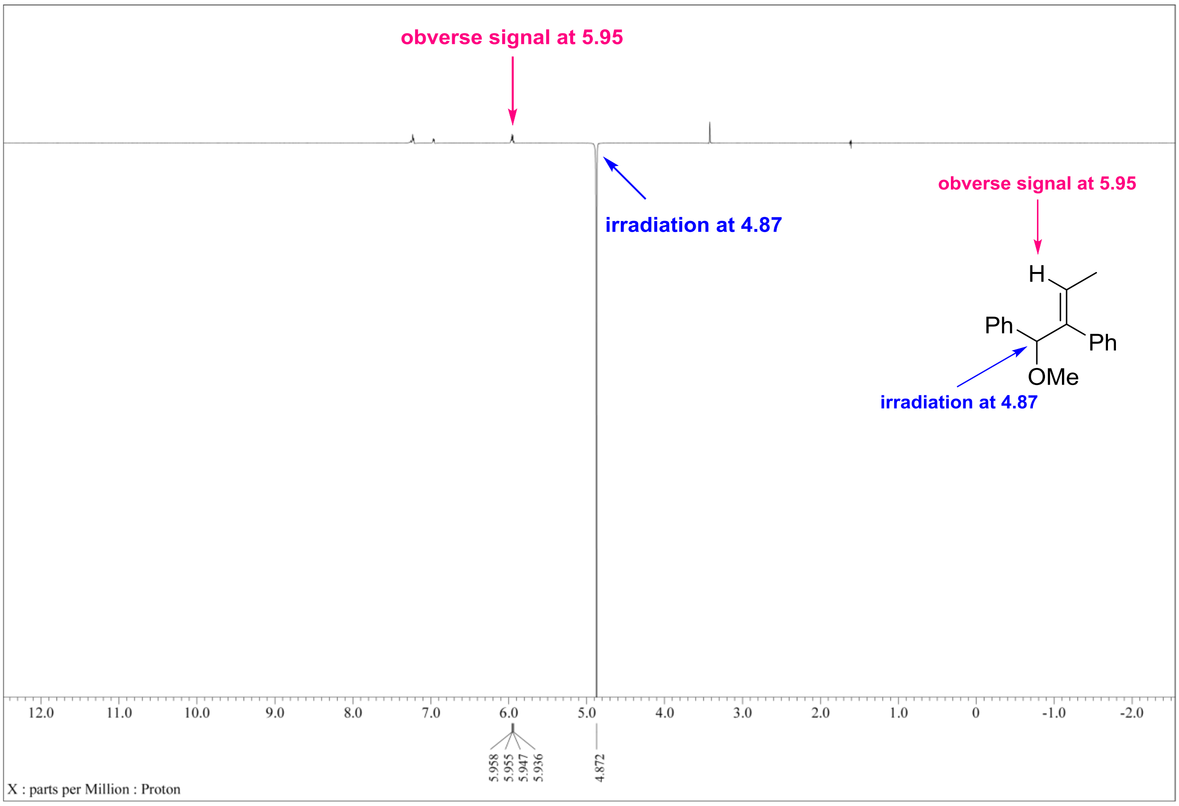
obverse signal at 5.95
irradiation at 4.87
obverse signal at 5.95
H
Ph
Ph
OMe
irradiation at 4.87
5.958
5.955
5.947
5.936
4.872
X : parts per Million : Proton

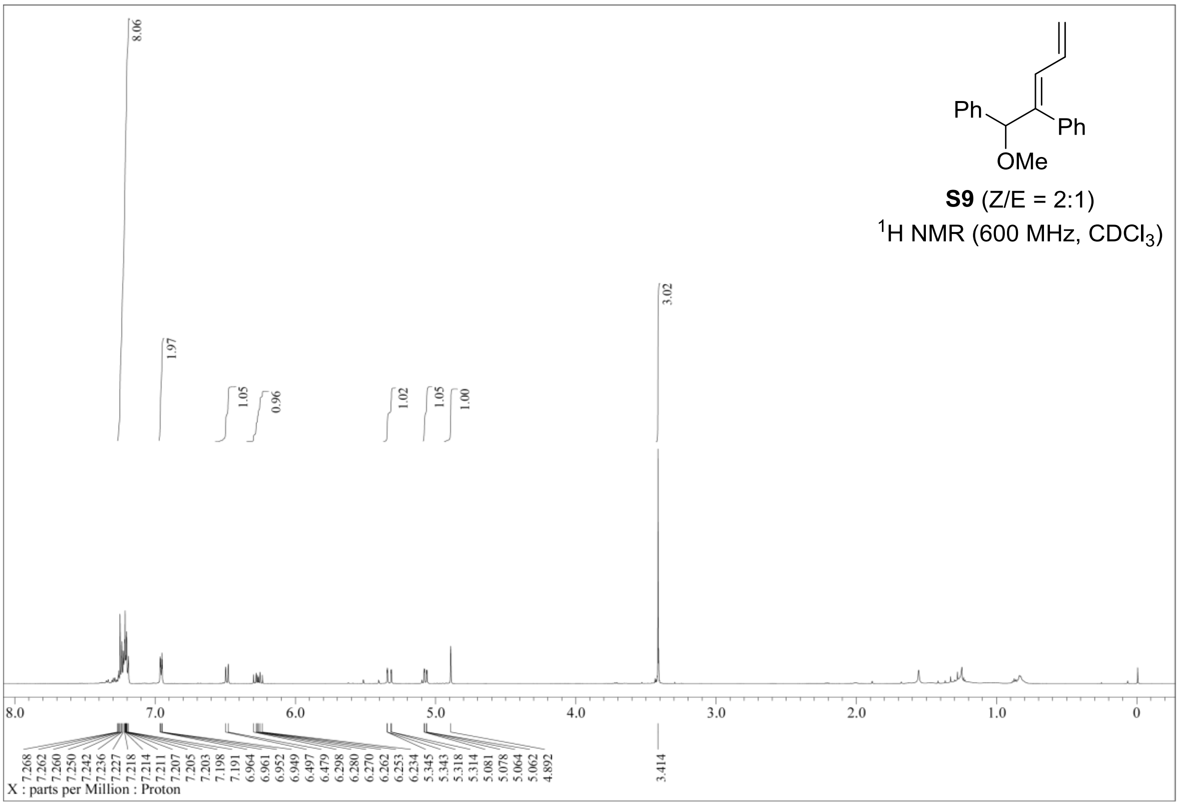
Ph
Ph
OMe
S9 (Z/E = 2:1)
1H NMR (600 MHz, CDCl3)
X : parts per Million : Proton

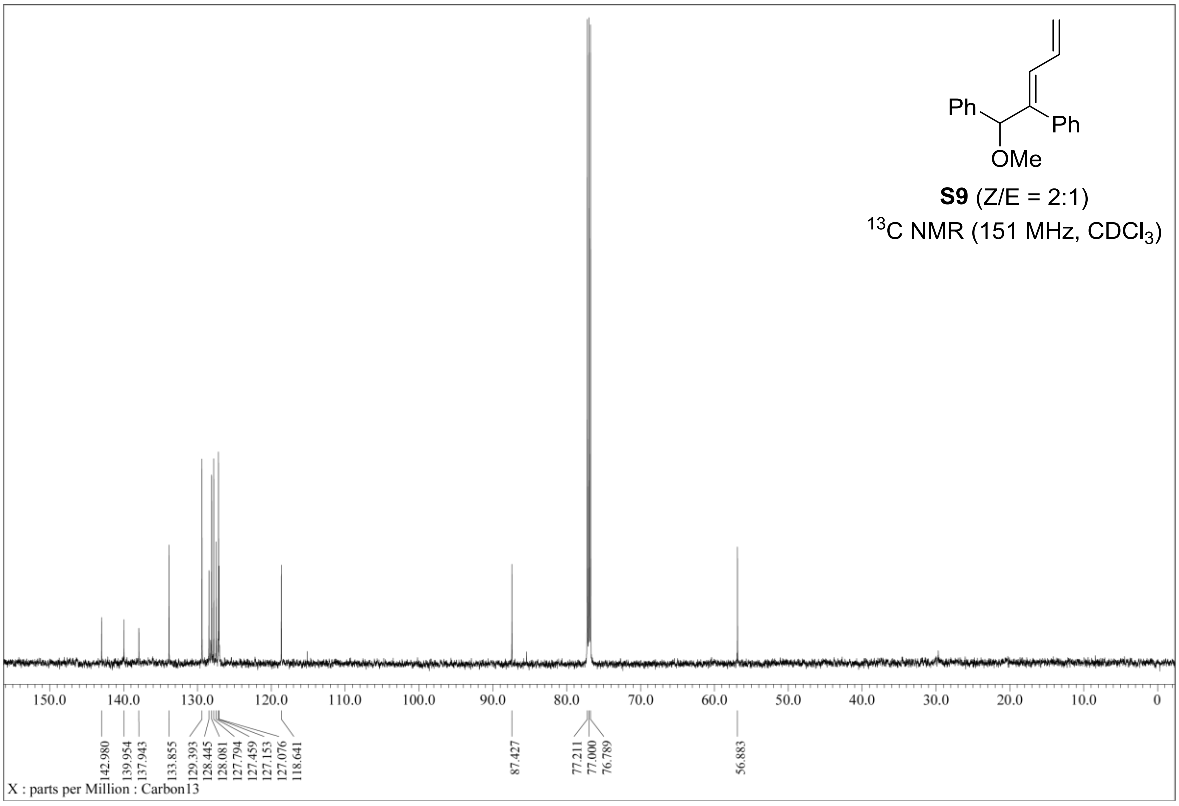
Ph
Ph
OMe
S9 (Z/E = 2:1)
13C NMR (151 MHz, CDCl3)
150.0
140.0
130.0
120.0
110.0
100.0
90.0
80.0
70.0
60.0
50.0
40.0
30.0
20.0
10.0
0
142.980
139.954
137.943
133.855
129.393
128.445
128.081
127.794
127.459
127.153
127.076
118.641
87.427
77.211
77.000
76.789
56.883
X : parts per Million : Carbon13

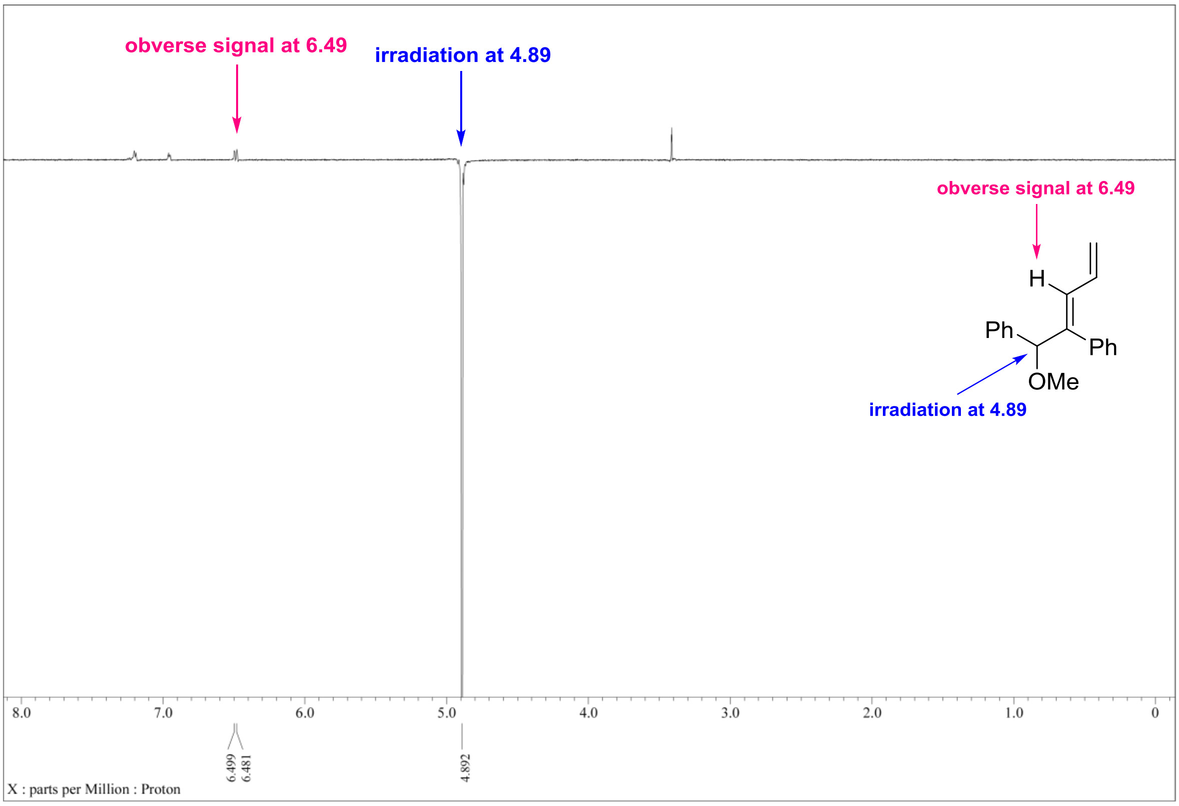
obverse signal at 6.49
irradiation at 4.89
obverse signal at 6.49
H
Ph
Ph
OMe
irradiation at 4.89
8.0
7.0
6.0
5.0
4.0
3.0
2.0
1.0
0
6.499
6.481
4.892
X : parts per Million : Proton

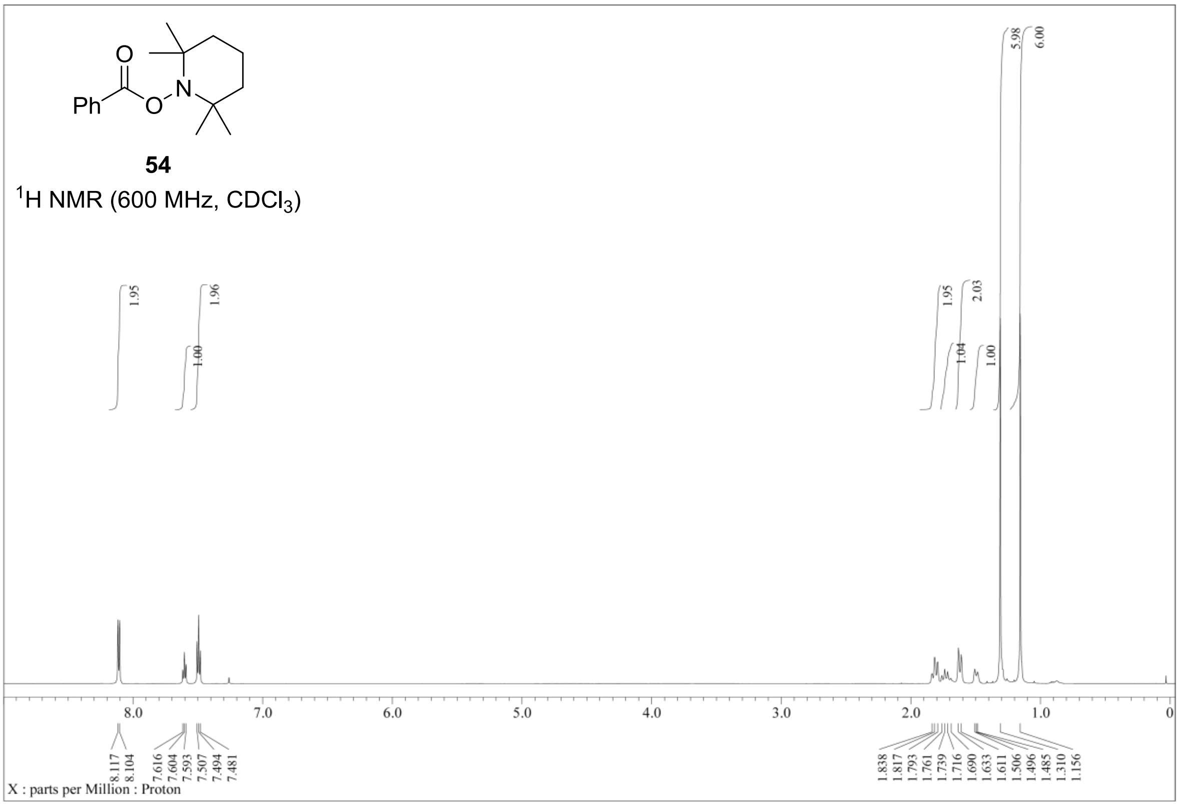
54
^{1}H NMR (600 MHz, CDCl$_3$)
X : parts per Million : Proton

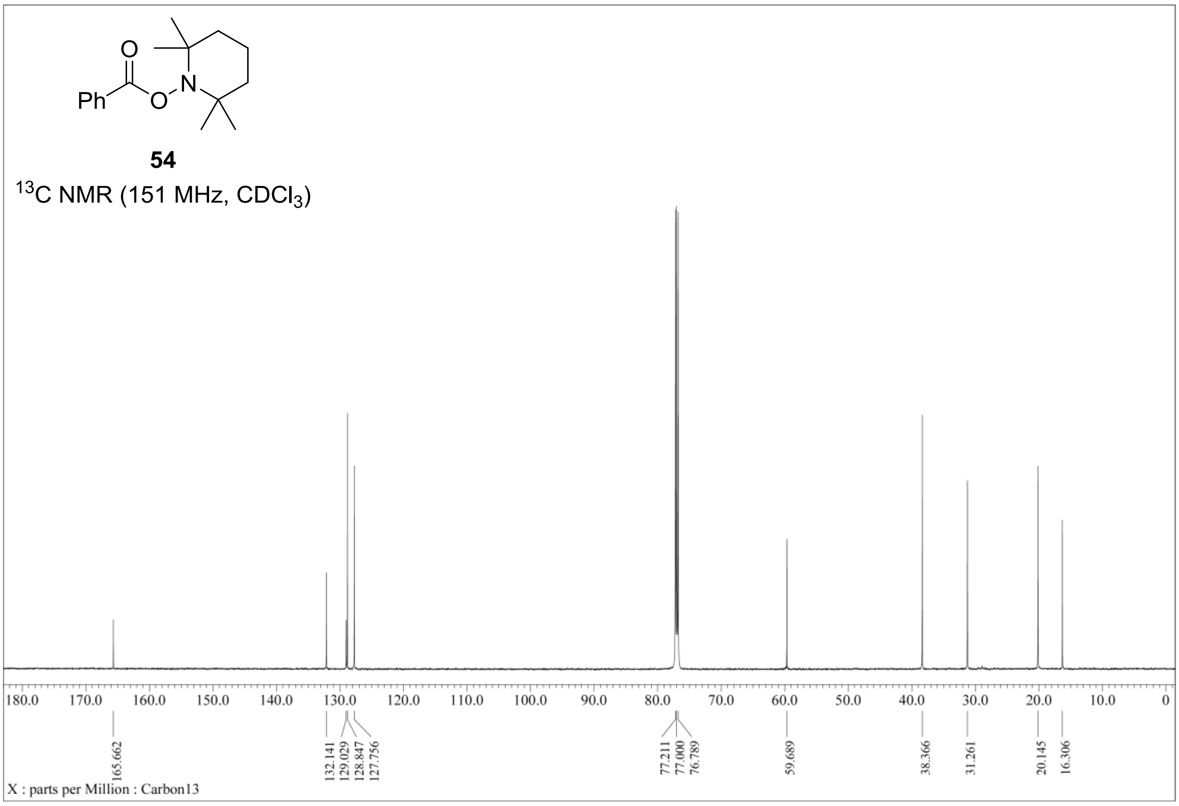
54
^{13}C NMR (151 MHz, CDCl$_3$)
X : parts per Million : Carbon13

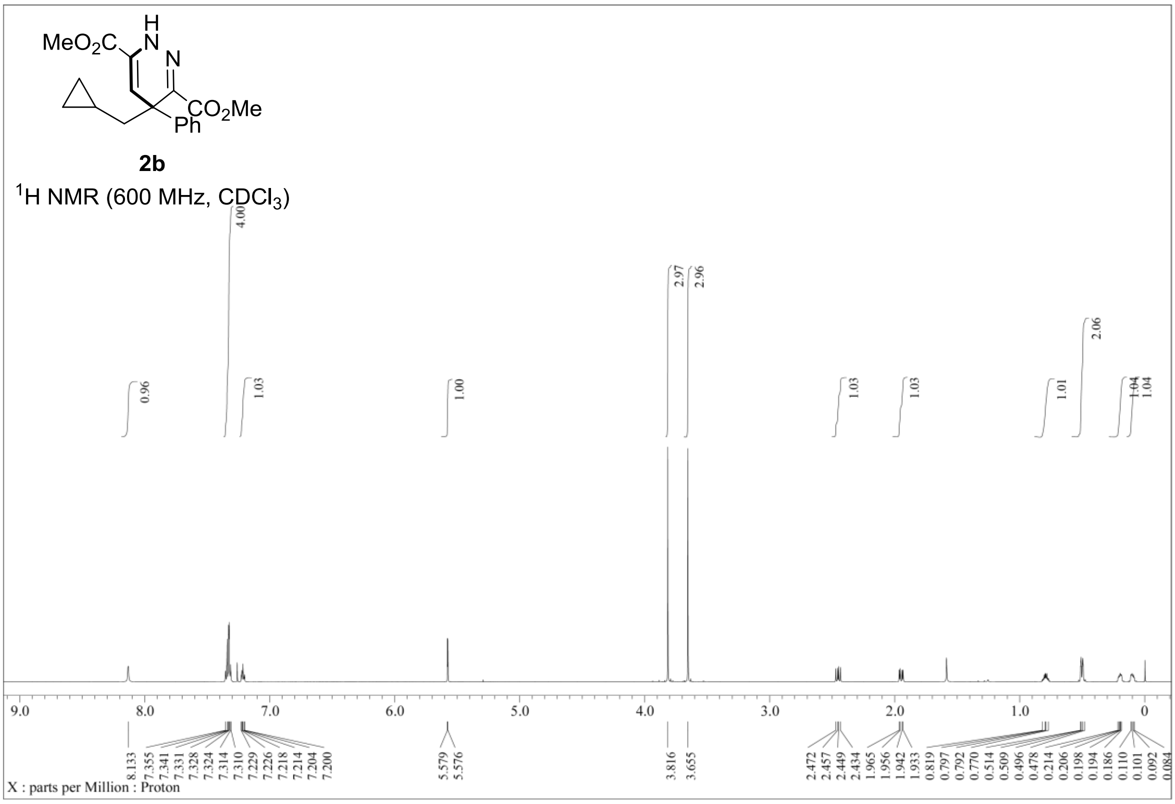
2b
1H NMR (600 MHz, CDCl3)
0.96
4.00
1.03
1.00
2.97
2.96
1.03
1.03
1.01
2.06
1.04
1.04
8.133
7.355
7.341
7.331
7.328
7.324
7.314
7.310
7.229
7.226
7.218
7.214
7.204
7.200
5.579
5.576
3.816
3.655
2.472
2.457
2.449
2.434
1.965
1.956
1.942
1.933
0.819
0.797
0.792
0.770
0.514
0.509
0.496
0.478
0.214
0.206
0.198
0.194
0.186
0.110
0.101
0.092
0.084
X : parts per Million : Proton

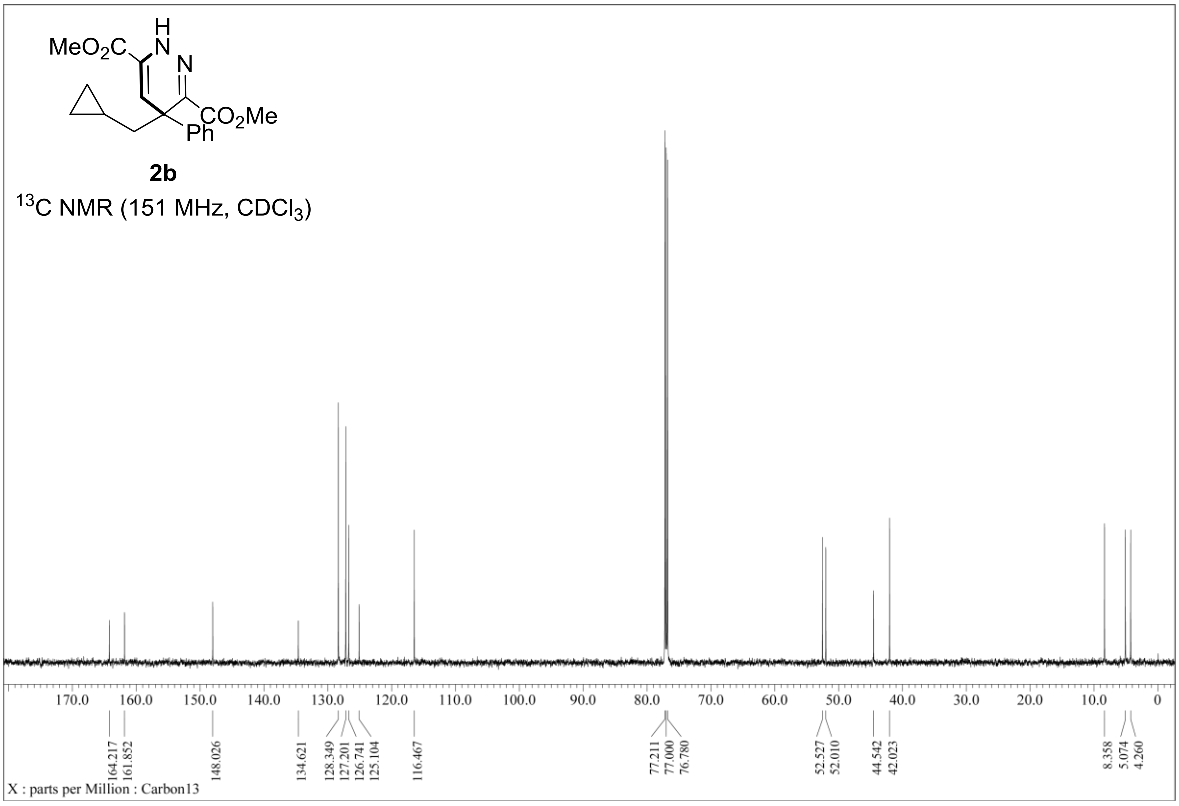
2b
13C NMR (151 MHz, CDCl3)
164.217
161.852
148.026
134.621
128.349
127.201
126.741
125.104
116.467
77.211
77.000
76.780
52.527
52.010
44.542
42.023
8.358
5.074
4.260
X : parts per Million : Carbon13

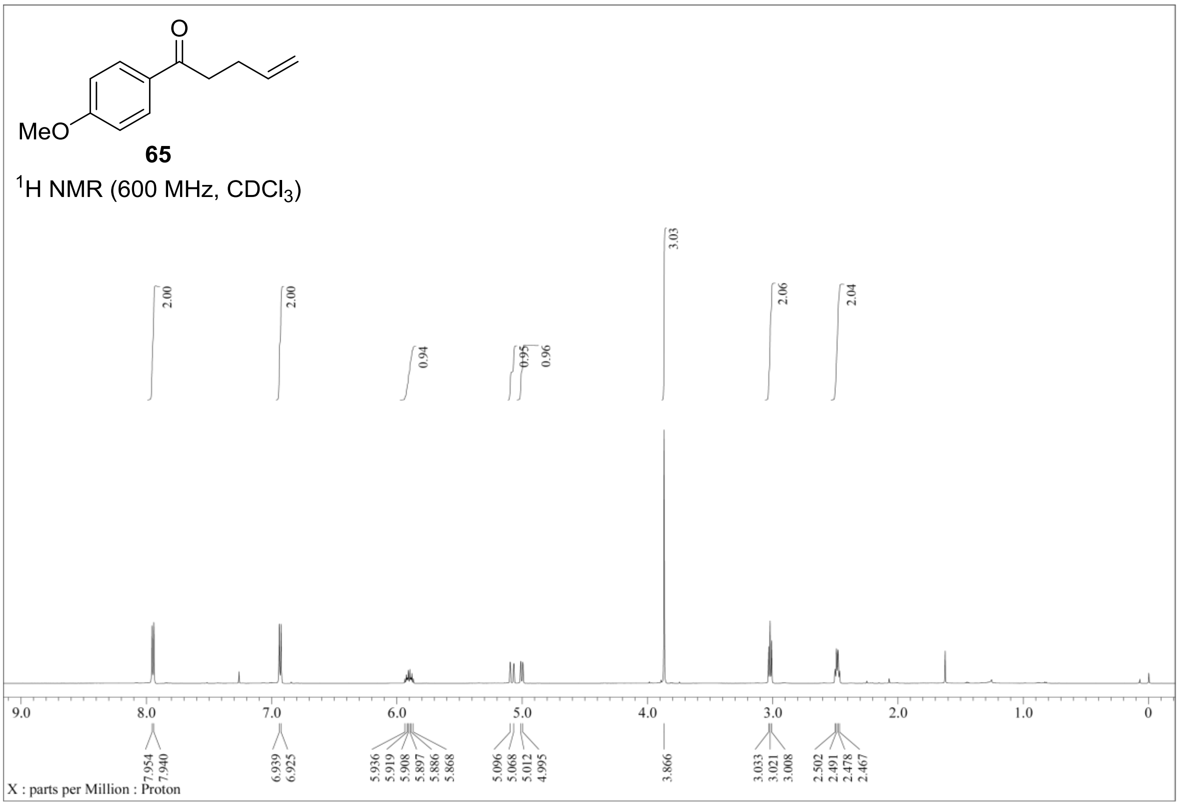
MeO
O
65
^{1}H NMR (600 MHz, $CDCl_3$)
X : parts per Million : Proton

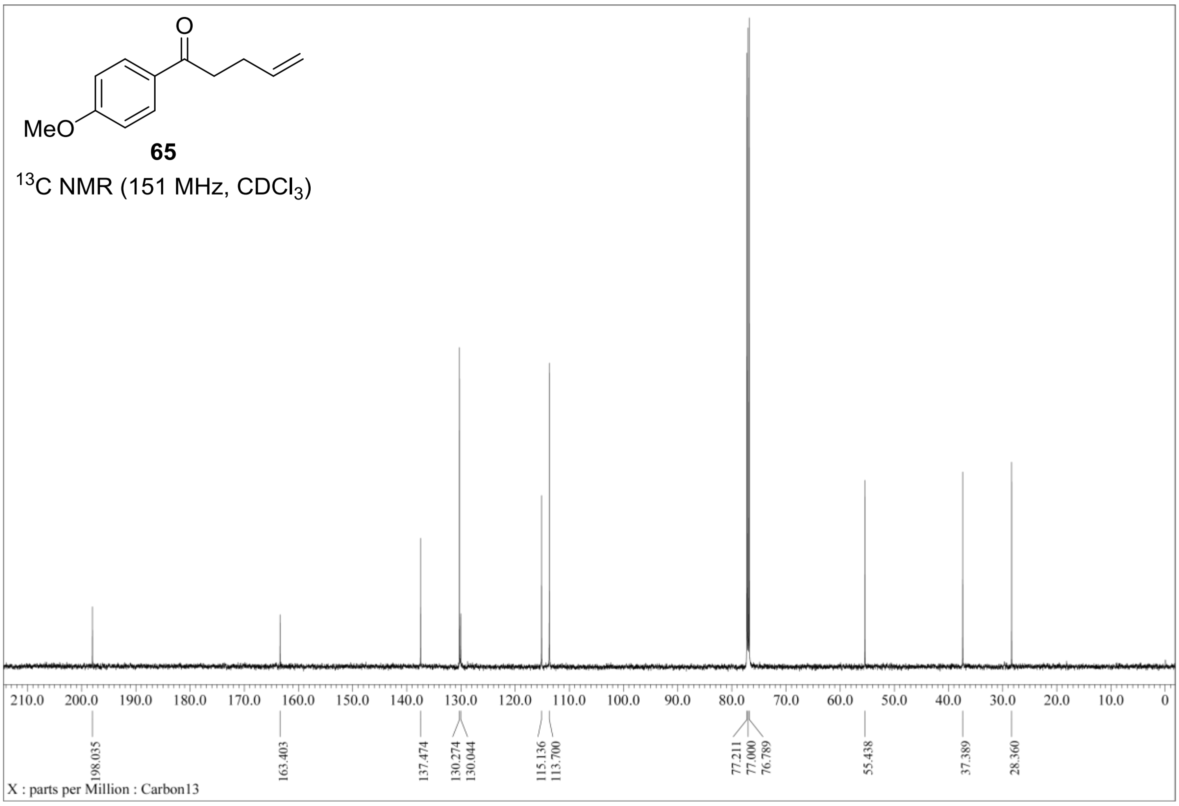
MeO
O
65
^{13}C NMR (151 MHz, $CDCl_3$)
X : parts per Million : Carbon13
